# Structure-Based Optimization of Covalent, Small-Molecule Stabilizers of the 14-3-3σ/ERα Protein–Protein Interaction from Nonselective Fragments

**DOI:** 10.1021/jacs.3c05161

**Published:** 2023-09-07

**Authors:** Markella Konstantinidou, Emira J. Visser, Edmee Vandenboorn, Sheng Chen, Priyadarshini Jaishankar, Maurits Overmans, Shubhankar Dutta, R. Jeffrey Neitz, Adam R. Renslo, Christian Ottmann, Luc Brunsveld, Michelle R. Arkin

**Affiliations:** † Department of Pharmaceutical Chemistry and Small Molecule Discovery Center (SMDC), University of California, San Francisco, California 94143, United States; ‡ Laboratory of Chemical Biology, Department of Biomedical Engineering and Institute for Complex Molecular Systems (ICMS), Eindhoven University of Technology, 5600 MB Eindhoven, The Netherlands

## Abstract

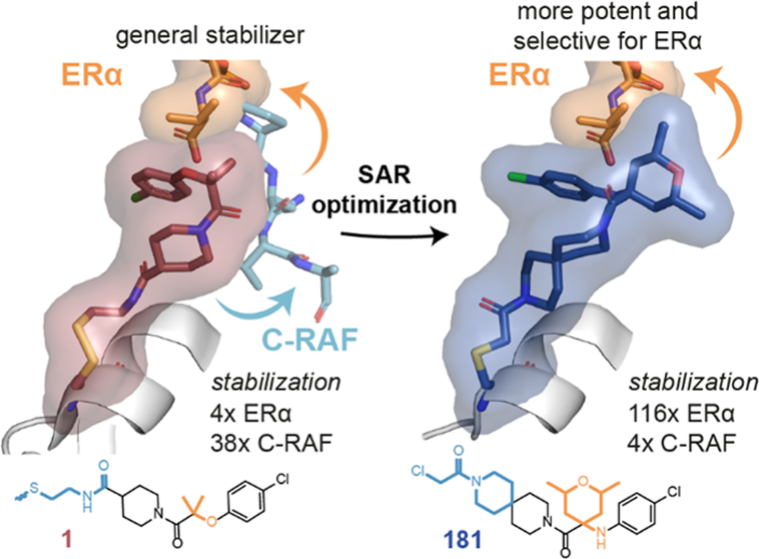

The stabilization of protein–protein interactions (PPIs) has emerged as a promising strategy in chemical biology and drug discovery. The identification of suitable starting points for stabilizing native PPIs and their subsequent elaboration into selective and potent molecular glues lacks structure-guided optimization strategies. We have previously identified a disulfide fragment that stabilized the hub protein 14-3-3σ bound to several of its clients, including ERα and C-RAF. Here, we show the structure-based optimization of the nonselective fragment toward selective and highly potent small-molecule stabilizers of the 14-3-3σ/ERα complex. The more elaborated molecular glues, for example, show no stabilization of 14-3-3σ/C-RAF up to 150 μM compound. Orthogonal biophysical assays, including mass spectrometry and fluorescence anisotropy, were used to establish structure–activity relationships. The binding modes of 37 compounds were elucidated with X-ray crystallography, which further assisted the concomitant structure-guided optimization. By targeting specific amino acids in the 14-3-3σ/ERα interface and locking the conformation with a spirocycle, the optimized covalent stabilizer **181** achieved potency, cooperativity, and selectivity similar to the natural product Fusicoccin-A. This case study showcases the value of addressing the structure, kinetics, and cooperativity for molecular glue development.

## Introduction

Protein–protein interactions (PPIs) play a central role in biological networks and are often dysregulated in pathological conditions.^1−3^ PPIs are considered particularly difficult targets for small-molecule modulation due to the large, usually hydrophobic, surfaces, the lack of suitable deep pockets, and the absence of known starting points from nature or high-throughput screening.^4^ Despite such challenges, both inhibition and stabilization of PPIs have emerged as attractive strategies in chemical biology and drug discovery.^5−7^ In particular, PPI stabilization has become a viable approach for targeting “undruggable” protein targets.^8−10^ Such stabilizers include “molecular glues” that bind to the composite surface between two proteins, and bivalent molecules, such as proteolysis-targeting chimeras (PROTACs), which induce proximity between two proteins that do not otherwise interact. Molecular glues can have various functions, including degradation of the target protein, as with the IMiDs, or augmentation of a native complex, as described for natural products and synthetic compounds that stabilize complexes between 14-3-3 and its partners.^11,12^

Particularly interesting challenges within PPI modulator discovery are the “hub” proteins that have the ability to interact with numerous protein clients.^13−15^ The extensive interactome of hub proteins provides tremendous potential for drug discovery, but at the same time raises the question of selective targeting. Not only might the underlying biology be intertwined but also the molecular recognition principles within a hub protein’s PPI network might be based on similar chemical motifs. In this work, we focus on the hub protein 14-3-3, a highly abundant adaptor and scaffolding protein that binds to hundreds of phosphorylated and mostly intrinsically disordered protein domains.^16,17^ In humans, 14-3-3 is present via seven highly conserved isoforms with seemingly overlapping functions.^18,19^ We aim to show that despite the vast number of 14-3-3 clients, selective small-molecule stabilizers, or molecular glues,^20,21^ can be systematically developed by targeting the chemically unique composite binding pocket formed by a given 14-3-3/client PPI interface.

14-3-3 is a dimeric hub protein that binds to its clients via their phospho-serine or phospho-threonine sites and upon binding creates order in these disordered client regions.^22,23^ 14-3-3 is involved in the regulation of transcription factors, cell signaling, cell cycle progression, signal-transduction pathways, and protein stability.^18,22,24−28^ Among 14-3-3 clients, there are many proteins of high therapeutic interest, including estrogen receptor α (ERα),^29^ several proteins in the RAS/MAPK pathway, such as the RAF kinases,^30−32^ transcription factors,^33−36^ and proteins associated with neurodegeneration pathways, such as LRRK2,^37,38^ tau,^39,40^ and α-synuclein.^41,42^

Our research goal is to develop platforms for the systematic discovery of molecular glues using 14-3-3 as a structurally tractable and biologically fascinating hub protein. Here, we demonstrate the selective stabilization of 14-3-3σ interactions with ERα via small molecules that act as orthosteric molecular glues. Blocking the function of ERα is a well-established strategy for targeting breast cancer and generally includes small molecules that inhibit ERα ligand binding at its ligand-binding pocket.^43^ Despite their successes, drug resistance is often encountered.^44,45^ 14-3-3 binds at the extreme C-terminus of ERα via recognition of its penultimate threonine phosphorylation (Thr594), thereby suppressing the transcriptional activity of ERα and concomitant breast cancer cell proliferation.^29^ Stabilization of this PPI by the natural product fusicoccin (FC-A) already demonstrated the ligandability of this novel interface. However, the chemical complexity of the natural product as well as the difficulties in isolation, or the development of semisynthetic approaches,^46−48^ limit its usefulness as a platform for systematic glue discovery. Additionally, FC-A and its semisynthetic analogues are promiscuous 14-3-3/client molecular glues and stabilize multiple 14-3-3 clients,^49−51^ including ERα.^29^

The idea of selective stabilization of 14-3-3 PPIs has recently received strong attention, but the identification of novel molecular glue stabilizers has been very challenging, a concept that extends to most PPI networks.^52−54^ In the urgent quest to explore new chemical matter for molecular glues, fragment-based approaches have recently been explored. For 14-3-3, screens have typically used phosphopeptides derived from the intrinsically disordered domains of client proteins. For example, a crystallography-based fragment screen identified amidine fragments that, although weak stabilizers, selectively bound to 14-3-3/p53-peptide or 14-3-3/TAZ-peptide complexes.^55^ A second crystallography screen identified aldehyde-containing fragments that targeted a conserved lysine residue on 14-3-3 in close proximity to the client protein binding site and stabilized the 14-3-3/p65 complex.^56^ Disulfide tethering^57,58^ has been applied both to cysteine residues on 14-3-3 and on the client phosphopeptide to identify disulfide-bound fragment stabilizers of 14-3-3/ERα^59^ and ERRγ, respectively.^60,61^

**Figure 1:**
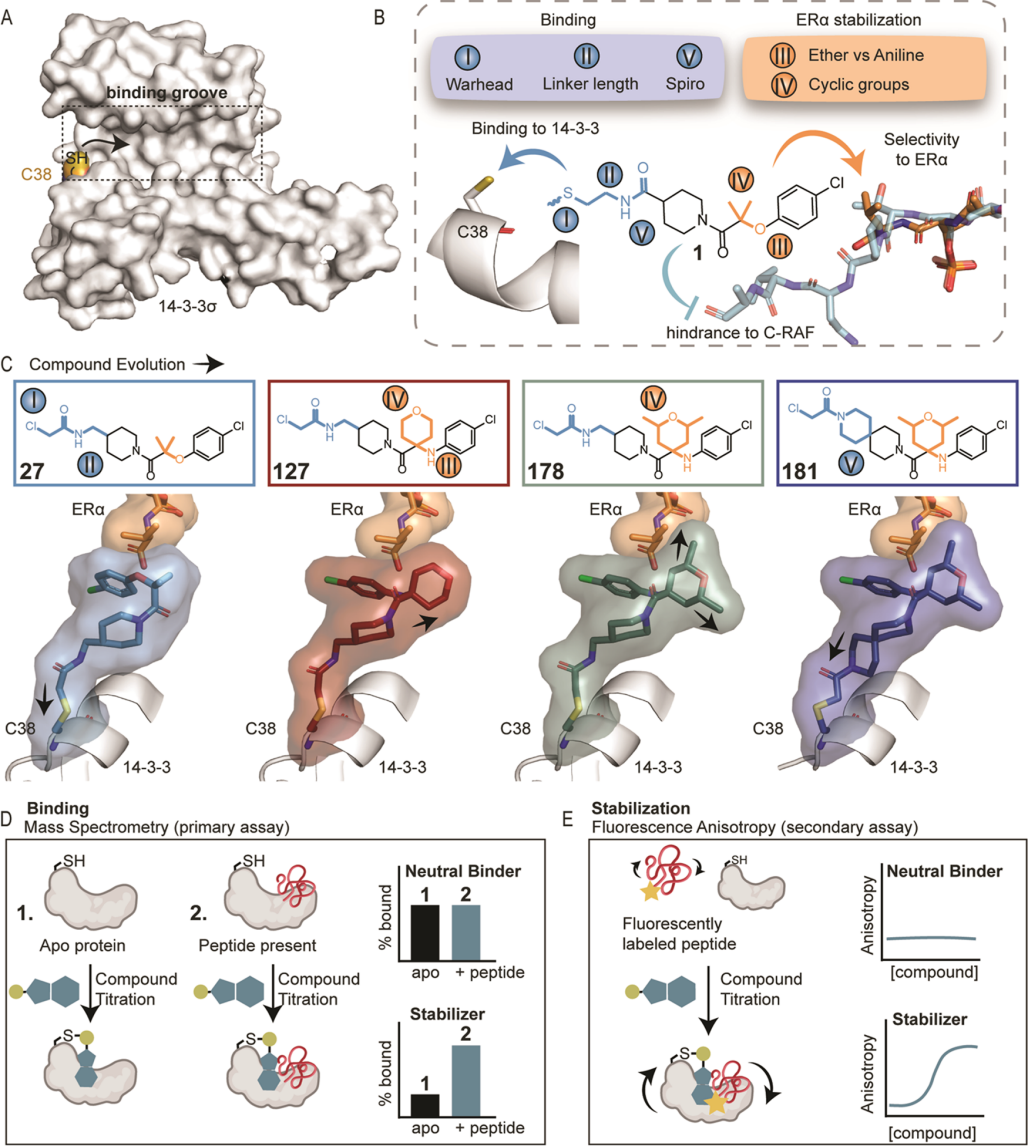
Overview of the structure-based optimization approach. (A) Binding groove of 14-3-3σ (gray surface). The position of the native cysteine (Cys38) is indicated in yellow. (B) Chemical structure of the nonselective **fragment 1** and key structural modifications (I–V) explored during the chemical optimization, aiming to increase the cooperativity with 14-3-3σ/ERα (orange peptide) and reduce the stabilization of 14-3-3σ/C-RAF (blue peptide). (C) Examples of compound evolution and X-ray crystallographic data starting from the disulfide **fragment 1** and resulting in potent and selective stabilizer **181**. (D) Overview of the mass spectrometry assay (primary assay). Compound titrations were performed in the absence of the peptide to determine % binding to 14-3-3σ (apo screening, D1) and then in the presence of both 14-3-3σ and the peptide, as an indirect indication of stabilization (D2). Compounds that showed % bound D2 > D1 were classified as stabilizers, whereas compounds for which % bound did not change significantly between D1 and D2 were classified as neutral binders. (E) Overview of the fluorescence anisotropy assay (secondary assay). Compound titrations were performed in the presence of 14-3-3σ (1 μM for ERα, 5 μM for C-RAF) and FAM-labeled peptides (10 nM). In the case of stabilizers, a dose-dependent increase in anisotropy was observed. No significant increase was observed for neutral binders.

We recently expanded the disulfide-tethering study to develop 14-3-3σ/client glues for peptides with diverse shapes and binding modes.^62^ The native Cys38 at the periphery of the peptide-binding groove on 14-3-3σ (Figure 1A) was targeted with a library consisting of ∼1600 disulfide fragments. Both client-selective and broadly stabilizing fragments were identified. For example, the nonselective disulfide **fragment 1** stabilized both 14-3-3σ/ERα and 14-3-3σ/C-RAF peptide complexes (Figure S1A). Although the disulfide fragment preferentially stabilized C-RAF (38-fold stabilization for C-RAF and 4-fold stabilization for ERα at 100 μM compound), the crystal structures showed a similar binding mode with both clients. Unlike the C-terminal motif on ERα, C-RAF binds to 14-3-3σ with an internal sequence centered on phospho-Ser259; this site is also a relevant onco-target.^63^

In this work, we report the structure-guided chemical optimization of this nonselective disulfide hit **fragment 1** toward small-molecule covalent stabilizers that preferentially bind the 14-3-3σ/ERα complex over 14-3-3σ/C-RAF. Because the de novo discovery and optimization of molecular glues is an emerging field, we describe our strategy and the molecular recognition between glues and the PPI in detail. Chemical modifications were strategically evaluated with a focus on increasing the cooperativity with 14-3-3σ/ERα and reducing stabilization of 14-3-3σ/C-RAF via increased steric hindrance (Figure 1B). The resulting optimized compound **181** is the first covalent compound to demonstrate selective stabilization of a 14-3-3 client complex. The degree of stabilization of the 14-3-3σ/ERα complex (116-fold) and EC_50_ value (1 μM) are similar to that of the natural product Fusiccocin-A. This strategy proposes a design framework for systematic optimization of client-selective molecular glues.

## Results and Discussion

Chemical modifications on the original disulfide fragment **1** can be divided into five groups (Figure 1B,C). Modifications (I) and (II), at the periphery of the 14-3-3 binding groove, included the replacement of the reversible disulfide tether with irreversible electrophiles (I) with varying linker lengths (II). Modifications (III) and (IV) at the 14-3-3/ERα interface focused on optimizing the substituents in close proximity to the client peptides, aiming to increase stabilization and selectivity via specific interactions with the client of 14-3-3. The last modification (V) was the rigidification of the linker, aiming to “lock” the compounds’ conformations, resulting in the best stabilizer of the series, compound **181**. Representative examples of the compound evolution and their cocrystal structures with 14-3-3σ/ERα are shown in Figure 1C.

Two orthogonal assays were developed for screening. For the primary assay, mass spectrometry (MS) was used to monitor the formation of the covalent bond between the compound and the native cysteine (Cys38 on 14-3-3σ) (Figure 1D). The assay was first performed in the absence of peptide to determine binding to 14-3-3σ only (apo screening, D1) and then in the presence of both 14-3-3σ and the peptide as an indication of how much the peptide stabilized compound binding (D2). To normalize the amount of 14-3-3σ/client complex, peptides were included at two times their dissociation constant (2 × *K*_D_), e.g., 2 μM for ERα phosphopeptide and 18 μM for C-RAF phosphopeptide. Compounds that bound better in the presence of peptide than in the absence were classified as stabilizers, whereas compounds for which % bound did not change significantly were classified as neutral binders. As a secondary assay, fluorescence anisotropy (FA) was used. In this case, compounds were titrated to mixtures of 14-3-3σ and FAM-labeled peptides. For stabilizers, a dose-dependent increase in anisotropy was observed with a corresponding EC_50_ value, whereas for neutral binders, no significant increase occurred (Figure 1E). Thus, the two assays are highly complementary; the MS assay reports on compound binding and the FA assay reports on peptide binding.

### Replacement of the Disulfide Warhead with Electrophiles and the Effect of the Linker Length

**Figure 2:**
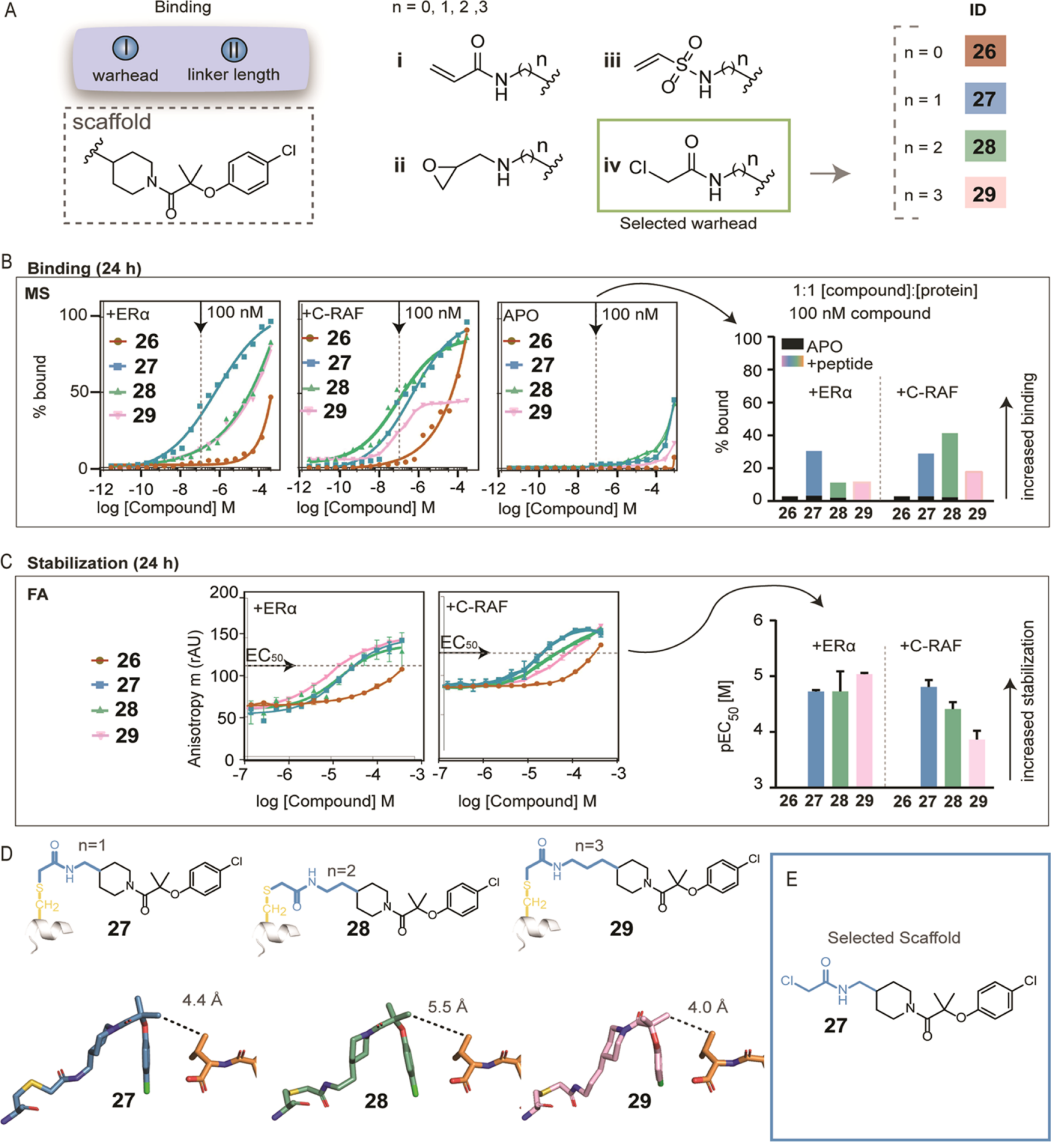
Warhead and linker length optimization. (A) Chemical modifications I and II focused on replacing the disulfide tether with covalent warheads (i–iv) with varying linker lengths. (B) Mass spectrometry dose–response curves for chloroacetamides (iv) with varying linker lengths (*n* = 0–3) after overnight incubation with 14-3-3σ/ERα, 14-3-3σ/C-RAF, and 14-3-3 alone (apo). Bar graphs of the mass spectrometry data at 100 nM compound concentration (1:1 ratio with the protein concentration), including the apo binding (plotted in black). (C) Fluorescence anisotropy (FA) dose–response curves for the chloroacetamides with varying linker lengths (*n* = 0–3) after overnight incubation with 14-3-3σ/ERα and 14-3-3σ/C-RAF. Bar graphs of FA compound titration pEC_50_ values. (D) Crystal structures of chloroacetamide analogues (iv) with varying linker lengths in complex with 14-3-3σ/ERα. Compounds are shown as blue, green, and pink sticks; the C-terminus of ERα phosphopeptide is shown as orange sticks. (E) Selected scaffold for further chemical optimization.

The first step of our strategy to develop selective, covalent stabilizers was the replacement of the disulfide tether with electrophiles. It is well-established for covalent inhibitors that cysteine-reactive electrophiles vary in their reactivity, selectivity, and kinetics.^64−66^ Therefore, we introduced four electrophilic warheads (acrylamides (i), oxiranes (ii), vinylsulfonamides (iii), and chloroacetamides (iv)) with varying linker lengths (Figure 2A). Both MS and FA assays showed that only some of the four warheads and linker lengths were tolerated. Compounds containing oxiranes and acrylamides were inactive; they did not label the protein in the MS assay, nor did they increase peptide binding to 14-3-3 in the FA assay (data not shown). Most of the vinylsulfonamides were also inactive, with the exception of the 1C-linker (compound **17**), which showed a weak stabilization effect (FA EC_50_ = 33 ± 1 μM for ERα; Figure S1B). By contrast, the compounds containing a chloroacetamide warhead showed both binding and stabilization of ERα and C-RAF phosphopeptides.

To visualize and easily compare the dose–response from MS and FA graphs, fixed values in their dose–response curves were depicted as bar graphs. For MS, we focused on the % bound at 100 nM of compound (1:1 [compound]:[protein]) (Figure 2B), and for FA, we plotted the negative log EC_50_ values, including the standard deviation from three independent experiments as error bars (pEC_50_, Figure 2C).

Linker length had a significant effect in the chloroacetamide series (Figures 2B,C and S2). The analogue with the shortest linker (*n* = 0) (**26**) was the weakest stabilizer (FA EC_50_ ≥ 150 μM), whereas the longer linkers significantly improved the potency. The 1C-linker (**27**) showed similar binding to 14-3-3σ/ERα and 14-3-3σ/C-RAF by both MS and FA (Tables S2 and S3, FA EC_50_ = 19 ± 1 μM for ERα, EC_50_ = 16 ± 4 μM for C-RAF). Longer linkers were less consistent. The 2C-linker (**28**) favored 14-3-3σ/C-RAF by MS (Figure S2 and Table S2) but showed similar EC_50_ values for 14-3-3σ/ERα and 14-3-3σ/C-RAF by FA (Table S3). The 3C-linker (**29**) bound similarly to 14-3-3σ/ERα and 14-3-3σ/C-RAF by MS at 100 nM compound, although a lower maximum was reached in the case of C-RAF in the dose–responses (Figure S2 and Table S2). We hypothesized that the lower plateau was indicative of steric hindrance. The C-RAF phosphopeptide sequence extended well beyond the binding site of the small molecules and was also highly dynamic, in contrast to the ERα peptide. It was thus reasonable to conclude that the compound was not fully bound. In the FA assay, compound (**29**) showed a lower EC_50_ for ERα (9 ± 0.4 μM) compared to C-RAF (142 ± 40 μM, Table S3). Encouragingly, all four chloroacetamide analogues showed low binding to 14-3-3σ in the absence of the peptide in the MS assay (Figure 2B), indicating that the compounds acted as molecular glues that stabilized the complex of 14-3-3σ with each of the phosphopeptides.

Crystal structures were solved for three chloroacetamide analogues (linkers *n* = 1, 2, 3) in complex with 14-3-3σ/ERα and compared with the binding mode of the original tethering hit **fragment 1** (Figure S3A). It is noteworthy that although the tethering hit was crystallized using a soaking method, for the electrophilic analogues, cocrystallization was more successful in obtaining high-resolution structures (Figure S3B), which confirmed covalent binding to Cys38. Cocrystallization was done by overnight incubation with the compound, which allowed enough time for the covalent bond to form. Compound **27** (1C-linker) bound in a similar mode as the tethering hit, with the *gem*-dimethyl substituent positioned 4.4 Å from the methyl group of Val595 of ERα (Figures 2D and S3A). Differences were observed, as expected, in the orientation of the amide bond close to the warhead, since the amide bonds in the two analogues were reversed (Figure S3A). Analogues **28** (2C-linker) and **29** (3C-linker) showed similarities with each other regarding the orientation of the linker, but changes were observed in the positioning of the *gem*-dimethyl group. For **28**, the distance from Val595 was 5.5 Å, whereas the *gem*-dimethyl of **29** was 4.0 Å from Val595. An additional difference for compound **29** was the orientation of the ether group, which was directed “outward” of the 14-3-3 binding groove, facing away from 14-3-3 (Figure 2D and detailed overlays in S3A).

Additional to the linker length optimization, we focused on the potential effect of the warhead reactivity in stabilization. The more reactive α-chloroketone analogue (**37**) was synthesized and tested (Figure S4). In the MS assay, the compound showed remarkably faster kinetics; however, this high reactivity correlated with increased binding to 14-3-3 in the absence of peptides. In the FA assay, a higher EC_50_ value was observed for α-chloroketone (**37**) compared to chloroacetamide analogue **27** (EC_50_ = 44 ± 2 μM vs 19 ± 1 μM). Overall, the high chemical reactivity and high binding to 14-3-3 alone made this compound unsuitable for further studies.

For further SAR optimization, the 1C-chloroacetamide warhead (**27**) was selected since it showed consistency in the MS and FA assays (Figure 2B,C), a similar conformation as the disulfide hit (Figure S3), and a clear cooperative effect for PPI stabilization. The short linker also allowed the initial conformational restriction of the warhead, which was helpful for early rounds of compound optimization.

### Chemical Modifications in Close Proximity to the Peptides Tuned the Selectivity and Increased the Cooperativity for the 14-3-3σ/ERα Complex

**Figure 3:**
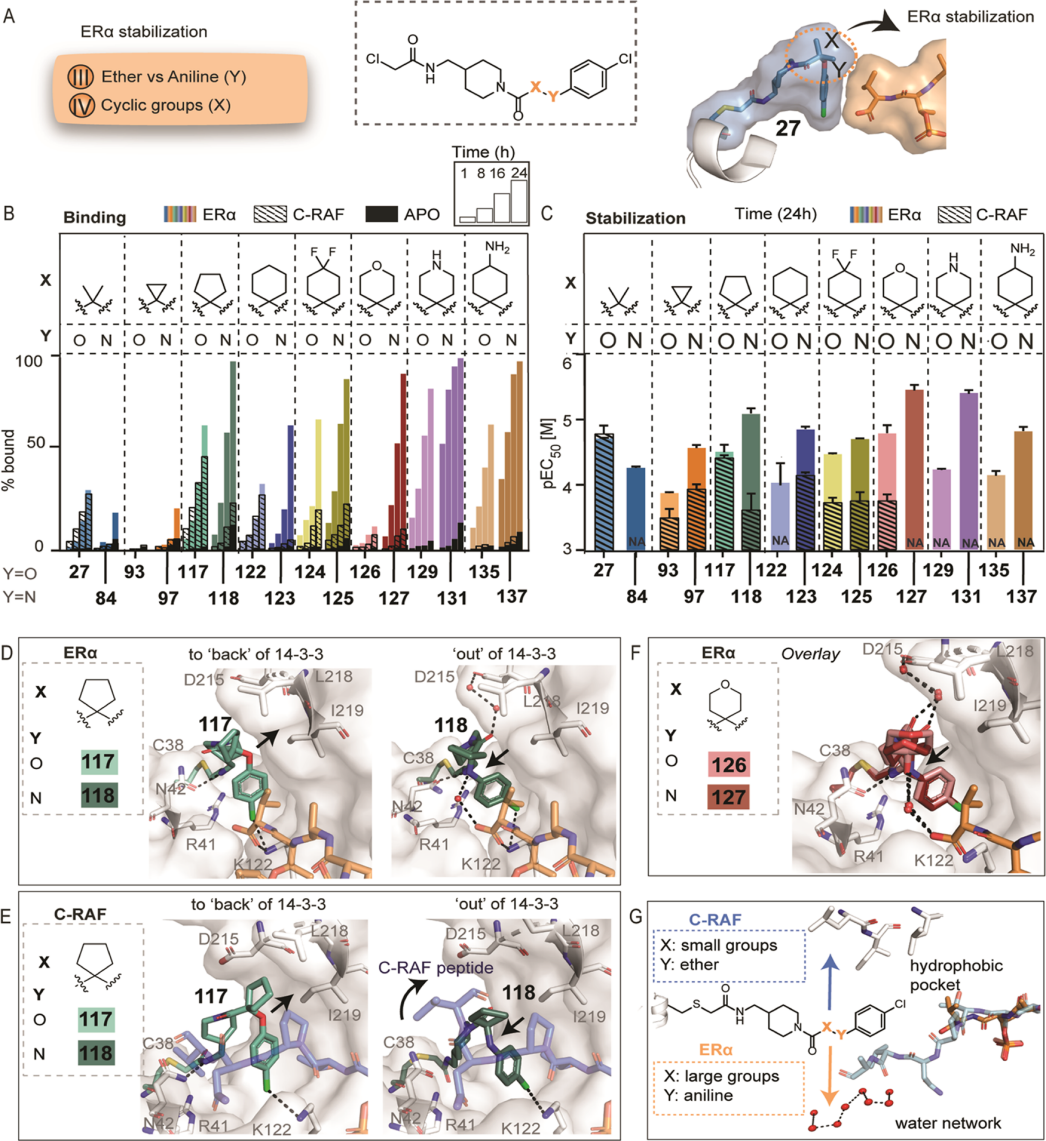
Improving selectivity and cooperativity for the 14-3-3σ/ERα complex. (A) Chemical modifications III and IV replacing the *gem*-dimethyl group with cyclic, aliphatic groups and comparing ethers and anilines. These changes aimed to increase the stabilization by targeting Val595 of ERα via hydrophobic, van der Waals interactions and interactions with hydrophobic residues on 14-3-3σ. (B) Bar graphs of mass spectrometry data at 100 nM [compound]. For each compound, time course experiments were performed with measurements at 1, 8, 16 and 24 h. ERα data are shown with different colors, C-RAF data with dashed lines and apo data in black. Full curves are depicted in Figures S13–S15. (C) Bar graphs of FA compound titration pEC_50_ values after overnight incubation. ERα data are shown with different colors and C-RAF data with dashed lines. Inactive compounds for C-RAF are described as nonapplicable (NA), whereas for weak compounds with EC_50_ values in the range of 140–160 μM and reasonable Hill slopes, bar graphs are shown. Full curves are depicted in Figures S16 and S17. (D) Crystal structures of the cyclopentyl analogues **117** (ether) and **118** (aniline) with 14-3-3σ/ERα. (E) Crystal structures of the cyclopentyl analogues **117** (ether, light green sticks) and **118** (aniline, dark green sticks) with 14-3-3σ (white surface) and C-RAF (blue sticks). (F) Crystal structures of the tetrahydropyran analogues **126** (ether, light red sticks) and **127** (aniline, dark red sticks) with 14-3-3σ (white surface) and ERα (orange sticks). Interacting water molecules are shown as red spheres. (G) Schematic representation of the preferred compound conformation with the two phosphopeptides. For 14-3-3σ/ERα stabilizers, larger groups in X position are preferred, with an aniline group facing in the front and participating in the water network. For 14-3-3σ/C-RAF, smaller groups, such as the cyclopentyl group in X position are preferred, with an ether group facing in the back, toward 14-3-3.

Starting from the scaffold of **27**, we aimed to increase the cooperativity and tune the selectivity toward ERα by applying two modifications: (III) introduction of anilines instead of ethers at position Y and (IV) replacement of the *gem*-dimethyl group with cyclic aliphatic rings at position X (Figure 3A). To address the aspects of kinetics early in the screening, we measured a dose–response MS assay every 8 h in the presence of 100 nM 14-3-3σ without peptide (Figure 3B, apo, black bars), in the presence of the ERα peptide (colored bars), or in the presence of the C-RAF peptide (dashed lines). Figure 3C shows the corresponding pEC_50_ values derived from FA dose–response curves in the presence of the ERα peptide (colored bars), or the C-RAF peptide (dashed lines).

Interestingly, selectivity for ERα was introduced by replacing the ether of **27** with an aniline, as observed for compound **84** (EC_50_ = 55 ± 2 μM for ERα and EC_50_ ≥ 150 μM for C-RAF Table S3). An alluring structural observation was that the aniline group of **84** was directed “outward” of the binding groove (similar to the ether functionality of **29**, with a linker length of *n* = 3, as observed previously), whereas the ether of **27** was positioned in the “back” (Figure S5A). Because of this conformational switch, the aniline of **84** could now participate in a water-mediated hydrogen bond with the terminal carboxyl group of ERα, and its carbonyl could interact via water-mediated hydrogen bonds with Asp215 of 14-3-3 (Figure S5A). An overlay with the C-RAF peptide shows that this “outward” conformation of **84** potentially sterically clashes with the C-RAF peptide (Figure S5B), which would explain its increase in selectivity for ERα. For both compounds (**27, 84**), despite the conformational switch, a halogen bond was formed between *p*-Cl and Lys122 at 3.5 Å (Figure S5A), similar to the halogen bond observed in the original fragment **1**. Of note, without the *p*-Cl group, the analogue was inactive (compound **85**), confirming its importance (Figure S6). Taking those observations into account, we considered the halogen bond a favorable structural feature and maintained it during SAR optimization. Halogen bonds, especially with chloro groups, are highly beneficial in drug discovery and have even been described as “magic chloros” in recent reviews.^67^ Additionally, the 3.5 Å distance indicated that bigger halogens would cause steric hindrance and smaller substituents would be unable to interact optimally with Lys122. In a related series, analogues where the *p*-Cl group was replaced with other functional groups were less effective in stabilizing the 14-3-3σ/ERα interaction.^68^

We hypothesized that we could increase stabilization by introducing alicyclic rings at the *gem*-dimethyl position (X) because of the hydrophobic +1 Val of ERα and the hydrophobic pocket of 14-3-3 at the peptide interaction interface (Figure S7). Notably, initial attempts for improving the cooperativity toward the 14-3-3σ/ERα complex were unsuccessful with the introduction of various larger hydrophobic substituents at position X (from 2-methyl-cyclopropyl to benzyl (Figure S8)), resulting in compounds **45**, **52**, **60**, **67**, and **76**–**83** (Table S1). Also, the incorporation of small cyclopropyl analogues with ether or aniline linkages did not improve stabilization (**93** and **97**) and the compounds were less selective than the *gem*-dimethyl analogues (Figure 3B,C). Excitingly, we noticed an increase in stabilization by increasing the ring size to cyclopentyl groups (**117** and **118**) (Figure 3B). Compound **117** (cyclopentyl, ether) showed improved stabilization with both peptides (FA EC_50_ = 15 ± 2 μM for ERα, 32 ± 8 μM for C-RAF), whereas **118** (cyclopentyl, aniline) showed greater than 30-fold selectivity for ERα (FA EC_50_ = 5 ± 0.4 μM, EC_50_ ≥ 150 μM for C-RAF) (Figure 3B,C and Tables S2 and S3). The selectivity for ERα can be explained by a similar conformational switch as was observed for the *gem*-dimethyl analogues, allowing for water-mediated hydrogen bonds between the aniline of **118** and the terminal carboxyl group of ERα (Figure 3D). Crystal structures of these analogues in the presence of the C-RAF peptide showed a similar conformational switch (Figure 3E), indeed indicating a steric clash of the aniline conformation (**118** EC_50_ for C-RAF ≥ 150 μM) with C-RAF, resulting in a peptide displacement. This explains why the ether analogue **117** was preferred for stabilizing C-RAF (EC_50_ 32 ± 8 μM) because its conformation close to 14-3-3 allowed for the peptide to wrap around the compound. Additionally, the larger cyclopentyl groups of both analogues (**117** and **118**) participated in hydrophobic interactions with Leu218 and Ile219 of 14-3-3, both in the presence of the ERα or C-RAF peptide, explaining their overall increase in stabilization (Figure 3D,E). Remarkably, removing the *p*-Cl group of the ether analogue (resulting in compound **119**) revealed an unexpected stabilization for both ERα and C-RAF (EC_50_ 24 ± 2 and 18 ± 0.3 μM, respectively) (Figure S9A,B). A crystal structure, in complex with 14-3-3 and ERα, showed the presence of a coordinated water molecule, interacting with the ether of **119** and Asn42 and Ser45 of 14-3-3 (Figure S9C). Although unusual, the location of this particular bond seemed to create a type of a macrocyclic intermolecular interaction between the compound and residues of 14-3-3, while lacking interactions with the peptide. This might be the underlying cause for the observed activity for both targets. While intriguing, this analogue was not followed up due to its nonselectivity.

Interestingly, the replacement of the cyclopentyl ring with a cyclohexyl led to weaker analogues for both peptides, but the introduction of heteroatoms (F, O, N) on the cyclohexyl ring significantly improved the activity for the 14-3-3σ/ERα complex (Figure 3A,B). Specifically, the *gem*-fluoro analogues were selective for stabilization of ERα over C-RAF, with the aniline analogue (**125**) being slightly more potent than the ether (**124**) (FA EC_50_ = 12 ± 2 and 18 ± 0.4 μM for ERα, respectively; EC_50_ ≥ 150 μM for C-RAF, Table S3). The tetrahydropyran analogue (**126**) with an ether linkage was more selective for ERα in the FA assay (FA EC_50_ = 9 ± 2 μM for ERα, EC_50_ ≥ 150 μM for C-RAF), whereas replacing the ether with an aniline (**127**) significantly improved the EC_50_ and maintained the selectivity for ERα in both the MS and FA assays (FA EC_50_ = 2 ± 0.3 μM, EC_50_ ≥ 150 μM for C-RAF). For the first time, both the ether and aniline groups of **126** and **127** were directed “outward” of 14-3-3, allowing the interaction via a water-mediated hydrogen bond with C-terminal carbonyl of ERα (Figures 3F and S10). Replacing tetrahydropyran with a piperidine or an amino-cyclohexyl group significantly improved the binding in the presence of ERα in the MS experiment and faster binding was observed, e.g., at the 1 h time point (Figure 3B and Table S2). In the FA assay, the aniline analogues **131** and **137** showed low EC_50_ values (FA EC_50_ = 2 ± 0.3 μM and 8 ± 1 μM, respectively, Table S3) and the two compounds were selective for ERα in both the MS and FA assays; no stabilization was observed for C-RAF (Figure 3B,C and Tables S2 and S3). A plausible explanation for the lack of stabilization for C-RAF is steric hindrance, as the size of the substituents close to the peptide increased (Figure S11). Additionally, close analogues of **131** and **137** without the *p*-Cl substituent were significantly less potent in both MS and FA assays, in agreement with previous observations (Figures S12 and Tables S2 and S3).

Taken together, in general for ERα, large cyclic groups in position X were well-tolerated, and anilines at position Y were preferably oriented out of the pocket, allowing for water-mediated hydrogen bonding with the terminal carboxyl of ERα. In contrast, for C-RAF, this conformation led to steric hindrance, and small groups in position X in combination with ether linkers were preferred to position the compound close to 14-3-3, allowing for C-RAF to wrap around the compound (Figure 3G). It is noteworthy that chemical modifications up to this point were correlated with cooperative binding, since the compounds showed very little binding to 14-3-3 in the absence of peptides (observed in the MS assay).

### Retesting the Effect of Linker Length Demonstrated that the Flexibility of the Warhead Affects the Cooperativity

**Figure 4:**
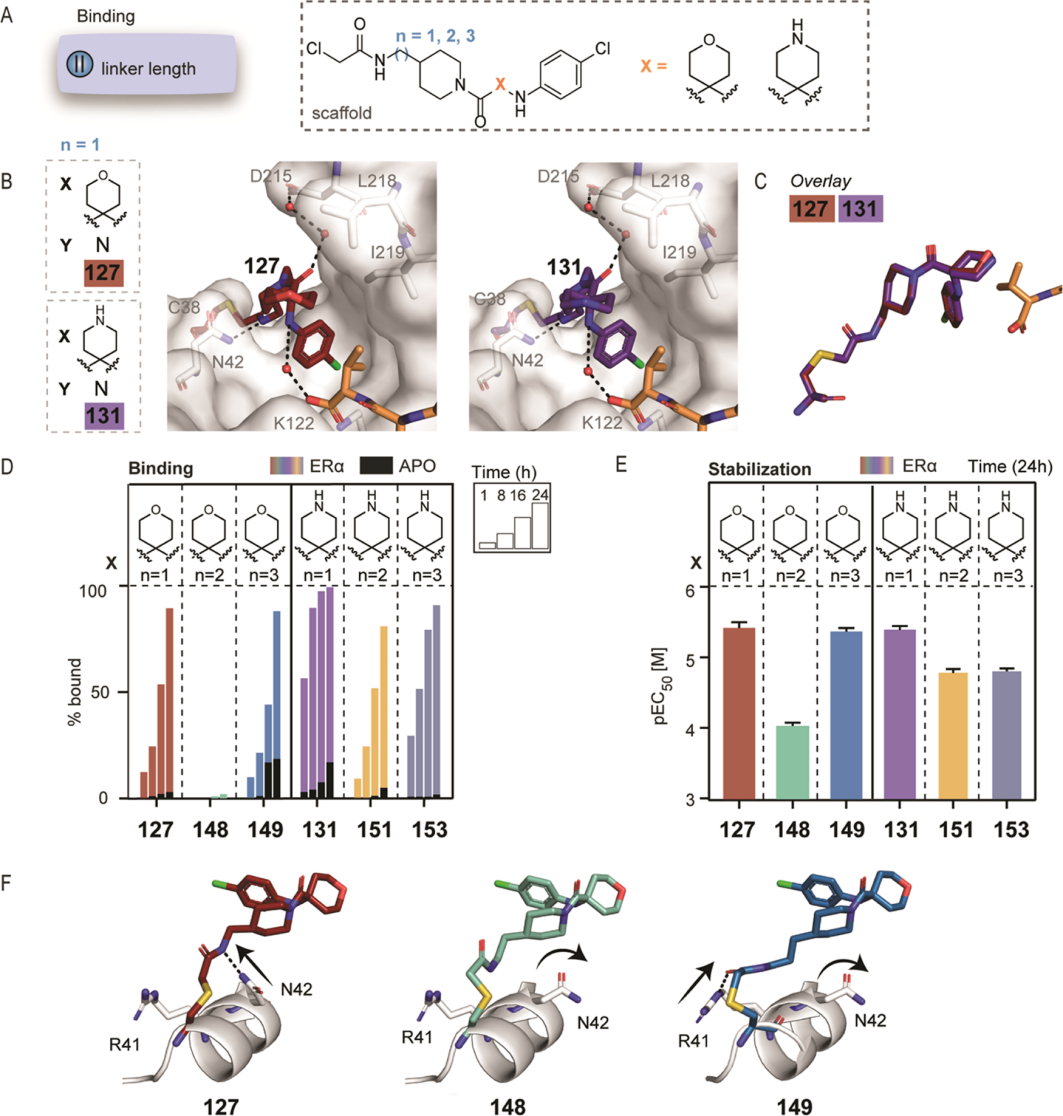
Retesting effect of linker length. (A) Modifications of linker length II of the warhead for both tetrahydropyran and piperidine analogues. (B) Crystal structures of aniline analogues **127** (red) and **131** (purple) in complex with 14-3-3σ (white) and ERα (orange). (C) Crystallographic overlay of **127** (red) and **131** (purple). (D) MS bar graphs (% bound to 14-3-3) of tetrahydropyran and piperidine analogues with linker length *n* = 1–3 at 100 nM [compound]. For each compound, measurements were performed at 1, 8, 16, and 24 h (ERα in colors, apo in black). Full curves are depicted in Figure S21. (E) Bar graphs of FA compound titrations pEC_50_ values after overnight incubation with ERα and 14-3-3σ. Full curves are depicted in Figure S21. (F) Crystal structures of compounds with increasing linker length (*n* = 1–3 of **127**, **148**, and **149**) in complex with 14-3-3σ/ERα, showing hydrogen bonds as black dashes with amino acids Arg41 or Asn42 of 14-3-3.

We next focused on the most promising and selective derivatives **127** and **131** and reanalyzed the effect of their linker length (*n*) for stabilizing the 14-3-3σ/ERα complex (Figure 4A). Both **127** and **131** (both with EC_50_ of 2 ± 0.3 μM) (*n* = 1) showed an identical binding mode and interacted via water-mediated hydrogen bonds with Asp215 of 14-3-3σ and the terminal carboxyl group of ERα (Figure 4B,C). Additionally, Asn42 of 14-3-3σ interacted directly with the nitrogen of the amide linker of both compounds. The halogen bond between the *p*-Cl group and Lys122 was also observed in both analogues.

To evaluate compound-mediated stabilization of the 14-3-3σ/ERα complex directly, FA protein titrations were performed at a saturating concentration of compound. FA protein titrations allowed the quantification of the cooperative effect by comparing the apparent dissociation constants (*appK*_D_) of the binary and ternary complexes. 14-3-3σ was titrated into 10 nM FAM-labeled ERα peptide in the presence of DMSO or 100 μM of the compounds (Table S4). The apparent dissociation constant (*appK*_D_) was 1493 nM in the absence of compounds and decreased to 77 nM in the presence of **127** and to 49 nM in the presence of **131**. Thus, compounds **127** and **131** stabilized the 14-3-3σ/ERα complex by 19- and 30-fold, respectively, compared to the DMSO control (Figure S18).

Four chloroacetamide analogues with longer linkers (2 or 3 carbons) were synthesized and compared to analogues **127** and **131** (Figure 4D,E). Different trends were observed; for tetrahydropyrans, 1C- (**127**) and 3C-linkers (**149**) were well-tolerated and resulted in comparable stabilization (**127**: EC_50_ 2 ± 0.3 μM, **149**: EC_50_ 4 ± 0.4 μM), while the 2C-linker was significantly weaker (**148**: EC_50_ 92 ± 8 μM). In FA protein titrations, 100 μM of **149** (3C-linker) decreased the *appK*_D_ of 14-3-3σ/ERα to 27 nM, thus showing a 55-fold stabilization and a strong cooperative effect (Figure S18). For the piperidine analogues, in both MS and FA assays, only the 1C-linker (**131** EC_50_ 2 ± 0.3 μM) showed comparable stabilization with the tetrahydropyran analogues, whereas 2C- (**151**) and 3C-linkers (**153**) were tolerated but weaker (**151**: EC_50_ 16 ± 2 μM, **153**: EC_50_ 16 ± 1 μM). Comparison of the crystal structures of tetrahydropyran analogues (**127**, **148**, **149**) showed a similar binding mode to the tetrahydropyran moiety (Figure S19), with an altered conformation of the linkers.

We noticed an interplay between the amide bond of the linkers and the 14-3-3 residues Arg41 and Asn42 (Figure 4F). At the shortest linker length (*n* = 1, **127**), a hydrogen bond was formed between Asn42 of 14-3-3 and the nitrogen of the amide of **127**. This hydrogen bond was disrupted by lengthening the linker by 1 carbon (*n* = 2, **148**), forcing Asn42 to move away from the compound due to steric hindrance. Interestingly, lengthening the linker even further by another carbon (*n* = 3, **149**) resulted in a newly formed hydrogen bond with the carbonyl group of **149** and Arg41 of 14-3-3. These observations explained the loss in stabilization observed for linker length *n* = 2, which could be rescued by lengthening the linker to *n* = 3.

Two more warheads were synthesized for the tetrahydropyran analogues: vinylsulfonamide **155** and α-chloroketone **156** (Figure S20). Consistent with our previous observations, the vinylsulfonamide was inactive (FA EC_50_ ≥ 150 μM). The ketone, although appearing active in the FA assay, lacked selectivity in the MS assay in the presence of ERα and C-RAF peptides and featured significantly increased apo binding (Figure S20).

### Conformational Locking of the Warhead Reduced the Flexibility of the Compound at the Rim of the Interface

**Figure 5:**
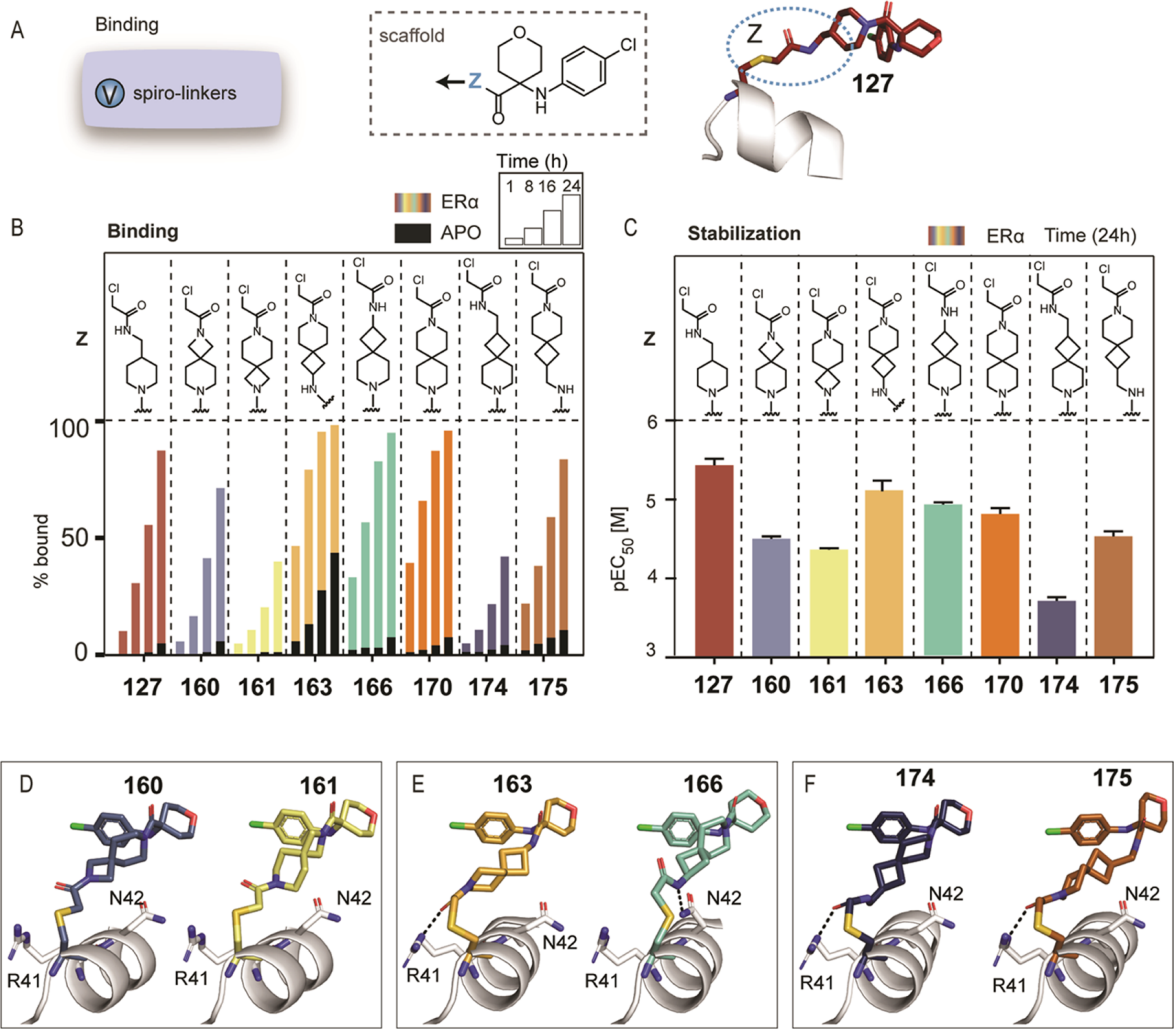
Conformationally locking the warhead. (A) Chemical modification V aimed to replace the long, flexible linkers with conformationally constrained spiro-linkers. Seven spiro-analogues were synthesized and tested, with varying linker lengths and reversed rings. (B) MS bar graphs indicate that although the spiro-linker is far from the protein–peptide interface, it can significantly affect binding and stabilization. For each compound, measurements were performed at 1, 8, 16, and 24 h (ERα in colors, apo in black). Full curves are depicted in Figure S22. (C) FA bar graphs of the spiro-analogues after overnight incubation with pEC_50_ values. Full curves are depicted in Figure S23. (D) Crystal structures of compounds **160** (light blue) and **161** (yellow) (pair of small spiro-analogues with reversed rings) in complex with 14-3-3σ/ERα. (E) Crystal structures of compounds **163** (orange) and **166** (green) (pair of spiro-analogues with one extra bond and reversed rings) in complex with 14-3-3σ/ERα. (F) Crystal structures of compounds **174** (dark blue) and **175** (brown) (pair of spiro-analogues with one extra −CH_2_– and reversed rings) in complex with 14-3-3σ/ERα.

The long flexible linkers of chloroacetamide analogues prompted us to focus on their rigidification, aiming to position the warhead for reaction but reduce the entropic penalty for binding. The warhead linker was rigidified by introducing spiro-cycles at the tetrahydropyran analogue **127**, thereby restricting the number of conformations in the vicinity of covalent bond formation (Figure 5A). While these modifications were far from the protein–peptide interface, they had a significant impact on their stabilization potential (Figure 5B,C). Crystallography studies indicated that the conformation of the spiro-rings, as well as their size, influenced how the warhead was oriented and whether it could form hydrogen bonds with the adjacent polar amino acids Arg41 and Asn42 (Figure S24).

Different sizes of spiro-rings were included. The first two compounds (**160** and **161**) had the smallest spiro-rings of the series and varied in the attachment of the same spiro building block. For those compounds, in both the MS and FA assays, less stabilization was observed, compared to the linear analogue **127** (FA EC_50_ = 2 ± 0.3 μM, 16 ± 1 μM for **160** and 22 ± 1 μM for **161**) (Figures 5B,C and S24A and Table S3). Both analogues adopted the same conformation where no hydrogen bonds were observed with Arg41 or Asn42 of 14-3-3 (Figure 5D). An overlay with analogue **149** (linear 3C-linker) indicated that the overall binding mode close to the protein/peptide interface was identical for compounds **149**, **160**, and **161** (Figure S24B). However, we contemplated that the spiro-analogues might have required a slightly longer linker to interact more favorably with Cys38.

Taking the crystallography-based information into account, one extra bond was added to the spiro warheads and compounds **163** and **166** were synthesized. In the MS assay, both compounds appeared highly potent and showed faster binding kinetics than **127** (Figure 5B and Table S2), which translated into low EC_50_ values in the FA compound titrations (EC_50_ = 4 ± 1 μM for **163** and 6 ± 0.3 μM for **166**) (Figure 5C and Table S3). For **163**, however, apo binding also increased over time (Figure 5B). One hypothesis for the increased apo binding was the formation of a direct hydrogen bond with Arg41 (2.8 Å between the carbonyl of the warhead and the Arg41) (Figure 5E). In contrast, for **166** the orientation of the warhead differed, the hydrogen bond was not formed, and apo binding was reduced (Figures 5E and S24C). The introduction of two fused piperidine rings (compound **170**) was well-tolerated in the MS assay, with relatively low apo binding and also a low EC_50_ in the FA assay (8 ± 1 μM) (Figure 5B,C and Tables S2 and S3). No crystal structure was solved for this analogue. All three analogues with similar lengths of spiro-rings (**163**, **166**, **170**) showed faster kinetics in the MS assays and potent stabilization effects in the FA assay, comparable to the linear analogue **127** (Figure 5B,C). In FA protein titrations, compounds **163**, **166**, and **170** showed *appK*_D_ of 77 nM (18-fold stabilization), 160 nM (13-fold stabilization), and 119 nM (12-fold stabilization), respectively (Figure S25 and Table S4).

To determine the optimum size for the spiro warheads, two larger analogues were synthesized by including an additional methylene group (compounds **174** and **175**). Both analogues appeared to be weaker (FA EC_50_ = 97 ± 9 μM and 15 ± 2 μM, respectively) than the structurally similar, but smaller, analogues **163** and **166** (Figure 5B,C and Tables S2 and S3). Significantly different orientations were observed in the crystal structures of **174** and **175**, especially for the latter analogue—an indication that the larger spiro warhead was less tolerated due to potential steric hindrance (Figures 5F and S24D). For these analogues, the formation of a hydrogen bond with Arg41 of 14-3-3 was not beneficial.

### Fine Tuning of Cooperativity with Synergistic Structural Modifications

**Figure 6:**
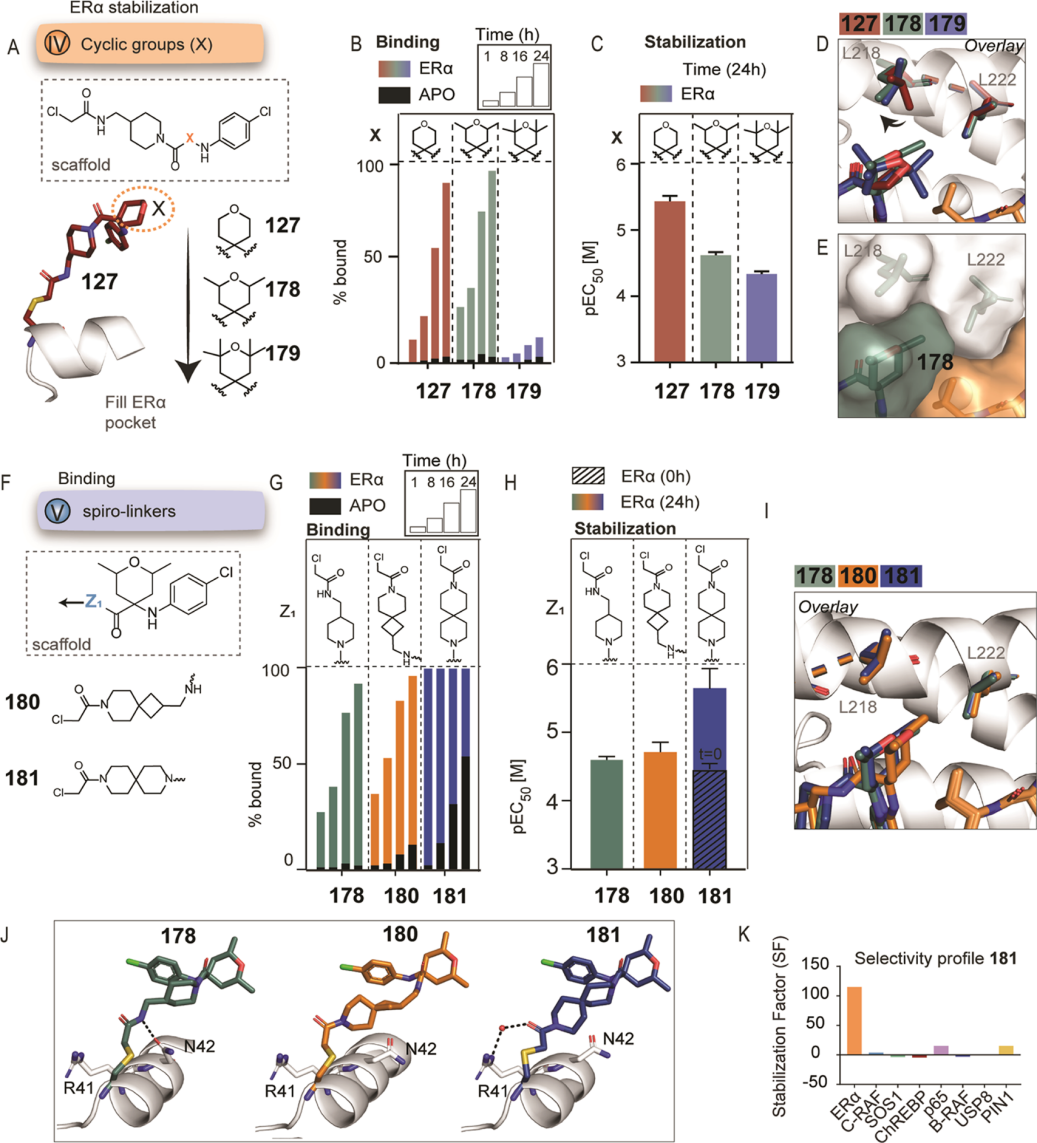
Combinations of 2,6-dimethyl tetrahydropyran with spiro-linkers. (A) Chemical modification IV, introducing methyl groups. (B) MS bar graphs (% bound to 14-3-3σ) for compounds **127**, **178**, and **179** at 100 nM [compound]. Each compound is measured at 1, 8, 16, and 24 h (ERα in colors, apo in black). Full curves are depicted in Figure S26A. (C) Bar graphs of FA compound titrations of the same analogues, pEC_50_ values after overnight measurement. Full curves are depicted in Figure S26B. (D) Crystal structures of compounds **127** (red), **178** (green), and **179** (blue) in complex with 14-3-3σ (white) and ERα (orange), with the surface representation of **178** in panel (E). (F) Combinations of 2,6-dimethyl tetrahydropyran with spiro-linkers. (G) MS bar graphs (% bound to 14-3-3σ) for compounds **178**, **180**, and **181**, at 100 nM [compound]. Each compound is measured at 1, 8, 16, and 24 h (ERα in colors, apo in black). Full curves are depicted in Figure S28A. (H) Bar graphs of FA compound titrations of the same analogues, EC_50_ values after overnight measurement. Full curves are depicted in Figure S28B. (I) Overlay of the crystal structures of compounds **178** (green), **180** (orange), and **181** (blue) in complex with 14-3-3 (white) and ERα (orange). (J) Complete chemical structures of the same compounds and the interplay with Arg41 and Asn42 of 14-3-3 (white sticks). (K) Selectivity profile of **181** based on FA protein titrations. Full graphs are depicted in Figure S31.

The structural modifications so far provided valuable insight both on the interactions in the interface and on the conformations in close proximity to the warhead. Four more analogues were synthesized (**178-181**): first, two methylated analogues of **127** (**178** and **179**) aimed to address both Val595 of ERα and the hydrophobic surface of 14-3-3 (Figure 6A).

Introducing two methyl groups at the tetrahydropyran moiety (compound **178**) led to faster binding in the MS experiment, especially at 1 and 8 h, in the presence of ERα (Figure 6B). However, this observation did not translate to improved stabilization in the FA assay (**178** EC_50_ = 12 ± 1 μM) (Figure 6C). The bulky analogue with four methyl groups (**179** EC_50_ = 23 ± 2 μM) appeared weaker in both assays, indicative of steric hindrance, which was confirmed by its crystal structure (Figure 6D). The addition of only two methyl groups (**178**) induced a movement of Leu218 of 14-3-3, forming a shallow hydrophobic pocket with Leu222, which was filled by one of the methyl groups of **178** (Figure 6E). FA protein titrations with 100 μM **178** showed an *appK*_D_ of 94 nM of the 14-3-3σ/ERα complex (15-fold stabilization) (Figure S27).

While crystallographic analysis hinted that the addition of two methyl groups led to more hydrophobic contacts with 14-3-3, this was not translated back into the FA assays. We contemplated that this could be due to the linker flexibility of **178**. This prompted us to combine the addition of two methyl groups with the conformationally restricted spiro-constructs.

Two more analogues were synthesized to explore potential synergistic effects of 2,6-dimethyl tetrahydropyran substitutions with spiro-linkers, resulting in analogues **180** and **181** (Figure 6F). In the MS assay, especially for compound **181**, 100% binding was reached at low compound concentrations in the 1 h time point (Figure 6G and Table S2) and this translated into faster stabilization in the FA assay (at 0 h: EC_50_ = 34 ± 3 μM, at 24 h: EC_50_ = 1 ± 0.8 μM) (Figure 6H and Table S3). The analogue with smaller spiro-rings (**180**) was weaker than **181** in the FA assay (at 24 h: EC_50_ = 10 ± 2 μM), with a similar activity as **178** (at 24 h: EC_50_ = 12 ± 1 μM). In protein titrations, compounds **180** and **181** showed an *app*K_D_ of 192 nM (7-fold stabilization) and 18 nM (116-fold stabilization), respectively (Figure S29 and Table S4). Crystal structures showed that one of the methyl groups of **180** and **181** adopted a similar binding mode as **178**, interacting with Leu222 and inducing the formation of the shallow hydrophobic pocket (Figure 6I). The two fused piperidine rings of **181** aligned with the linear linker of **178** and simultaneously filled the available space toward 14-3-3 (Figure S30). Overall, compounds **180** and **181** shared a common binding mode, similar interactions, and were able to participate in the water network, thus forming indirect polar interactions both with 14-3-3 and ERα (Figure 6J).

Despite the similarities in the binding mode of compounds **180** and **181**, remarkable differences were observed both in the MS and FA assays. Compound **181** maintained key interactions both with 14-3-3 and ERα and high shape complementarity with the binding pocket. Together, this translated to remarkable cooperativity in the activity assays. By adopting the optimal conformation, the compound could avoid paying the energetic penalty upon binding, a feature that seemed to be missing for compound **180** or **178**, despite the observed similarities in the crystal structures. Furthermore, restricting the possible compound conformations may have been crucial for improved cooperativity by facilitating the transition from disorder-to-order upon binding to 14-3-3 and formation of the ternary complex.

### Compound **181** Showed High Selectivity for the 14-3-3σ/ERα PPI Complex

As a hub protein, 14-3-3 interacts with numerous clients with a wide range of affinities. We hypothesized that selective stabilization with small molecules could be achieved by aiming for unique interactions at the 14-3-3/client interface and by conformationally locking the ternary complex. To confirm, we performed selectivity studies with a panel of 14-3-3 clients. 14-3-3 protein titrations were performed at 100 μM **181** with 10 nM of ERα (pT594, +1 Val), C-RAF (pS259, +1 Thr), SOS1 (pS1161, +1 Ala), ChREBP (phosphorylation-independent 14-3-3 interactor), p65 (pS45, +1 Ile), B-RAF (pS365, +1 Ala), USP8 (pS718, +1 Ser), or Pin1 (pS72, +1 Trp) (Figure S31) to determine the cooperative effects on those complexes. In addition to the differences in the +1 residues, the peptides varied in their shape, binding mode, and occupancy of the amphipathic groove. Compound **181** showed remarkable selectivity for 14-3-3σ/ERα (*appK*_D_ of 18 nM, 116-fold stabilization at 100 μM compound), whereas for the other clients, fold-stabilization varied from 0- to 15-fold (Figure 6K). An overlay with the other peptides indicated the lack of favorable interactions and shape complementarity (Figure S31A). The same experiment was performed for the natural product, FC-A. Notably, compound **181** had a similar selectivity profile as FC-A (Figure S31B).

### Compound **181** was as Potent as the Natural Product Fusicoccin-A

**Figure 7:**
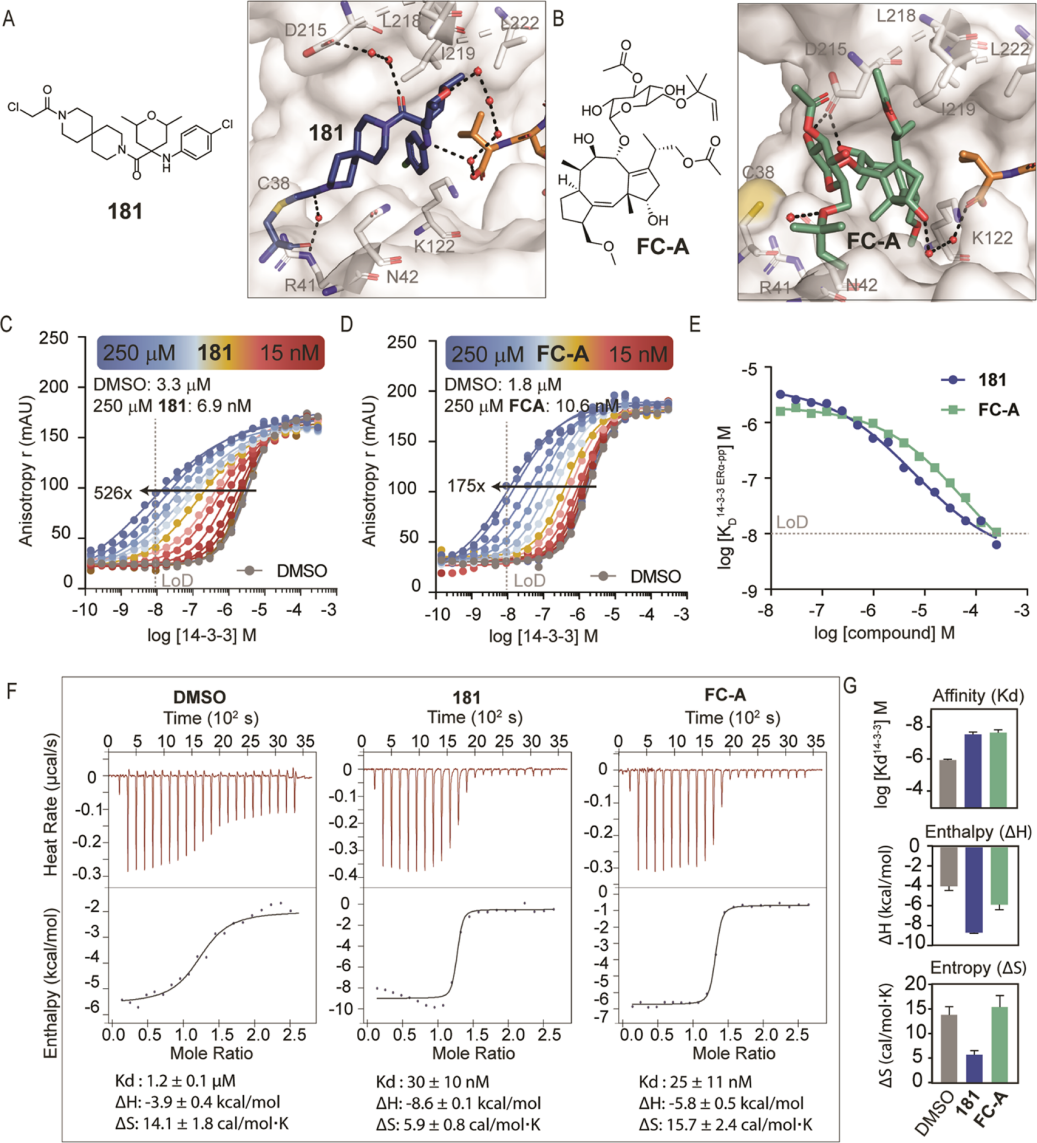
Comparison between the optimized covalent stabilizer **181** and the natural product fusicoccin-A. (A) Chemical structure of **181** and crystal structure of **181** (blue sticks) in complex with 14-3-3σ (white) and ERα (orange sticks). Water network is depicted as black dashes and red spheres. (B) Chemical structure of fusicoccin-A (**FC-A**) and crystal structure of **FC-A** (green sticks) with 14-3-3σ (white) and ERα (orange sticks) (PDB ID: 4JDD). (C) Titration of 14-3-3σ to FAM-labeled ERα (10 nM) against decreasing concentrations of **181** (between 0 and 250 μM). (D) Titration of 14-3-3σ to FAM-labeled ERα (10 nM) against decreasing concentrations of **FC-A** (between 0 and 250 μM). (E) Apparent K_D_ value of 14-3-3σ/ERα interactions (*y*-axis) in the presence of a range of concentrations (0–250 μM) of **181** (blue) or **FC-A** (green) (*x*-axis). (F) ITC experiments of ERα peptide titrations (300 μM) to 14-3-3σ (30 μM) in the presence of DMSO or 500 μM of **181** or **FC-A**. (G) Biophysical parameters derived from ITC experiments, comparing enthalpic and entropic differences between **181** and **FC-A** (measured in two independent experiments, Figure S32).

It is instructive to further compare the covalent stabilizer **181** to the natural product Fusicoccin-A (**FC-A**) (Figure 7). Structurally, **181** formed polar interactions both with 14-3-3 and ERα via the water network and interacted with Lys122 via a halogen bond (Figure 7A), whereas **FC-A** formed hydrogen bonds with Asp215 and Lys122 directly (Figure 7B). Since ternary complex formation in solution is dependent on relative concentrations of binding partners, we performed FA 2D titrations of **181** and **FC-A**. 14-3-3σ was titrated to FAM-labeled ERα-peptide (10 nM) in the presence of decreasing concentrations (0–250 μM) of either **181** (Figure 7C) or **FC-A** (Figure 7D). Plotting the change in *appK*_D_ value of the 14-3-3σ/ERα complex against a range of stabilizer concentrations (Figure 7E) showed that both compounds elicited a comparable stabilization profile.

ITC experiments were performed to study and compare the thermodynamics of the interaction between the ERα-peptide and 14-3-3σ in the presence of either 500 μM **181** or **FC-A** (Figure 7F). In the reference experiment, a full binding curve was obtained by titrating ERα peptide (300 μM stock concentration) into 14-3-3σ (30 μM). The determined *K*_D_ value for 14-3-3σ/ERα of 1.2 ± 0.1 μM was in close agreement with the K_D_ value of 2.4 μM determined by FA (Supp Methods, page S2). The measured negative enthalpy (Δ*H* = −3.9 ± 0.4 kcal.mol^–1^) indicated favorable binding interactions driving the complex formation, coupled with an increased entropy (Δ*S* = 14.1 ± 1.8 cal/mol·K). Addition of the covalent stabilizer **181** (500 μM) or noncovalent **FC-A** (500 μM) to the 14-3-3 solution lowered the calculated *K*_D_ of the 14-3-3σ/ERα interaction (30 ± 10 and 25 ± 11 nM, respectively). Deconvolution of similar Δ*G* for **181** and **FC-A** ternary complexes showed a difference in enthalpic and entropic contributions. Addition of **181** led to an increased enthalpically driven process, while ERα binding to 14-3-3 in the presence of **FC-A** was mainly entropically driven (Figure 7G). The decreased entropy in the **181** stabilized complex, compared to the DMSO reference, could be associated with the presence of structured water molecules that were stabilized by **181** and therefore not displaced from the binding site (Figure 7A). In the **FC-A** complex, more water molecules were displaced from the binding pocket, resulting in an increased disorder of the system (Figure 7B).

## Conclusions

In summary, we describe the structure-guided optimization of a nonselective disulfide fragment toward first-in-class, potent and selective small-molecule stabilizers for the 14-3-3σ/ERα complex. The hub protein 14-3-3 is involved in complex PPI networks and regulates pathways often dysregulated in pathological conditions. The extensive interactome of 14-3-3 represents a considerable challenge in targeting specific 14-3-3/client complexes via a molecular glue; furthermore, there has been a dearth of suitable starting points for chemical optimization of PPI molecular glues, including for those involving 14-3-3. Here, we show that even a nonselective fragment hit with low affinity for 14-3-3σ/ERα compared to 14-3-3σ/C-RAF can be used as a starting point for targeted chemical optimization. Supported by crystallographic data and focusing on differences between the two peptides in the interface with 14-3-3, we were able to dissect and optimize the substituents necessary to tune the selectivity toward ERα. Additionally, conformational restrictions of the synthesized stabilizers, especially in the rim of the PPI interface, resulted in increased cooperativity. The resulting compound **181** showed potency comparable to the natural product FC-A, as well as similar selectivity across a representative panel of 14-3-3 clients. The use of **181** as a probe of 14-3-3σ/ ERα cell biology will be reported in due course.

Our primary design principle focused on increasing orthosteric interactions with the phosphopeptide. Indeed, in the crystal structures of our compounds with 14-3-3σ/ERα, the phosphopeptide and 14-3-3 maintained the same conformations as in the binary complex, whereas peptide plasticity was observed for C-RAF. In principle, allosteric stabilization of the protein/peptide complex could also be employed.

The overall strategy, combining medicinal chemistry, biophysical assays, and crystallography, can be applied to other significant 14-3-3 clients for the development of client-selective stabilizers. More broadly, this work represents a proof of concept for the rational structure-guided optimization and development of molecular glues. Despite the increasing interest in modulating PPIs with new modalities, such as PROTACs or molecular glues, the identification of the latter largely relies on serendipity. Here, we show that a PPI of interest can be targeted selectively with small molecules derived from fragment hits. The nontrivial task of optimizing fragments toward small molecules can be facilitated by covalent binding and a combination of biophysical assays that address binding, kinetics, and cooperativity early on, whereas crystallography elucidates the binding mode of the compounds. It is noteworthy for molecular glues optimization that even one-atom modifications, such as an ether versus an aniline substituent, can have a significant impact on potency and selectivity; crystallography is crucial for elucidating the reasons behind this subtle structure–activity relationship. Covalent small molecules are occasionally considered nonselective and hence potentially toxic; however, as shown in this work and in many other recent publications for diverse targets,^69−74^ the fine tuning between covalent binding and reactivity of the electrophilic warhead can be achieved. The rationale and observations for the optimization of molecular glues presented here are readily applicable to the rapidly expanding field of PPI stabilization, particularly for targeting scaffold proteins that bind to intrinsically disordered regions.

## Supporting Information Available

Experimental methods, supplementary figures and tables, synthetic procedures, compound characterization, NMR spectra, and crystallography data (PDF)
