# Supplementary material for: Structure-Based Optimization of Covalent, Small-Molecule Stabilizers of the 14-3-3σ/ERα Protein–Protein Interaction from Nonselective Fragments
Source: J Am Chem Soc. 2023 Sep 7;145(37):20328–43. doi: 10.1021/jacs.3c05161 (PMC10515640; doi:10.1021/jacs.3c05161)
Supplement: Supplementary file 1 — ja3c05161_si_001.pdf [file ja3c05161_si_001.pdf]

Structure-based optimization of covalent, small molecule stabilizers of the 14-3-3σ/ERα protein-protein interaction from nonselective fragments

Markella Konstantinidou,†‡ Emira J. Visser,∥‡ Edmee Vandenboorn,∥ Sheng Chen,† Priyadarshini Jaishankar,† Maurits Overmans,∥ Shubhankar Dutta,† R. Jeffrey Neitz,† Adam R. Renslo,† Christian Ottmann,∥ * Luc Brunsveld,∥* and Michelle R. Arkin†*

†Department of Pharmaceutical Chemistry and Small Molecule Discovery Center (SMDC), University of California, San Francisco 94143, United States, ∥ Laboratory of Chemical Biology, Department of Biomedical Engineering and Institute for Complex Molecular Systems (ICMS), Eindhoven University of Technology, 5600 MB Eindhoven, The Netherlands

Table of contents

1. **Supplementary Methods**
   - Protein expression and purification p2
   - Peptide sequences p2
   - Mass spectrometry (MS) dose response assays p3
   - Fluorescence anisotropy (FA) measurements p3
   - Isothermal titration calorimetry (ITC) p4
   - X-ray crystallography data collection and refinement p4
   - Docking p5
   - Software p5
2. **Supplementary Figures**
   - Figures S1 – S34 p6 – 31
3. **Supplementary Tables**
   - Table S1: Overview of molecular structures of compounds and assays p32 – 34
   - Table S2: Percentage bound of compound to 14-3-3 measured by MS p35
   - Table S3: EC_{50} values measured by FA p36
   - Table S4: Protein titrations by FA p37
   - Tables S5-S11: crystallography data collection parameters p37 – 44
4. **Synthetic Procedures** p45 – 86
5. **Representative NMR spectra** p87 – 125
6. **References** p126 – 127

1. SUPPLEMENTARY METHODS

PROTEIN EXPRESSSION AND PURIFICATION

The 14-3-3 σ isoform (full-length for mass spectrometry and fluorescence anisotropy assays, ΔC for crystallography) with an N-terminal His6 tag was expressed in Rosetta^{TM} 2(DE3)pLysS competent E. coli (Novagen) from a pPROEX HTb expression vector. After transformation following manufacturer's instructions, single colonies were picked to inoculate 30 mL precultures (LB), which were added to 1.5 L terrific broth (TB) medium after overnight growth at 37°C, 250 rpm. Expression was induced upon reaching OD_{600} 1.9−2.1 by adding 400 μM IPTG. After overnight expression at 30°C, 150 rpm, cells were harvested by centrifugation at 6,500 rpm, resuspended in lysis buffer (50 mM HEPES pH 7.5, 500 mM NaCl, 20 mM imidazole, 10% glycerol, 1 mM TCEP), and lysed by sonication. The His6-tagged protein was purified by Ni-affinity chromatography (Ni-NTA Agarose, Invitrogen) (Wash buffer 50 mM HEPES pH 7.5, 500 mM NaCl, 20 mM imidazole, 1 mM TCEP; Elution buffer 50 mM HEPES pH 7.5, 500 mM NaCl, 500 mM imidazole, 1 mM TCEP) and analyzed for purity by SDS-PAGE and Q-Tof LC/MS. The protein was buffer exchanged (Storage buffer 25 mM HEPES pH 7.5, 150 mM NaCl, 1 mM TCEP) and concentrated to ~16 mg/mL and aliquots flash-frozen for storage at −80°C. The ΔC variant was truncated at the C-terminus after T231 to enhance crystallization and after the first Ni-affinity chromatography column, the construct was treated with TEV protease to cleave off the His6 tag during dialysis (25 mM HEPES, pH 7.5, 200 mM NaCl, 5% glycerol, 10 mM $MgCl_2$, 250 μM TCEP) overnight at 4 °C. The flow-through of a second Ni-affinity column was subjected to a final purification step by size exclusion chromatography (Superdex 75 pg 16/60 size exclusion column (GE Life Science) (SEC buffer: 25 mM HEPES pH 7.5, 100 mM NaCl, 10 mM $MgCl_2$, 250 μM TCEP). The protein was concentrated to ~60 mg/mL, analyzed for purity by SDS-PAGE and Q-Tof LC/MS and aliquots flash-frozen for storage at -80 °C.

PEPTIDE SEQUENCES

Peptides for mass spec dose responses were purchased from Elim Biopharmaceuticals, Inc. (Hayward, CA). Sequences were as follows: *Ac*-KYYITGEAEGFPA{pT}V-*COOH* (ERα-pp), *Ac*-RQRST{pS}TPNVH-*CONH₂* (CRAF pS259-pp). Peptides for X-ray crystallography and fluorescein-labeled peptides were purchased from GenScript Biotech Corp. Sequences were: *Ac*- or *5-FAM*-AEGFPA{pT}V-*COOH* (8mer ERα-pp) and QRST{pS}TPNVH-*CONH₂* (CRAF pS259-pp). K_d values of the fluorescently labeled peptides for 14-3-3σ are 2 μM for ERα and 10 μM for C-RAF (see titration curves below).

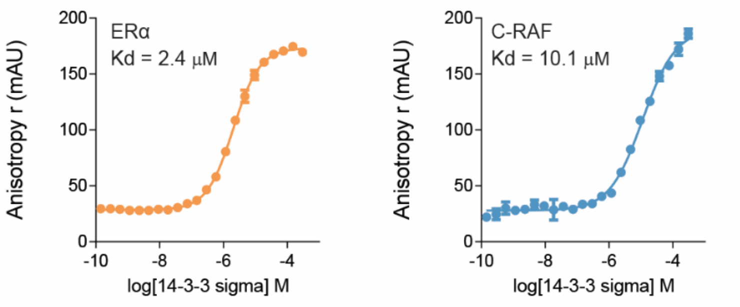

Titration of 14-3-3σ to 10 nM of FAM-labeled ERα (left) or 10 nM FAM-labeled C-RAF (right) with a determined Kd value of 2.4 μM for ERα and 10.1 μM for C-RAF.

LC-MS DOSE RESPONSE ASSAYS

Mass spectrometry dose response assays were performed on a Waters Acquity UPLC/ Xevo G2-XS Q-Tof mass spectrometer. A Waters UPLC Protein BEH-C4 Column (300 Å, 1.7 µm, 2.1 mm x 50 mm) was used to desalt the samples prior to application on the mass spectrometer. For 19-point MS dose responses, 50 mM compound stocks in DMSO were serially diluted in 3-fold increment in a master plate, then 1000 nl of the compounds were transferred in the assay plates. Master mixes containing 100 nM full-length wild type 14-3-3σ in the absence or presence of either 2 µM ERα or 18 µM C-RAF were then dispensed into 384 well plates (Greiner Bio-One, catalog number 784201). Assay buffer was TRIS (10 mM, pH 8.0) and final volume per well was 50 µl, with final top concentration of compounds dose response series at 1 mM. The reaction mixtures were incubated for 1h at rt before subjected to MS. Four measurements (1h, 8h, 16h, 24h) were performed for time-course experiments. The injection volume for each sample was 6 µl. 24 µl of sample were needed for the time-course experiments, so the total volume in the assay plate was adjusted to 50 µl, to account for the dead volume in the injections. Data collection and automated processing followed a custom workflow, as previously described.[1] z Plots were created using GraphPad Prism with the log(agonist) vs. response (variable slope, four parameters) fitting model.

FLUORESCENCE ANISOTROPY MEASUREMENTS

Fluorescein-labeled peptides (5-FAM), 14-3-3σ FL protein, and the compounds (50 mM stock solution in DMSO) were diluted in buffer (10 mM HEPES, pH 7.5, 150 mM NaCl, 0.1% Tween20, 1 mg/mL Bovine Serum Albumin (BSA; Sigma-Aldrich). Final DMSO in the assay was always 1%. Dilution series of 14-3-3 proteins or compounds were made in black, round-bottom 384-microwell plates (Corning) in a final sample volume of 10 µL in triplicates.

Compound titrations were made by titrating the compound in a 2-fold dilution series (starting at 500 or 1000 µM) to a mix of fluorescein-labeled peptide (10 nM) and 14-3-3σ (concentration at approximately the EC_{20} to EC_{30} value of the protein-peptide complex; 1 µM for ERα and 5 µM for C-RAF). Using the ~EC_{25} value makes the assay sensitive for measuring an increase in PPI formation. Fluorescence anisotropy measurements were performed directly and after overnight incubation at room temperature. The high protein concentration used in this assay limits the sensitivity for potent compounds (EC_{50} < 1µM).

Protein titrations were made by titrating 14-3-3σ in a 2-fold dilution series (starting at 300 µM) to a mix of fluorescein-labeled peptide (10 nM) and DMSO or compound (100 µM). Fluorescence anisotropy measurements were performed after overnight incubation at room temperature. Protein titrations were more sensitive than compound titrations for highly potent compounds and the $app\mathrm{K}_{D}$ values obtained were not limited by the protein concentration used.

Protein 2D titrations were made by titrating 14-3-3σ in a 2-fold dilution series (starting at 300 µM) to a mix of fluorescein-labeled peptide (10 nM) against varying fixed concentrations of compound (2-fold dilution, starting at 250 µM), or DMSO. Fluorescence anisotropy measurements were performed after overnight incubation at room temperature.

Fluorescence anisotropy values were measured using a Tecan Infinite F500 plate reader (filter set lex: 485 ± 20 nm, lem: 535 ± 25 nm; mirror: Dichroic 510; flashes:20; integration time: 50 ms; settle time: 0 ms; gain: 55; and Z-position: calculated from well). Wells containing only FAM-peptide were used to set as G-factor at 35 mP. Data reported are at endpoint. EC_{50} and apparent K_d values were obtained from fitting the data with a four-parameter logistic model (4PL) in GraphPad Prism 7 for Windows. Data was obtained and averaged based on either three (compound titrations) or two (protein titrations) independent experiments.

ISOTHERMAL TITRATION CALORIMETRY

The 14-3-3σ FL protein, ac-ERα peptide and compounds (50 mM stock solution in DMSO) were dissolved in ITC buffer (25 mM HEPES pH 7.4, 100 mM NaCl, 10 mM $MgCl_2$, 0.5 M tris-(2-carboxyethyl)phosphine (TCEP)) to a concentration of 30 µM of 14-3-3σ for the cell, and 300 µM of ERα-peptide for the syringe. DMSO or compound concentrations were matched in the cell and syringe till 500 µM of compound and 1% DMSO. Samples were degassed at 450 mmHg, 10 minutes prior to measurement. Measurements were performed on an Affinity ITC LV (TA instruments), with injection size set to 2 µL, stirring speed of 150 rpm and temperature at 25 °C. The data was processed and analyzed with NanoAnalyze v3.11. The baseline was manually inspected and corrected, after which a blank constant model was fitted to correct for the heat of injection. Subsequently, an independent model was fitted, which the NanoAnalyze software uses to report the thermodynamic binding properties reported in this paper.

X-RAY CRYSTALLOGRAPHY DATA COLLECTION AND REFINEMENT

The 14-3-3σΔC protein, *Ac*-ERα or *Ac*-C-RAF peptide and compounds (50 mM stock solution in DMSO) were dissolved in complexation buffer (25 mM HEPES pH 7.5, 2 mM $MgCl_2$ and 2 mM βME) and mixed in a 1:2:2 molar stoichiometry (protein : peptide : compound) at a final protein concentration of 12 mg/mL. The complex was set up for sitting-drop crystallization after overnight incubation at 4 °C, in a custom crystallization liquor (0.095 M HEPES (pH 7.1, 7.3, 7.5, 7.7), 0.19 M $CaCl_2$, 24-29 % PEG 400 and 5% (v/v) glycerol). Crystals grew within 10 – 14 days at 4 °C. Crystals were fished and flash-cooled in liquid nitrogen. X-ray diffraction (XRD) data were collected at either an in-house system Rigaku Micromax-003 (Rigaku, Europe, Kemsing Sevenoaks, UK) equipped with an Dectris Pilatus 200K detector, the Deutsche Elektronen-Synchrotron (DESY) Petra III beamline P11, Hamburg, Germany, the European Synchrotron Radiation Facility (ESRF Grenoble, France, beamline ID23-1, ID23-2, ID30A-1/MASSIF-1 or beamline ID30B/MAD) or at Diamond Light Source (DLS) (Oxfordshire, United Kingdom, beamline I03). Initial data processing was performed at DESY using XDS or at ESRF and Diamond using DIALS[2] after which pre-processed data was taken towards further scaling steps, molecular replacement and refinement.

Data was processed using the CCP4i2 suite (version 8.0.003)[3]. After indexing and integrating the data, scaling was done using AIMLESS.[4,5] The data was phased with MolRep[6], using 4JC3 and 3IQU as a template for ERα and C-RAF containing crystals, respectively. Presence of co-crystallized ligands was verified by visual inspection of the Fo-Fc and 2Fo-Fc electron density maps in COOT (version 0.9.6)[7]. If electron density corresponding to the co-crystallized ligand was present, its structure and restrains were generated using either AceDRG[8] or eLBOW.[9] For compounds **85** (PDB ID **8AI0**) and **123** (PDB ID **8ALV**), eLBOW was used to generate the structures and restraints, followed by model rebuilding and refinement using phenix.refine[10,11]from the Phenix software suite (version 1.19.2-4158) and Coot. For all the remaining compounds AceDRG was used to generate the structures and restraints, followed by model rebuilding and refinement using REFMAC5.[12] The PDB REDO server (pdb-redu.edu)[13] was used to complete the model building and refinement. The images were created using the PyMOL Molecular Graphics System (Schrödinger LLC, version 2.2.3). The structures were deposited in the protein data bank (PDB) with IDs: **8AV7** (27 non-covalent), **8AWG** (27 covalent), **8AXE** (28), **8ANF** (29), **8ARO** (84), **8AI0** (85), **8ARX** (93), **8ARZ** (97), **8AT9** (98), **8AXU** (117 - ERα) , **8ATR** (117 – C-RAF), **8AZE** (118 – ERα), **8ATS** (118 – C-RAF), **8AV8** (119), **8ALR** (121), **8ARY** (122), **8ALV** (123), **8AV3** (124), **8ALT** (125), **8AV4** (126), **8ALW** (127), **8AM7** (129), **8ATP** (131), **8AS1** (133), **8ARW** (135), **8ARR** (137), **8ARG** (139),

8AQZ (148), **8AOY** (149), **8AU2** (151), **8AR5** (160), **8ARQ** (161), **8AQC** (163), **8AQE** (166), **8AUS** (174), **8AUY** (175), **8ARX** (178), **8AR4** (179), **8AQ1** (180), **8APS** (181).

See SI tables S5-S11 for data collection and refinement statistics and Fig S33-S34 for representation of compound densities.

DOCKING

Computational design for SAR optimization and docking was performed with SeeSAR version 11.2.0; BioSolveIT GmbH, Sankt Augustin, Germany, 2022, www.biosolveit.de/SeeSAR

SOFTWARE VERSIONS

Prism (7.00)
Illustrator (22.1 (64-bit))
Biorender (64-bit)
Pymol (2.2.3)
CCP4i2 (8.0.003)
COOT (0.9.8.1)
Phenix (1.19.2-4158)
Nanoanalyze (for analyzing ITC graphs) (3.11)

2. SUPPLEMENTARY FIGURES

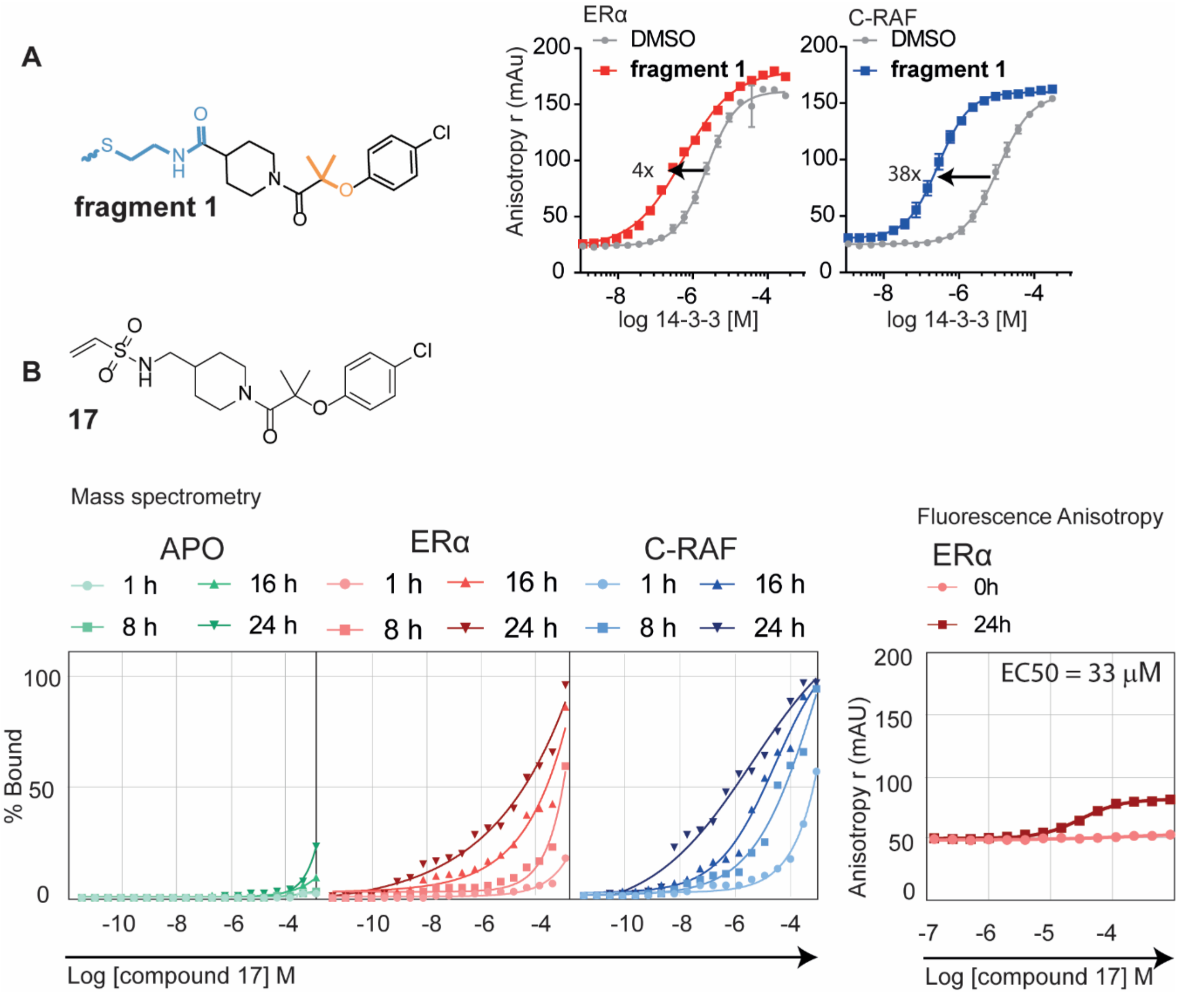

Figure S1: (A) FA protein titrations for disulfide hit **fragment 1** (100 μM) with 10 nM of ERα-peptide (left) or 10 nM of C-RAF peptide (right). (B) MS dose-response curves and FA dose-response curves for vinylsulfonamide **17** with 1C linker.

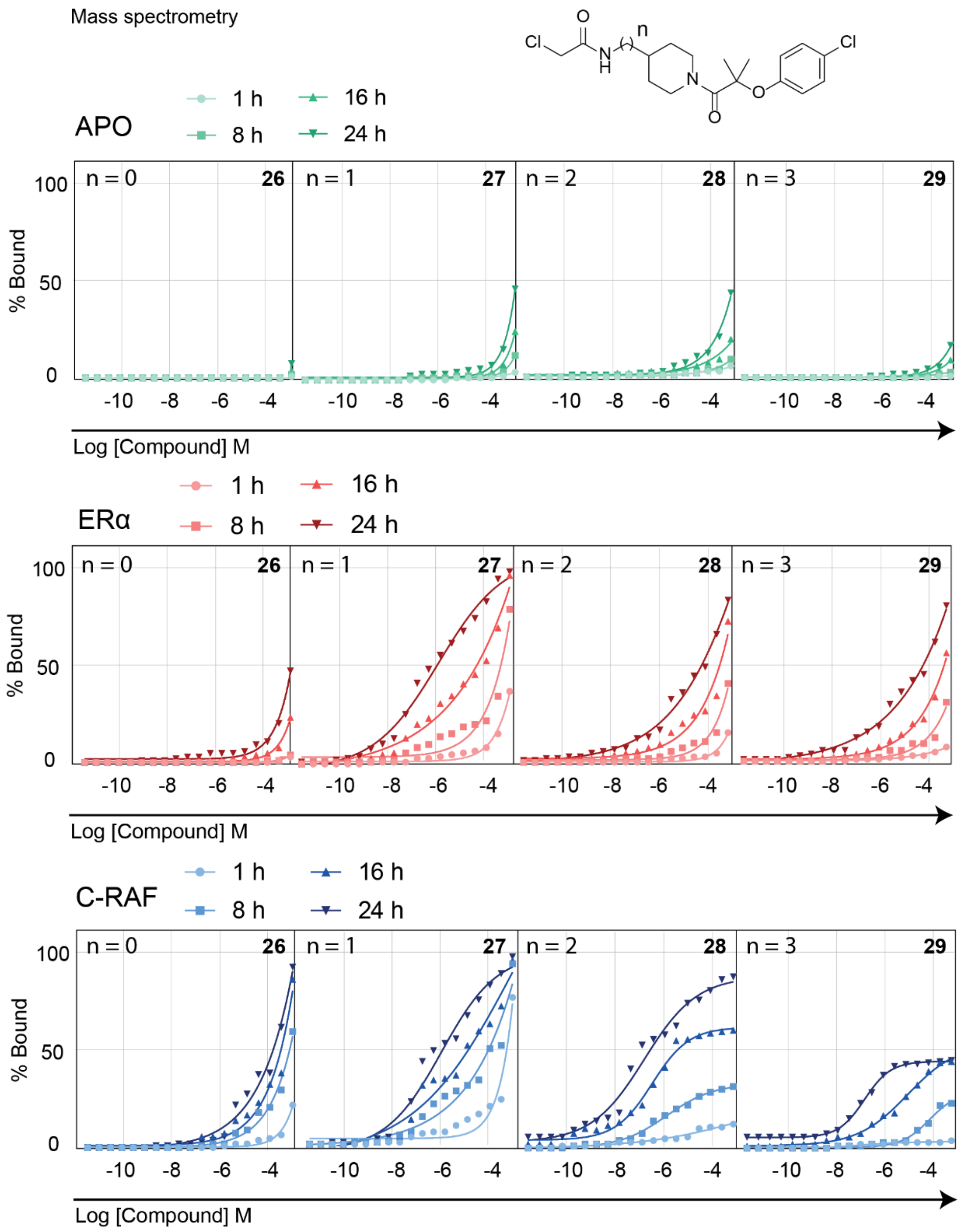

Figure S2: MS dose-response curves for chloroacetamides with varying linker length (n= 0-3, compounds discussed in Fig 2B).

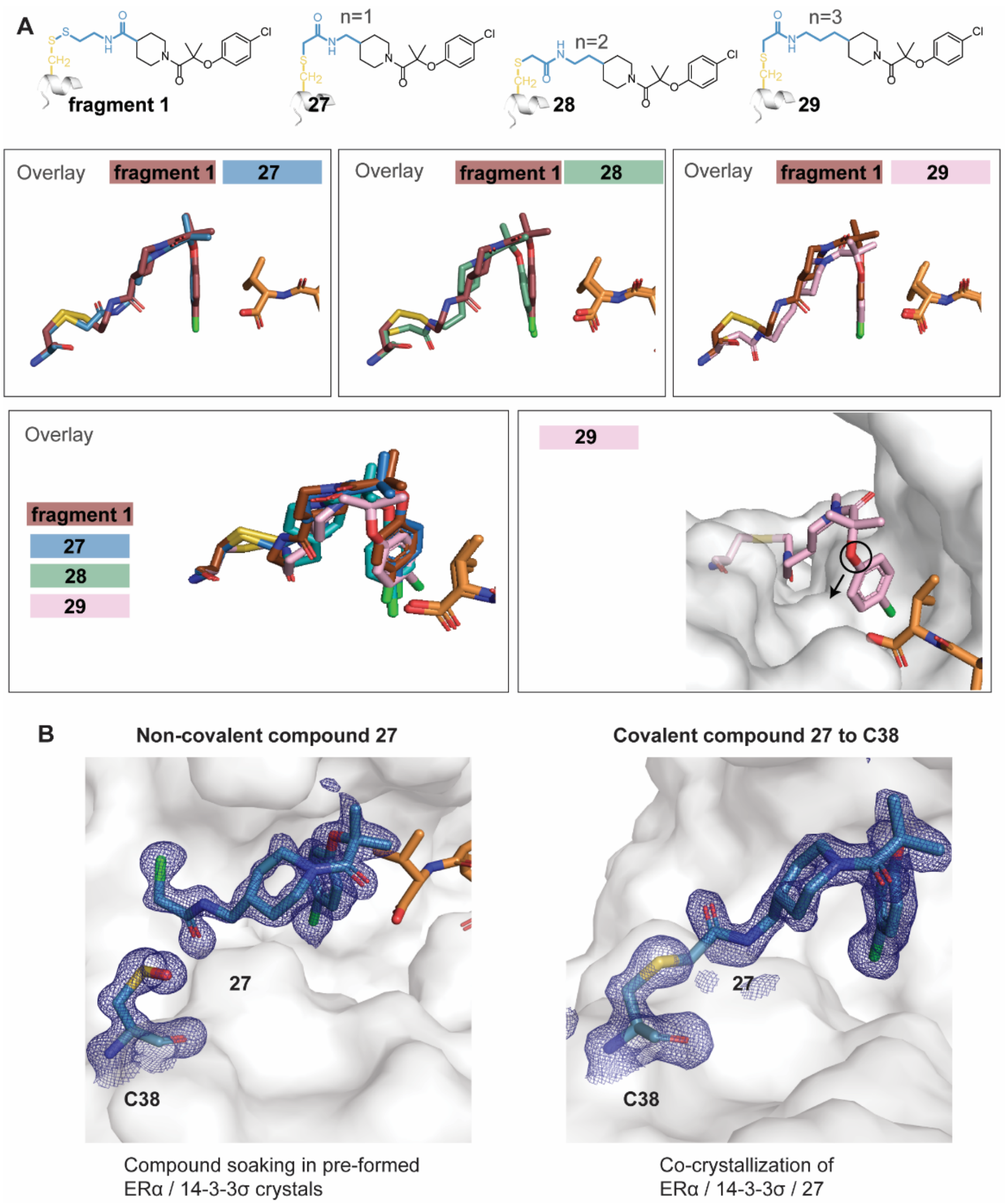

Figure S3: (A) Crystal structure overlays of **fragment 1** with compounds **27**, **28** and **29** with 14-3-3σ/ERα. (B) Non-covalent crystal structure of 14-3-3σ/ERα/**27** with the soaking method (left) and covalent crystal structure with co-crystallization (right). Comparison of the structures for compound 27 (soaking versus co-crystallization) did not indicate any differences in the protein or peptide conformations. In the crystal structure obtained by soaking, Cys38 was in oxidized form. Co-crystallization was done by overnight incubation with the compound, which allowed enough time for the covalent bond to form.

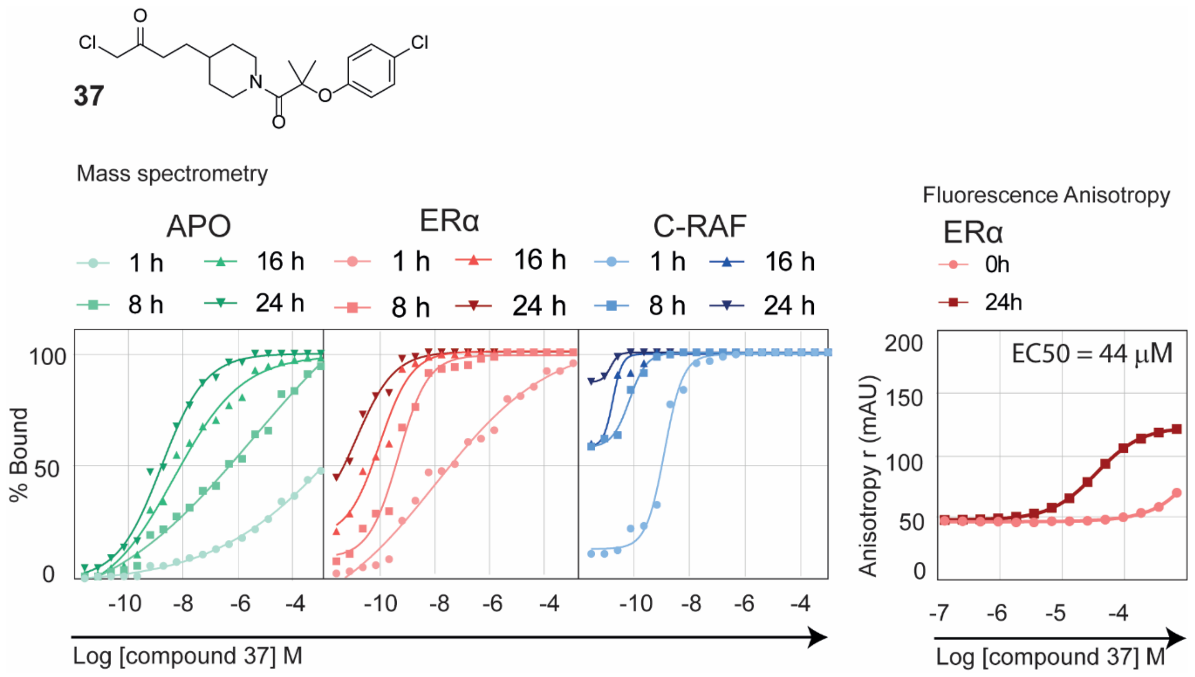

Figure S4: MS dose-response curves and FA dose-response curves for α-chloroketone **37**.

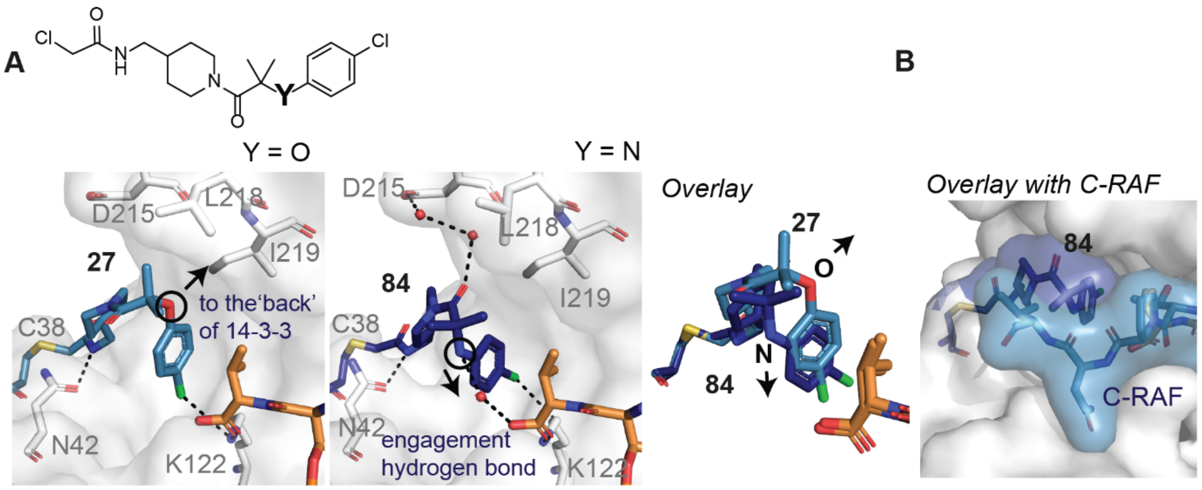

Figure S5: (A) Comparison of crystal structures of **27** (*gem*-dimethyl, ether, blue) with **84** (*gem*-dimethyl, aniline, purple) in complex with 14-3-3σ (white) and ERα (orange), and their overlay. (B) Crystal structure of **84** (purple) in complex with 14-3-3σ (white), overlayed with C-RAF peptide (blue).

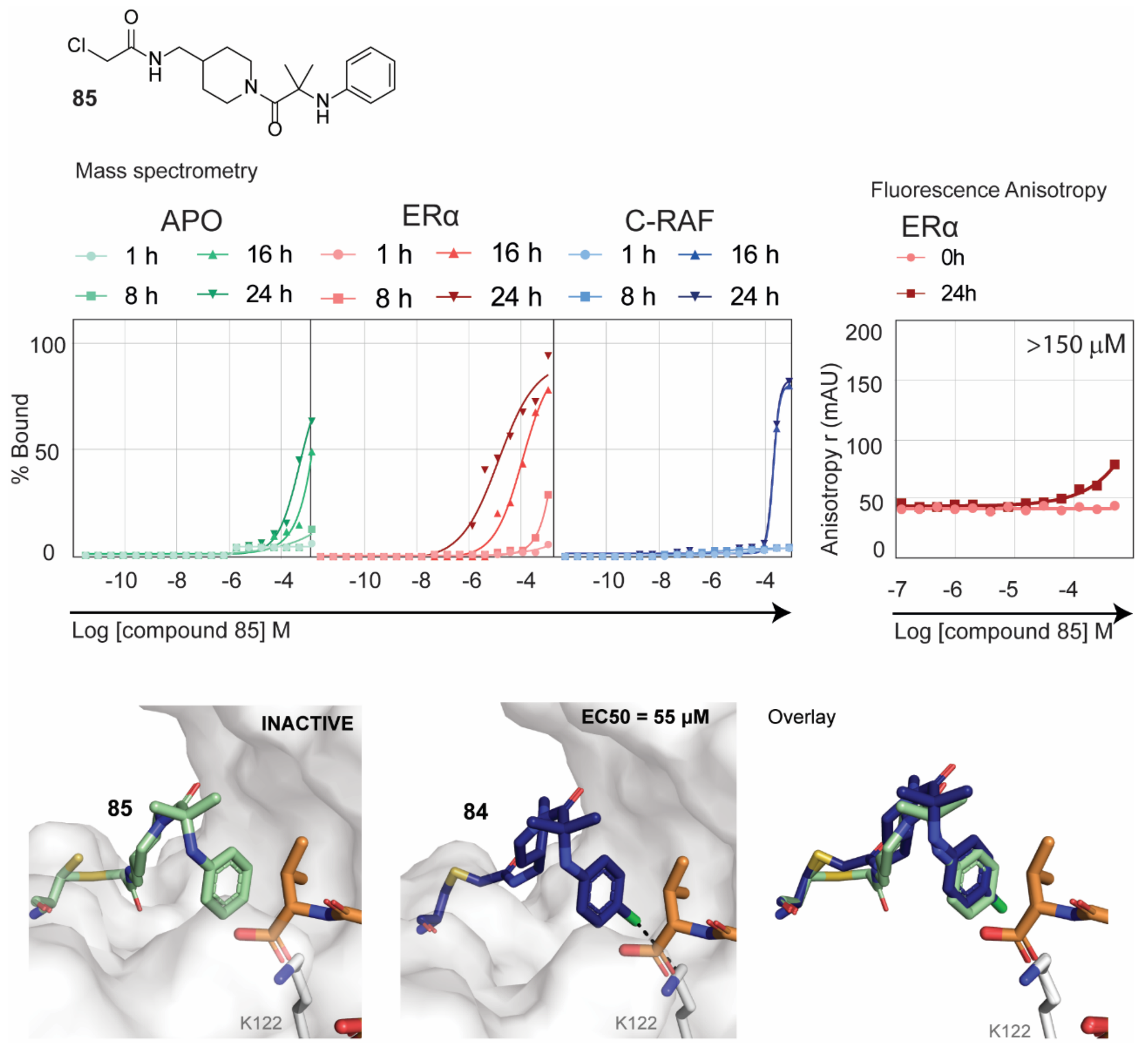

Figure S6: MS dose-response curves (left) and FA dose response curve (right) for **85** (*gem*-dimethyl, aniline, lacking the *p*-Cl substituent). For MS the compound is titrated to 14-3-3σ (100 nM) without peptide (apo, green), with ERα peptide (red) or with C-RAF peptide (blue). % bound to 14-3-3σ is measured at 1h, 8h, 16h and 24h. For the FA curve, compound is titrated to 14-3-3σ (1 μM) and ERα (10 nM) and anisotropy levels are measured at 0h and 24h. Comparison of crystal structures of **85** (green) and **84** (blue) in complex with 14-3-3σ (white) and ERα (orange).

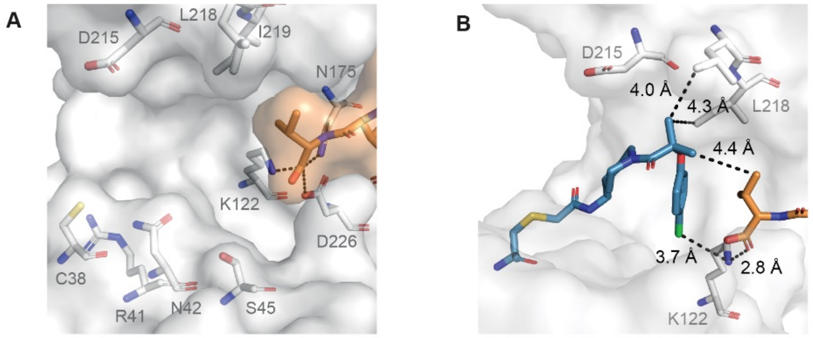

Figure S7: (A) Amino acid residues in the protein/peptide interface with ERα peptide, (B) interactions of **27** with 14-3-3σ/ERα.

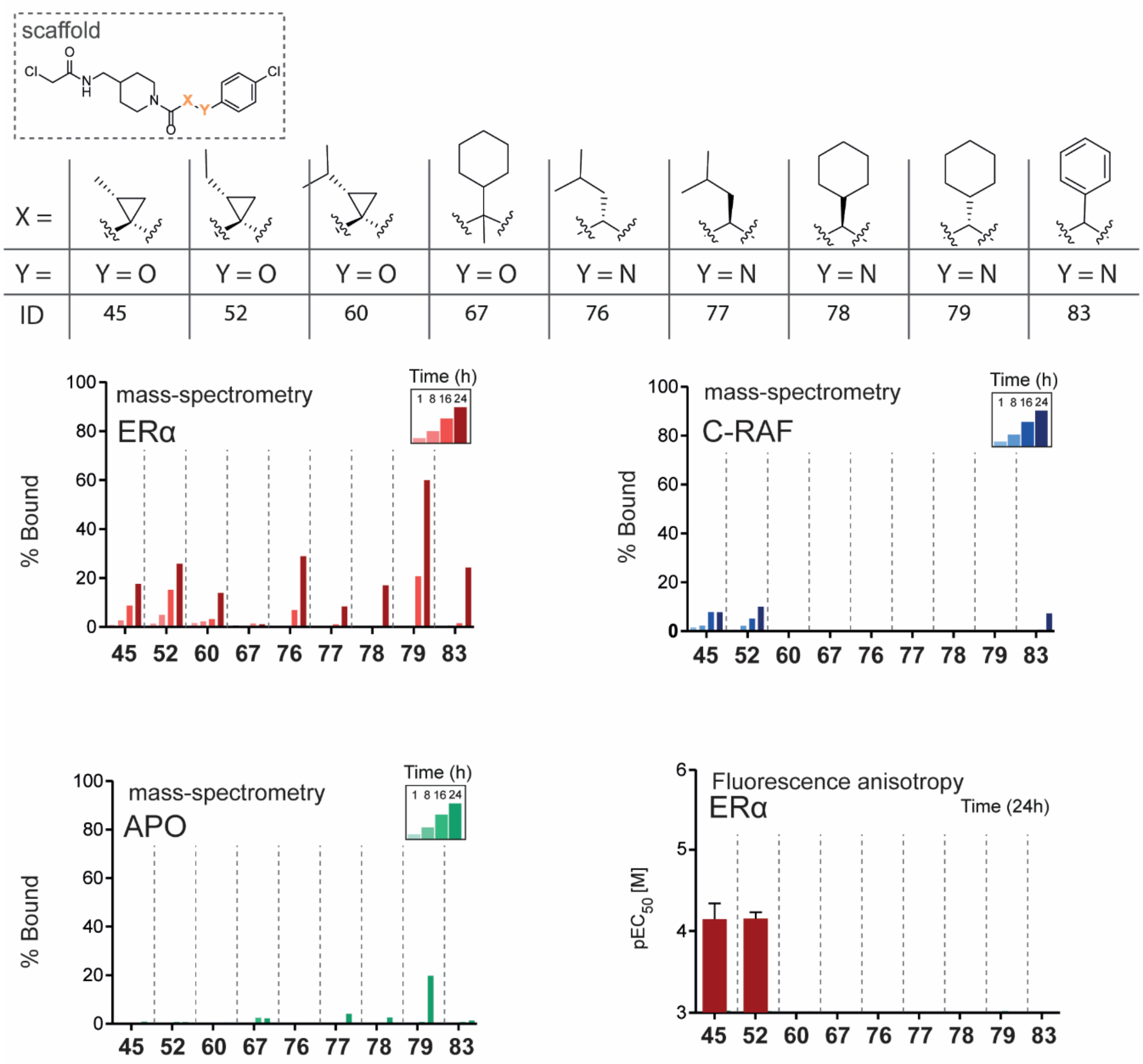

Figure S8: Analogs where the *gem*-dimethyl group was replaced with larger, aliphatic groups. MS and FA bar graphs, indicating inconsistent SAR between the two assay formats.

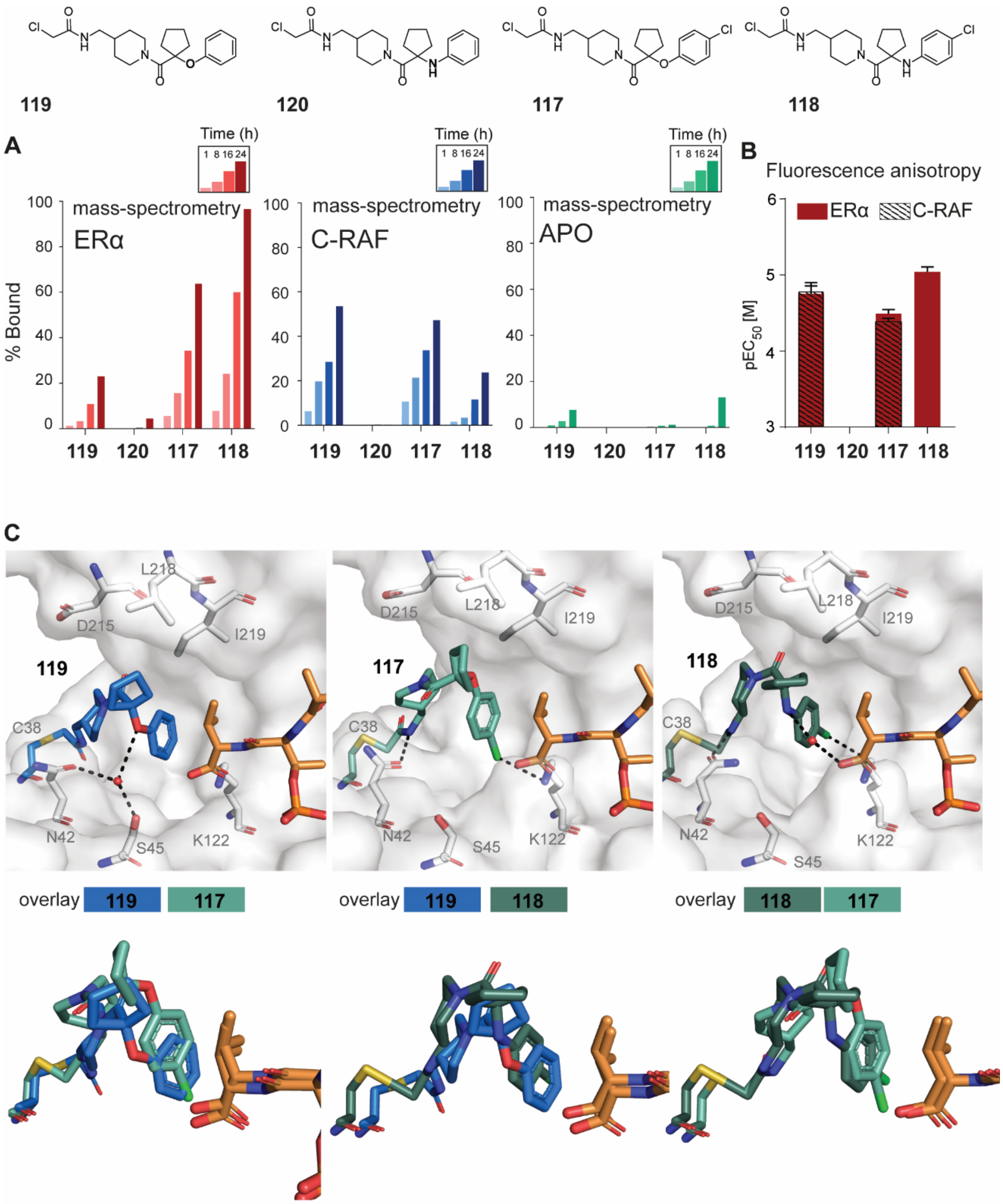

Figure S9: Comparison of cyclopentyl analogs **119**, **120**, **117** and **118**. (A) MS bar graphs % bound (1h, 8h, 16h, 24h) of 100 nM compound titrated to 100 nM 14-3-3σ in the presence of ERα (red), C-RAF (blue), or without peptide (apo, green). (B) EC_{50} value of FA compound titrations to ERα (10 nM, red) or C-RAF (10 nM, dashed lines) in the presence of 14-3-3σ. (C) Crystal structures of **119** (blue), **117** (light green), **118** (dark green) in complex with 14-3-3σ (white) and ERα (orange), and their overlay.

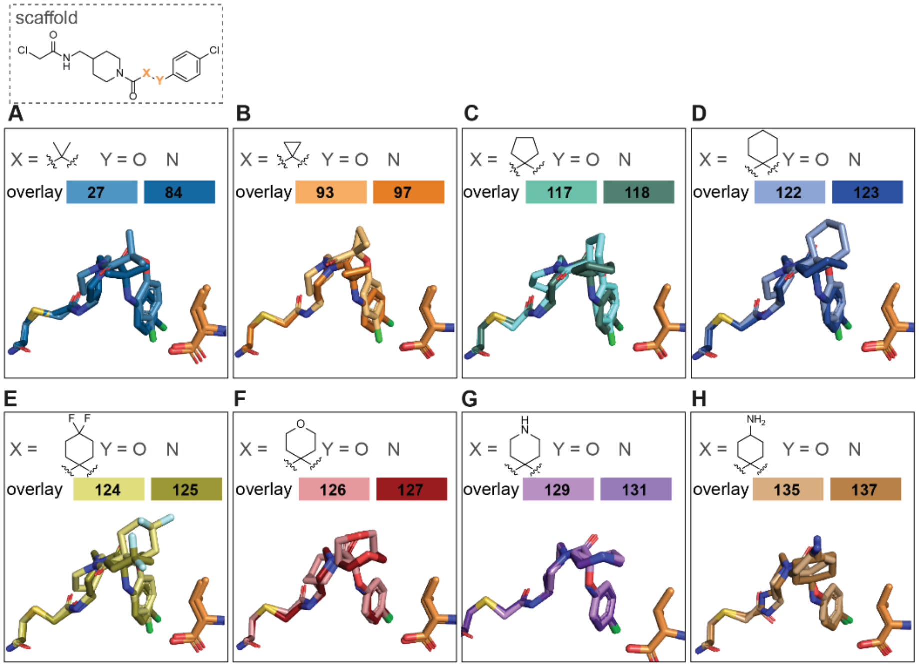

Figure S10: Crystal structures of compounds discussed in Fig 3 with 14-3-3σ/ERα, as alignments of ethers and anilines. For *gem*-dimethyl-, cyclopropyl-, cyclopentyl-, cyclohexyl- and *gem*-difluoro-cyclohexyl analogs, the ether was oriented in the back and the aniline in the front. For tetrahydropyrane-, piperidine- and aminocyclohexyl- analogs, both ether and aniline were oriented in the front.

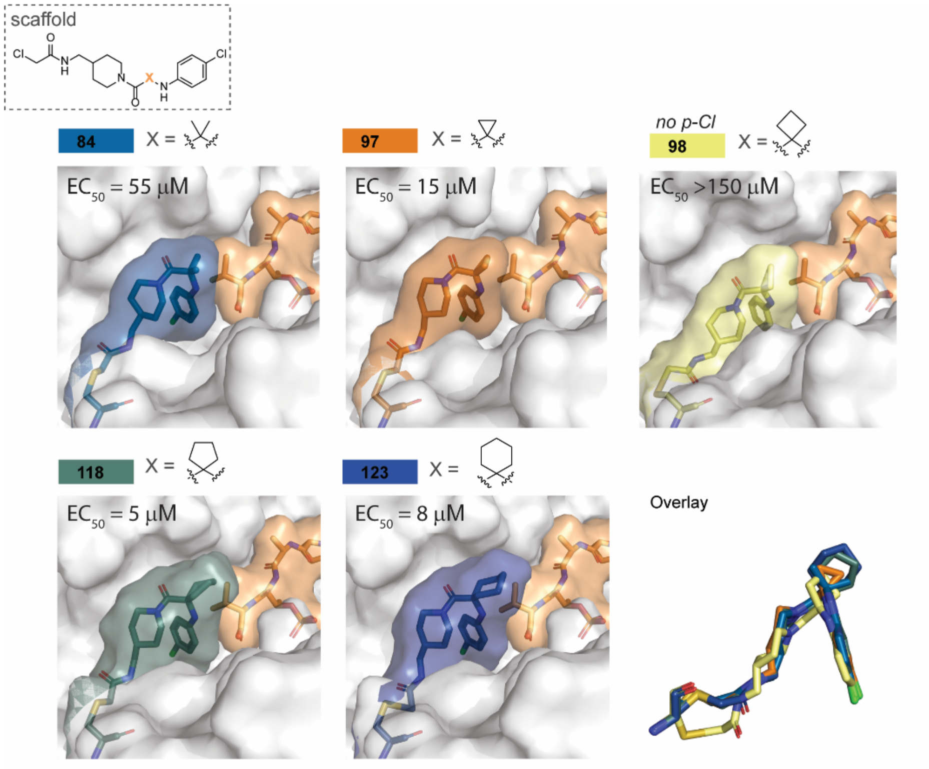

Figure S11: Crystal structures of aniline derivatives (discussed in Figure 3) in complex with 14-3-3σ (white) and ERα (orange). The cyclopentyl and cyclohexyl analogs formed more favorable interactions with both ERα and 14-3-3.

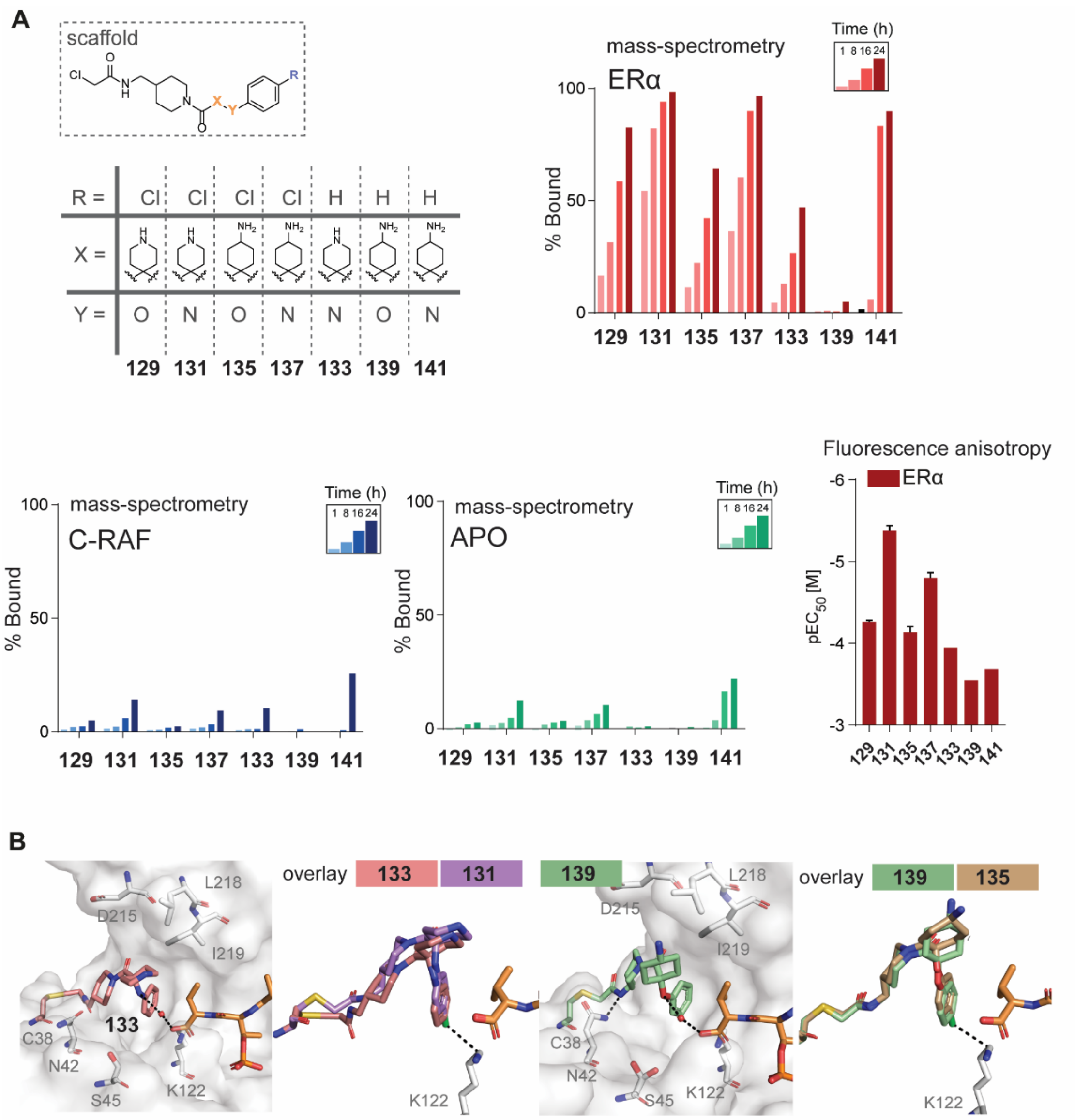

Figure S12: Close analogs of **131** and **137**. (A) MS and FA data, (B) crystal structures with 14-3-3σ/ERα.

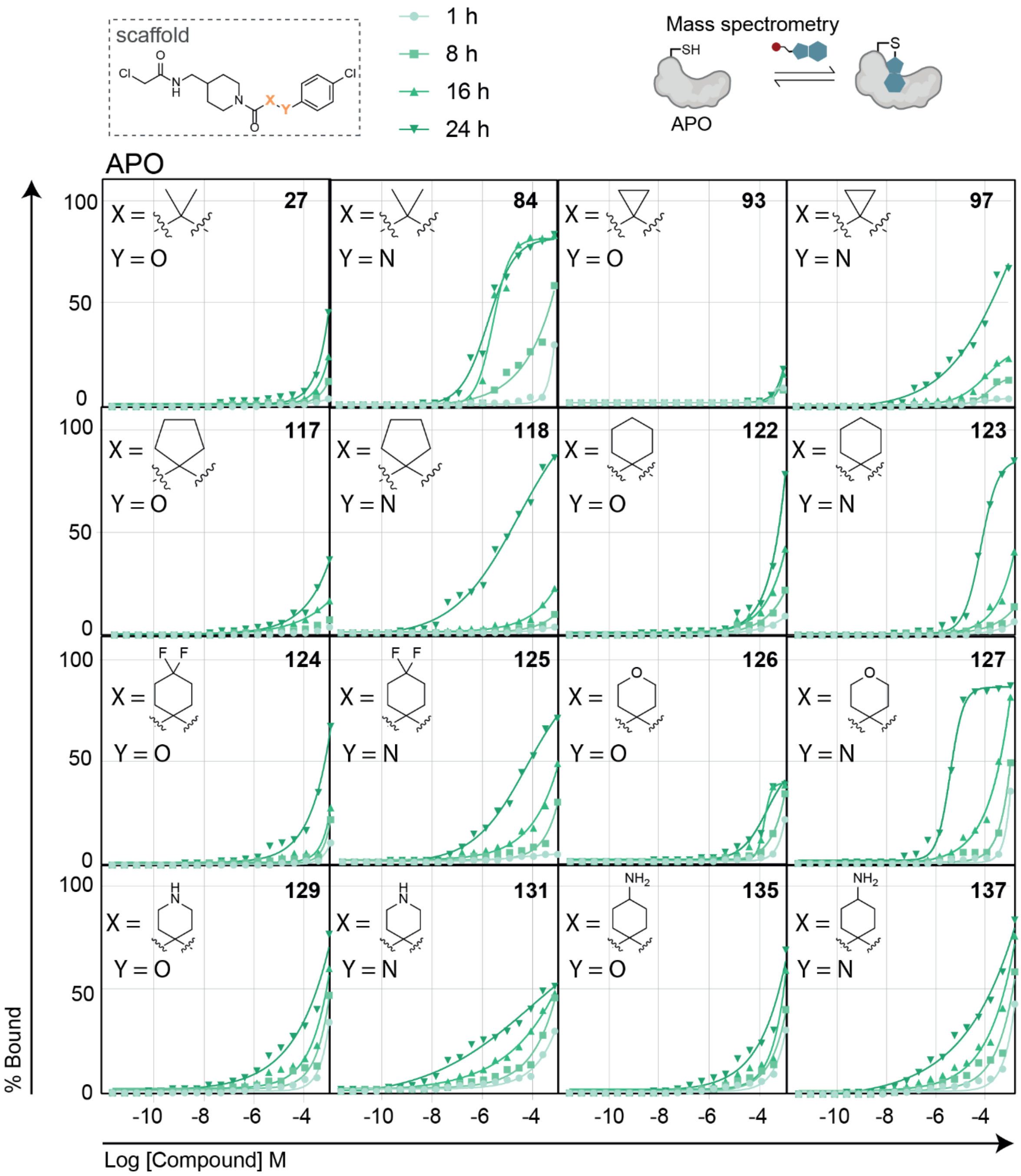

Figure S13: MS dose-response curves (apo) for compounds discussed in fig 3B.

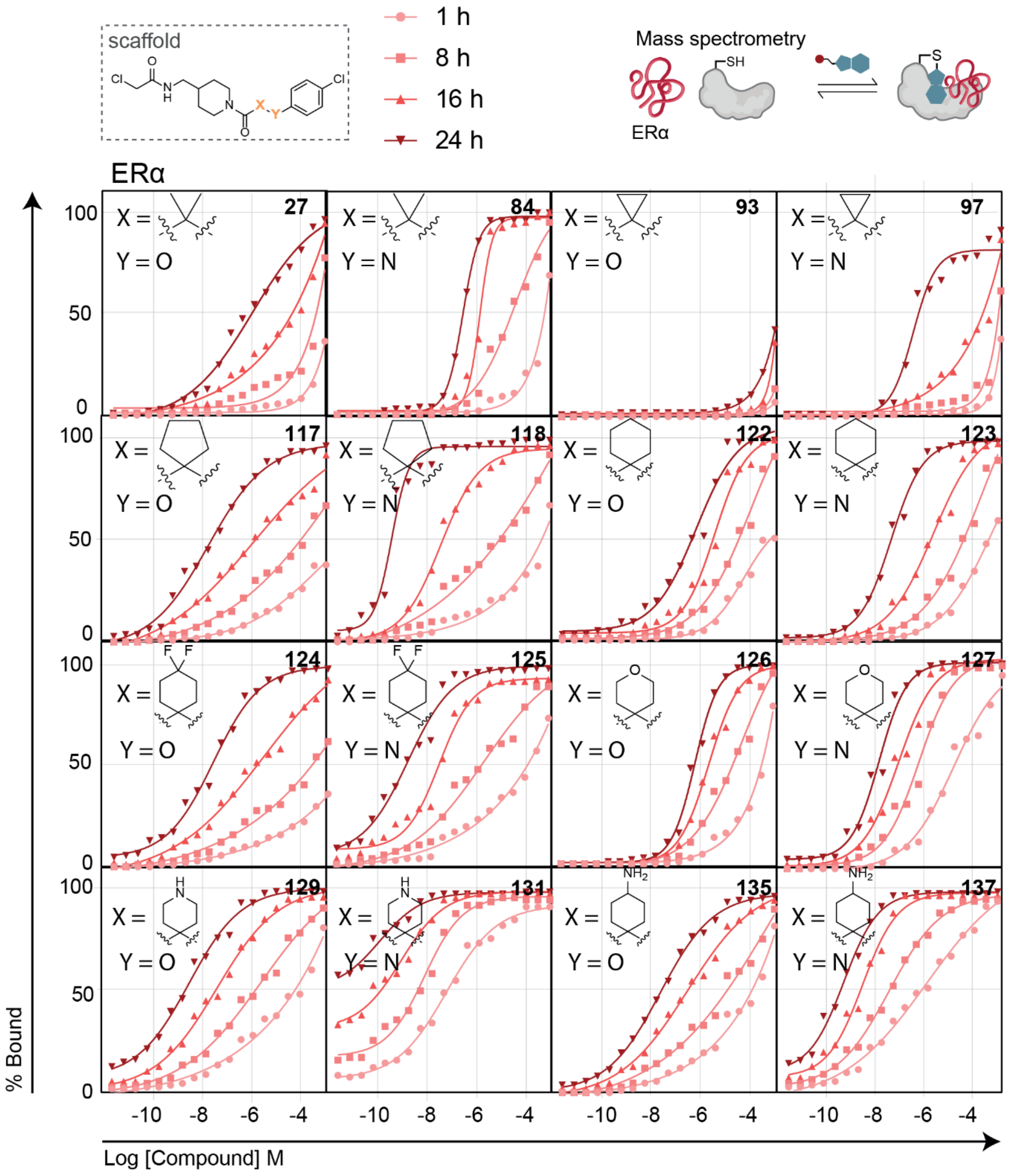

Figure S14: MS dose-response curves in the presence of ERα for compounds discussed in fig 3B.

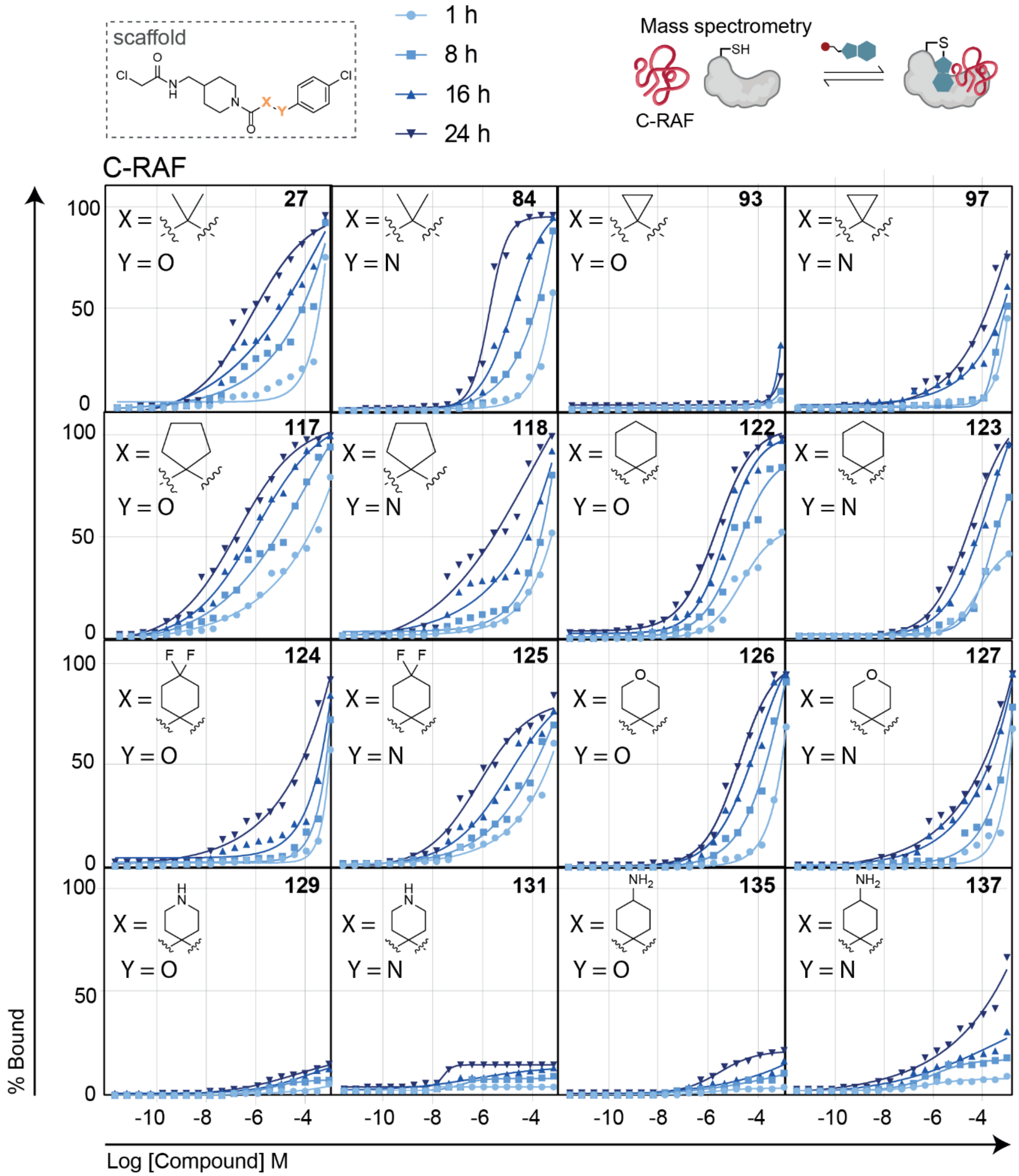

Figure S15: MS dose-response curves in the presence of C-RAF for compounds discussed in fig 3B.

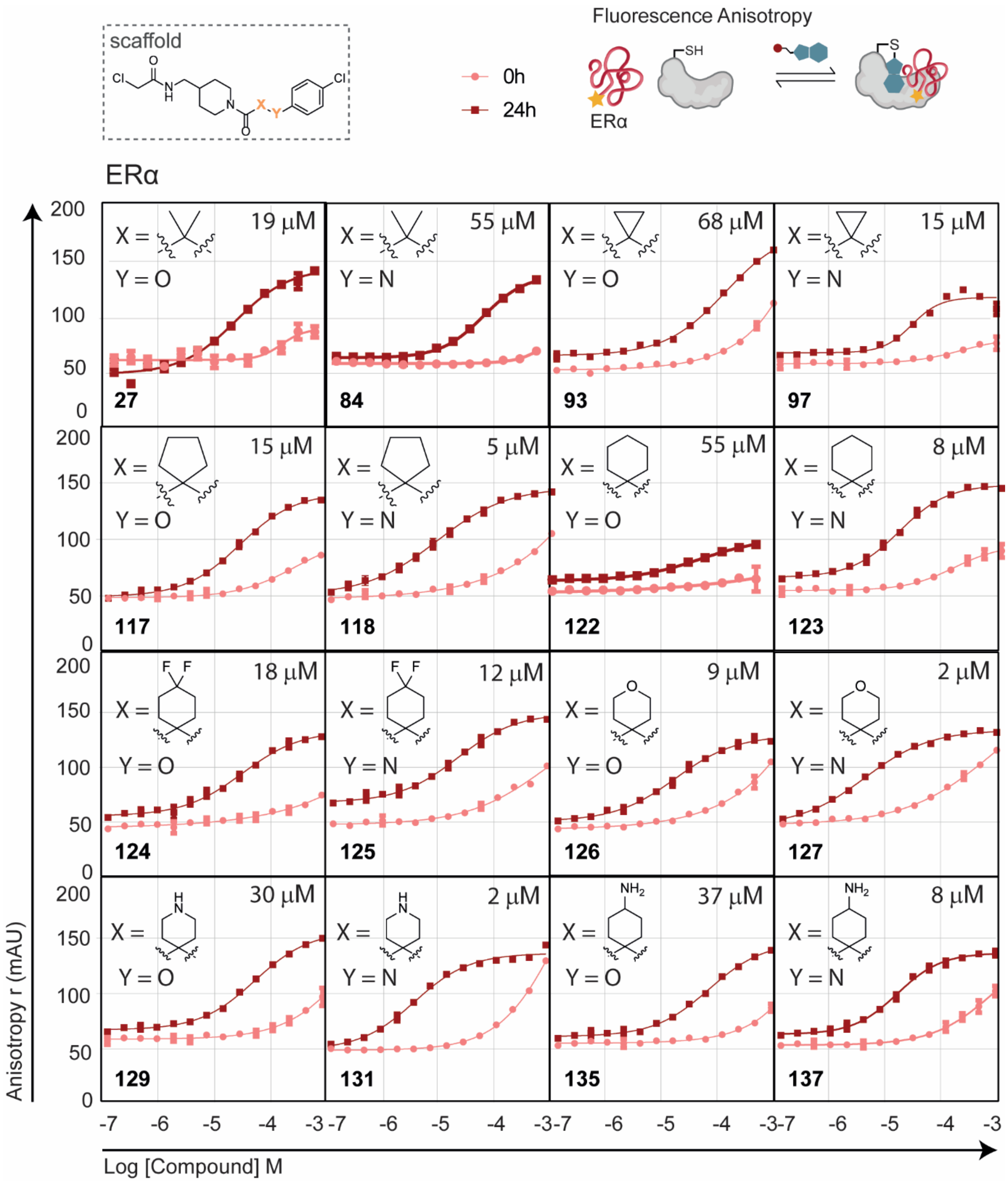

Figure S16: FA dose-response curves with ERα for compounds discussed in fig 3C.

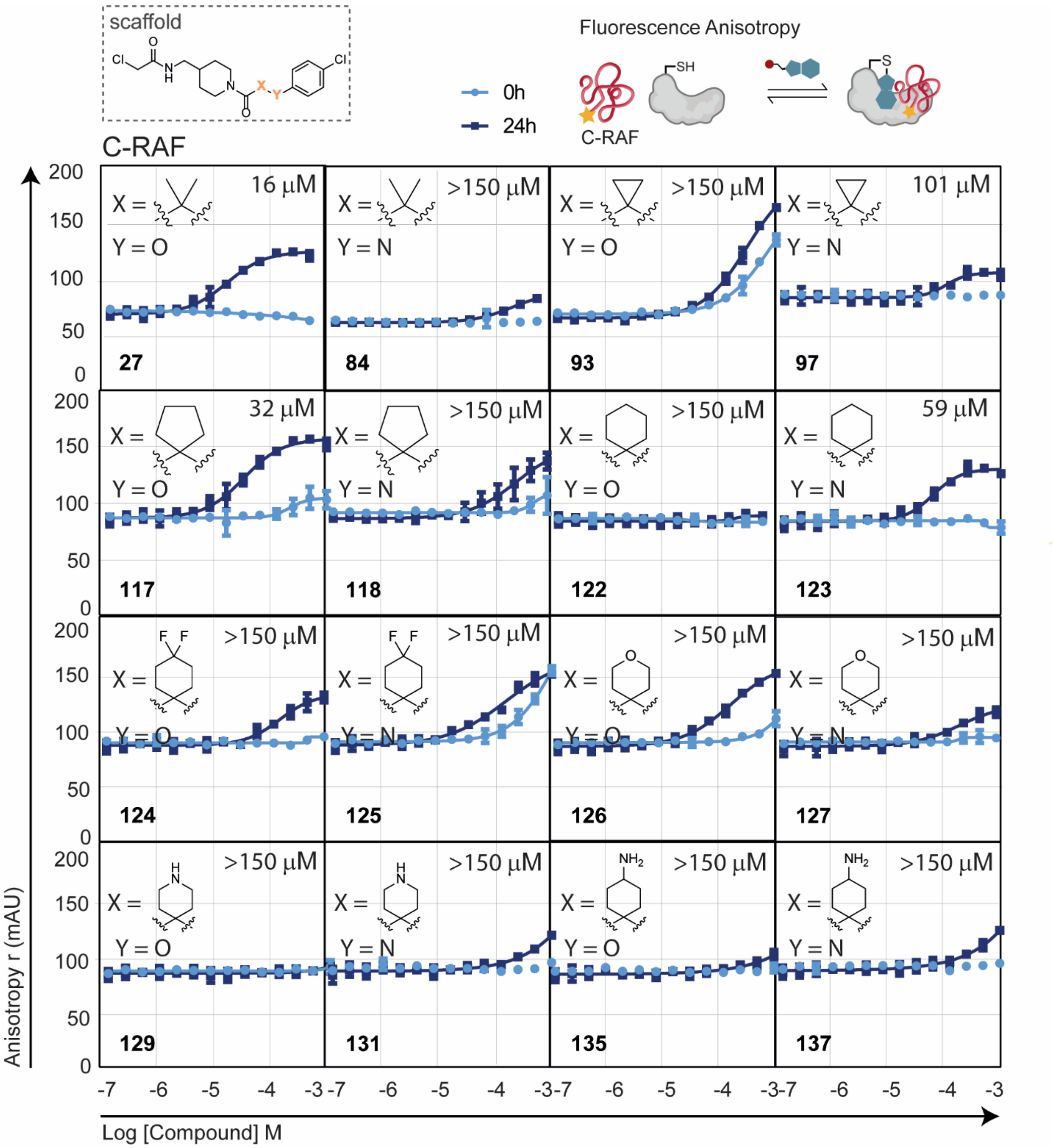

Figure S17: FA dose-response curves with C-RAF for compounds discussed in fig 3C.

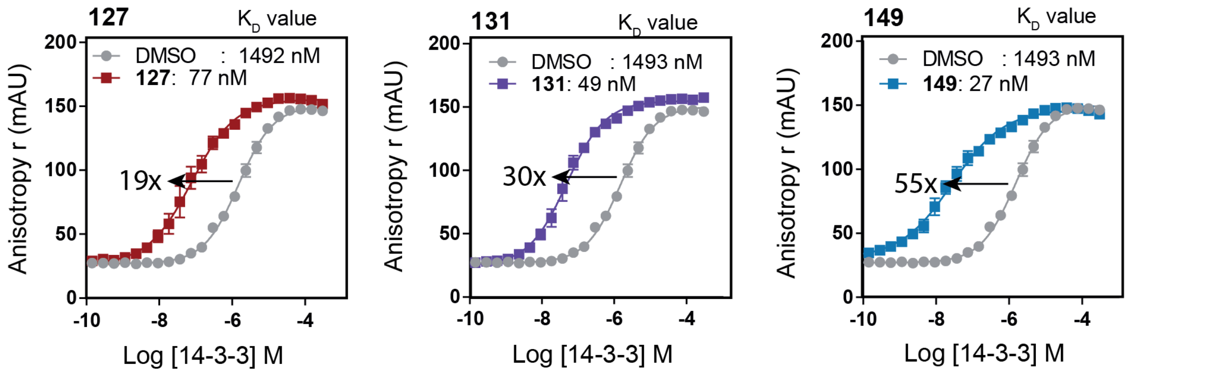

Figure S18: FA protein titrations for **127**, **131** and **149** at 100 µM compound with 10 nM of ERα-peptide. Measured K_D values are indicated in the legend.

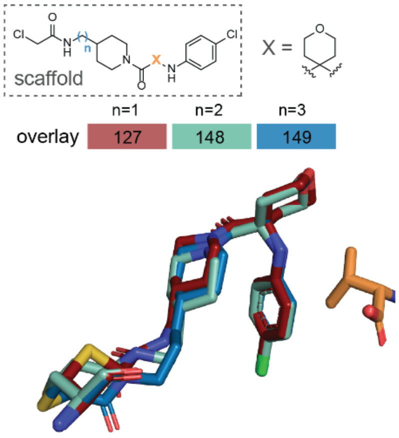

Figure S19: Overlay of crystal structures of tetrahydropyrans with varying linker lengths in complex with 14-3-3σ/ERα.

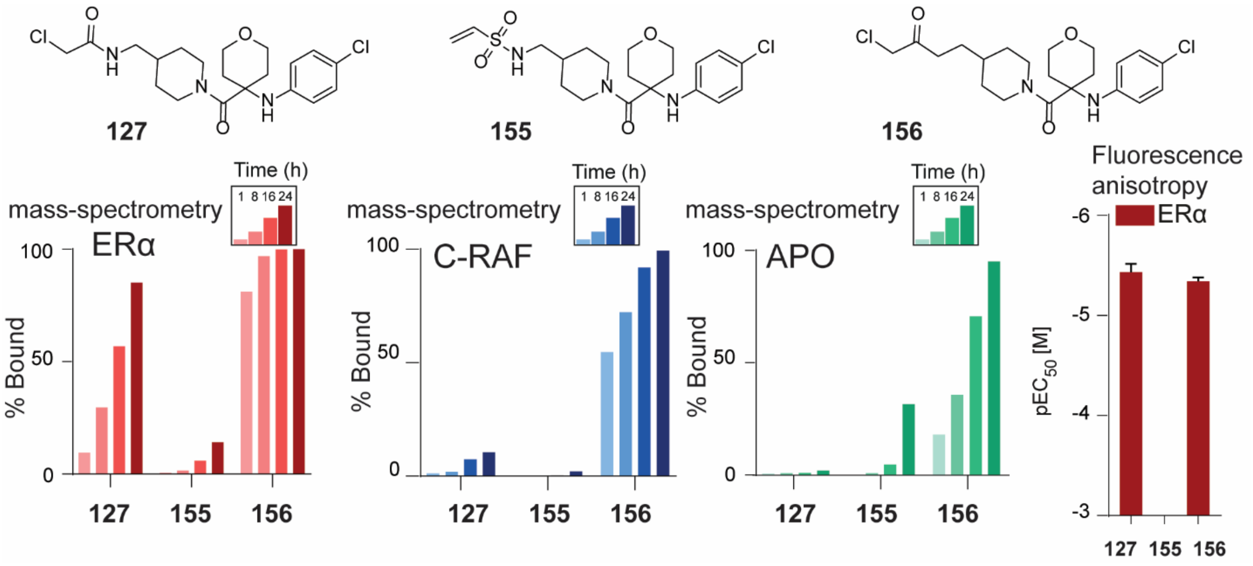

Figure S20: Bar graphs for MS and FA for the vinylsulfonamide **155** and the α-chloroketone **156**.

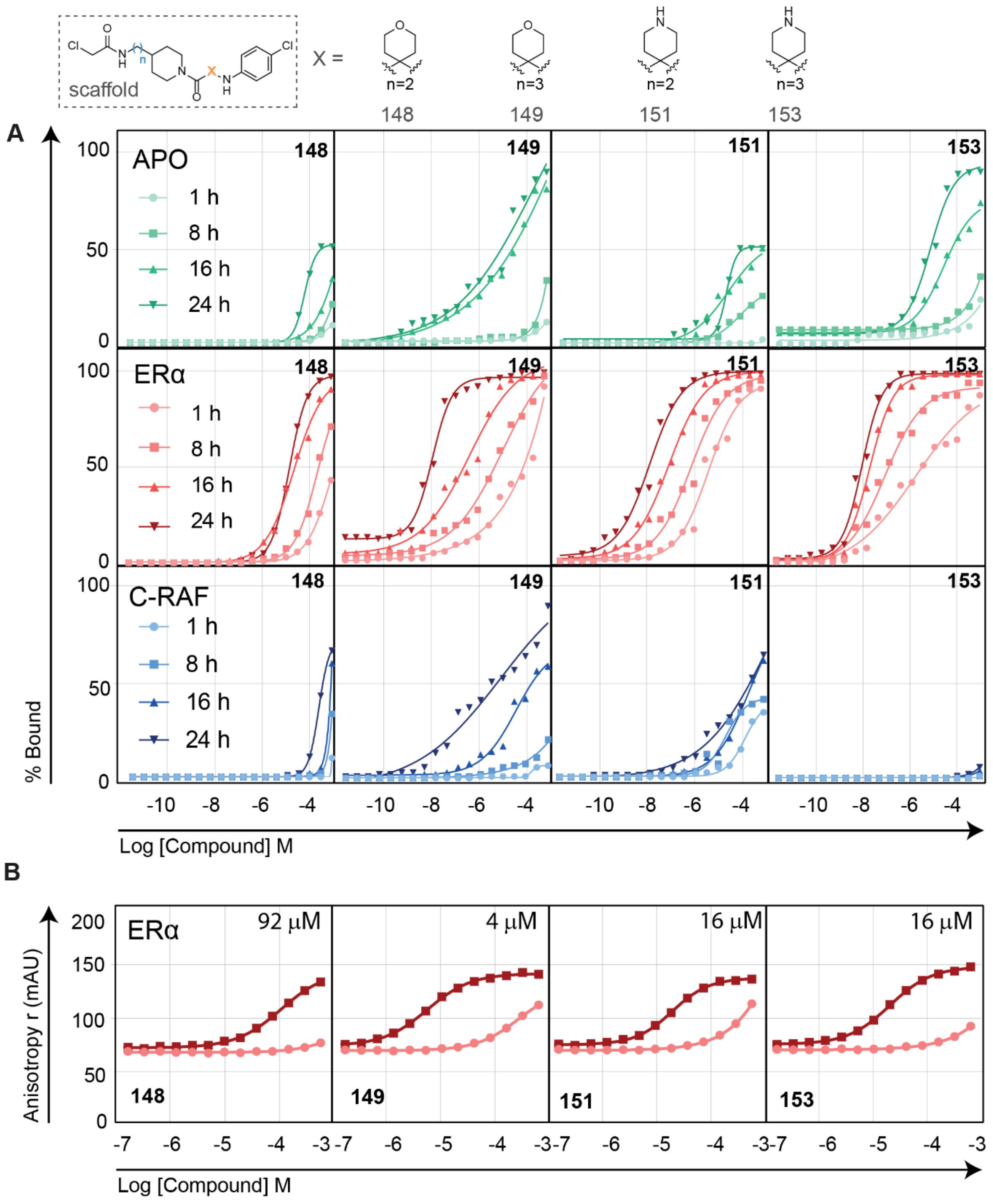

Figure S21: Warhead linker variations discussed in fig 4D and 4E. (A) MS dose-response curves and (B) FA dose-response curves.

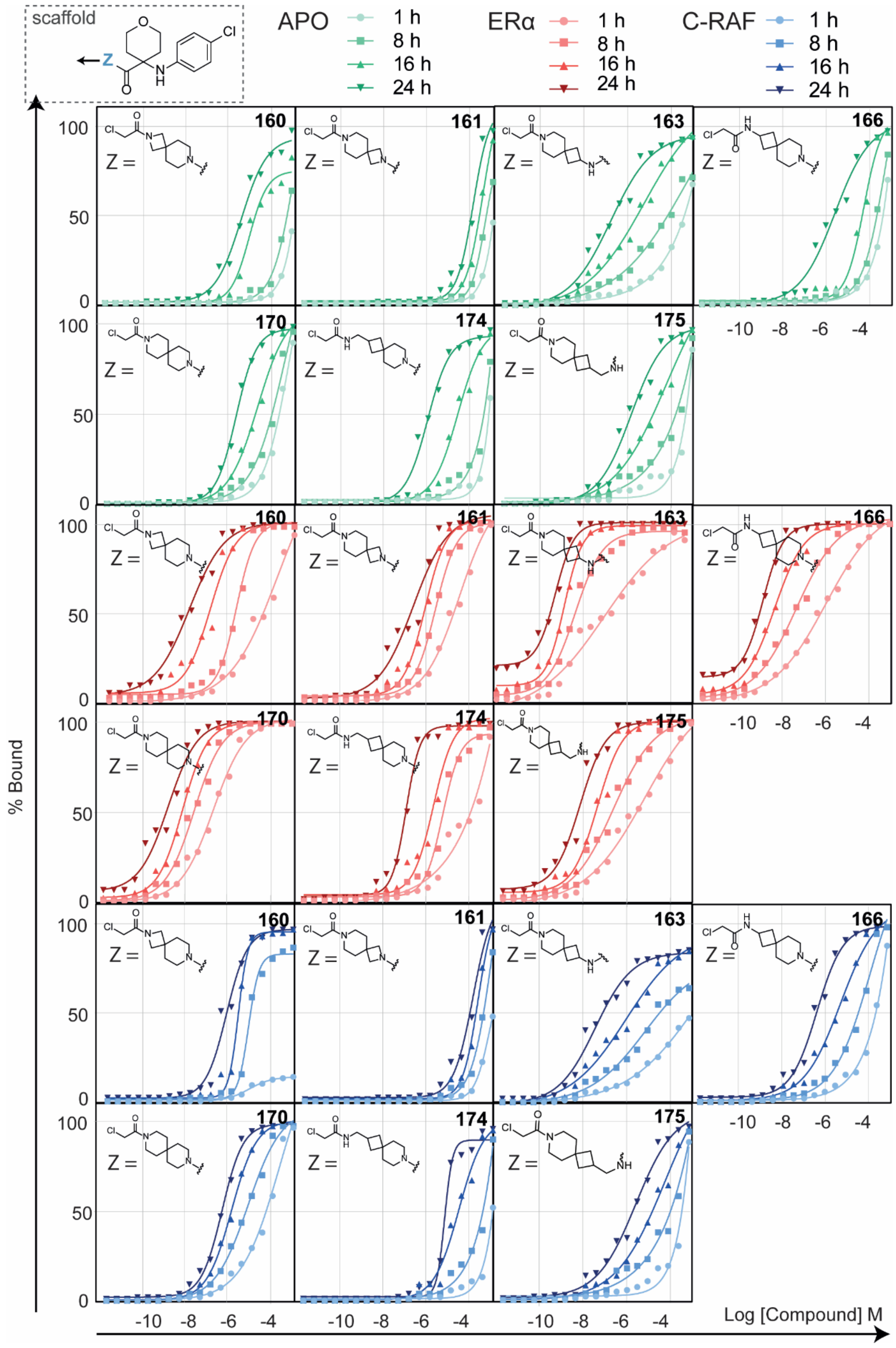

Figure S22: MS dose-response curves for the spiro analogs discussed in Fig 5B.

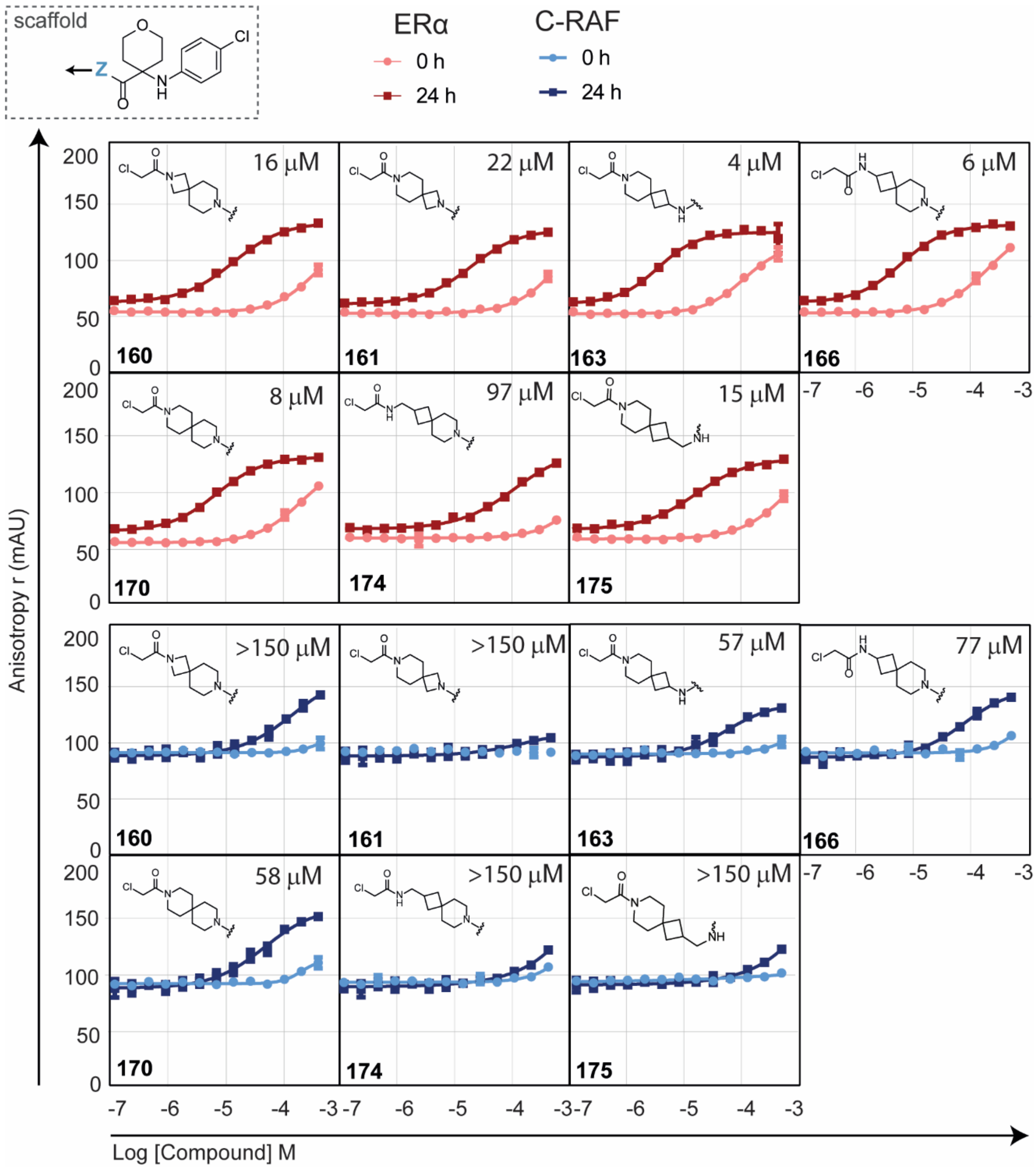

Figure S23: FA dose-response curves for the spiro analogs discussed in Fig 5C.

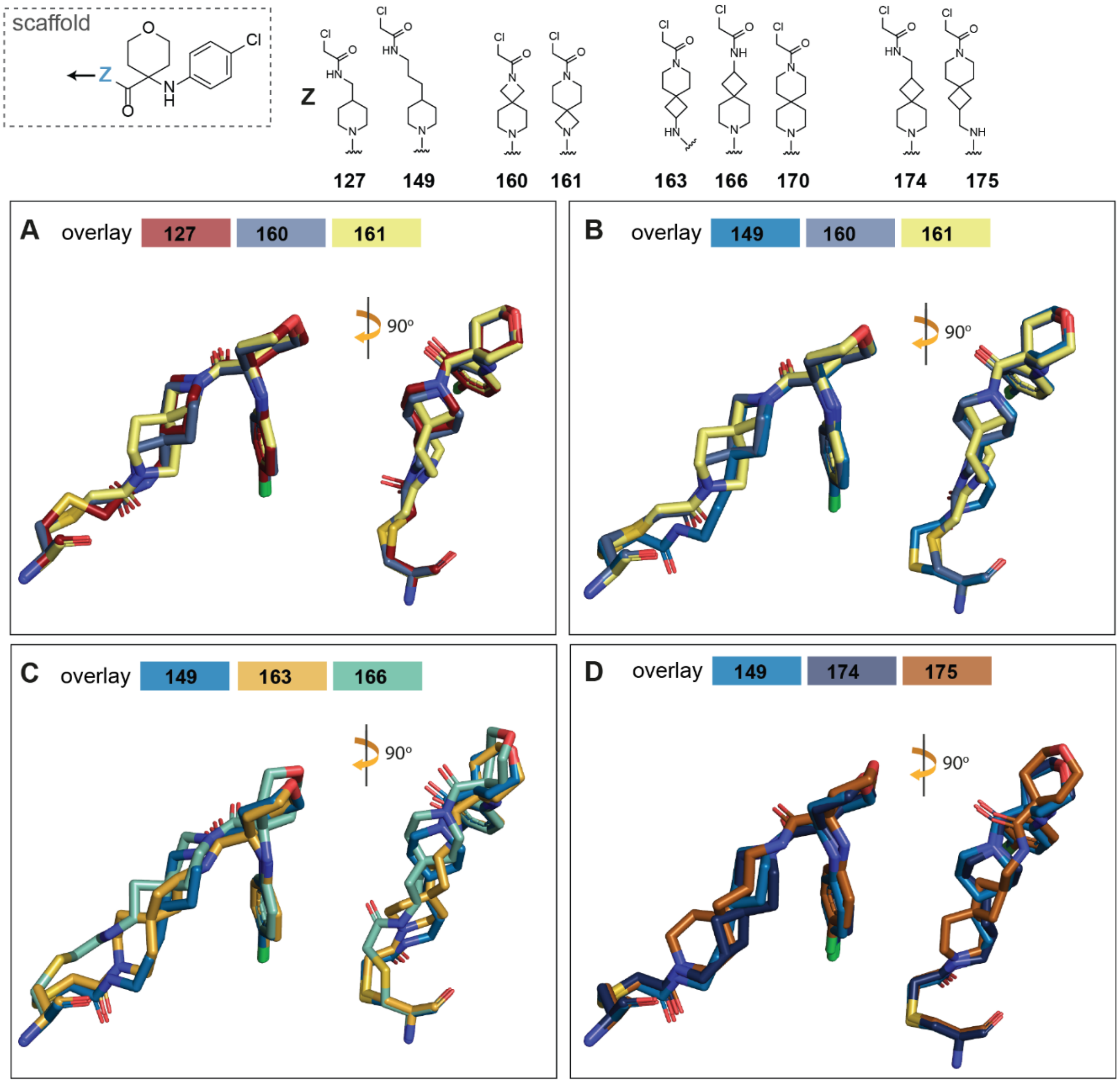

Figure S24: Crystallographic overlay of spiro-compounds discussed in fig. 5D-F in complex with 14-3-3σ/ERα.

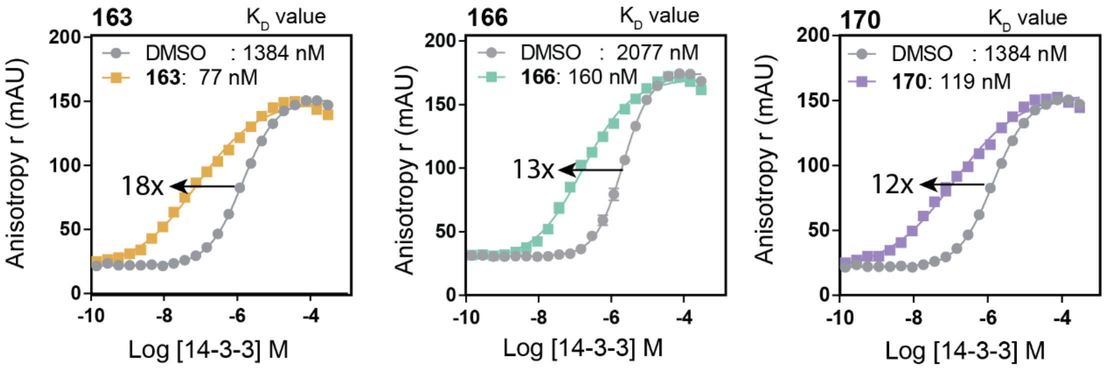

Figure S25: FA protein titrations for **163**, **166** and **170** at 100 µM compound. Measured K_D values are indicated in the legend.

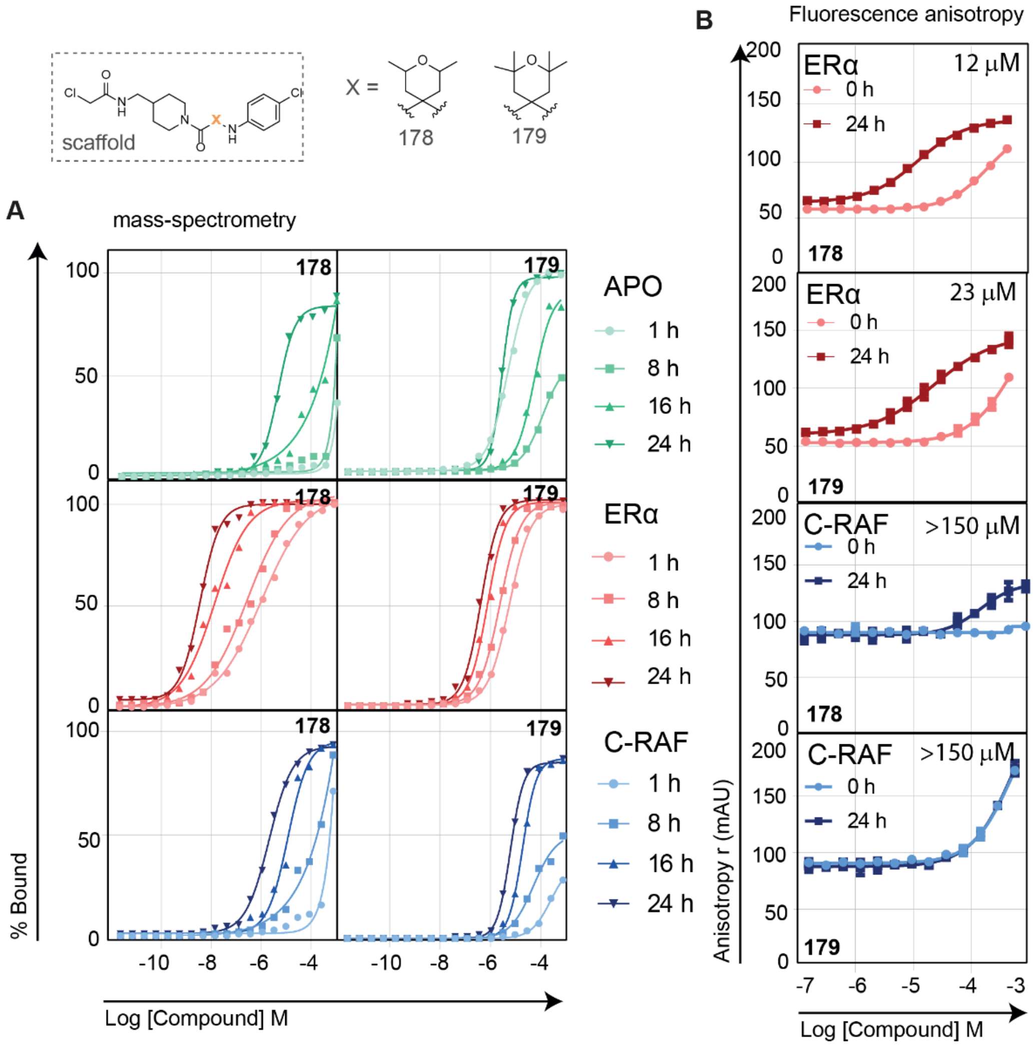

Figure S26: (A) MS dose-response curves and (B) FA dose response curves for methylated tetrahydropyran analogs discussed in Fig 6B and 6C.

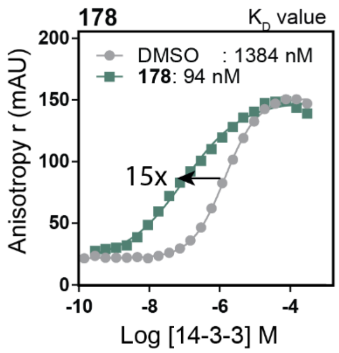

Figure S27: FA protein titration for **178** at 100 µM compound. Measured K_D values are indicated in the legend.

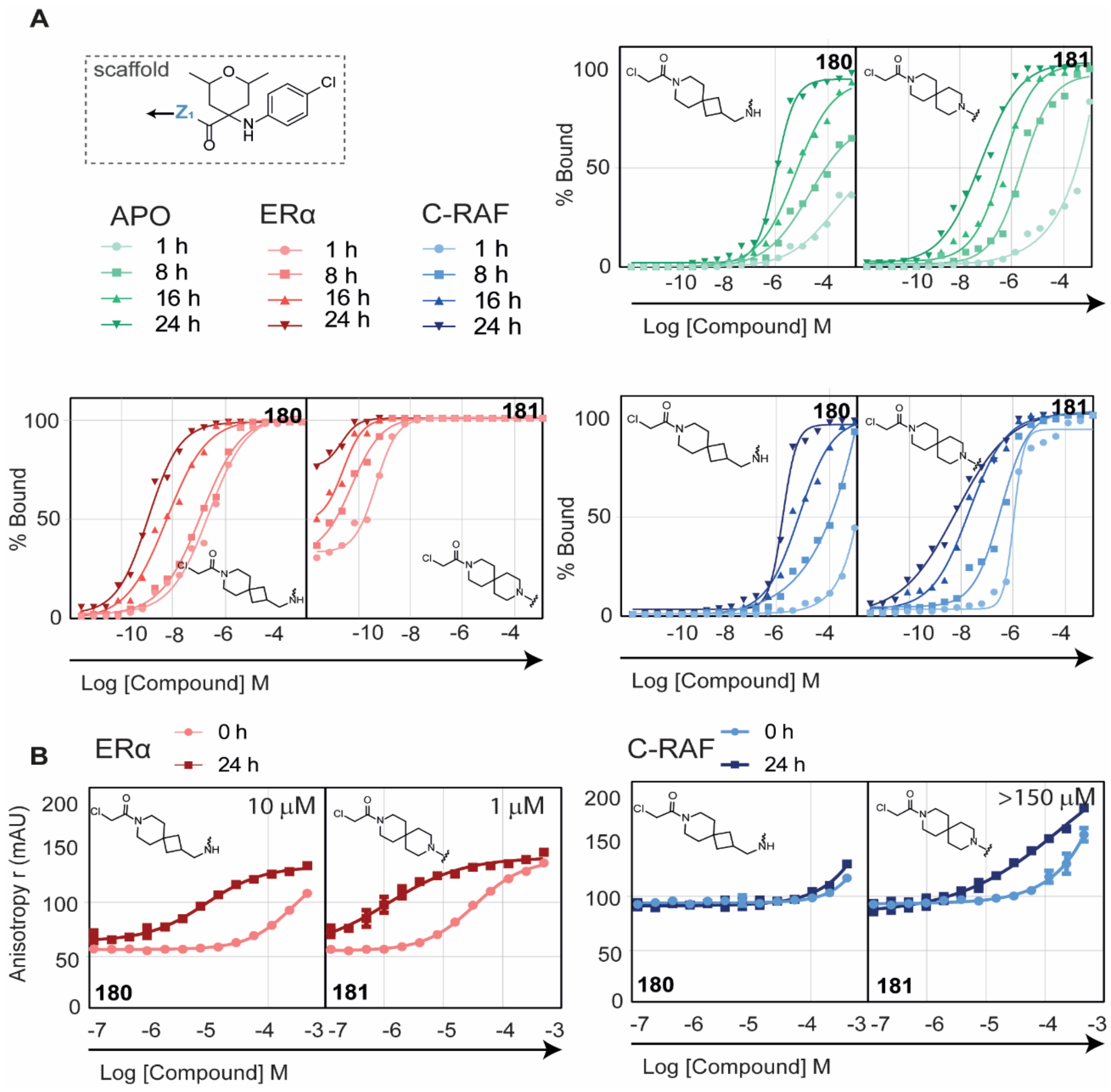

Figure S28: (A) MS dose-response curves and (B) FA dose-response curves for **180** and **181**.

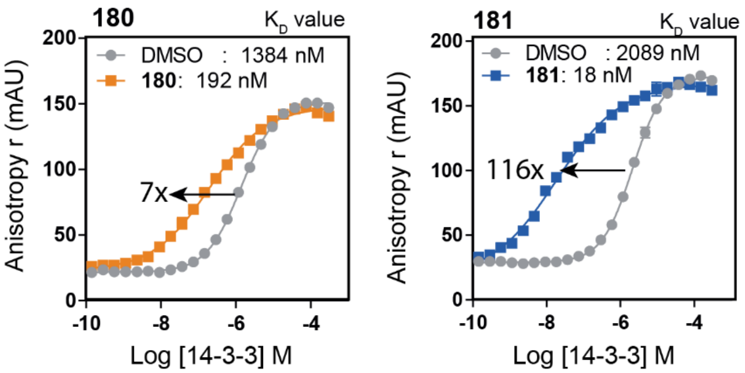

Figure S29: FA protein titrations for **180** and **181** at 100 μM compound. Measured K_D values are indicated in the legend.

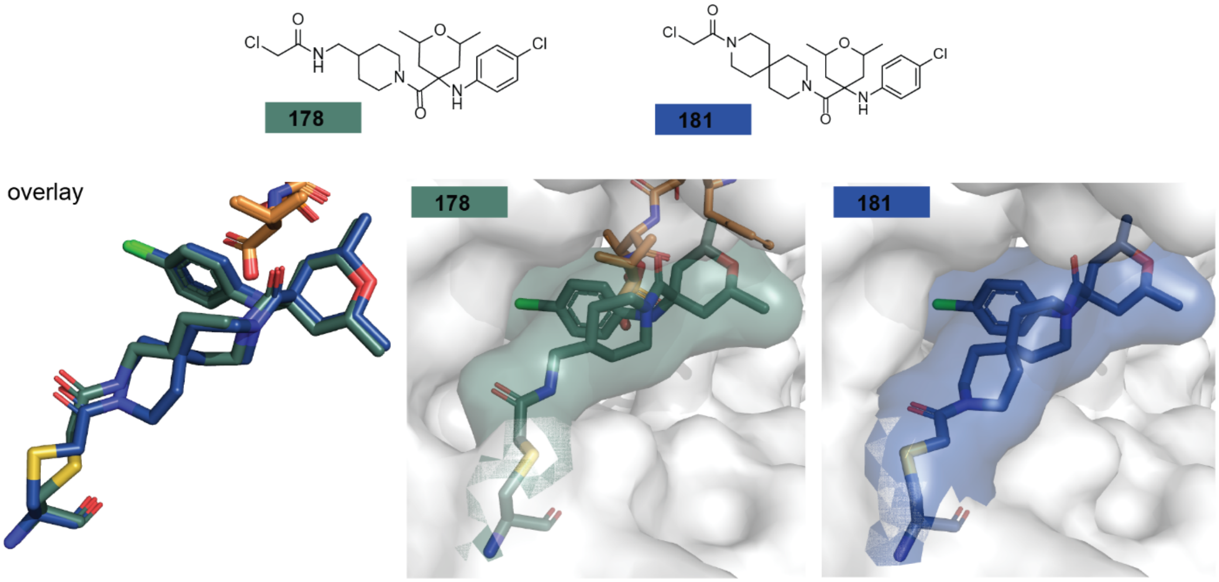

Figure S30: Crystallographic overlay and space-filling model of crystal structures of **178** and **181** with 14-3-3σ/ERα.

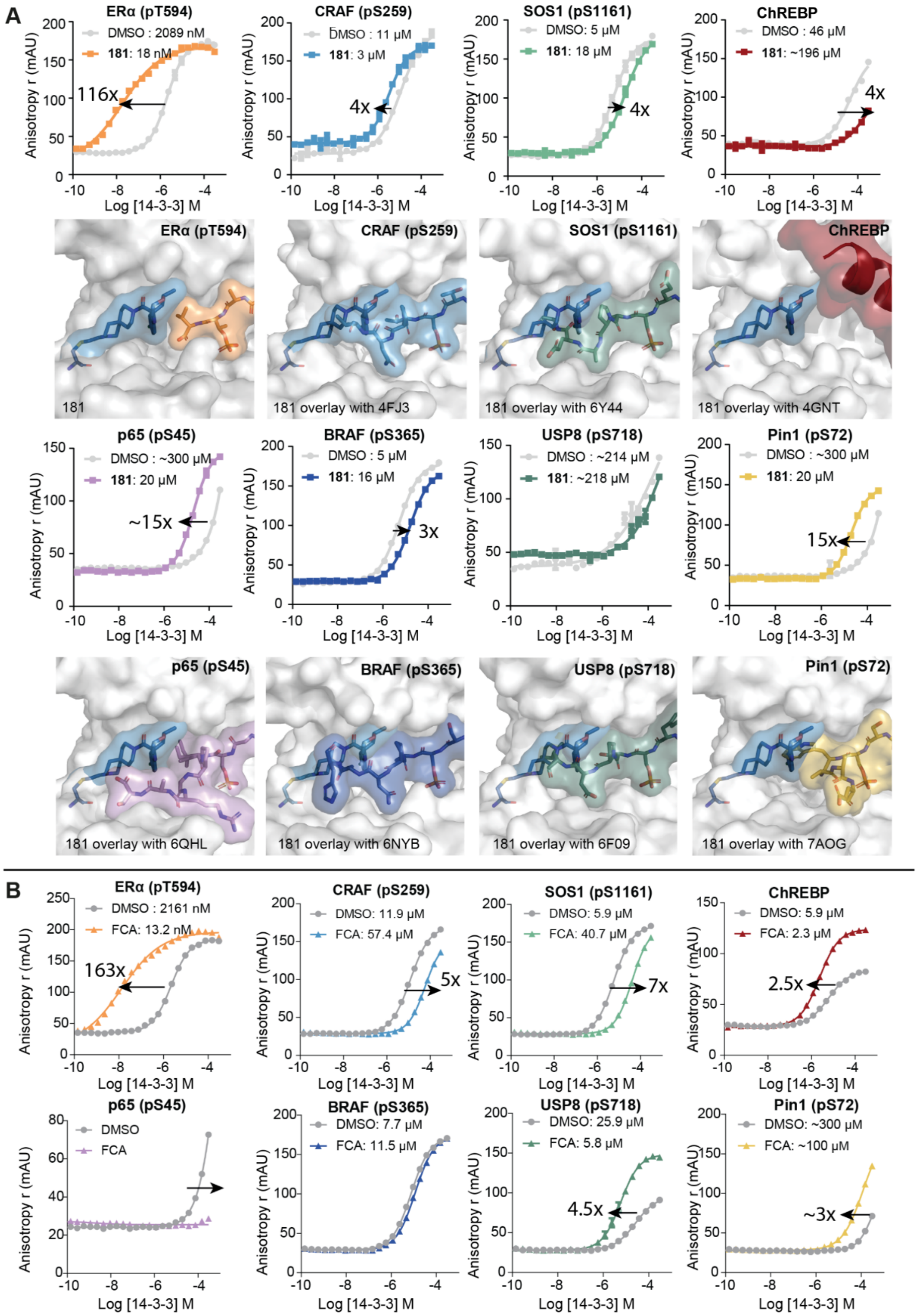

Figure S31: Selectivity studies of compound **181** (A) and FC-A (B) measured in FA protein titrations. 14-3-3σ was titrated to eight different FAM-labeled peptides (ERα, C-RAF, SOS1, ChREBP, p65, B-RAF, USP8 and Pin1, each 10 nM) in the presence of DMSO (1%) or **181** or FC-A (100 μM). Apparent K_d values (stated in each legend) were determined for the interaction of each peptide with 14-3-3σ, in the presence of DMSO or **181** or FC-A, resulting in a calculated fold-stabilization by **181** or FC-A shown at the arrow of each graph. The crystal structure of **181** complexed with 14-3-3σ and ERα (top left) was overlayed with the known

crystal structures of the measured peptides (CRAF (4FJ3), SOS1 (6Y44), ChREBP (4GNT), p65 (6QHL), BRAF (6NYB), USP8 (6F09) and Pin1 (7AOG)).

Replicate 1

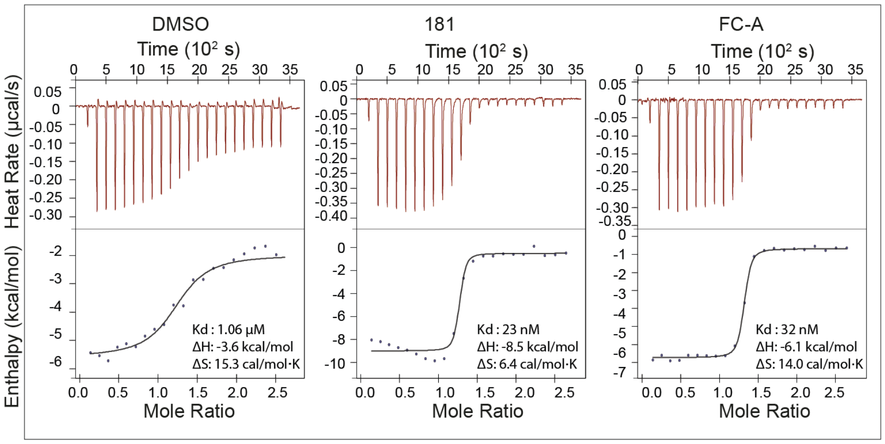

Replicate 2

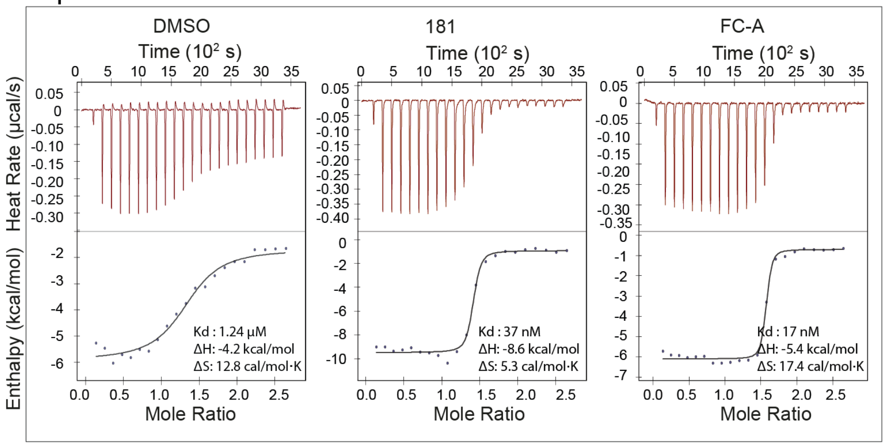

	DMSO R1	DMSO R2	181 R1	181 R2	FC-A R1	FC-A R2
**Blank (μcal)**	-1.19032	-1.01084	-0.28981	-0.54994	-0.40659	-0.41400
**Kd (M)**	1.059E-6	1.238E-6	2.300E-8	3.744E-8	3.161E-8	1.733E-8
**n**	1.216	1.321	1.214	1.354	1.261	1.513
**ΔG (kcal/mol)**	-8.15	-8.06	-10.42	-10.13	-10.23	-10.59
**ΔH (kcal/mol)**	-3.602	-4.238	-8.507	-8.560	-6.052	-5.412
**ΔS (cal/mol*K)**	15.26	12.82	6.418	5.272	14.02	17.36

Figure S32: ITC thermograms replicates as discussed in fig 7H and derived thermodynamic values. 300 μM of ERα-peptide was titrated to 30 μM of 14-3-3σ in the presence of DMSO or 500 μM **181** or **FC-A**. A constant blank model is used to correct for heat of injection and an independent model is used to model peptide binding. From these data, the thermodynamic parameters are determined as noted in the table. Each peptide is measured in two independent experiments (R1 and R2).

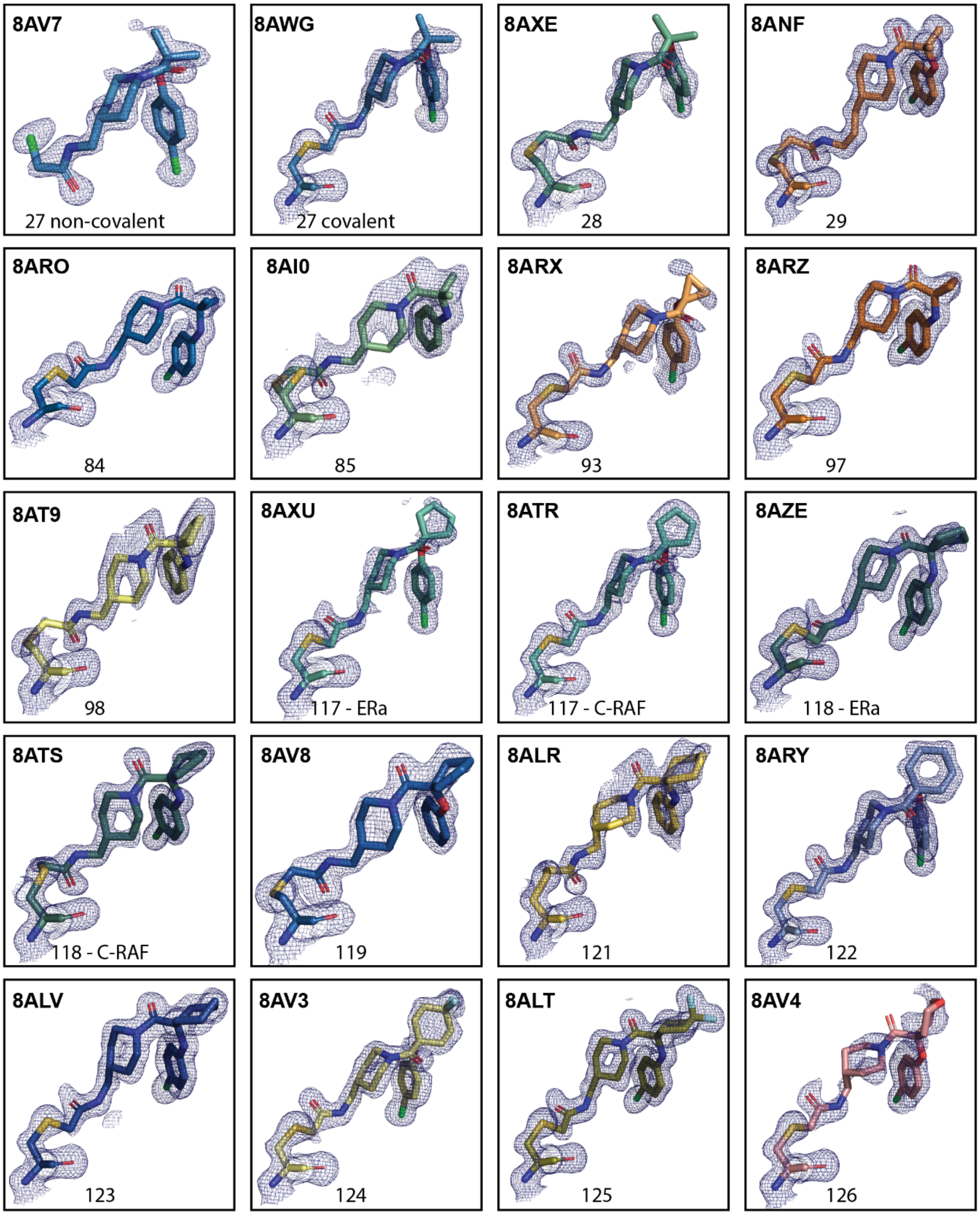

Figure S33. Densities and PDB IDs (top left) of C38 14-3-3σ tethered stabilizers (represented as sticks, compound number at bottom). 2Fo-Fc electron density maps (blue mesh) are contoured at 1σ. Crystallographic statistics are listed in tables S5-S11.

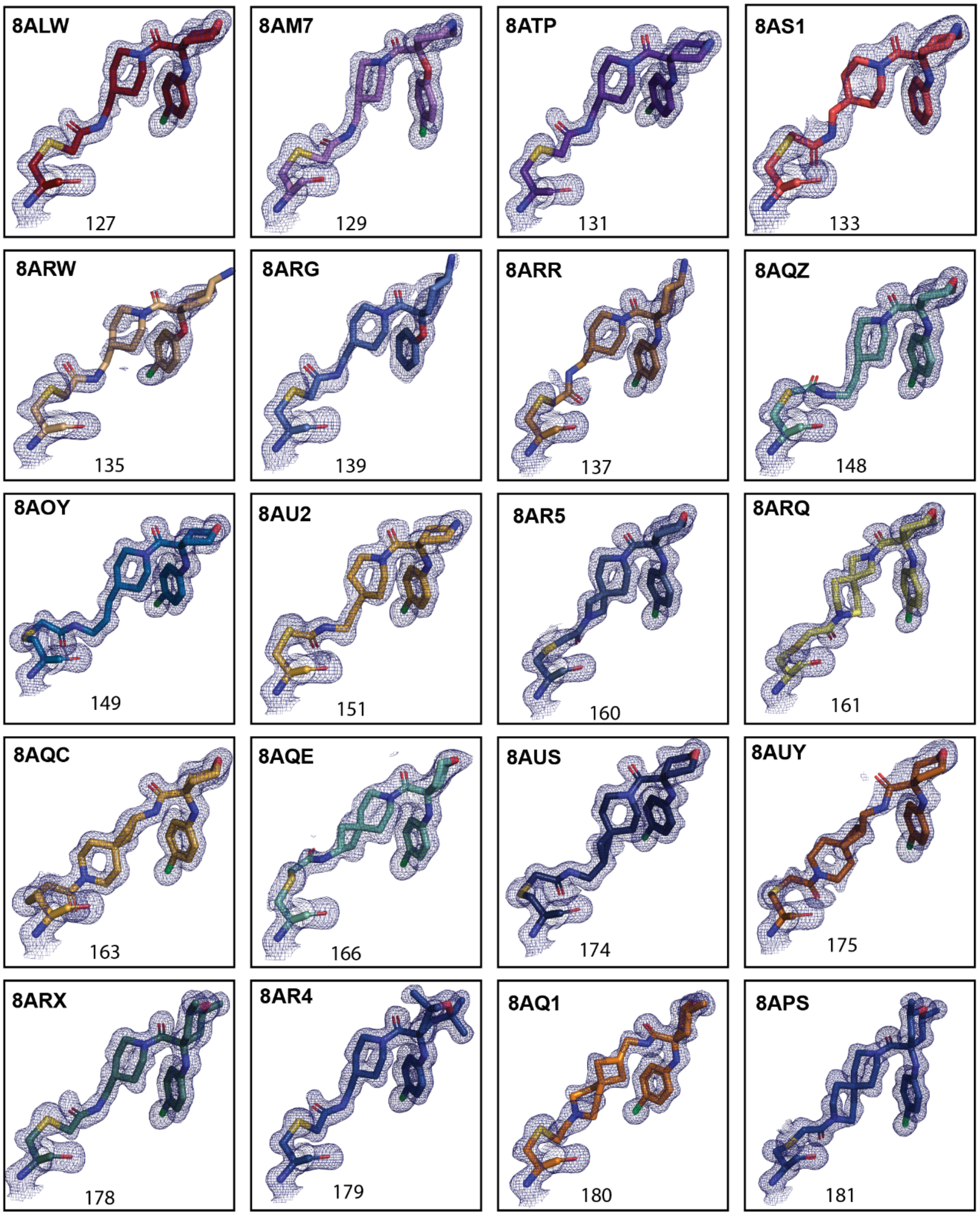

Figure S34. Densities and PDB IDs (top left) of C38 14-3-3σ tethered stabilizers (represented as sticks, compound number at bottom). 2Fo-Fc electron density maps (blue mesh) are contoured at 1σ. Crystallographic statistics are listed in tables S5-S11.

3. SUPPLEMENTARY TABLES

Table S1. Molecular structure of compounds used in the paper and assays performed.

No.	SMDC ID	Structure	MS [a]	FA – Compound titration [b]	Crystallography [c]	FA - Protein titration [d]	ITC
**(17)**	**1074199**	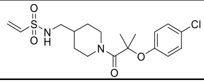	Fig S1	Fig S1	-	-	-
**(26)**	**1074360**	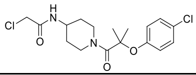	Fig S2	Fig 2	-	-	-
**(27)**	**1074202**	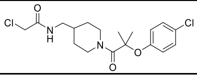	Fig S2	Fig 2	Table S5 (covalent & non-covalent)	-	-
**(28)**	**1074210**	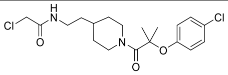	Fig S2	Fig 2	Table S5	-	-
**(29)**	**1074359**	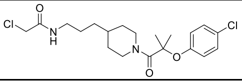	Fig S2	Fig 2	Table S5	-	-
**(37)**	**1074203**	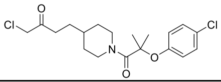	Fig S4	Fig S4	-	-	-
**(45)**	**1075294**	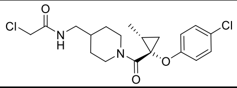	Fig S8	Fig S8	-	-	-
**(52)**	**1075308**	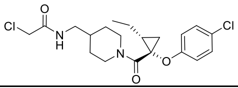	Fig S8	Fig S8	-	-	-
**(60)**	**1075343**	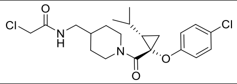	Fig S8	Fig S8	-	-	-
**(67)**	**1075314**	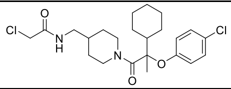	Fig S8	Fig S8	-	-	-
**(76)**	**1075321**	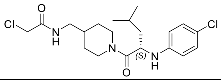	Fig S8	Fig S8	-	-	-
**(77)**	**1075322**	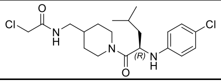	Fig S8	Fig S8	-	-	-
**(78)**	**1075319**	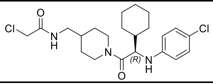	Fig S8	Fig S8	-	-	-
**(79)**	**1075320**	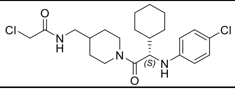	Fig S8	Fig S8	-	-	-
**(83)**	**1080270**	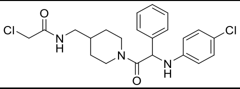	Fig S8	Fig S8	-	-	-
**(84)**	**1080291**	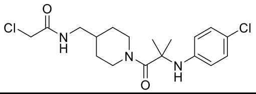	Fig S13 – S15	Fig S16 – S17	Table S5	-	-
**(85)**	**1080268**	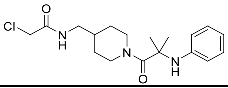	Fig S6	Fig S6	Table S6	-	-
**(93)**	**1074378**	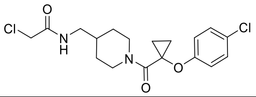	Fig S13 – S15	Fig S16 – S17	Table S6	-	-

**(97)**	**1076406**	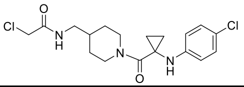	Fig S13 – S15	Fig S16 – S17	Table S6	-	-
**(98)**	**1080269**	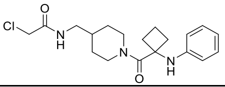	Table S2	Table S3	Table S6	-	-
**(117)**	**1075297**	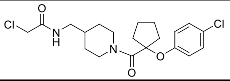	Fig S13 – S15	Fig S16 – S17	Table S6 (ERα)<br>Table S7 (C-RAF)	-	-
**(118)**	**1075306**	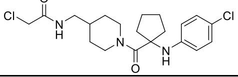	Fig S13 – S15	Fig S16 – S17	Table S7 (ERα & C-RAF)	-	-
**(119)**	**1075300**	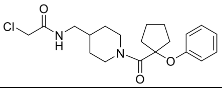	Fig S9	Fig S9	Table S7	-	-
**(120)**	**1080271**	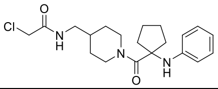	Fig S9	Fig S9	-	-	-
**(121)**	**1080272**	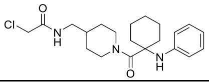	Table S2	Table S3	Table S7	-	-
**(122)**	**1080273**	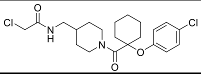	Fig S13 – S15	Fig S16 – S17	Table S7	-	-
**(123)**	**1076403**	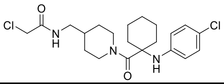	Fig S13 – S15	Fig S16 – S17	Table S8	-	-
**(124)**	**1075299**	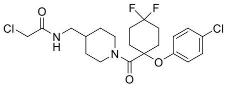	Fig S13 – S15	Fig S16 – S17	Table S8	-	-
**(125)**	**1075311**	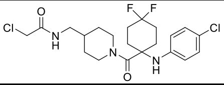	Fig S13 – S15	Fig S16 – S17	Table S8	-	-
**(126)**	**1075305**	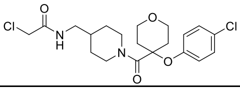	Fig S13 – S15	Fig S16 – S17	Table S8	-	-
**(127)**	**1075310**	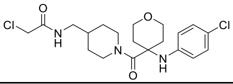	Fig S13 – S15	Fig S16 – S17	Table S8	Fig S18	-
**(129)**	**1076397**	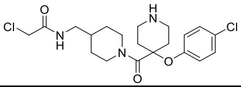	Fig S13 – S15	Fig S16 – S17	Table S8	-	-
**(131)**	**1075481**	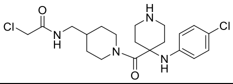	Fig S13 – S15	Fig S16 – S17	Table S9	Fig S18	-
**(133)**	**1076398**	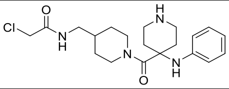	Fig S12	Fig S12	Table S9	-	-
**(135)**	**1076402**	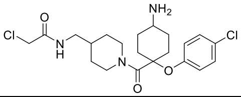	Fig S13 – S15	Fig S16 – S17	Table S9	-	-
**(137)**	**1076394**	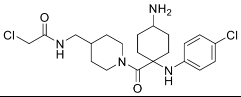	Fig S13 – S15	Fig S16 – S17	Table S9	-	-
**(139)**	**1076405**	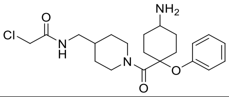	Fig S12	Fig S12	Table S9	-	-

**(141)**	**1076400**	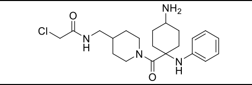	Fig S12	Fig S12	-	-	-
**(148)**	**1080267**	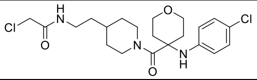	Fig S21	Fig S21	Table S9	-	-
**(149)**	**1075478**	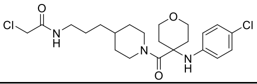	Fig S21	Fig S21	Table S10	Fig S18	-
**(151)**	**1080293**	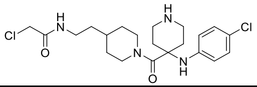	Fig S21	Fig S21	Table S10	-	-
**(153)**	**1076392**	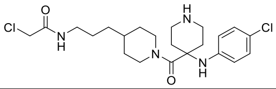	Fig S21	Fig S21	-	-	-
**(155)**	**1075479**	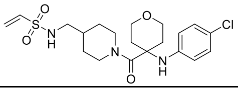	Fig S20	Fig S20	-	-	-
**(156)**	**1075351**	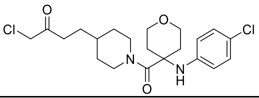	Fig S20	Fig S20			
**(160)**	**1080265**	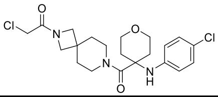	Fig S22	Fig S23	Table S10	-	-
**(161)**	**1080266**	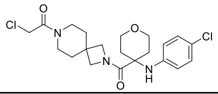	Fig S22	Fig S23	Table S10	-	-
**(163)**	**1080294**	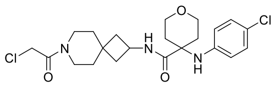	Fig S22	Fig S23	Table S10	Fig S25	-
**(166)**	**1080295**	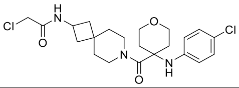	Fig S22	Fig S23	Table S10	Fig S25	-
**(170)**	**1080296**	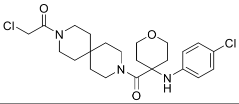	Fig S22	Fig S23	-	Fig S25	-
**(174)**	**1080297**	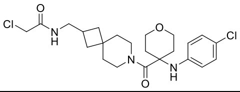	Fig S22	Fig S23	Table S11	-	-
**(175)**	**1080298**	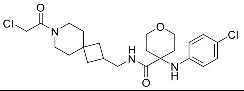	Fig S22	Fig S23	Table S11	-	-
**(178)**	**1080299**	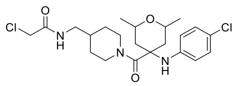	Fig S26	Fig S26	Table S11	Fig S27	-
**(179)**	**1080300**	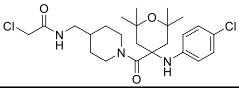	Fig S26	Fig S26	Table S11	-	-
**(180)**	**1083743**	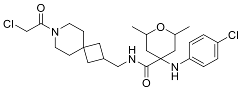	Fig S28	Fig S28	Table S11	Fig S29	-
**(181)**	**1083744**	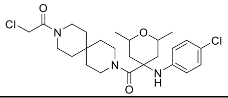	Fig S28	Fig S28	Table S11	Fig S29	Fig S32

a) Mass spec % labeled at 1:1 [protein] : [compound] in table S2, b) EC_{50} values from FA compound titrations in table S3, c) tables S5-S11 include crystallographic data for each compound, d) apparent Kd values and fold stabilization from FA protein titrations in table S4.

Table S2. Percentage (%) bound of compound (100 nM) to 14-3-3σ (100 nM) measured by mass spectrometry in the absence of peptide (apo), with ERα-peptide (2 μM) or with C-RAF peptide (18 μM) after 1, 8, 16 and 24 hours.

		APO				ERα				CRAF			
**No.**	**SMDC ID**	**1h**	**8h**	**16h**	**24h**	**1h**	**8h**	**16h**	**24h**	**1h**	**8h**	**24h**	**24h**
**(17)**	**1074199**	0	0	1.0	1.0	0.8	4.7	11.0	18.8	4.7	8.0	11.6	32.4
**(26)**	**1074360**	0	0	0	0	0	0	0	1.8	0.0	1.0	2.6	4.3
**(27)**	**1074202**	0.0	1.0	1.4	2.0	1.4	6.4	14.6	30.6	4.1	10.6	19.2	28.3
**(28)**	**1074210**	1.0	1.0	1.9	1.9	0.3	1.5	4.2	10.9	3.1	7.5	22.1	40.1
**(29)**	**1074359**	0	0	0	0	0.4	1.0	3.1	11.0	1.0	1.0	7.1	18.9
**(37)**	**1074203**	12.0	40.8	72.0	90.3	56.4	97.6	100	100	98.8	99.9	99.9	100
**(84)**	**1080291**	0	0	1.0	5.8	0.9	2.8	7.5	19.0	0.8	1.0	2.9	3.3
**(85)**	**1080268**	0	0	0	0	0.9	1.2	1.5	1.5	0.9	1.2	1.5	1.5
**(93)**	**1074378**	0	0	0	0	0	0.9	1.1	1.7	0.1	0.1	0.1	2.3
**(97)**	**1076406**	0.3	0.4	0.8	5.9	1.0	2.5	5.8	21.3	1.0	1.7	5.1	5.5
**(98)**	**1080269**	0	1.5	4.0	4.0	0	0	0	0	0	0	0	0
**(117)**	**1075297**	0	0.3	0.8	1.1	5.6	15.7	34.4	63.7	10.7	21.4	33.7	47.3
**(118)**	**1075306**	0	0	0.8	13.0	11.2	24.1	60.1	96.6	2.9	3.4	11.5	23.7
**(119)**	**1075300**	0	1.0	1.0	2.5	1.9	5.3	10.9	21.0	6.2	19.7	28.5	53.6
**(120)**	**1080271**	0	0	0	0	0	0	0	4.5	0	0	0	0
**(121)**	**1080272**	0	0	0	0	0	0	0	0	0	0	0	0
**(122)**	**1080273**	0	0	0.8	1.3	1.6	7.1	11.5	32.3	1.5	4.8	8.9	17.5
**(123)**	**1076403**	0.0	1.0	1.0	1.9	3.4	8.3	20.8	63.9	1.0	1.9	1.9	3.5
**(124)**	**1075299**	0	1.0	1.4	1.6	7.4	15.3	33.4	66.9	2.0	2.9	9.1	15.3
**(125)**	**1075311**	0.4	1.1	2.1	6.2	13.8	30.0	64.6	87.5	4.9	7.6	12.4	23.2
**(126)**	**1075305**	0	0	0	1.0	1.7	6.2	9.1	15.1	0.0	1.0	3.6	4.7
**(127)**	**1075310**	0	0	1.0	2.0	9.5	19.6	56.8	85.2	1.1	2.9	7.4	10.5
**(129)**	**1076397**	0.9	2.0	2.4	4.8	16.5	31.4	58.4	82.6	0.4	0.6	1.9	2.6
**(131)**	**1075481**	1.4	2.2	5.8	14.1	54.4	82.2	94.0	98.3	2.0	2.4	4.6	12.5
**(133)**	**1076398**	0.7	1.1	1.2	10.3	4.5	12.9	26.7	47.0	0	1.0	1.1	1.1
**(135)**	**1076402**	0.8	0.9	1.8	2.4	11.3	22.2	42.2	64.2	0.5	1.8	2.6	3.4
**(137)**	**1076394**	1.4	1.9	3.2	9.3	36.3	60.4	90.0	96.4	1.7	3.6	6.6	10.4
**(139)**	**1076405**	0	0	2.0	2.0	0	0	0	1.0	0	0	0	1.0
**(141)**	**1076400**	0	0	0.8	25.6	1.0	10.2	83.3	89.9	1.0	2.9	16.3	22.1
**(148)**	**1080267**	0	0	0	0	0	0	1.0	1.1	0	0	0	0
**(149)**	**1075478**	0.6	0.8	14.2	17.7	7.6	16.2	37.7	86.7	0	1.0	3.4	23.3
**(151)**	**1080293**	0	0	1.8	2.2	6.5	20.6	48.0	75.0	0	1.0	1.5	3.7
**(153)**	**1076392**	4.7	7.4	7.8	8.0	23.1	44.7	76.1	88.8	0	0	0	0
**(155)**	**1075479**	0	0.7	4.6	31.4	0.6	1.5	5.9	14.1	0	0	0	1.0
**(156)**	**1075351**	18.0	35.7	70.7	95.1	81.1	97.0	100	100	54.7	62.3	92.0	99.4
**(160)**	**1080265**	0	0	1.0	5.8	6.9	8.6	48.4	76.6	0.4	1.9	3.8	8.4
**(161)**	**1080266**	0	0	0.5	1.0	4.7	7.8	14.9	39.8	0	0.6	1.0	2.1
**(163)**	**1080294**	5.6	11.2	22.6	40.5	42.3	80.4	96.6	99.8	6.0	13.4	27.1	49.6
**(166)**	**1080295**	1.2	3.2	5.5	13.3	32.6	60.3	87.6	94.0	1.8	4.0	11.8	23.3
**(170)**	**1080296**	1.0	1.8	3.4	5.3	38.2	68.1	86.2	94.0	3.6	8.0	12.1	19.7
**(174)**	**1080297**	1.0	1.0	1.5	3.0	5.0	5.0	8.4	41.4	0.6	0.7	1.6	2.1
**(175)**	**1080298**	1.9	5.4	10.6	16.7	21.7	39.4	64.1	87.3	2.4	6.0	9.6	17.4
**(178)**	**1080299**	0.9	1.7	2.4	2.5	24.6	36.8	77.5	96.4	0	0.5	2.0	5.7
**(179)**	**1080300**	0	0	0.3	0.4	1.5	3.0	6.4	11.8	0	0	0	1.0
**(180)**	**1083743**	1.4	2.9	7.3	11.5	40.8	50.1	84.4	96.1	0	2.5	4.5	8.5
**(181)**	**1083744**	1.9	11.1	27.1	50.1	100	100	100	100	6.5	32.8	75.8	79.2

Table S3. EC_{50} values derived from FA compound titrations in the presence of 10 nM ERα-FAM labeled peptide, or 10 nM of C-RAF-FAM labeled peptide.

No.	SMDC ID	EC_{50} value ERα (μM)	EC_{50} value C-RAF (μM)
**(17)**	**1074199**	33 ± 1	-
**(26)**	**1074360**	>150	>150
**(27)**	**1074202**	19 ± 1	16 ± 4
**(28)**	**1074210**	24 ± 16	40 ± 1
**(29)**	**1074359**	9 ± 0.4	142 ± 40
**(37)**	**1074203**	44 ± 2	-
**(84)**	**1080291**	55 ± 2	>150
**(85)**	**1080268**	>150	-
**(93)**	**1074378**	68 ± 2	>150
**(97)**	**1076406**	15 ± 1	101 ± 15
**(98)**	**1080269**	>150	-
**(117)**	**1075297**	15 ± 2	32 ± 8
**(118)**	**1075306**	5 ± 0.4	>150
**(119)**	**1075300**	24 ± 2	18 ± 0.3
**(120)**	**1080271**	>150	>150
**(121)**	**1080272**	>150	-
**(122)**	**1080273**	55 ± 14	>150
**(123)**	**1076403**	8 ± 1	59 ± 4
**(124)**	**1075299**	18 ± 0.4	>150
**(125)**	**1075311**	12 ± 2	>150
**(126)**	**1075305**	9 ± 2	>150
**(127)**	**1075310**	2 ± 0.3	>150
**(129)**	**1076397**	30 ± 1	>150
**(131)**	**1075481**	2 ± 0.3	>150
**(133)**	**1076398**	115	-
**(135)**	**1076402**	37 ± 5	>150
**(137)**	**1076394**	8 ± 1	>150
**(139)**	**1076405**	>150	-
**(141)**	**1076400**	>150	-
**(148)**	**1080267**	92 ± 8	-
**(149)**	**1075478**	4 ± 0.4	-
**(151)**	**1080293**	16 ± 2	-
**(153)**	**1076392**	16 ± 1	-
**(155)**	**1075479**	>150	-
**(156)**	**1075351**	5 ± 0.3	-
**(160)**	**1080265**	16 ± 1	>150
**(161)**	**1080266**	22 ± 1	>150
**(163)**	**1080294**	4 ± 1	57 ± 5
**(166)**	**1080295**	6 ± 0.3	77 ± 5
**(170)**	**1080296**	8 ± 1	58 ± 11
**(174)**	**1080297**	97 ± 9	>150
**(175)**	**1080298**	15 ± 2	>150
**(178)**	**1080299**	12 ± 1	86 ± 11
**(179)**	**1080300**	23 ± 2	>150
**(180)**	**1083743**	10 ± 2	>150
**(181)**	**1083744**	1 ± 0.8	>150

Table S4: FA protein titrations

No.	SMDC ID	X Fold stabilization (100 µM compound)	Kd with DMSO reference (nM)	Apparent Kd (nM) (100 µM compound)
-	**FC-A**	166	2152	13
**(127)**	**1075310**	19	1493	77
**(131)**	**1075481**	30	1493	49
**(149)**	**1075478**	55	1493	27
**(163)**	**1080294**	18	1384	77
**(166)**	**1080295**	13	2077	160
**(170)**	**1080296**	12	1384	119
**(178)**	**1080299**	15	1384	94
**(180)**	**1083743**	7	1384	192
**(181)**	**1083744**	116	2089	18

For Tables S5-S11:

[a] Number in parentheses is for the highest resolution shell used in the refinement
[b] $CC_{1/2}$ = Pearson's intra-dataset correlation coefficient, as described by Karplus and Diederichs.[14]
[c] R_{merge} (= R_{sym}) = $\sum_h \sum_1 |I_{h1} - \langle I_h \rangle| / \sum_h \sum_1 \langle I_h \rangle$, where I_{h1} is the intensity of the 1th observation of reflection h and $\langle I_h \rangle$ is the average intensity of reflection h
[d] R_{meas} = $\sum_h \left| \sqrt{(n_h/(n_h - 1)} \sum_1 |I_{h1} - \langle I_h \rangle| \right| / \sum_h \sum_1 \langle I_h \rangle$ where n_h is the number of observations of reflection h
[e] Correlation of experimental intensities with intensities calculated from refined model, as described by Karplus and Diederichs.[14]

Table S5: XRD data collection and refinement statistics for 14-3-3σ / ERα structures

14-3-3σΔC / ERα	27 (1074202) (non- covalent)	27 (1074202) (covalent)	28 (1074210)	29 (1074359)	84 (1080291)
**PDB ID**	**8AV7**	**8AWG**	**8AXE**	**8ANF**	**8ARO**
**Data collection**					
**Collection source**	DLS io3	In-House Rigaku	ESRF ID30B	DESY PETRAIII P11	ESRF ID30B
**Collection date**	15/09/2019	09/12/2020	05/02/2021	05/05/2021	03/12/2021
**Beamtime**	mx19800-27		mx2268	11010503	mx2268
**Wavelength (Å)**	0.976254	1.54187	0.96863	1.033200	0.976254
**Resolution (Å)**	45.43 – 1.40 (1.42 – 1.40)	29.38 - 1.8 (1.864 - 1.8)	33.64 - 1.8 (1.864 - 1.8)	45.46 - 1.4 (1.45 - 1.4)	45.41 – 1.60 (1.63 – 1.60)
**Space group**	C 2 2 21	C 2 2 21	C 2 2 21	C 2 2 21	C 2 2 21
**Unit cell**	81.977 111.839 62.521	82.3741 112.65 62.7316	79.8955 111.262 61.9221	82.115 112.503 62.425	81.971 112.223 62.403
**Total reflections[a]**	728053 (25886)	162343 (6019)	87267 (4233)	765754 (34659)	108353 (5130)
**Unique reflections[a]**	56798 (2826)	27198 (2630)	24645 (2379)	57052 (5604)	36904 (1837)
**Redundancy[a]**	12.8 (9.2)	6.0 (4.6)	3.5 (3.5)	13.4 (12.6)	2.9 (2.8)
**Completeness (%)[a]**	99.9 (97.8)	99.12 (97.99)	94.85 (93.33)	99.84 (99.22)	96.7 (98.7)
**Average I/σ(I) [a]**	21.7 (3.1)	19.83 (3.65)	14.44 (5.68)	19.2 (2.7)	13.6 (5.3)
**Wilson B-factor ($Å^2$)**	13.39	9.93	17.00	12.49	10.91
**$CC_{1/2}$[a,b,c]**	1.000 (0.879)	0.999 (0.992)	0.972 (0.953)	0.999 (0.860)	0.995 (0.965)
**R_{merge}[a,c,e]**	0.057 (0.582)	0.039 (0.077)	0.073 (0.152)	0.075 (0.896)	0.049 (0.133)
**R_{meas}[a,d,e]**	0.059 (0.616)	0.047 (0.095)	0.097 (0.207)	0.078 (0.934)	0.060 (0.163)
**Refinement**					
**Reflections in set: Refinement / R-free**	56698 (5549) / 2834 (279)	34062 (735)/ 1750 (37)	25478 (2549)/ 1274 (121)	57046 (5605)/ 2902 (281)	36868 (3722) / 1892 (188)
**Non-H atoms: Overall / solvent**	2197 / 270	2262 / 337	2132 / 207	2334 / 306	2252 (326)
**R_{work} / R_{free} (%)**	0.1581 (0.1900) / 0.1828 (0.2463)	0.1730 (0.2026) /0.1938 (0.1977)	0.1995 (0.2327) /0.2295 (0.2575)	0.1508 (0.1780) / 0.1738 (0.1885)	0.1776 (0.1797) / 0.2027 (0.2325)
**RMSD from ideal geometry:**					
**Bond length (Å) / angles (°)**	0.011 / 0.134	0.015 / 1.55	0.010 / 1.27	0.011/1.40	0.197 / 3.09
**Average protein B-factor ($Å^2$)**	19.84	12.64	23.42	17.54	15.02
**Ramachandran: Favored / outlier (%)**	98.28 / 0.00	98.72 / 0.00	97.87 / 0.43	98.72 / 0.00	98.72 / 0.00
**Clashscore**	2.11	1.32	2.90	3.42	1.05

Table S6: XRD data collection and refinement statistics for 14-3-3σ / ERα structures

14-3-3σΔC / ERα	85 (1080268)	93 (1074378)	97 (1076406)	98 (1080269)	117 (1075297)
**PDB ID**	**8AI0**	**8ARX**	**8ARZ**	**8AT9**	**8AXU**
**Data collection**					
**Collection source**	DESY PETRAIII P11	DESY PETRAIII P11	DESY PETRAIII P11	DESY PETRAIII P11	ESRF ID30B
**Collection date**	26/08/2021	04/11/2021	09/08/2021	26/08/2021	05/02/2021
**Beamtime**	11012310	11011126	11010888	11012310	mx2268
**Wavelength (Å)**	1.033200	1.033200	1.033200	1.033200	0.96863
**Resolution (Å)**	45.42 – 1.60 (1.63 – 1.6)	45.46 – 1.40 (1.42 – 1.40)	45.47 – 1.50 (1.53 – 1.50)	45.40 – 1.40 (1.42 – 1.40)	45.55 - 1.6 (1.657 - 1.6)
**Space group**	C 2 2 21	C 2 2 21	C 2 2 21	C 2 2 21	C 2 2 21
**Unit cell**	81.801 112.230 62.504	81.979 112.338 62.521	82.049 112.494 62.499	81.886 112.364 62.404	82.2828 112.264 62.6288
**Total reflections**[a]	498947 (21567)	747519 (32829)	613855 (29560)	739162 (32652)	492193 (25125)
**Unique reflections**[a]	37776 (1786)	57003 (2724)	46626 (2297)	56855 (2772)	38639 (3845)
**Redundancy**[a]	13.2 (12.1)	13.1 (12.1)	13.2 (12.9)	13.0 (11.8)	12.7 (13.1)
**Completeness (%)**[a]	98.9 (95.8)	99.8 (96.9)	100.0 (100.0)	99.9 (98.8)	99.95 (100.00)
**Average I/σ(I)** [a]	40.6 (10.5)	49.7 (10.5)	38.2 (11.2)	13.8 (1.9)	29.65 (8.05)
**Wilson B-factor (Å²)**	15.72	12.55	14.06	14.60	14.15
**$CC_{1/2}$**[a,b,c]	1.000 (0.986)	1.000 (0.988)	1.000 (0.990)	0.998 (0.601)	0.999 (0.976)
**R_{merge}**[a,c,e]	0.038 (0.214)	0.029 (0.200)	0.041 (0.205)	0.106 (1.411)	0.055 (0.198)
**R_{meas}**[a,d,e]	0.040 (0.233)	0.030 (0.211)	0.043 (0.213)	0.110 (1.532)	0.060 (0.224)
**Refinement**					
**Reflections in set: Refinement / R-free**	37755 (3620) / 1924 (196)	56975 (5588) / 2899 (285)	46601 (4606) / 2339 (227)	56769 (5597) / 2888 (289)	38692 (3853) / 1985 (192)
**Non-H atoms: Overall / solvent**	2322 / 399	2239 / 313	2225 / 299	2174 / 250	2190 / 262
**R_{work} / R_{free} (%)**	0.1508 (0.1609) / 0.1742 (0.2093)	0.1504 (0.1461) / 0.1748 (0.1775)	0.1483 (0.1184) / 0.1762 (0.1705)	0.1611 (0.2418) / 0.1868 (0.2653)	0.1540 (0.1130) / 0.1843 (0.1643)
**RMSD from ideal geometry:**					
**Bond length (Å) / angles (°)**	0.010 / 1.23	0.015 / 1.57	0.013 / 1.40	0.016 / 1.67	0.011 / 1.32
**Average protein B-factor (Å²)**	20.19	17.38	18.75	21.74	18.16
**Ramachandran: Favored / outlier (%)**	98.30 / 0.00	98.72 / 0.00	98.72 / 0.00	98.30 / 0.00	98.30 / 0.00
**Clashscore**	4.75	2.64	2.37	2.64	3.94

Table S7: XRD data collection and refinement statistics for 14-3-3σ / ERα or – C-RAF structures

14-3-3σΔC	117 (1075297) / C-RAF	118 (1075306) / ERα	118 (1075306) / C-RAF	119 (1075300) / ERα	121 (1080272) / ERα	122 (1080273) / ERα
**PDB ID**	**8ATR**	**8AZE**	**8ATS**	**8AV8**	**8ALR**	**8ARY**
**Data collection**						
**Collection source**	ESRF ID23-2	ESRF ID30A (MASSIF-1)	DESY PETRAIII P11	ESRF ID30A (MASSIF-1)	DESY PETRAIII P11	DESY PETRAIII P11
**Collection date**	29/01/2022	26/11/2020	05/05/2021	05/02/2021	26/08/2021	26/08/2021
**Beamtime**	mx2268	mx2268	11010503	mx2268	11012310	11012310
**Wavelength (Å)**	0.873130	1.033200	1.033220	0.968626	1.033200	1.033200
**Resolution (Å)**	42.20 – 1.70 (1.73 – 1.70)	45.32 - 1.6 (1.657 - 1.6)	45.42 – 1.40 (1.42 – 1.40)	44.25 - 1.6 (1.657 - 1.6)	45.38 – 1.60 (1.63 – 1.60)	45.53 – 1.40 (1.43 – 1.40)
**Space group**	C 2 2 21	C 2 2 21	C 2 2 21	C 2 2 21	C 2 2 21	C 2 2 2 1
**Unit cell**	83.106 113.239 63.290	81.8381 111.827 62.3026	81.627 112.368 62.575	81.2195 111.851 59.8566	81.735 112.169 62.445	82.135 112.296 62.651
**Total reflections[a]**	63518 (3397)	177670 (8708)	109380 (5262)	454621 (22260)	514700 (21390)	744527 (35921)
**Unique reflections[a]**	33236 (1755)	37965 (3726)	56825 (2721)	36324 (3584)	38232 (1850)	57050 (28.62)
**Redundancy[a]**	1.9 (1.9)	4.7 (4.8)	1.9 (1.9)	12.5 (12.4)	13.5 (11.6)	13.1 (12.6)
**Completeness (%)[a]**	100.0 (100.0)	99.71 (99.57)	99.8 (96.9)	82.48 (18.96)	100.0 (99.7)	99.8 (97.8)
**Average I/σ(I) [a]**	13.1 (3.6)	17.6 (6.5)	25.2 (3.1)	6.4 (0.2)	33.5 (13.0)	39.4 (9.5)
**Wilson B-factor ($Å^2$)**	14.27	12.22	16.39	26.56	12.28	14.37
**$CC_{1/2}$[a,b,c]**	0.998 (0.930)	0.997 (0.968)	1.000 (0.889)	0.998 (0.289)	0.999 (0.991)	1.000 (0.986)
**R_{merge}[a,c,e]**	0.035 (0.162)	0.049 (0.166)	0.013 (0.201)	0.154 (3.988)	0.052 (0.160)	0.037 (0.236)
**R_{meas}[a,d,e]**	0.050 (0.229)	0.055 (0.189)	0.018 (0.285)	0.164 (4.267)	0.054 (0.168)	0.038 (0.246)
**Refinement**						
**Reflections in set: Refinement / R-free**	33194 (3287) / 1641 (165)	37970 (3730)/ 1851 (162)	56806 (5587) / 2900 (266)	25592 (2548) / 1338 (139)	56707 (5606) / 2889 (287)	46603 (4607) / 2339 (227)
**Non-H atoms: Overall / solvent**	2226 / 267	2217 / 290	2143 / 183	1844 / 96	2237 / 310	2247 / 322
**R_{work} / R_{free} (%)**	0.1956 (0.2102) / 0.2164 (0.2257)	0.1766 (0.1828) / 0.2028 (0.2729)	0.1643 (0.1977) / 0.1861 (0.2319)	0.2205 (0.3818) / 0.2623 (0.3593)	0.1585 (0.1489) / 0.1790 (0.1940)	0.1462 (0.1133) / 0.1717 (0.1635)
**RMSD from ideal geometry: Bond length (Å) / angles (°)**	0.009 / 1.10	0.010 / 1.36	0.012 / 1.28	0.013 / 1.43	0.012 / 1.44	0.013 / 1.48
**Average protein B-factor ($Å^2$)**	18.72	18.97	23.60	30.71	17.24	19.05
**Ramachandran: Favored / outlier (%)**	98.74 / 0.00	96.17 / 0.85	96.64 / 0.00	98.67 / 0.00	98.72 / 0.00	98.72 / 0.00
**Clashscore**	1.55	4.48	1.81	2.71	2.11	2.90

Table S8: XRD data collection and refinement statistics for 14-3-3σ / ERα structures

14-3-3σ ΔC / ERα	123 (1076403)	124 (1075299)	125 (1075311)	126 (1075305)	127 (1075310)	129 (1076397)
**PDB ID**	**8ALV**	**8AV3**	**8ALT**	**8AV4**	**8ALW**	**8AM7**
**Data collection**						
**Collection source**	DESY PETRAIII P11	ESRF ID30B	DESY PETRAIII P11	ESRF ID30A (MASSIF-1)	DESY PETRAIII P11	DESY PETRAIII P11
**Collection date**	09/08/2021	05/02/2021	04/11/2021	26/11/2020	04/11/2021	09/08/2021
**Beamtime**	11010888	mx2268	11011126	mx2268	11011126	11010888
**Wavelength (Å)**	1.03320	0.968626	1.033220	0.965459	1.033220	1.033220
**Resolution (Å)**	45.45 – 1.60 (1.63 – 1.60)	62.80 – 1.80 (1.84 – 1.80)	45.38 – 1.40 (1.43 – 1.40)	41.61 – 1.60 (1.63 – 1.60)	45.42 – 1.50 (1.53 – 1.50)	45.48 – 1.50 (1.53 – 1.50)
**Space group**	C 2 2 21	C 2 2 21	C 2 2 21	C 2 2 21	C 2 2 21	C 2 2 21
**Unit cell**	82.052 112.316 62.468	82.256 112.327 62.801	81.980 112.142 62.347	81.849 111.826 62.288	81.896 112.282 62.452	82.207 112.483 62.453
**Total reflections**[a]	514902 (25084)	344126 (19316)	748589 (35833)	121232 (5979)	618900 (29343)	620286 (28157)
**Unique reflections**[a]	38464 (1884)	27363 (1608)	56782 (2987)	37583 (1818)	46393 (2276)	46359 (2228)
**Redundancy**[a]	13.4 (13.3)	12.6 (12.0)	13.2 (12.0)	3.2 (3.3)	13.3 (12.9)	13.4 (12.6)
**Completeness (%)**[a]	100.0 (100.0)	100.0 (100.0)	100.0 (99.8)	98.9 (99.2)	99.9 (99.9)	99.9 (97.4)
**Average I/σ(I)** [a]	46.4 (16.1)	15.9 (5.1)	37.7 (13.1)	12.8 (4.1)	30.8 (8.8)	28.8 (7.5)
**Wilson B-factor (Å²)**	13.65	14.19	11.38	11.47	10.92	12.64
**$CC_{1/2}$**[a,b,c]	0.999 (0.995)	0.998 (0.935)	0.999 (0.994)	0.960 (0.944)	0.999 (0.987)	0.999 (0.979)
**R_{merge}**[a,c,e]	0.037 (0.121)	0.104 (0.465)	0.044 (0.144)	0.059 (0.213)	0.050 (0.198)	0.056 (0.293)
**R_{meas}**[a,d,e]	0.093 (0.126)	0.109 (0.485)	0.046 (0.150)	0.071 (0.254)	0.052 (0.207)	0.059 (0.293)
**Refinement**						
**Reflections in set: Refinement / R-free**	38440 (3809) / 1902 (193)	27332 (2682) / 1365 (123)	56759 (5639) / 2891 (287)	37519 (3703) / 1839 (162)	46366 (4588) / 2325 (225)	46321 (4542) / 2323 (220)
**Non-H atoms: Overall / solvent**	2327 / 406	2239 / 309	2259 / 328	2233 / 304	2254 / 326	2241 / 312
**R_{work} / R_{free} (%)**	0.1512 (0.1553) / 0.1755 (0.1922)	0.1778 (0.2624) / 0.2069 (0.2466)	0.1488 (0.1376) / 0.1690 (0.1703)	0.1769 (0.1895) / 0.2038 (0.2452)	0.1473 (0.1107) / 0.1739 (0.1452)	0.1463 (0.1287) / 0.1685 (0.1707)
**RMSD from ideal geometry: Bond length (Å) / angles (°)**	0.009 / 1.01	0.008 / 1.12	0.014 / 1.67	0.011 / 1.36	0.011 / 1.40	0.010 / 1.29
**Average protein B-factor (Å²)**	18.62	17.86	15.96	15.66	14.89	17.71
**Ramachandran: Favored / outlier (%)**	98.72 / 0.00	97.87 / 0.00	98.72 / 0.00	98.72 / 0.00	98.72 / 0.00	98.72 / 0.00
**Clashscore**	6.61	0.79	3.16	1.32	2.63	2.11

Table S9: XRD data collection and refinement statistics for 14-3-3σ / ERα structures

14-3-3σΔC / ERα	131 (1075481)	133 (1076398)	135 (1076402)	137 (1076394)	139 (1076405)	148(1080267)
**PDB ID**	**8ATP**	**8AS1**	**8ARW**	**8ARR**	**8ARG**	**8AQZ**
**Data collection**						
**Collection source**	DESY PETRAIII P11	DESY PETRAIII P11	DESY PETRAIII P11	ESRF ID23-1	DESY PETRAIII P11	DESY PETRAIII P11
**Collection date**	05/05/2021	09/08/2021	09/08/2021	26/06/2021	09/08/2021	26/08/2021
**Beamtime**	11010503	11010888	11010888	mx2268	11010888	11012310
**Wavelength (Å)**	1.033200	1.033200	1.033200	0.774899	1.033200	1.033200
**Resolution (Å)**	45.49 - 1.40 (1.45 - 1.40)	45.45 – 1.50 (1.53 – 1.50)	45.51 – 1.50 (1.53 – 1.50)	41.63 – 1.30 (1.32 – 1.30)	45.48 – 1.50 (1.53 – 1.50)	45.51 – 1.40 (1.42 – 1.40)
**Space group**	C 2 2 21	C 2 2 21	C 2 2 21	C 2 2 21	C 2 2 21	C 2 2 21
**Unit cell**	82.113 112.517 62.496	81.828 112.376 62.558	81.996 112.270 62.646	81.577 111.964 62.269	82.012 112.385 62.550	82.066 112.491 62.588
**Total reflections**[a]	763998 (35116)	612358 (28191)	608234 (27113)	892299 (42732)	619192 (28600)	714120 (32669)
**Unique reflections**[a]	56961 (5599)	46476 (2284)	45963 (2173)	70258 (3438)	46400 (2259)	56657 (2684)
**Redundancy**[a]	13.4 (12.8)	13.2 (12.3)	13.2 (12.5)	12.7 (12.4)	13.3 (12.7)	12.6 (12.2)
**Completeness (%)**[a]	99.79 (98.94)	99.9 (99.9)	99.3 (95.7)	100.0 (100.0)	99.8 (98.3)	99.2 (95.9)
**Average I/σ(I)** [a]	34.1 (10.8)	18.7 (2.5)	29.7 (7.4)	12.5 (2.0)	42.4 (18.4)	28.3 (10.6)
**Wilson B-factor (Å²)**	10.22	17.42	13.49	14.04	12.96	13.62
**$CC_{1/2}$**[a,b,c]	1.000 (0.990)	0.999 (0.799)	0.999 (0.978)	0.998 (0.652)	0.999 (0.996)	0.999 (0.988)
**R_{merge}**[a,c,e]	0.044 (0.174)	0.079 (1.107)	0.051 (0.297)	0.099 (1.902)	0.042 (0.118)	0.057 (0.189)
**R_{meas}**[a,d,e]	0.013 (0.050)	0.082 (1.155)	0.053 (0.309)	0.103 (1.989)	0.044 (0.123)	0.059 (0.198)
**Refinement**						
**Reflections in set: Refinement / R-free**	56959 (5599) / 2900 (283)	46434 (4583) / 2328 (230)	45817 (4435) / 2306 (217)	62699 (6152) / 3144 (306)	46374 (4570) / 2331 (229)	56641 (5480) / 2874 (272)
**Non-H atoms: Overall / solvent**	2286 / 357	2210 / 283	2220 / 290	2175 / 265	2231 / 302	2205 / 275
**R_{work} / R_{free} (%)**	0.1421 (0.1521)/ 0.1656 (0.1835)	0.1798 (0.2318) / 0.1972 (0.2628)	0.1519 (0.1386) / 0.1838 (0.1747)	0.1826 (0.2419) / 0.2052 (0.2420)	0.1446 (0.1078) / 0.1714 (0.1601)	0.1510 (0.1287) / 0.1673 (0.1666)
**RMSD from ideal geometry: Bond length (Å) / angles (°)**	0.016 / 1.66	0.013 / 1.47	0.015 / 1.56	0.012 / 1.50	0.015 / 1.57	0.014 / 1.54
**Average protein B-factor (Å²)**	15.12	22.01	20.64	18.42	17.98	18.38
**Ramachandran: Favored / outlier (%)**	98.72 / 0.00	98.72 / 0.00	98.72 / 0.00	98.70 / 0.00	98.72 / 0.00	98.72 / 0.00
**Clashscore**	2.63	2.11	2.37	2.12	3.42	1.85

Table S10: XRD data collection and refinement statistics for 14-3-3σ / ERα structures

14-3-3σΔC / ERα	149 (1075478)	151 (1080293)	160 (1080265)	161 (1080266)	163 (1080294)	166 (1080295)
**PDB ID**	**8AOY**	**8AU2**	**8AR5**	**8ARQ**	**8AQC**	**8AQE**
**Data collection**						
**Collection source**	DESY PETRAIII P11	ESRF ID30B	DESY PETRAIII P11	DESY PETRAIII P11	DESY PETRAIII P11	ESRF ID30B
**Collection date**	05/05/2021	03/12/2021	26/08/2021	26/08/2021	03/03/2022	03/12/2021
**Beamtime**	11010503	mx2268	11012310	11012310	11012787	mx2268
**Wavelength (Å)**	1.033200	0.976254	1.033200	1.033200	1.033200	0.97625
**Resolution (Å)**	41.86 - 1.401 (1.451 - 1.401)	45.44 – 1.60 (1.63 – 1.60)	41.75 – 1.40 (1.43 – 1.40)	45.50 – 1.40 (1.43 – 1.40)	45.38 – 1.50 (1.52 – 1.50)	34.07 – 1.60 (1.63 – 1.60)
**Space group**	C 2 2 21	C 2 2 21	C 2 2 21	C 2 2 21	C 2 2 21	C 2 2 21
**Unit cell**	82.377 112.627 62.565	82.037 112.418 62.433	82.140 112.354 62.410	82.355 112.308 62.458	81.876 112.352 62.344	81.941 112.391 62.426
**Total reflections**[a]	766089 (34053)	119560 (6016)	741909 (34510)	742530 (34088)	621442 (28047)	110864 (5461)
**Unique reflections**[a]	56782 (5546)	37059 (1853)	56728 (2804)	56969 (2790)	46273 (2218)	31647 (1677)
**Redundancy**[a]	13.5 (12.6)	3.2 (3.2)	13.1 (12.3)	13.0 (12.2)	13.4 (12.6)	3.5 (3.3)
**Completeness (%)**[a]	98.86 (98.02)	96.8 (98.7)	99.8 (99.2)	99.8 (98.8)	99.7 (97.4)	83.0 (86.4)
**Average I/σ(I)** [a]	37.2 (12.5)	17.0 (9.3)	41.6 (17.1)	44.7 (10.2)	23.6 (5.2)	14.9 (8.9)
**Wilson B-factor ($Å^2$)**	10.24	10.58	9.96	13.18	13.91	11.55
**$CC_{1/2}$**[a,b,c]	0.998 (0.994)	0.990 (0.974)	0.999 (0.996)	1.000 (0.988)	0.999 (0.957)	0.989 (0.975)
**R_{merge}**[a,c,e]	0.041 (0.120)	0.048 (0.096)	0.043 (0.112)	0.032 (0.208)	0.063 (0.455)	0.062 (0.099)
**R_{meas}**[a,d,e]	0.042 (0.125)	0.057 (0.114)	0.045 (0.117)	0.034 (0.217)	0.065 (0.474)	0.073 (0.118)
**Refinement**						
**Reflections in set: Refinement / R-free**	56776 (5546) / 2892 (277)	36971 (3740) / 1892 (188)	56704 (5627) / 2890 (289)	56944 (5633) / 2902 (285)	46179 (4561) / 2271 (209)	31600 (3249) / 1631 (156)
**Non-H atoms: Overall / solvent**	2284 / 353	2263 / 333	2274 / 344	2237 / 307	2209 / 280	2215 / 285
**R_{work} / R_{free} (%)**	0.1408(0.1489) / 0.1646 (0.1747)	0.1803 (0.1792) / 0.2072 (0.2295)	0.1460 (0.1136) / 0.1694 (0.1584)	0.1517 (0.1226) / 0.1772 (0.1745)	0.1581 (0.1500) / 0.1856 (0.2047)	0.2071 (0.2051) / 0.2436 (0.2529)
**RMSD from ideal geometry: Bond length (Å) / angles (°)**	0.013 / 1.59	0.055 / 2.29	0.014 / 1.55	0.016 / 1.69	0.010 / 1.31	0.009 / 1.25
**Average protein B-factor ($Å^2$)**	15.06	14.20	14.45	17.21	18.35	15.12
**Ramachandran: Favored / outlier (%)**	98.72 / 0.00	98.72 / 0.00	98.72 / 0.00	98.72 / 0.00	98.72 / 0.00	98.30 / 0.00
**Clashscore**	2.10	3.16	2.90	0.53	2.64	2.11

Table S11: XRD data collection and refinement statistics for 14-3-3σ / ERα structures

14-3-3σΔC / ERα	174 (1080297)	175 (1080298)	178 (1080299)	179 (1080300)	180 (1083743)	181(1083744)
**PDB ID**	**8AUS**	**8AUY**	**8ARX**	**8AR4**	**8AQ1**	**8APS**
**Data collection**						
**Collection source**	ESRF ID30B	ESRF ID30B	ESRF ID30B	ESRF ID30B	ESRF ID23-2	ESRF ID23-2
**Collection date**	03/12/2021	03/12/2021	03/12/2021	03/12/2021	29/01/2022	29/01/2022
**Beamtime**	mx2268	mx2268	mx2268	mx2268	mx2268	mx2268
**Wavelength (Å)**	0.976254	0.97625	0.976254	0.976254	0.873128	0.873128
**Resolution (Å)**	45.44 – 1.40 (1.42 – 1.40)	66.23 – 1.50 (1.53 – 1.50)	45.48 – 1.40 (1.42 – 1.40)	62.28 – 1.50 (1.53 – 1.50)	41.84 – 1.40 (1.42 – 1.40)	41.90 – 1.20 (1.22 – 1.20)
**Space group**	C 2 2 21	C 2 2 21	C 2 2 21	C 2 2 21	C 2 2 21	C 2 2 21
**Unit cell**	81.879 112.428 62.485	81.971 112.406 62.344	82.110 112.675 62.456	82.032 111.718 62.281	82.249 112.428 62.645	82.565 112.833 62.581
**Total reflections[a]**	138758 (6418)	137694 (6715)	180258 (8639)	143014 (7118)	736924 (35817)	1047550 (27541)
**Unique reflections[a]**	54973 (2686)	44445 (2208)	54927 (2749)	39200 (2016)	57400 (2810)	90357 (4159)
**Redundancy[a]**	2.5 (2.4)	3.1 (3.0)	3.3 (3.1)	3.6 (3.5)	12.8 (12.7)	11.6 (6.6)
**Completeness (%)[a]**	96.8 (96.1)	96.3 (97.3)	96.5 (98.4)	86.2 (90.1)	100.0 (99.8)	99.0 (93.2)
**Average I/σ(I) [a]**	8.5 (2.6)	9.7 (3.0)	16.3 (6.5)	18.9 (6.4)	25.1 (6.2)	22.2 (4.4)
**Wilson B-factor (Å²)**	11.37	12.21	9.20	12.59	9.34	9.02
**$CC_{1/2}$[a,b,c]**	0.978 (0.837)	0.928 (0.404)	0.994 (0.967)	0.992 (0.950)	0.991 (0.967)	0.653 (0.945)
**R_{merge}[a,c,e]**	0.074 (0.279)	0.074 (0.332)	0.051 (0.141)	0.041 (0.137)	0.061 (0.386)	0.058 (0.331)
**R_{meas}[a,d,e]**	0.095 (0.351)	0.091 (0.404)	0.060 (0.169)	0.048 (0.161)	0.064 (0.402)	0.061 (0.358)
**Refinement**						
**Reflections in set: Refinement / R-free**	54906 (5501) / 2780 (279)	44362 (4391) / 2249 (206)	54873 (5534) / 2771 (275)	60887 (6306) / 3109 (331)	57318 (5645) / 2916 (283)	90268 (8432) / 4592 (448)
**Non-H atoms: Overall / solvent**	2241 / 310	2263 / 331	2274 / 343	2246 / 313	2289 / 350	2290 / 344
**R_{work} / R_{free} (%)**	0.1926 (0.2175) / 0.2166 (0.2321)	0.2050 (0.2294) / 0.2354 (0.2294)	0.1474 (0.1313) / 0.1701 (0.1712)	0.2035 (0.2449) / 0.2282 (0.2416)	0.1705 (0.2042) / 0.1921 (0.2320)	0.1462 (0.1515) / 0.1665 (0.1870)
**RMSD from ideal geometry: Bond length (Å) / angles (°)**	0.012 / 1.52	0.008 / 1.15	0.014 / 1.56	0.012 / 1.45	0.014 / 1.59	0.010 / 1.37
**Average protein B-factor (Å²)**	14.74	16.65	14.37	16.73	13.25	13.67
**Ramachandran: Favored / outlier (%)**	98.30 / 0.00	98.72 / 0.00	98.72 / 0.00	98.72 / 0.00	98.72 / 0.00	98.72 / 0.00
**Clashscore**	3.16	1.58	3.69	3.42	1.05	2.35

4. SYNTHETIC PROCEDURES

GENERAL REMARKS

All solvents and reagents were commercially available and used without purification, unless otherwise stated. Deuterated solvents were obtained from Cambridge Isotope Laboratories. Reaction progress was monitored by analytical thin-layer chromatography (TLC, pre-coated silica gel 60 F254 plates, Merck) using ultraviolet (UV) light (254 and 365 nm). Analytical liquid chromatography coupled with mass spectrometry (LC-MS) was performed on a C4 Jupiter SuC4300A 150 x 2.0 mm column (using a 15 min. gradient of 5% to 100% acetonitrile in H_2O with 0.1% formic acid), connected to a ThermoFischer LCQ Fleet Ion Trap Mass Spectrometer. Preparative high-pressure column chromatography was performed on a Waters 150 system using SRC C18 cartridges (20 min method). NMR data were recorded on a Bruker Advance-III 400 MHz equipped with a BBFO probe from Bruker (400 MHz for ^{1}H-NMR and 100 MHz for ^{13}C-NMR). Chemical shifts were reported in parts per million (ppm) referenced to an internal standard (*d*-chloroform; 7.26 ppm for ^{1}H-NMR and 77 ppm for ^{13}C-NMR), relative to tetramethylsilane (TMS).

Scheme 1. Synthetic route for *gem*-dimethyl derivatives with irreversible covalent warheads [a]

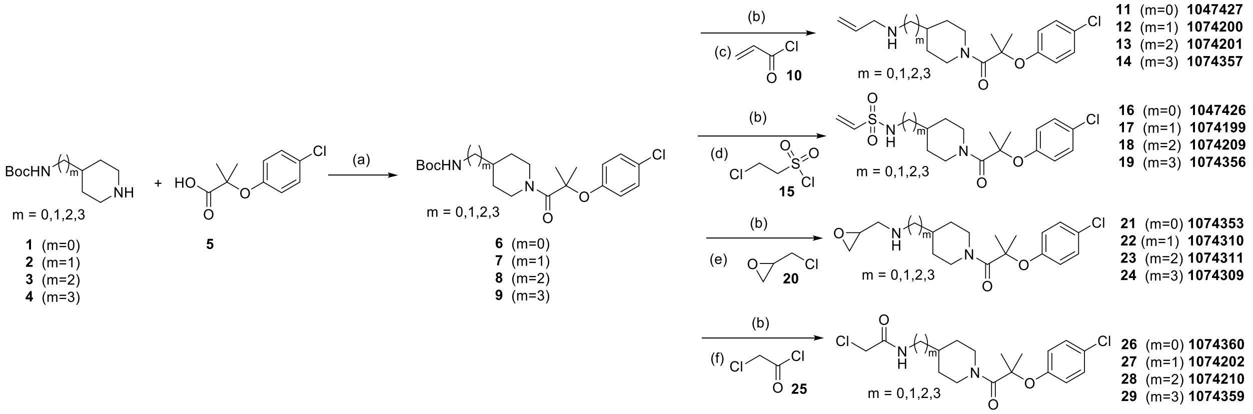

[a] Reagents and conditions: (a) HATU, DIPEA, DMF, rt, overnight; (b), 4N HCl/dioxane, rt, 3h; (c) acrolyl chloride **10**, Et_3N, DCM, 0°C to rt, 2h; (d) 2-chloroethanesulfonyl chloride **15**, Et_3N, DCM, 0°C to rt, 2h; (e) 2-(chloromethyl)oxirane **20**, K_2CO_3, DMF, 60°C, overnight; (f) chloroacetyl chloride **25**, DIPEA, DCM, 0°C to rt, 2h.

General procedures

Procedure A. At 0°C 2-(4-chlorophenoxy)isobutyric acid **5** (1 equiv) and HATU (1.2 equiv) were dissolved in dry DMF (2 ml for 1 mmol reaction scale). The appropriate amine (1.2 equiv) was dissolved in 1 ml DMF, and DIPEA (3 equiv) was added. The solution of the amine was added to the reaction mixture under stirring. Stirring at 0 °C for 30 min, then rt overnight. The reaction mixture was diluted with sat. NH_4Cl (10 ml) and extracted with ethyl acetate (3x10ml). The combined organic phases were washed with Brine, dried over MgSO4, filtered and concentrated under reduced pressure. The crude was purified with flash column chromatography (Biotage, hexane - EtOAc, 0-100% EtOAc in hexane). The product was confirmed with LCMS and used directly in the next step.

Procedure B. The boc-protected amine was dissolved in 3ml HCl/dioxane (4N). Stirring rt for 3h. The solvent was removed under reduced pressure and the obtained HCl salt was used directly in the next step.

Procedure C. The HCl salt (1 equiv) was suspended in 2 ml dry DCM. At 0°C, Et_3N (4 equiv) was added. After 10 min, acrolyl chloride **10** was added slowly (1.2 equiv). Stirring at 0°C for 30 min and then at rt for 2h. The reaction mixture was quenched with sat. $NaHCO_3$ (10 ml) and extracted with DCM (3 x 10 ml). The combined organic phases were dried over $MgSO_4$, filtered and concentrated under reduced pressure. The obtained oil was purified with HPLC (column C18, H_2O – CH_3CN + 0.05% formic acid, gradient 30-100% CH_3CN in H_2O, 20 min total).

N-(1-(2-(4-chlorophenoxy)-2-methylpropanoyl)piperidin-4-yl)acrylamide **(11) 1047427**

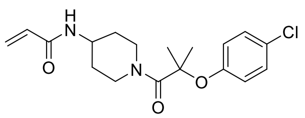

Obtained using procedure C on 0.2 mmol scale, colorless oil, 25 mg, 35% yield. ^{1}H NMR (400 MHz, $CDCl_3$) δ: 7.14 – 7.11 (m, 2H), 6.73 – 6.69 (m, 2H), 6.23 – 6.19 (m, 1H), 6.08 – 6.01 (m, 1H), 5.59 (dd, *J* = 10.2, 1.5 Hz, 1H), 4.63 – 4.60 (m, 2H), 3.15 – 3.10 (m, 1H), 3.04 – 2.99 (m, 1H), 2.87 – 2.81 (m, 1H), 2.53 – 2.47 (m, 1H), 1.73 – 1.66 (m, 2H), 1.56 (s, 6H), 1.05 – 0.96 (m, 1H), 0.75 – 0.67 (m, 1H). ^{13}C NMR (100 MHz, $CDCl_3$) δ: 171.1, 165.7, 154.0, 130.6, 129.1, 126.5, 126.1, 118.2, 81.0, 45.4, 44.4, 43.1, 36.2, 30.0, 29.5, 26.0, 25.8. LCMS (ESI): m/z calcd for $C_{18}H_{23}ClN_2O_3$; found $[M+H]^+$ 351.80

N-((1-(2-(4-chlorophenoxy)-2-methylpropanoyl)piperidin-4-yl)methyl)acrylamide **(12) 1074200**

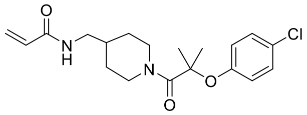

Obtained using procedure C on 0.2 mmol scale, colorless oil, 20 mg, 27% yield. ^{1}H NMR (400 MHz, $CDCl_3$) δ: 7.16 (d, *J* = 9.0 Hz, 2H), 6.74 (d, *J* = 9.0 Hz, 2H), 6.29 – 6.17 (m, 1H), 6.09 – 5.96 (m, 1H), 5. 92 – 5.76 (m, 1H), 5.68 – 5.51 (m, 1H), 4.78 –4.52 (m, 2H), 3.25 – 2.76 (m, 3H), 2.64 – 2.41 (m, 1H), 1.72 – 1.70 (m, 3H), 1.56 (s, 6H), 1.05 – 0.96 (m, 1H), 0.75 – 0.67 (m, 1H). LCMS (ESI): m/z calcd for $C_{19}H_{25}ClN_2O_3$; found $[M+H]^+$ 365.18

N-(2-(1-(2-(4-chlorophenoxy)-2-methylpropanoyl)piperidin-4-yl)ethyl)acrylamide **(13) 1074201**

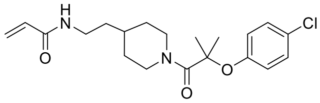

Obtained using procedure C on 0.2 mmol scale, colorless oil, 19 mg, 25% yield. ^{1}H NMR (400 MHz, $CDCl_3$) δ: 7.17 (d, *J* = 9.0 Hz, 2H), 6.75 (d, *J* = 9.0 Hz, 2H), 6.32 – 6.17 (m, 1H), 6.10 – 5.93 (m, 1H), 5.70 – 5.50 (m, 2H), 4.75 – 4.44 (m, 2H), 3.42 – 3.11 (m, 2H), 2.82 – 2.80 (m, 1H), 2.64 – 2.42 (m, 1H), 1.64 – 1.62 (m, 3H), 1.61 (s, 6H), 1. 41 – 1.30 (m, 2H), 1.05 – 0.96 (m, 1H), 0.75 – 0.67 (m, 1H) . LCMS (ESI): m/z calcd for $C_{20}H_{27}ClN_2O_3$; found $[M+Na]^+$ 399.00

N-(3-(1-(2-(4-chlorophenoxy)-2-methylpropanoyl)piperidin-4-yl)propyl)acrylamide **(14) 1074357**

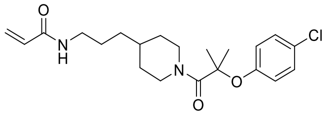

Obtained using procedure C on 0.2 mmol scale, colorless oil, 20 mg, 25% yield. ^{1}H NMR (400 MHz, $CDCl_3$) δ: 7.17 (d, *J* = 9.0 Hz, 2H), 6.75 (d, *J* = 9.0 Hz, 2H), 6.30 – 6.20 (m, 1H), 6.10 – 5.96 (m, 1H), 5.71 – 5.50 (m, 2H), 4.76 – 4.44 (m, 2H), 3.33 – 3.18 (m, 2H), 2.91 – 2.73 (m, 1H), 2.62 – 2.34 (m, 1H), 1.62 (s, 6H), 1.54 – 1.35 (m, 5H), 1.22 – 1.07 (m, 2H), 1.03 – 0.89 (m, 1H), 0.76 – 0.56 (m, 1H). ^{13}C NMR (100 MHz, $CDCl_3$) δ: 171.0, 165.5, 154.2, 130.8, 129.3, 126.3, 118.2, 117.0, 81.1, 45.8, 43.6, 39.6, 35.6, 33.3, 31.8, 26.5, 25.9
LCMS (ESI): m/z calcd for $C_{21}H_{29}ClN_2O_3$; found $[M+H]^+$ 393.24

Procedure D. The HCl salt (1 equiv) was suspended in 2 ml dry DCM. At 0°C, Et_3N (4 equiv) was added. After 10 min, 2-chloroethanesulfonyl chloride **15** was added slowly (1.2 equiv). Stirring at 0°C for 30min and then at rt for 2h. The reaction mixture was quenched with sat. $NaHCO_3$ (10 ml) and extracted with DCM (3 x 10 ml). The combined organic phases were dried over $MgSO_4$, filtered and concentrated under reduced pressure. The obtained oil was purified with HPLC (column C18, H_2O – CH_3CN + 0.05% formic acid, gradient 30-100% CH_3CN in H_2O, 20 min total).

N-(1-(2-(4-chlorophenoxy)-2-methylpropanoyl)piperidin-4-yl)ethenesulfonamide **(16) 1047426**

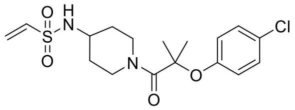

Obtained using procedure D on 0.1 mmol scale, colorless oil, 21 mg, 54% yield. ^{1}H NMR (400 MHz, $CDCl_3$) δ: 7.16 (d, *J* = 9.0 Hz, 2H), 6.73 (d, *J* = 9.0 Hz, 2H), 6.43 (dd, *J* = 16.5, 9.9 Hz, 1H), 6.18 (d, *J* = 16.5 Hz, 1H), 5.89 (d, *J* = 9.9 Hz, 1H), 4.87 – 4.84 (m, 1H), 4.63 (d, *J* = 13.3 Hz, 2H), 2.86 – 2.82 (m, 1H), 2.73 (t, *J* = 6.3 Hz, 2H), 2.52 – 2.50 (m, 1H), 1.75 – 1.72 (m, 1H), 1.66 – 1.63 (m, 1H), 1.58 (s, 6H), 1.00 – 0.95 (m, 1H), 0.72 – 0.64 (m, 1H). ^{13}C NMR (100 MHz, $CDCl_3$) δ: 171.2, 154.0, 135.7, 129.2, 126.6, 126.2, 118.2, 81.0, 48.0, 45.2, 42.3, 36.4, 29.9, 29.3, 25.9. LCMS (ESI): m/z calcd for $C_{17}H_{23}ClN_2O_4S$; found [M+Na]$^+$ 409.81

N-((1-(2-(4-chlorophenoxy)-2-methylpropanoyl)piperidin-4-yl)methyl)ethenesulfonamide **(17) 1074199**

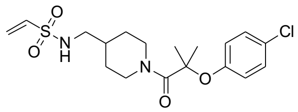

Obtained using procedure D on 0.2 mmol scale, white solid, 15.2 mg, 20% yield. HPLC retention time 12.5 min. ^{1}H NMR (400 MHz, $CDCl_3$) δ: 7.17 (d, *J* = 8.9 Hz, 2H), 6.74 (d, *J* = 8.9 Hz, 2H), 6.45 (dd, *J* = 16.5, 9.9 Hz, 1H), 6.20 (d, *J* = 16.5 Hz, 1H), 5.92 (d, *J* = 9.9 Hz, 1H), 4.67 (d, *J* = 13.3 Hz, 2H), 4.52 (t, *J* = 6.3 Hz, 1H), 2.87 (t, *J* = 12.6 Hz, 1H), 2.76 (t, *J* = 6.3 Hz, 2H), 2.53 (t, *J* = 12.3 Hz, 1H), 1.77 – 1.75 (m, 2H), 1.68 – 1.66 (m, 1H), 1.61 (s, 6H), 1.04 – 0.96 (m, 1H), 0.72 – 0.64 (m, 1H). ^{13}C NMR (100 MHz, $CDCl_3$) δ: 171.2, 154.1, 135.7, 129.2, 126.8, 126.3, 118.2, 81.0, 48.1, 45.3, 43.0, 36.5, 29.9, 29.4, 26.0. LCMS (ESI): m/z calcd for $C_{18}H_{25}ClN_2O_4S$; found [M+H]$^+$ 401.25, [M+Na]$^+$ 423.15

N-(2-(1-(2-(4-chlorophenoxy)-2-methylpropanoyl)piperidin-4-yl)ethyl)ethenesulfonamide **(18) 1074209**

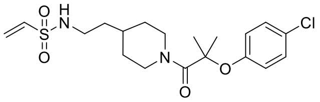

Obtained using procedure D on 0.2 mmol scale, colorless oil, 21.0 mg, 25% yield. ^{1}H NMR (400 MHz, $CDCl_3$) δ: 7.18 (d, *J* = 9.0 Hz, 2H), 6.76 (d, *J* = 9.0 Hz, 2H), 6.45 (dd, *J* = 16.5, 9.9 Hz, 1H), 6.20 (d, *J* = 16.5 Hz, 1H), 5.92 (d, *J* = 9.9 Hz, 1H), 4.77 – 4.51 (m, 2H), 4.29 – 4.17 (m, 1H), 3.07 – 2.93 (m, 2H), 3.01 – 2.79 (m, 3H), 2.65 – 2.44 (m, 2H), 1.62 (s, 6H), 1.42 – 1.35 (m, 2H), 1.09 – 0.94 (m, 1H), 0.75 – 0.59 (m, 1H). ^{13}C NMR (100 MHz, $CDCl_3$) δ: 171.0, 154.1, 129.2, 126.2, 118.2, 81.0, 45.6, 43.4, 42.6, 37.2, 35.7, 33.6, 32.2, 31.7, 25.9. LCMS (ESI): m/z calcd for $C_{19}H_{27}ClN_2O_4S$; found [M+Na]$^+$ 437.80

N-(3-(1-(2-(4-chlorophenoxy)-2-methylpropanoyl)piperidin-4-yl)propyl)ethenesulfonamide **(19) 1074356**

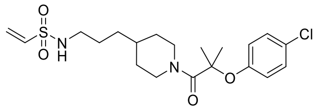

Obtained using procedure D on 0.2 mmol scale, colorless oil, 22.0 mg, 25% yield. ^{1}H NMR (400 MHz, $CDCl_3$) δ: 7.18 (d, *J* = 9.0 Hz, 2H), 6.76 (d, *J* = 9.0 Hz, 2H), 6.45 (dd, *J* = 16.5, 9.9 Hz, 1H), 6.20 (d, *J* = 16.5 Hz, 1H), 5.92 (d, *J* = 9.9 Hz, 1H), 4.87 – 4.343 (m, 2H), 4.36 – 3.96 (m, 1H), 3.05 – 2.75 (m, 3H), 2.58 – 2.40 (m, 1H), 1.64 – 1.63 (m, 1H), 1.62 (s, 6H), 1.54 – 1.31 (m, 4H), 1.24 -1.13 (m, 2H), 1.07 – 0.91 (m, 1H), 0.76 – 0.50 (m, 1H). ^{13}C NMR (100 MHz, $CDCl_3$) δ: 171.4, 154.5, 129.5, 127.0, 126.5, 118.6, 81.4, 46.1, 43.8, 43.4, 35.9, 33.3, 32.7, 32.1, 27.3, 26.4, 26.3. LCMS (ESI): m/z calcd for $C_{20}H_{29}ClN_2O_4S$; found [M+H]$^+$ 429.06

Procedure E. The HCl salt (1 equiv) was dissolved in 2 ml dry DMF. At 0°C, K_2CO_3 (3 equiv) was added. After 10 min, 2-(chloromethyl)oxirane **20**, was added slowly (1.2 equiv). Stirring at 0°C for 30 min and then heating at 60°C overnight. The reaction mixture was quenched with sat. $NaHCO_3$ (10 ml), diluted with EtOAc (10ml) and extracted (x3). The combined organic phases were dried over $MgSO_4$, filtered and concentrated under reduced pressure. The obtained oil was purified with flash column chromatography (Biotage, hexane - EtOAc, 0-100% EtOAc in hexane).

2-(4-chlorophenoxy)-2-methyl-1-(4-((oxiran-2-ylmethyl)amino)piperidin-1-yl)propan-1-one **(21) 1074353**

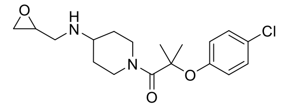

Obtained using procedure E on 0.2 mmol scale, colorless oil, 8.0 mg, 10% yield. ^{1}H NMR (400 MHz, $CDCl_3$) δ: 7.16 (d, *J* = 9.0 Hz, 2H), 6.74 (d, *J* = 9.0 Hz, 2H), 4.53 (t, *J* = 11.7 Hz, 2H), 3.00 (t, *J* = 12.2 Hz, 1H), 2.82 (ddd, *J* = 14.1, 10.0, 3.9 Hz, 1H), 2.72 (t, *J* = 12.2 Hz, 1H), 1.83 – 1.80 (m, 1H), 1.70 – 1.68 (m, 7H), 1.61 (s, 6H), 1.18 – 1.14 (m, 1H), 0.92 – 0.86 (m, 1H). ^{13}C NMR (100 MHz, $CDCl_3$) δ: 171.2, 154.1, 129.3, 126.3, 118.2, 81.0, 53.4, 48.5, 44.1, 42.1, 35.5, 35.1, 26.0. LCMS (ESI): m/z calcd for $C_{18}H_{25}ClN_2O_3$; found $[M+H]^+$ 353.16

2-(4-chlorophenoxy)-2-methyl-1-(4-(((oxiran-2-ylmethyl)amino)methyl)piperidin-1-yl)propan-1-one **(22) 1074310**

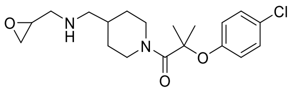

Obtained using procedure E on 0.4 mmol scale, colorless oil, 48.0 mg, 32% yield. ^{1}H NMR (400 MHz, $CDCl_3$) δ: 7.16 (d, *J* = 9.0 Hz, 2H), 6.74 (d, *J* = 9.0 Hz, 2H), 4.71 – 4.43 (m, 3H), 3.94 – 3.72 (m, 1H), 3.67 – 3.33 (m, 2H), 3.29 – 3.03 (m, 2H), 3.00 – 2.71 (m, 2H), 2.62 – 2.27 (m, 1H), 1.82 – 1.66 (m, 1H), 1.60 (s, 6H), 1.51 – 1.26 (m, 3H), 1.09 – 0.88 (m, 1H), 0.78 – 0.56 (m, 1H). ^{13}C NMR (100 MHz, $CDCl_3$) δ: 171.0, 154.1, 129.2, 126.1, 118.2, 81.0, 62.8, 45.7, 45.3, 43.4, 41.4, 33.4, 33.2, 32.1, 31.7. LCMS (ESI): m/z calcd for $C_{19}H_{27}ClN_2O_3$; found $[M+H]^+$ 367.20

2-(4-chlorophenoxy)-2-methyl-1-(4-(2-((oxiran-2-ylmethyl)amino)ethyl)piperidin-1-yl)propan-1-one **(23) 1074311**

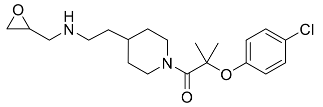

Obtained using procedure E on 0.4 mmol scale, colorless oil, 45.0 mg, 30% yield. ^{1}H NMR (400 MHz, $CDCl_3$) δ: 7.18 (d, *J* = 9.0 Hz, 2H), 6.76 (d, *J* = 9.0 Hz, 2H), 4.71 – 4.43 (m, 3H), 4.29 – 4.17 (m, 2H), 3.67 – 3.33 (m, 2H), 3.29 – 3.03 (m, 2H), 3.00 – 2.71 (m, 2H), 2.62 – 2.27 (m, 2H), 1.82 – 1.66 (m, 1H), 1.60 (s, 6H), 1.51 – 1.26 (m, 3H), 1.09 – 0.88 (m, 1H), 0.78 – 0.56 (m, 1H). ^{13}C NMR (100 MHz, $CDCl_3$) δ: 171.0, 154.1, 129.2, 126.2, 118.2, 81.0, 45.6, 43.4, 42.6, 37.2, 35.7, 33.6, 32.2, 31.7, 25.9. LCMS (ESI): m/z calcd for $C_{20}H_{29}ClN_2O_3$; found $[M+H]^+$ 380.04.

2-(4-chlorophenoxy)-2-methyl-1-(4-(3-((oxiran-2-ylmethyl)amino)propyl)piperidin-1-yl)propan-1-one **(24) 1074309**

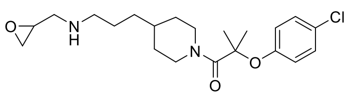

Obtained using procedure E on 0.2 mmol scale, colorless oil, 8.0 mg, 10% yield. ^{1}H NMR (400 MHz, $CDCl_3$) δ: 7.18 (d, *J* = 9.0 Hz, 2H), 6.76 (d, *J* = 9.0 Hz, 2H), 4.73 – 4.55 (m, 3H), 4.30 – 4.21 (m, 2H), 3.67 – 3.33 (m, 2H), 3.29 – 3.03 (m, 2H), 3.04 – 2.74 (m, 2H), 2.61 – 2.42 (m, 2H), 1.64 – 1.62 (m, 1H), 1.61 (s, 6H), 1.54 – 1.28 (m, 3H), 1.24 – 1.08 (m, 2H), 1.06 – 0.89 (m, 1H), 0.76 – 0.52 (m, 1H). ^{13}C NMR (100 MHz, $CDCl_3$) δ: 171.4, 154.5, 129.5, 127.0, 126.5, 118.6, 81.4, 46.1, 43.8, 43.4, 35.9, 33.3, 32.7, 32.1, 27.3, 26.4, 26.3. LCMS (ESI): m/z calcd for $C_{21}H_{31}ClN_2O_3$; found $[M+H]^+$ 395.80

Procedure F. The HCl salt (1 equiv) was suspended in 2 ml dry DCM. At 0°C, DIPEA (4 equiv) was added. After 10 min, chloroacetyl chloride **25** was added slowly (1.2 equiv). Stirring at 0°C for 30min and then at rt for 2h. The reaction mixture was quenched with sat. $NaHCO_3$ (10 ml) and extracted with DCM (3 x 10 ml). The combined organic phases were dried over $MgSO_4$, filtered and concentrated under reduced pressure. The obtained oil was purified with HPLC (column C18, H_2O – CH_3CN + 0.05% formic acid, gradient 30-100% CH_3CN in H_2O, 20 min total).

2-chloro-N-(1-(2-(4-chlorophenoxy)-2-methylpropanoyl)piperidin-4-yl)acetamide **(26) 1074360**

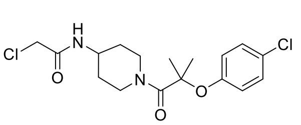

Obtained using procedure F on 0.3 mmol scale, colorless oil, 28.0 mg, 25% yield. ^{1}H NMR (400 MHz, $CDCl_3$) δ 7.20 – 7.16 (m, 2H), 6.77 – 6.73 (m, 2H), 6.32 (d, *J* = 7.6 Hz, 1H), 4.59 – 4.57 (m, 2H), 3.98 (s, 2H), 3.93 – 3.85 (m, 1H), 3.06 (t, *J* = 12.3 Hz, 1H), 2.75 (t, *J* = 12.2 Hz, 1H), 1.93 (d, *J* = 11.9 Hz, 1H), 1.78 (d, *J* = 12.1 Hz, 1H), 1.61 (s, 6H),

1.30 -1.26 (m, 1H), 0.97 – 0.87 (m, 1H). ^{13}C NMR (100 MHz, $CDCl_3$) δ 171.3, 165.2, 154.0, 129.3, 126.4, 118.2, 81.0, 47.0, 44.2, 42.4, 42.1, 32.0, 31.5, 26.1, 25.8. LCMS (ESI): m/z calcd for $C_{17}H_{22}Cl_2N_2O_3$; found $[M+H]^+$ 374.30

2-chloro-N-(1-(2-(4-chlorophenoxy)-2-methylpropanoyl)piperidin-4-yl)acetamide **(27) 1074202**

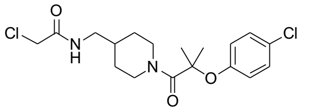

Obtained using procedure F on 0.1 mmol scale, white semi-solid, 30.0 mg, yield 78%. ^{1}H NMR (400 MHz, $CDCl_3$) δ 7.18 (d, *J* = 9.0 Hz, 2H), 6.75 (d, *J* = 9.0 Hz, 2H), 6.58 (s, 1H), 4.68 (d, *J* = 13.3 Hz, 2H), 4.03 (s, 2H), 3.10 (td, *J* = 6.3, 2.6 Hz, 2H), 2.88 (t, *J* = 13.0 Hz, 1H), 2.55 (t, *J* = 12.3 Hz, 1H), 1.71 (dd, *J* = 15.9, 7.3 Hz, 2H), 1.62 (s, 6H), 1.54 – 1.53 (m, 1H), 1.06 (dd, *J* = 22.3, 10.1 Hz, 1H), 0.75 (dd, *J* = 22.1, 9.5 Hz, 1H). ^{13}C NMR (100 MHz, $CDCl_3$) δ 171.2, 166.0, 154.1, 129.2, 126.2, 118.2, 81.0, 45.3, 44.7, 43.0, 42.6, 36.1, 30.0, 29.4, 26.0, 25.9. LCMS (ESI): m/z calcd for $C_{18}H_{24}Cl_2N_2O_3$; found $[M+H]^+$ 378.09

2-chloro-N-(2-(1-(2-(4-chlorophenoxy)-2-methylpropanoyl)piperidin-4-yl)ethyl)acetamide **(28) 1074210**

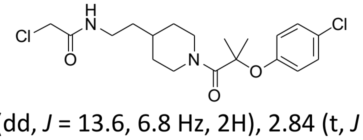

Obtained using procedure F on 0.15 mmol scale, yellow oil, 38.9 mg, 65% yield. HPLC retention time 12.5 min. ^{1}H NMR (400 MHz, $CDCl_3$) δ 7.15 (d, *J* = 9.0 Hz, 2H), 6.73 (d, *J* = 9.0 Hz, 2H), 6.53 (b, 1H), 4.62 (d, *J* = 13.2 Hz, 2H), 4.00 (s, 2H), 3.26 (dd, *J* = 13.6, 6.8 Hz, 2H), 2.84 (t, *J* = 12.4 Hz, 1H), 2.51 (t, *J* = 12.2 Hz, 1H), 1.91 (b, 1H), 1.71 (d, *J* = 12.6 Hz, 2H), 1.60 (s, 6H), 1.44 – 1.33 (m, 2H), 1.05 – 1.00 (m, 1H), 0.74 – 0.68 (m, 1H). ^{13}C NMR (100 MHz, $CDCl_3$) δ 171.1, 165.8, 154.2, 129.2, 126.2, 118.3, 81.1, 45.7, 43.4, 42.6, 37.2, 35.8, 33.6, 32.3, 31.8, 26.0, 25.7. LCMS (ESI): m/z calcd for $C_{19}H_{26}Cl_2N_2O_3$; found $[M+H]^+$ 401.40, $[M+Na]^+$ 423.10

2-chloro-N-(3-(1-(2-(4-chlorophenoxy)-2-methylpropanoyl)piperidin-4-yl)propyl)acetamide **(29) 1074359**

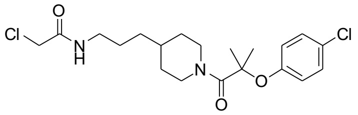

Obtained using procedure F on 0.15 mmol scale colorless oil, 34.0 mg, 55% yield. HPLC retention time 13.0 min. ^{1}H NMR (400 MHz, $CDCl_3$) δ 7.16 (d, *J* = 8.9 Hz, 2H), 6.75 (d, *J* = 8.9 Hz, 2H), 6.57 (s, 1H), 4.64 – 4.61 (m, 2H), 4.02 (s, 2H), 3.24 (dd, *J* = 13.5, 6.9 Hz, 2H), 2.83 (t, *J* = 12.5 Hz, 1H), 2.50 (t, *J* = 12.3 Hz, 1H), 2.04 – 2.03 (m, 1H), 1.69 – 1.67 (m, 1H), 1.61 (s, 6H), 1.52 – 1.38 (m, 3H), 1.17 – 1.12 (m, 2H), 1.00 – 0.96 (m, 1H), 0.68 – 0.64 (m, 1H).^{13}C NMR (100 MHz, $CDCl_3$) δ 171.0, 165.8, 154.2, 129.2, 126.1, 118.2, 81.0, 45.8, 43.6, 42.6, 39.9, 35.6, 33.2, 32.3, 31.8, 26.3, 26.0. LCMS (ESI): m/z calcd for $C_{20}H_{28}Cl_2N_2O_3$; found $[M+H]^+$ 415.20, $[M+Na]^+$ 437.16

Scheme 2. Synthetic route for *gem*-dimethyl α-chloro-ketone derivative **37 (1074203)** [a]

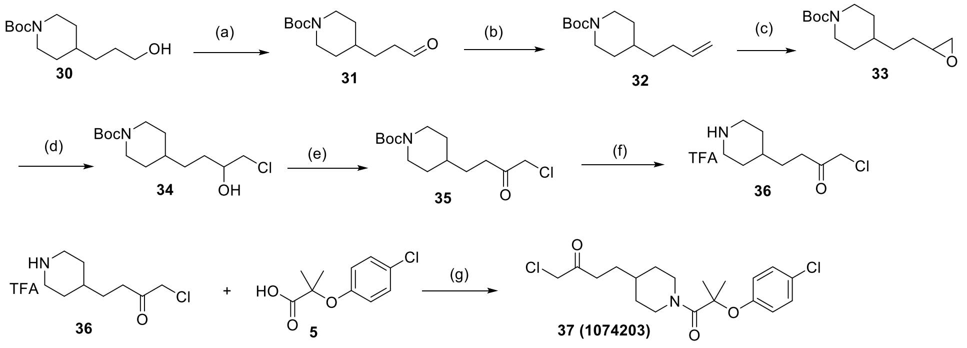

[a] Reagents and conditions: (a) DMP, $NaHCO_3$, DCM, 0°C to rt, 1h; (b) CH_3PPh_3Br, KHMDS, THF, -78°C, 1h; (c) *m*-CPBA, DCM, 0°C to rt, 2h; (d) LiCl, AcOH, THF, rt, overnight; (e) DMP, $NaHCO_3$, DCM, 0°C to rt, 1h; (f) TFA, DCM, 0°C to rt, 2h; (g) HATU, DIPEA, DMF, rt, 2h

Procedure A. To a stirred solution of **30** (2 g, 8.2 mmol, 1 equiv) in DCM (20 ml) at 0°C, $NaHCO_3$ (1.4 g, 16.4 mmol, 2 equiv) and Dess-Martin periodinane (4.2 g, 9.8 mmol, 1.2 equiv) were added sequentially. The resulting mixture was stirred at room temperature for 1 h. The reaction was quenched with sat. aq. $Na_2S_2O_3$ (20 mL) and sat. aq. $NaHCO_3$

(20 mL) and then extracted with DCM (3 x 20 ml). The combined organic phases were dried over Na_2SO_4, filtered and the solvents were removed under reduced pressure. The crude was purified with flash column chromatography (Biotage, hexane - EtOAc, 0-100% EtOAc in hexane).

Procedure B. To a stirred solution of PPh_3CH_3Br (5.4 g, 15 mmol, 2 equiv) in THF (25 ml) at -78°C, KHMDS (1M in THF, 15 ml) was added dropwise. After stirring for 15 min, the mixture was warmed up to room temperature and stirred for 1 h, at which point the resulted yellow solution was re-cooled to -78°C followed by the addition of **31** (1.8 g, 7.5 mmol, 1 equiv). After stirring at -78°C for 1 h, the mixture was warmed up to room temperature and the progress of the reaction was monitored by using TLC. The reaction was diluted with sat. NH_4Cl (50 ml) and EtOAc (30 ml) when the TLC analysis indicated full conversion. The organic phase was separated, and the aqueous phase was extracted with EtOAc (2 × 30 ml). The combined organic phases were washed with brine, dried over $MgSO_4$, filtered, and concentrated under reduced pressure. The crude was purified with flash column chromatography (Biotage, hexane - EtOAc, 0-20% EtOAc in hexane).

Procedure C. To a stirred solution of **32** (1 g, 4.2 mmol, 1equiv) in DCM (10 ml) at 0°C, *m*-CPBA (77%, 1.1 g, 5 mmol, 1.2 equiv) was added carefully. After addition, the reaction mixture was allowed to reach room temperature and stirred for 2 h before TLC analysis indicated full conversion. The reaction was diluted with sat. Na_2SO_3 (20 ml) and extracted with DCM (3 x 10 ml). The combined organic phases were washed with brine, dried over $MgSO_4$, filtered, and concentrated under reduced pressure. The crude was purified with flash column chromatography (Biotage, hexane - EtOAc, 0-100% EtOAc in hexane).

Procedure D. To a solution of **33** (580 mg, 2.3 mmol, 1equiv) and acetic acid (0.4 ml, 6.9 mmol, 3 equiv) in dry THF (6 ml) was added anhydrous LiCl (193 mg, 4.6 mmol, 2 equiv). The mixture was stirred at room temperature overnight. The reaction was diluted with sat. NH_4Cl (20 ml) and extracted with EtOAc (3 x 20 ml). The combined organic phases were washed with brine, dried over $MgSO_4$, filtered, and concentrated. The crude was purified with flash column chromatography (Biotage, hexane - EtOAc, 0-100% EtOAc in hexane).

Procedure E. To a stirred solution of **34** (300 mg, 1.03 mmol, 1 equiv) in DCM (5 ml) at 0°C, $NaHCO_3$ (173 mg, 2.06 mmol, 2 mmol) and Dess-Martin periodinane (524 mg, 1.24 mmol, 1.2 equiv) were added sequentially. The resulting mixture was stirred at room temperature for 1 h. The reaction was quenched with sat. aq. $Na_2S_2O_3$ (10 ml) and sat. aq. $NaHCO_3$ (20 ml) and then extracted with DCM (3 x 10 ml). The combined organic phases were dried over $MgSO_4$, filtered and the solvents were removed under reduced pressure. The crude was purified with flash column chromatography (Biotage, hexane - EtOAc, 0-100% EtOAc in hexane).

Procedure F. Intermediate **35** (120 mg, 0.42 mmol, 1 equiv) was dissolved in DCM (3 ml) and cooled to 0°C. TFA (640 ul, 8.3 mmol, 20 equiv) was added dropwise. After addition, the reaction mixture was allowed to reach to room temperature and stirred for 2h. Solvents were removed under reduced pressure to afford the corresponding TFA salt **36**, which was used directly in the next step.

Procedure G. The carboxylic acid **5** (32 mg, 0.15 mmol, 1 equiv) and HATU (68mg, 0.18 mmol, 1.2 equiv) were dissolved in 2 ml dry DMF at 0°C. The TFA amine salt **36** (0.18 mmol, 1.2 equiv) was dissolved in 1 ml DMF, and DIPEA (80 µl, 0.45 mmol, 3 equiv) was added. The solution of the amine was added to the reaction mixture under stirring. Stirring rt for 2h. The reaction mixture was diluted with ethyl acetate (10 ml) and sat. NH_4Cl was added. The organic phase was washed with NH_4Cl (2x10ml) and Brine (1x10ml), and then dried over $MgSO_4$, filtered and concentrated under reduced pressure. The obtained crude was purified by HPLC (column C18, H_2O – CH_3CN + 0.05% formic acid, gradient 30-100% CH_3CN in H_2O, 20 min total).

tert-butyl 4-(3-oxopropyl)piperidine-1-carboxylate **(31)**

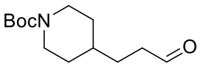

Obtained using procedure A on 8.2 mmol scale, slight yellow oil, 1.9 g, 95% yield. ^{1}H NMR (400 MHz, $CDCl_3$) δ 9.80 (t, *J* = 1.7 Hz, 1H), 4.30 – 3.98 (m, 2H), 2.68 (t, *J* = 12.2 Hz, 2H), 2.49 (td, *J* = 7.6, 1.7 Hz, 2H), 1.74 – 1.53 (m, 4H), 1.47 (s, 9H), 1.44 – 1.22 (m, 1H), 1.11 (ddd, *J* = 24.5, 12.5, 4.4 Hz, 2H). ^{13}C NMR (100 MHz, $CDCl_3$) δ 202.3, 154.8, 79.3, 41.1, 35.5, 31.9, 28.5, 28.4.

tert-butyl 4-(but-3-en-1-yl)piperidine-1-carboxylate **(32)**

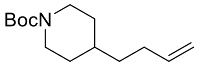

Obtained using procedure B on 7.5 mmol scale, colorless oil, 1.5 g, 83% yield. ^{1}H NMR (400 MHz, $CDCl_3$) δ 5.82 (ddt, *J* = 16.9, 10.2, 6.6 Hz, 1H), 5.08 – 4.86 (m, 2H), 4.08 (s, 2H), 2.69 (t, *J* = 12.2 Hz, 2H), 2.19 – 1.99 (m, 2H), 1.72 – 1.60 (m, 2H), 1.47 (s, 9H), 1.45 – 1.28 (m, 3H), 1.10 (ddd, *J* = 24.1, 12.7, 4.4 Hz, 2H). ^{13}C NMR (100 MHz, $CDCl_3$) δ 154.9, 138.8, 114.4, 79.2, 35.6, 35.4, 32.1, 30.8, 28.5.

tert-butyl 4-(2-(oxiran-2-yl)ethyl)piperidine-1-carboxylate **(33)**

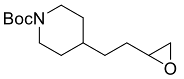

Obtained using procedure C on 4.2 mmol scale, yellow oil, 600 mg, 60% yield. ^{1}H NMR (400 MHz, $CDCl_3$) δ 4.09 (s, 2H), 2.97 – 2.87 (m, 1H), 2.77 (dd, *J* = 4.9, 4.1 Hz, 1H), 2.69 (t, *J* = 12.3 Hz, 2H), 2.48 (dd, *J* = 5.0, 2.7 Hz, 1H), 1.66 (t, *J* = 10.4 Hz, 2H), 1.62 – 1.50 (m, 2H), 1.47 (s, 9H), 1.45 – 1.25 (m, 3H), 1.10 (qd, *J* = 16.6, 4.0 Hz, 2H). ^{13}C NMR (100 MHz, $CDCl_3$) δ 154.9, 79.2, 52.4, 47.1, 35.8, 32.6, 32.1, 29.6, 28.5.

tert-butyl 4-(4-chloro-3-hydroxybutyl)piperidine-1-carboxylate **(34)**

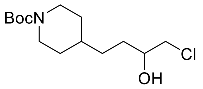

Obtained using procedure D on 2.3 mmol scale, colorless oil, 520 mg, 78% yield. ^{1}H NMR (400 MHz, $CDCl_3$) δ 4.09 (s, 2H), 3.80 (s, 1H), 3.65 (dd, J = 11.1, 3.4 Hz, 1H), 3.50 (dd, J = 11.1, 7.0 Hz, 1H), 2.68 (t, J = 12.2 Hz, 2H), 2.26 (s, 1H), 1.67 (d, J = 11.4 Hz, 2H), 1.62 – 1.51 (m, 2H), 1.47 (s, 9H), 1.45 – 1.25 (m, 3H), 1.18 – 1.03 (m, 2H). ^{13}C NMR (100 MHz, $CDCl_3$) δ 154.9, 79.3, 71.6, 50.4, 36.0, 32.3, 32.1, 31.3, 28.5.

tert-butyl 4-(4-chloro-3-oxobutyl)piperidine-1-carboxylate **(35)**

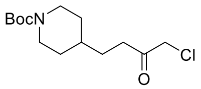

Obtained using procedure E on 1.03 mmol scale, colorless oil, 255 mg, 86% yield. ^{1}H NMR (400 MHz, $CDCl_3$) δ 4.30 – 3.94 (m, 4H), 2.66 (dd, *J* = 18.8, 11.3 Hz, 4H), 1.72 – 1.54 (m, 4H), 1.47 (s, 9H), 1.45 – 1.25 (m, 1H), 1.11 (ddd, *J* = 24.5, 12.5, 4.4 Hz, 2H). ^{13}C NMR (100 MHz, $CDCl_3$) δ 202.7, 154.8, 79.3, 48.1, 36.8, 35.4, 31.9, 29.9, 28.5.

1-chloro-4-(1-(2-(4-chlorophenoxy)-2-methylpropanoyl)piperidin-4-yl)butan-2-one **(37) 1074203**

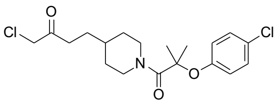

Obtained using procedure G on 0.15 mmol scale colorless oil, 17.7 mg, 31% yield. HPLC retention time 14.5 min. ^{1}H NMR (400 MHz, $CDCl_3$) δ 7.17 (d, *J* = 9.0 Hz, 2H), 6.75 (d, *J* = 9.0 Hz, 2H), 4.64 (d, *J* = 13.0 Hz, 2H), 4.03 (s, 2H), 2.84 (t, *J* = 12.5 Hz, 1H), 2.54 – 2.50 (m, 3H), 1.69 – 1.66 (m, 1H), 1.61 (s, 6H), 1.52 – 1.37 (m, 4H), 1.03 – 0.95 (m, 1H), 0.72 – 0.64 (m, 1H).^{13}C NMR (100 MHz, $CDCl_3$) δ 202.5, 171.2, 154.2, 129.2, 126.2, 118.2, 81.1, 48.0, 45.7, 43.4, 36.4, 35.2, 32.2, 31.5, 29.4, 26.0, 25.9. LCMS (ESI): m/z calcd for $C_{19}H_{25}Cl_2NO_3$; found $[M+H]^+$ 386.14.

Scheme 3. Synthetic route for analog **45 (1075294)** [a]

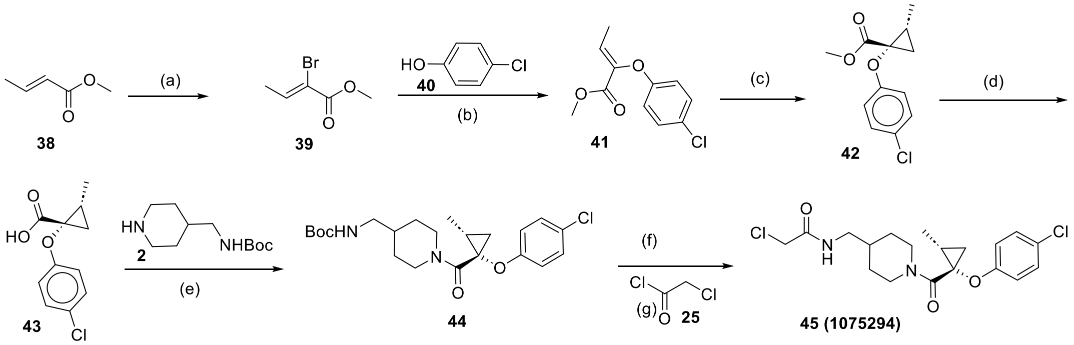

[a] Reagents and conditions: (a) $PyHBr_3$, MeOH, 55°C, 3h, then K_2CO_3, 3h; (b) 4-chlorophenol **40**, Cs_2CO_3, DMF, 80°C, 2h; (c) (CH3)$_3$IO, NaH, DMSO, 0°C to rt 3h; (d) LiOH, THF/H_2O, rt, overnight; (e) 4-(boc-aminomethyl)piperidine **2**, HATU, DIPEA, DMF, 0°C to rt, overnight; (f) TFA, DCM, 0°C to rt, 2h; (g) chloroacetyl chloride **25**, DIPEA, DCM, 0°C to rt, 2h.

Procedure A. To a stirred a solution of **38** (1 g, 10 mmol, 1 equiv) in MeOH (10 ml), $PyHBr_3$ (3.8 g, 12 mmol, 1.2 equiv) was added in one portion. The resulting red mixture was heated to 55°C for 3 h before TLC analysis indicated full conversion. K_2CO_3 (2.8 g, 20 mmol, 2equiv) was added and the suspension was heated for another 3 h before TLC analysis showed full conversion. The precipitate was filtered off and the filtrate was concentrated. The residue was partitioned between EtOAc (30 ml) and saturated aqueous $NaHSO_3$ (30ml). The organic phase was sequentially washed with 1M HCl (30 ml) and brine (30 ml), dried over $MgSO_4$, filtered, and concentrated under reduced pressure. The crude product was purified with flash column chromatography (Biotage, hexane - EtOAc, 0 -10% EtOAc in hexane).

Procedure B. Methyl (*Z*)-2-bromobut-2-enoate **39** (1 g, 5.5 mmol, 1 equiv) and 4-chlorophenol **40** (718 mg, 5.5 mmol, 1 equiv) were dissolved in DMF (10 ml). Cs_2CO_3 (3.6 g, 11 mmol, 1 equiv) was added in one portion and the resulting mixture was heated to 80°C for 2h. The reaction mixture was diluted with sat. NH_4Cl (50 ml) and extracted with EtOAc (3 x 50 ml). The combined organic phases were washed with brine (1 x 50ml), dried over MgSO4, filtered, and concentrated under reduced pressure. The crude product was purified with flash column chromatography (Biotage, hexane - EtOAc, 0 - 20% EtOAc in hexane).

Procedure C. To a suspension of trimethylsulfoxonium iodide (730 mg, 3.3 mmol, 1.1 equiv) in DMSO (10 ml) at 0°C, NaH (132 mg, 60% oil dispersion, 3.3 mmol, 1.1 equiv) was added in portions over 5 minutes. The suspension was stirred for 1 h, and then a solution of methyl (*Z*)-2-(4-chlorophenoxy)but-2-enoate **41** (500 mg, 2.2 mmol, 1 equiv) in DMSO (5 ml) was added dropwise over 10 minutes. The reaction mixture was allowed to warm to room temperature and was stirred for 3 h. The reaction mixture was diluted with sat. NH_4Cl (50 ml) and extracted with EtOAc (3 x 50 ml). The combined organic phases were washed with brine (1 x 50ml), dried over MgSO4, filtered, and concentrated under reduced pressure. The crude product was purified with flash column chromatography (Biotage, hexane - EtOAc, 0 - 20% EtOAc in hexane).

Procedure D. (±)-Methyl (*1S,2R*)-1-(4-chlorophenoxy)-2-methylcyclopropane-1-carboxylate **42** (120 mg, 0.5 mmol, 1equiv) was suspended in a mixture of THF – H_2O (2:1, 4ml) and LiOH (60 mg, 2.5 mmol, 5 equiv) was added. The reaction mixture was stirred rt overnight. Solvents were removed under reduced pressure. The residue was dissolved in 10ml H_2O and extracted with EtOAc (1 x 10ml). The aqua phase was separated, cooled at 0°C and acidified with 2N HCl until pH = 1. Extraction with EtOAc (3x 20ml), drying over $MgSO_4$, filtration and evaporation under reduced pressure. The crude was used directly in the next step.

Procedure E. At 0°C carboxylic acid **43** (110 mg, 0.5 mmol, 1 equiv) and HATU (228 mg, 0.6 mmol, 1.2 equiv) were dissolved in 3ml dry DMF. 4-(Boc-aminomethyl)piperidine **2** (128 mg, 0.6 mmol, 1.2 equiv) was dissolved in 1 ml DMF, and DIPEA (267 µl, 1.5 mmol, 3 equiv) was added. The solution of the amine was added to the reaction mixture under stirring. Stirring at 0 °C for 30 min, then rt overnight. The reaction mixture was diluted with sat. NH_4Cl (10 ml) and extracted with ethyl acetate (3x10ml). The combined organic phases were washed with Brine, dried over MgSO4, filtered and concentrated under reduced pressure. The crude was purified with flash column chromatography (Biotage, hexane - EtOAc, 0-100% EtOAc in hexane).

Procedure F. The boc-protected intermediate **44** (100mg, 0.24 mmol, 1 equiv) was dissolved in DCM (3ml) and cooled at 0°C. TFA (370 µl, 4.8 mmol, 20 equiv) was added dropwise. Stirring at 0°C for 30min, then rt for 2h. Solvents were removed under reduced pressure and the obtained TFA salt was used directly in the next step.

Procedure G. The TFA salt (1 equiv) was suspended in 2 ml dry DCM. At 0°C, DIPEA (4 equiv) was added. After 10 min, chloroacetyl chloride **25** was added slowly (1.2 equiv). Stirring at 0°C for 30min and then at rt for 2h. The reaction mixture was quenched with sat. $NaHCO_3$ (10 ml) and extracted with DCM (3 x 10 ml). The combined organic phases were dried over $MgSO_4$, filtered and concentrated under reduced pressure. The obtained oil was purified with HPLC (column C18, H_2O – CH_3CN + 0.05% formic acid, gradient 30-100% CH_3CN in H_2O, 20 min total).

Methyl (Z)-2-bromobut-2-enoate **(39)**

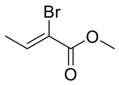

Obtained using procedure A on 10 mmol scale, slight yellow oil, 1.7 g, 95% yield. ^{1}H NMR (400 MHz, CDCl3) δ: 6.78 (q, *J* = 7.6 Hz, 1H), 3.82 (s, 3H), 2.05 (d, *J* = 7.6 Hz, 3H). ^{13}C NMR (100 MHz, CDCl3) δ: 163.0, 141.7, 117.1, 53.2, 17.9. LCMS (ESI): m/z calcd for $C_5H_7BrO_2$; found $[M+H]^+$ 180.82

Methyl (Z)-2-(4-chlorophenoxy)but-2-enoate **(41)**

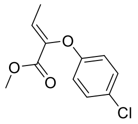

Obtained using procedure B on 5.5 mmol scale, slight yellow oil, 1.1 g, 90% yield. ^{1}H NMR (400 MHz, $CDCl_3$) δ: 7.24 – 7.20 (m, 2H), 6.88 – 6.81 (m, 2H), 6.72 (q, *J* = 7.2 Hz, 1H), 3.71 (s, 3H), 1.77 (d, *J* = 7.2 Hz, 3H). ^{13}C NMR (100 MHz, $CDCl_3$) δ: 163.3, 155.82, 141.6, 129.5, 127.9, 127.1, 116.3, 52.2, 11.5. LCMS (ESI): m/z calcd for $C_{11}H_{11}ClO_3$; found $[M+H]^+$ 227.10

(±)-Methyl (1S,2R)-1-(4-chlorophenoxy)-2-methylcyclopropane-1-carboxylate **(42)**

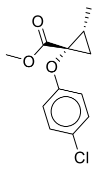

Obtained using procedure C on 2.2 mmol scale, colorless oil, 158 mg, 30% yield. ^{1}H NMR (400 MHz, CDCl3) δ: 7.27 – 7.20 (m, 2H), 6.95 – 6.84 (m, 2H), 3.72 (s, 3H), 1.90 (m, 1H), 1.39 (dd, J = 10.3, 5.5 Hz, 1H), 1.22 (d, *J* = 6.2 Hz, 3H), 0.88 (dd, *J* = 7.8, 5.3 Hz, 1H). ^{13}C NMR (100 MHz, CDCl3) δ: 172.8, 156.4, 129.3, 126.4, 116.5, 61.1, 52.5, 23.9, 22.9, 12.3. LCMS (ESI): m/z calcd for $C_{12}H_{13}ClO_3$; found $[M+Na]^+$ 264.09

(±)-Tert-butyl ((1-((1S,2R)-1-(4-chlorophenoxy)-2-methylcyclopropane-1-carbonyl)piperidin-4-yl)methyl) carbamate **(44)**

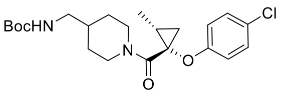

Obtained using procedure E on 0.5 mmol scale, colorless oil, 127 mg, 60% yield (over 2 steps). ^{1}H NMR (400 MHz, $CDCl_3$) δ: 7.19 (m, 2H), 7.02 (m, 2H), 4.56 (s, 1H), 4.41 (s, 2H), 3.14 – 2.73 (m, 3H), 2.70 – 2.18 (m, 1H), 1.76 – 1.56 (m, 5H), 1.43 (s, 9H), 1.17 (s, 2H), 1.10 – 0.88 (m, 3H), 0.62 (s, 1H). ^{13}C NMR (100 MHz, $CDCl_3$) δ: 168.7, 156.4, 156.0, 129.4, 126.4, 116.6, 79.4, 63.4, 45.8, 42.9, 36.9, 30.1, 29.5, 28.4, 19.0, 18.4, 12.1.

(±)-2-Chloro-N-((1-((1S,2R)-1-(4-chlorophenoxy)-2-methylcyclopropane-1-carbonyl)piperidin-4-yl)methyl)acetamide **(45) 1075294**

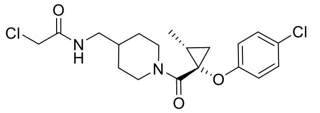

Obtained using procedure G on 0.24 mmol scale, yellow oil, 42 mg, 45% yield (over 2 steps). ^{1}H NMR (400 MHz, $CDCl_3$) δ: 7.25 – 7.17 (m, 2H), 7.05 – 6.94 (m, 2H), 6.64 (b, 1H), 4.45 – 4.43 (m, 2H), 4.02 (s, 2H), 3.32 – 3.06 (m, 2H), 3.04 – 2.88 (m, 1H), 2.73 – 2.49 (m, 1H), 1.90 – 1.52 (m, 5H), 1.20 (s, 3H), 1.08 – 0.93 (m, 2H), 0.64 (d, *J* = 22.5 Hz, 1H). ^{13}C NMR (100 MHz, $CDCl_3$) δ: 168.8, 166.0, 156.4, 129.4, 126.4, 116.7, 63.3, 45.6, 44.9, 42.7, 36.3, 30.2, 29.6, 18.2, 12.1. LCMS (ESI): m/z calcd for $C_{19}H_{24}Cl_2N_2O_3$; found $[M+Na]^+$ 401.04

Scheme 4. Synthetic route for analog **52 (1075308)** [a]

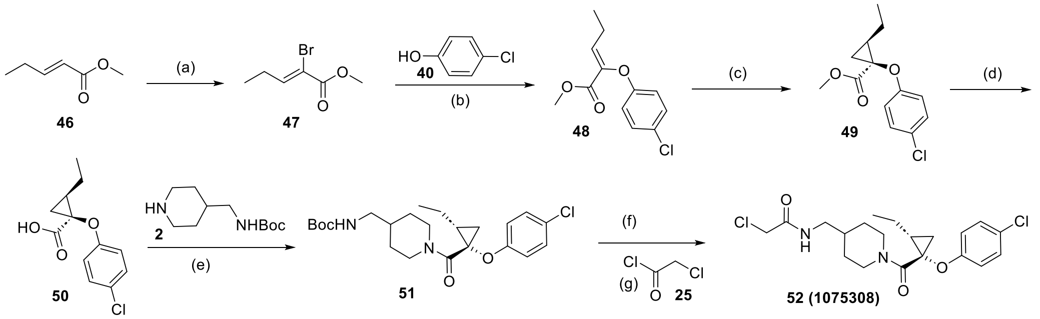

[a] Reagents and conditions: (a) $PyHBr_3$, MeOH, 55°C, 3h, then K_2CO_3, 3h; (b) 4-chlorophenol **40**, Cs_2CO_3, DMF, 80°C, 2h; (c) $(CH3)_3IO$, NaH, DMSO, 0°C to rt 3h; (d) LiOH, THF/H_2O, rt, overnight; (e) 4-(boc-aminomethyl)piperidine **2**, HATU, DIPEA, DMF, 0°C to rt, overnight; (f) TFA, DCM, 0°C to rt, 2h; (g) chloroacetyl chloride **25**, DIPEA, DCM, 0°C to rt, 2h.

Procedure A. To a stirred a solution of **46** (2 g, 17.5 mmol, 1 equiv) in MeOH (20 ml), $PyHBr_3$ (6.7 g, 21.5 mmol, 1.2 equiv) was added in one portion. The resulting red mixture was heated to 55°C for 3h before TLC analysis indicated full conversion. K_2CO_3 (4.8 g, 35 mmol, 2equiv) was added and the reaction mixture was heated for another 3 h before TLC analysis showed full conversion. The reaction mixture was quenched with saturated aqueous $Na_2S_2O_3$ (50ml) and extracted with EtOAc (3x50ml). The combined organic phases were dried over $MgSO_4$, filtered, and concentrated under reduced pressure. The crude product was purified with flash column chromatography (Biotage, hexane - EtOAc, 0 -20% EtOAc in hexane).

Procedure B. Methyl (*Z*)-2-bromopent-2-enoate **47** (1.1 g, 5.5 mmol, 1 equiv) and 4-chlorophenol **40** (718 mg, 5.5 mmol, 1 equiv) were dissolved in DMF (10 ml). Cs_2CO_3 (3.6 g, 11 mmol, 1 equiv) was added in one portion and the resulting mixture was heated to 80°C for 2h. The reaction mixture was diluted with sat. NH_4Cl (50 ml) and extracted with EtOAc (3 x 50 ml). The combined organic phases were washed with brine (1 x 50ml), dried over MgSO4, filtered, and concentrated under reduced pressure. The crude product was purified with flash column chromatography (Biotage, hexane - EtOAc, 0 - 20% EtOAc in hexane).

Procedure C. To a suspension of trimethylsulfoxonium iodide (730 mg, 3.3 mmol, 1.1 equiv) in DMSO (10 ml) at 0°C, NaH (132 mg, 60% oil dispersion, 3.3 mmol, 1.1 equiv) was added in portions over 5 minutes. The suspension was stirred for 1 h, and then a solution of methyl (*Z*)-2-(4-chlorophenoxy)pent-2-enoate **48** (528 mg, 2.2 mmol, 1 equiv) in DMSO (5 ml) was added dropwise over 10 minutes. The reaction mixture was allowed to reach room temperature and was stirred for 3 h. The reaction mixture was diluted with sat. NH_4Cl (50 ml) and extracted with EtOAc (3 x 50 ml). The combined organic phases were washed with brine (1 x 50ml), dried over MgSO4, filtered, and concentrated

under reduced pressure. The crude product was purified with flash column chromatography (Biotage, hexane - EtOAc, 0 - 20% EtOAc in hexane).

Procedure D. (±)-Methyl (*1S,2R*)-1-(4-chlorophenoxy)-2-ethylcyclopropane-1-carboxylate **49** (127 mg, 0.5 mmol, 1equiv) was suspended in a mixture of THF – H_2O (2:1, 4ml) and LiOH (60 mg, 2.5 mmol, 5 equiv) was added. The reaction mixture was stirred rt overnight. Solvents were removed under reduced pressure. The residue was dissolved in 10ml H_2O and extracted with EtOAc (1 x 10ml). The aqua phase was separated, cooled at 0°C and acidified with 2N HCl until pH = 1. Extraction with EtOAc (3x 20ml), drying over $MgSO_4$, filtration and evaporation under reduced pressure. The crude was used directly in the next step.

Procedure E. At 0°C carboxylic acid **50** (100 mg, 0.5 mmol, 1 equiv) and HATU (228 mg, 0.6 mmol, 1.2 equiv) were dissolved in 3ml dry DMF. 4-(Boc-aminomethyl)piperidine **2** (128 mg, 0.6 mmol, 1.2 equiv) was dissolved in 1 ml DMF, and DIPEA (267 µl, 1.5 mmol, 3 equiv) was added. The solution of the amine was added to the reaction mixture under stirring. Stirring at 0 °C for 30 min, then rt overnight. The reaction mixture was diluted with sat. NH_4Cl (10 ml) and extracted with ethyl acetate (3x10ml). The combined organic phases were washed with Brine, dried over MgSO4, filtered and concentrated under reduced pressure. The crude was purified with flash column chromatography (Biotage, hexane - EtOAc, 0-100% EtOAc in hexane).

Procedure F. The boc-protected intermediate **51** (105 mg, 0.24 mmol, 1 equiv) was dissolved in DCM (3ml) and cooled at 0°C. TFA (370 µl, 4.8 mmol, 20 equiv) was added dropwise. Stirring at 0°C for 30min, then rt for 2h. Solvents were removed under reduced pressure and the obtained TFA salt was used directly in the next step.

Procedure G. The TFA salt (1 equiv) was suspended in 2 ml dry DCM. At 0°C, DIPEA (4 equiv) was added. After 10 min, chloroacetyl chloride **25** was added slowly (1.2 equiv). Stirring at 0°C for 30 min and then at rt for 2h. The reaction mixture was quenched with sat. $NaHCO_3$ (10 ml) and extracted with DCM (3 x 10 ml). The combined organic phases were dried over $MgSO_4$, filtered and concentrated under reduced pressure. The obtained oil was purified with HPLC (column C18, H_2O – CH_3CN + 0.05% formic acid, gradient 30-100% CH_3CN in H_2O, 20 min total).

Methyl (Z)-2-bromopent-2-enoate **(47)**

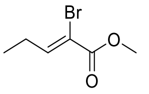

Obtained using procedure A on 17.5 mmol scale, slight yellow oil, 3.2 g, 95% yield. ^1H NMR (400 MHz, $CDCl_3$) δ: 6.70 (t, *J* = 7.8 Hz, 1H), 3.84 (s, 3H), 2.54 (p, *J* = 7.6 Hz, 2H), 1.09 (t, *J* = 7.5 Hz, 3H). ^{13}C NMR (100 MHz, $CDCl_3$) δ: 163.2, 150.7, 110.2, 53.0, 25.3, 12.6. LCMS (ESI): m/z calcd for $C_6H_9BrO_2$; found $[M+H]^+$ 194.91

Methyl (Z)-2-(4-chlorophenoxy)pent-2-enoate **(48)**

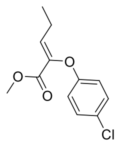

Obtained using procedure B on 5.5 mmol scale, slight yellow oil, 1.1 g, 86% yield. ^1H NMR (400 MHz, $CDCl_3$) δ: 7.28 – 7.23 (m, 2H), 6.92 – 6.83 (m, 2H), 6.67 (t, *J* = 7.6 Hz, 1H), 3.75 (s, 3H), 2.23 (p, *J* = 7.6 Hz, 2H), 1.06 (t, *J* = 7.6 Hz, 3H). ^{13}C NMR (100 MHz, $CDCl_3$) δ: 163.4, 156.0, 140.2, 134.3, 129.5, 127.1, 116.8, 52.2, 19.3, 12.8. LCMS (ESI): m/z calcd for $C_{12}H_{13}ClO_3$; found $[M+H]^+$ 241.05

(±)-Methyl (1S,2R)-1-(4-chlorophenoxy)-2-ethylcyclopropane-1-carboxylate **(49)**

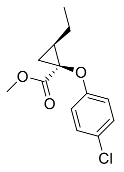

Obtained using procedure C on 3.3 mmol scale, colorless oil, 250 mg, 30% yield. ^1H NMR (400 MHz, $CDCl_3$) δ: 7.26 – 7.21 (m, 2H), 6.92 – 6.79 (m, 2H), 3.73 (s, 3H), 1.85 – 1.74 (m, 2H), 1.67 – 1.60 (m, 1H), 1.50 – 1.37 (m, 1H), 1.13 – 0.97 (m, 3H), 0.91 – 0.83 (m, 1H). ^{13}C NMR (100 MHz, $CDCl_3$) δ: 172.1, 156.2, 129.3, 126.4, 116.5, 61.3, 52.6, 31.9, 22.0, 21.1, 13.4. LCMS (ESI): m/z calcd for $C_{13}H_{15}ClO_3$; found $[M+H]^+$ 255.29

(±)-Tert-butyl ((1-((1S,2R)-1-(4-chlorophenoxy)-2-ethylcyclopropane-1-carbonyl)piperidin-4-yl)methyl)carbamate **(51)**

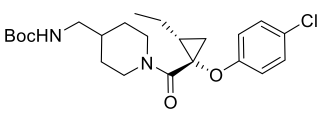

Obtained using procedure E on 0.5 mmol scale, colorless oil, 127 mg, 60% yield (over 2 steps). ^{1}H NMR (400 MHz, $CDCl_3$) δ: 7.26 – 7.14 (m, 2H), 7.08 – 6.93 (m, 2H), 4.75 – 4.32 (m, 3H), 3.23 – 2.77 (m, 3H), 2.59 (dt, *J* = 20.7, 10.3 Hz, 1H), 1.86 – 1.59 (m, 5H), 1.44 (s, 9H), 1.19 – 0.85 (m, 6H), 0.62 (s, 1H). ^{13}C NMR (100 MHz, $CDCl_3$) δ: 168.7, 166.9, 156.2, 129.5, 126.4, 116.8, 64.6, 45.7, 43.0, 36.8, 29.7, 28.4, 21.4, 20.7 (2C), 17.1, 14.2, 13.6, 13.2.

(±)-2-chloro-N-((1-((1S,2R)-1-(4-chlorophenoxy)-2-ethylcyclopropane-1-carbonyl)piperidin-4-yl)methyl)acetamide **(52) 1075308**

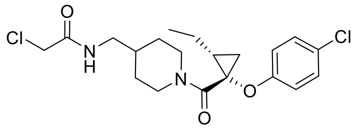

Obtained using procedure G on 0.24 mmol scale, yellow oil, 42 mg, 45% yield (over 2 steps). ^{1}H NMR (400 MHz, $CDCl_3$) δ: 7.25 – 7.18 (m, 2H), 7.03 – 6.91 (m, 2H), 6.63 (s, 1H), 4.69 – 4.31 (m, 2H), 4.05 (s, 2H), 3.33 – 2.82 (m, 3H), 2.66 – 2.48 (m, 1H), 1.87 – 1.60 (m, 5H), 1.50 – 1.38 (m, 2H), 1.04 (dt, *J* = 26.0, 7.3 Hz, 5H), 0.61 (s, 1H). ^{13}C NMR (100 MHz, $CDCl_3$) δ: 168.7, 166.0, 156.4, 129.4, 126.4, 116.6, 63.8, 44.9, 42.7, 36.3, 30.1, 20.6, 13.6. LCMS (ESI): m/z calcd for $C_{20}H_{26}Cl_2N2O_3$; found $[M+Na]^+$ 413.09

Scheme 5. Synthetic route for analog **60 (1075343)** [a]

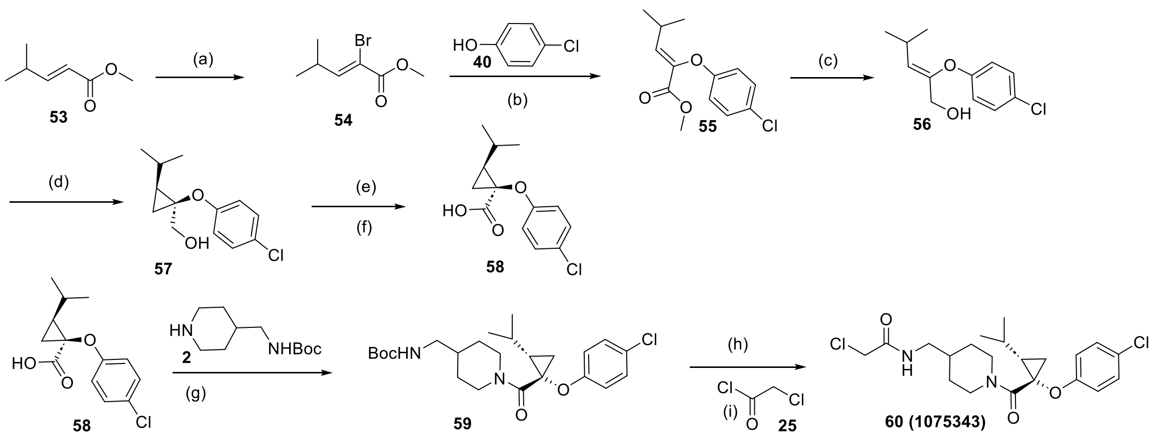

[a] Reagents and conditions: (a) $PyHBr_3$, MeOH, 55°C, 3h, then K_2CO_3, 3h; (b) 4-chlorophenol **40**, Cs_2CO_3, DMF, 80°C, 2h; (c) DIBAL-*H*, DCM, 0°C, 2h; (d) $ZnEt_2$, CH_2I_2, DCM, 0°C, 4h; (e) DMP, DCM, rt, 1h; (f) KMnO4, *t*-BuOH, acetone, rt, 2h; (g) 4-(boc-aminomethyl)piperidine **2**, HATU, DIPEA, DMF, 0°C to rt, overnight; (h) TFA, DCM, 0°C to rt, 2h; (i) chloroacetyl chloride **25**, DIPEA, DCM, 0°C to rt, 2h.

Procedure A. To a stirred a solution of **53** (2.2 g, 17.5 mmol, 1 equiv) in MeOH (20 ml), $PyHBr_3$ (6.7 g, 21.5 mmol, 1.2 equiv) was added in one portion. The resulting red mixture was heated to 55°C for 3h before TLC analysis indicated full conversion. K_2CO_3 (4.8 g, 35 mmol, 2equiv) was added and the reaction mixture was heated for another 3 h before TLC analysis showed full conversion. The reaction mixture was quenched with saturated aqueous $Na_2S_2O_3$ (50ml) and extracted with EtOAc (3x50 ml). The combined organic phases were dried over $MgSO_4$, filtered, and concentrated under reduced pressure. The crude product was purified with flash column chromatography (Biotage, hexane - EtOAc, 0 -20% EtOAc in hexane).

Procedure B. Methyl (*Z*)-2-bromo-4-methylpent-2-enoate **54** (1.1 g, 5.5 mmol, 1 equiv) and 4-chlorophenol **40** (718 mg, 5.5 mmol, 1 equiv) were dissolved in DMF (10 ml). Cs_2CO_3 (3.6 g, 11 mmol, 1 equiv) was added in one portion and the resulting mixture was heated to 80°C for 2h. The reaction mixture was diluted with sat. NH_4Cl (50 ml) and extracted with EtOAc (3 x 50 ml). The combined organic phases were washed with brine (1 x 50 ml), dried over

$MgSO_4$, filtered, and concentrated under reduced pressure. The crude product was purified with flash column chromatography (Biotage, hexane - EtOAc, 0 - 20% EtOAc in hexane).

Procedure C. To a solution methyl (*Z*)-2-(4-chlorophenoxy)-4-methylpent-2-enoate **55** (1 g, 4 mmol, 1 equiv) in DCM (10 ml) cooled at 0°C, DIBAL-*H* (1M in DCM, 4.8 ml) was added dropwise over 10 minutes. The mixture was stirred for 2 h, before TLC analysis indicated full conversion. The reaction was carefully quenched with 1 N HCl. The aqueous phase was extracted with DCM (3 ×10 ml) and the combined organic phases were washed with brine, dried over $MgSO_4$, filtered, and concentrated under reduced pressure. The crude product was purified with flash column chromatography (Biotage, hexane - EtOAc, 0 - 20% EtOAc in hexane).

Procedure D. To a solution of Et_2Zn (11 ml, 11 mmol, 1.0 M in hexane, 5 equiv) in dry DCM (10 ml) was added dropwise CH_2I_2 (887 ul, 11 mmol, 5 equiv) at 0°C. After the reaction mixture became a white suspension, a solution of (*Z*)-2-(4-chlorophenoxy)-4-methylpent-2-en-1-ol **56** (500 mg, 2.2 mmol, 1 equiv) in DCM (5 ml) was added. The reaction was stirred for 4h at 0 °C and quenched with sat. aq. NH_4Cl (20 ml). The aqueous phase was extracted with DCM (3 ×10 ml) and the combined organic phases were washed with brine, dried over $MgSO_4$, filtered, and concentrated under reduced pressure. The crude product was purified with flash column chromatography (Biotage, hexane - EtOAc, 0 - 20% EtOAc in hexane).

Procedures E and F. To a solution of (±)-((*1S,2S*)-1-(4-chlorophenoxy)-2-isopropylcyclopropyl)methanol **57** (140 mg, 0.58 mmol, 1 equiv) in DCM (5 mL) was added Dess-Martin periodinane (295 mg, 0.7 mmol, 1.2 equiv) in small portions at room temperature. The resulting mixture was stirred for 1h and then it was quenched with sat. aq. $Na_2S_2O_3$ (10 ml) and sat. aq. $NaHCO_3$ (10 ml) and extracted with DCM (3 x 10ml). The combined organic layers were dried over anhydrous Na_2SO_4, filtered and concentrated under reduced pressure. The residue was directly dissolved in *t*-BuOH (2 ml) and acetone (2 ml). $KMnO_4$ (275 mg, 1.7 mmol, 3 equiv) was added in one portion and resulting the red mixture was stirred at room temperature for 2 h before TLC analysis indicated full conversion. The reaction was diluted with H_2O (10 ml) and extracted with CH_2Cl_2 (3 x 10 ml). The combined organic phases were washed with brine, dried over $MgSO_4$, filtered, and concentrated under reduced pressure. The crude product was purified with flash column chromatography (Biotage, DCM - MeOH, 0 - 20% MeOH in DCM).

Procedure G. At 0°C carboxylic acid **58** (63 mg, 0.25 mmol, 1 equiv) and HATU (114 mg, 0.3 mmol, 1.2 equiv) were dissolved in 2 ml dry DMF. 4-(Boc-aminomethyl)piperidine **2** (64 mg, 0.3 mmol, 1.2 equiv) was dissolved in 1 ml DMF, and DIPEA (134 µl, 0.75 mmol, 3 equiv) was added. The solution of the amine was added to the reaction mixture under stirring. Stirring at 0 °C for 30 min, then rt overnight. The reaction mixture was diluted with sat. NH_4Cl (10 ml) and extracted with ethyl acetate (3x10 ml). The combined organic phases were washed with Brine, dried over MgSO4, filtered and concentrated under reduced pressure. The crude was purified with flash column chromatography (Biotage, hexane - EtOAc, 0-100% EtOAc in hexane).

Procedure H. The boc-protected intermediate **59** (80 mg, 0.18 mmol, 1 equiv) was dissolved in DCM (3ml) and cooled at 0°C. TFA (278 µl, 3.6 mmol, 20 equiv) was added dropwise. Stirring at 0°C for 30min, then rt for 2h. Solvents were removed under reduced pressure and the obtained TFA salt was used directly in the next step.

Procedure I. The TFA salt (1 equiv) was suspended in 2 ml dry DCM. At 0°C, DIPEA (4 equiv) was added. After 10 min, chloroacetyl chloride **25** was added slowly (1.2 equiv). Stirring at 0°C for 30min and then at rt for 2h. The reaction mixture was quenched with sat. $NaHCO_3$ (10 ml) and extracted with DCM (3 x 10 ml). The combined organic phases were dried over $MgSO_4$, filtered and concentrated under reduced pressure. The obtained oil was purified with HPLC (column C18, H_2O – CH_3CN + 0.05% formic acid, gradient 30-100% CH_3CN in H_2O, 20 min total).

Methyl (Z)-2-bromo-4-methylpent-2-enoate **(54)**

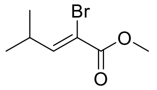

Obtained using procedure A on 17.5 mmol scale, slight yellow oil, 3.2 g, 95% yield. ^{1}H NMR (400 MHz, $CDCl_3$) δ: 7.12 (d, *J* = 9.3 Hz, 1H), 3.84 (s, 3H), 2.88 (ddt, *J* = 13.4, 9.3, 6.7 Hz, 1H), 1.12 – 1.09 (m, 6H). ^{13}C NMR (100 MHz, $CDCl_3$) δ: 163.2, 152.4, 113.6, 53.1, 34.2, 20.9. LCMS (ESI): m/z calcd for $C_7H_{11}BrO_2$; found $[M+H]^+$ 207.01

Methyl (Z)-2-(4-chlorophenoxy)-4-methylpent-2-enoate **(55)**

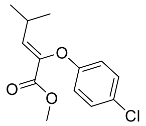

Obtained using procedure B on 5.5 mmol scale, slight yellow oil, 1.1 g, 85% yield. ^{1}H NMR (400 MHz, $CDCl_3$) δ: 7.27 – 7.22 (m, 2H), 6.88 – 6.84 (m, 2H), 6.50 (d, *J* = 10.0 Hz, 1H), 3.73 (s, 3H), 2.85 – 2.67 (m, 1H), 1.05 (d, *J* = 6.7 Hz, 6H). ^{13}C NMR (100 MHz, $CDCl_3$) δ: 163.5, 156.2, 139.2, 129.5, 127.1, 116.9, 116.2, 52.2, 35.1, 21.9. LCMS (ESI): m/z calcd for $C_{13}H_{15}ClO_3$; found $[M+H]^+$ 255.09

(Z)-2-(4-chlorophenoxy)-4-methylpent-2-en-1-ol **(56)**

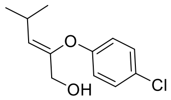

Obtained using procedure C on 4.0 mmol scale, colorless oil, 587 mg, 65% yield. ^{1}H NMR (400 MHz, $CDCl_3$) δ: 7.28 – 7.23 (m, 2H), 6.98 – 6.89 (m, 2H), 5.22 (d, *J* = 9.7 Hz, 1H), 4.11 (t, *J* = 4.1 Hz, 2H), 2.74 – 2.50 (m, 1H), 0.98 (d, *J* = 6.7 Hz, 6H). ^{13}C NMR (100 MHz, $CDCl_3$) δ: 155.5, 147.2, 129.5, 125.2, 117.1, 116.7, 61.2, 24.8, 22.6. LCMS (ESI): m/z calcd for $C_{12}H_{15}ClO_2$; found $[M - OH]^+$ 209.11

(±)-((1S,2S)-1-(4-chlorophenoxy)-2-isopropylcyclopropyl)methanol **(57)**

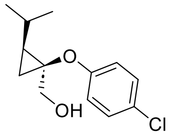

Obtained using procedure D on 2.2 mmol scale, colorless oil, 158 mg, 30% yield. ^{1}H NMR (400 MHz, $CDCl_3$) δ: 7.26 – 7.21 (m, 2H), 7.01 – 6.94 (m, 2H), 4.18 (d, *J* = 12.3 Hz, 1H), 3.36 (d, *J* = 12.3 Hz, 1H), 1.92 (s, 1H), 1.53 – 1.34 (m, 1H), 1.14 (d, *J* = 6.6 Hz, 3H), 1.11 – 1.02 (m, 4H), 0.85 (td, *J* = 9.6, 7.0 Hz, 1H), 0.67 (t, *J* = 6.5 Hz, 1H). ^{13}C NMR (100 MHz, $CDCl_3$) δ: 156.0, 129.3, 126.0, 117.7, 66.1, 64.7, 31.0, 27.9, 22.7, 22.3, 16.6. LCMS (ESI): m/z calcd for $C_{13}H_{17}ClO_2$; found $[M + Na]^+$ 264.14

(±)-(1S,2S)-1-(4-chlorophenoxy)-2-isopropylcyclopropane-1-carboxylic acid **(58)**

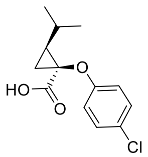

Obtained using procedure F on 0.58 mmol scale, yellow oil, 66 mg, 45% yield (over 2 steps). ^{1}H NMR (400 MHz, $CDCl_3$) δ: 7.25 (d, *J* = 8.9 Hz, 2H), 6.90 (d, *J* = 8.9 Hz, 2H), 1.84 (dd, *J* = 9.5, 5.4 Hz, 1H), 1.69 – 1.49 (m, 2H), 1.15 (d, *J* = 6.3 Hz, 3H), 1.09 (t, *J* = 6.3 Hz, 3H), 0.93 (dd, *J* = 7.7, 5.4 Hz, 1H). ^{13}C NMR (100 MHz, $CDCl_3$) δ: 178.4, 156.2, 129.3, 126.7, 116.9, 60.8, 38.4, 31.1, 28.3, 22.3, 22.0.

(±)-tert-butyl ((1-((1S,2S)-1-(4-chlorophenoxy)-2-isopropylcyclopropane-1-carbonyl)piperidin-4-yl)methyl)carbamate **(59)**

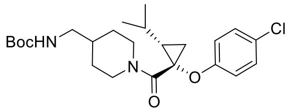

Obtained using procedure G on 0.25 mmol scale, colorless oil, 84 mg, 75% yield. ^{1}H NMR (400 MHz, $CDCl_3$) δ: 7.24 – 7.16 (m, 2H), 7.00 – 6.90 (m, 2H), 4.62 (s, 2H), 4.42 (s, 1H), 3.11 – 2.44 (m, 4H), 1.94 – 1.79 (m, 2H), 1.75 – 1.57 (m, 4H), 1.43 (s, 9H), 1.19 – 1.09 (m, 4H), 1.03 (dd, *J* = 18.9, 6.2 Hz, 3H), 0.95 – 0.76 (m, 1H), 0.58 – 0.48 (m, 1H). ^{13}C NMR (100 MHz, $CDCl_3$) δ: 171.2, 168.8, 156.2, 129.4, 126.5, 116.7, 79.3, 64.6, 60.4, 45.6, 36.8, 28.9, 27.4, 22.7, 22.7, 21.1, 16.9, 14.2.

(±)-2-chloro-N-((1-((1S,2S)-1-(4-chlorophenoxy)-2-isopropylcyclopropane-1-carbonyl)piperidin-4-yl)methyl) acetamide **(60) 1075343**

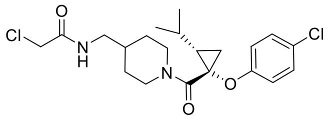

Obtained using procedure I on 0.18 mmol scale, yellow oil, 38 mg, 50% yield (over 2 steps). ^{1}H NMR (400 MHz, $CDCl_3$) δ: 7.23 (d, *J* = 8.9 Hz, 2H), 7.01 – 6.90 (m, 2H), 6.62 (s, 1H), 4.73 – 4.34 (m, 2H), 4.04 (d, *J* = 3.5 Hz, 2H), 3.38 – 2.79 (m, 3H), 2.60 (d, *J* = 24.1 Hz, 1H), 2.13 – 1.53 (m, 6H), 1.45 (d, *J* = 6.2 Hz, 1H), 1.14 (d, *J* = 6.6 Hz, 3H),

1.07 (d, J = 6.8 Hz, 3H), 0.93 (d, J = 25.6 Hz, 1H), 0.54 (s, 1H). ^{13}C NMR (100 MHz, $CDCl_3$) δ: 168.7, 166.0, 156.4, 129.4, 126.5, 116.6, 63.8, 44.9, 43.1, 42.7, 36.3, 30.1, 27.4, 20.6, 17.0. LCMS (ESI): m/z calcd for $C_{21}H_{28}Cl_2N_2O_3$; found $[M + H]^+$ 427.09.

Scheme 6. Synthetic route for analog **67 (1075314)** [a]

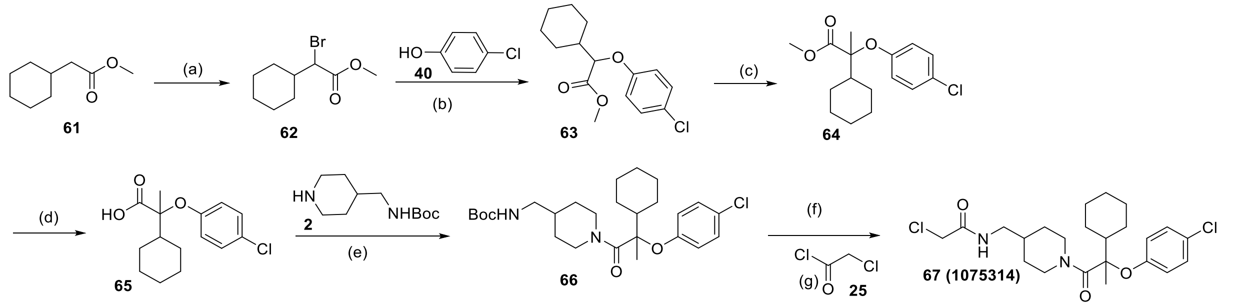

[a] Reagents and conditions: (a) $PyHBr_3$, MeOH, 55°C, 3h; (b) 4-chlorophenol **40**, Cs_2CO_3, DMF, 80°C, 2h; (c) LDA, MeI, THF, -78°C to rt, 2h; (d) LiOH, MeOH/H_2O, reflux, 4h; (e) 4-(boc-aminomethyl)piperidine **2**, HATU, DIPEA, DMF, 0°C to rt, overnight; (f) TFA, DCM, 0°C to rt, 2h; (g) chloroacetyl chloride **25**, DIPEA, DCM, 0°C to rt, 2h.

Procedures A and B. To a stirred a solution of **61** (500 mg, 3.2 mmol, 1 equiv) in MeOH (5 ml), $PyHBr_3$ (1.1 g, 3.5 mmol, 1.2 equiv) was added in one portion. The resulting red mixture was heated to 55°C for 3h before TLC analysis indicated full conversion. The reaction mixture was allowed to reach room temperature and solvents were removed under reduced pressure. The residue was dissolved in DMF (5 ml). 4-Chlorophenol **40** (448 mg, 3.5 mmol, 1.1 equiv) and Cs_2CO_3 (1.6 g, 4.8 mmol, 1.5 equiv) were added in one portion. The resulting mixture was heated to 80°C for 2h. The reaction mixture was quenched with saturated aqueous $Na_2S_2O_3$ (30ml) and extracted with EtOAc (3x30ml). The combined organic phases were washed with 1N HCl, brine, dried over over $MgSO_4$, filtered, and concentrated under reduced pressure. The crude product was purified with flash column chromatography (Biotage, hexane - EtOAc, 0 - 10% EtOAc in hexane).

Procedure C. To a stirred a solution of (±)-methyl 2-(4-chlorophenoxy)-2-cyclohexylacetate **63** (300 mg, 1.1 mmol, 1 equiv) in THF (3 ml) at -78°C, LDA (1M in THF, 1.3 ml) was added dropwise. The reaction mixture was stirred for 1h and then MeI (80 µl, 1.3 mmol, 1.18 equiv) was added. The reaction was allowed to reach rt and stirred for 2h. The reaction was quenched with sat. NH_4Cl (10 ml) and extracted with EtOAc (3x10ml). The combined organic phases were washed with brine, dried over over $MgSO_4$, filtered, and concentrated under reduced pressure. The crude product was purified with flash column chromatography (Biotage, hexane - EtOAc, 0 - 10% EtOAc in hexane).

Procedure D. (±)-Methyl 2-(4-chlorophenoxy)-2-cyclohexylpropanoate **64** (150 mg, 0.5 mmol, 1equiv) was dissolved in a mixture of MeOH – H_2O (1:1, 4ml) and LiOH (60 mg, 2.5 mmol, 5 equiv) was added. The reaction mixture was heated to reflux for 4h. Solvents were removed under reduced pressure. The residue was dissolved in 10ml H_2O and extracted with EtOAc (1 x 10ml). The aqua phase was separated, cooled at 0°C and acidified with 2N HCl until pH = 1. Extraction with EtOAc (3x 20ml), drying over $MgSO_4$, filtration and evaporation under reduced pressure. The crude product was purified with flash column chromatography (Biotage, hexane - EtOAc, 0 - 100% EtOAc in hexane).

Procedure E. At 0°C carboxylic acid **65** (80 mg, 0.3 mmol, 1 equiv) and HATU (137 mg, 0.36 mmol, 1.2 equiv) were dissolved in 2 ml dry DMF. 4-(Boc-aminomethyl)piperidine **2** (77 mg, 0.36 mmol, 1.2 equiv) was dissolved in 1 ml DMF, and DIPEA (156 µl, 0.9 mmol, 3 equiv) was added. The solution of the amine was added to the reaction mixture under stirring. Stirring at 0 °C for 30 min, then rt overnight. The reaction mixture was diluted with sat. NH_4Cl (10 ml) and extracted with ethyl acetate (3x10ml). The combined organic phases were washed with Brine, dried over MgSO4,

filtered and concentrated under reduced pressure. The crude was purified with flash column chromatography (Biotage, hexane - EtOAc, 0-100% EtOAc in hexane).

Procedure F. The boc-protected intermediate **66** (75 mg, 0.15 mmol, 1 equiv) was dissolved in DCM (2 ml) and cooled at 0ºC. TFA (230 µl, 3.0 mmol, 20 equiv) was added dropwise. Stirring at 0ºC for 30min, then rt for 2h. Solvents were removed under reduced pressure and the obtained TFA salt was used directly in the next step.

Procedure G. The TFA salt (1 equiv) was suspended in 2 ml dry DCM. At 0ºC, DIPEA (4 equiv) was added. After 10 min, chloroacetyl chloride **25** was added slowly (1.2 equiv). Stirring at 0ºC for 30min and then at rt for 2h. The reaction mixture was quenched with sat. $NaHCO_3$ (10 ml) and extracted with DCM (3 x 10 ml). The combined organic phases were dried over $MgSO_4$, filtered and concentrated under reduced pressure. The obtained oil was purified with HPLC (column C18, H_2O – CH_3CN + 0.05% formic acid, gradient 30-100% CH_3CN in H_2O, 20 min total).

(±)-Methyl 2-(4-chlorophenoxy)-2-cyclohexylacetate **(63)**

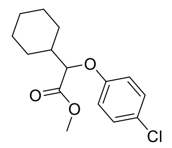

Obtained using procedure B on 3.2 mmol scale, colorless oil, 406 mg, 45% yield (over 2 steps). ^{1}H NMR (400 MHz, $CDCl_3$) δ: 7.26 – 7.20 (m, 2H), 6.86 – 6.78 (m, 2H), 4.36 (d, *J* = 5.8 Hz, 1H), 3.76 (s, 3H), 2.10 – 1.78 (m, 4H), 1.75 – 1.65 (m, 2H), 1.40 – 1.05 (m, 5H). ^{13}C NMR (100 MHz, $CDCl_3$) δ: 171.5, 156.9, 129.5, 126.4, 116.4, 81.6, 52.1, 41.0, 28.9, 28.2, 26.1, 25.9, 25.9. LCMS (ESI): m/z calcd for $C_{15}H_{19}ClO_3$; found $[M+Na]^+$ 306.39.

(±)-Methyl 2-(4-chlorophenoxy)-2-cyclohexylpropanoate **(64)**

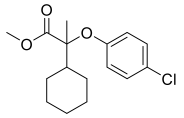

Obtained using procedure C on 1.1 mmol scale, colorless oil, 220 mg, 70% yield. ^{1}H NMR (400 MHz, $CDCl_3$) δ: 7.24 – 7.15 (m, 2H), 6.87 – 6.75 (m, 2H), 3.78 (s, 3H), 2.09 – 1.78 (m, 4H), 1.76 – 1.55 (m, 2H), 1.39 (s, 3H), 1.33 – 0.99 (m, 5H). ^{13}C NMR (100 MHz, $CDCl_3$) δ: 174.3, 154.2, 129.1, 127.2, 120.8, 85.0, 52.2, 46.2, 27.2, 26.9, 26.4, 26.4, 17.1. LCMS (ESI): m/z calcd for $C_{16}H_{21}ClO_3$; found $[M+K]^+$ 334.09.

(±)-Methyl 2-(4-Chlorophenoxy)-2-cyclohexylpropanoic acid **(65)**

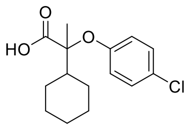

Obtained using procedure D on 0.5 mmol scale, colorless oil, 112 mg, 80% yield. ^{1}H NMR (400 MHz, $CDCl_3$) δ: 7.26 – 7.17 (m, 2H), 6.96 – 6.84 (m, 2H), 2.09 – 1.64 (m, 6H), 1.40 (s, 3H), 1.36 – 1.07 (m, 5H). ^{13}C NMR (100 MHz, $CDCl_3$) δ: 179.2, 153.8, 129.2, 128.0, 121.8, 85.1, 46.8, 27.0, 26.4, 26.3, 26.3, 17.5.

(±)-tert-butyl ((1-(2-(4-chlorophenoxy)-2-cyclohexylpropanoyl)piperidin-4-yl)methyl)carbamate **(66)**

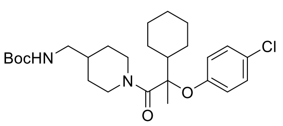

Obtained using procedure E on 0.3 mmol scale, yellow oil, 81 mg, 60% yield. ^{1}H NMR (400 MHz, $CDCl_3$) δ: 7.18 (d, *J* = 8.7 Hz, 2H), 6.80 – 6.73 (m, 2H), 4.78 – 4.72 (m, 2H), 4.58 – 4.49 (m, 1H), 3.01 – 2.79 (m, 3H), 2.60 – 2.48 (m, 1H), 2.13 – 2.11 (m, 1H), 1.90 – 1.82 (m, 3H), 1.78 – 1.62 (m, 4H), 1.53 – 1.50 (m, 1H), 1.43 (s, 9H), 1.40 – 1.38 (m, 3H), 1.30 – 1.04 (m, 7H). ^{13}C NMR (100 MHz, $CDCl_3$) δ: 176.9, 170.9, 153.7, 129.9, 128.0, 121.6, 86.3, 47.3, 46.0, 45.1, 43.3, 29.9, 28.4, 28.1, 27.1, 26.4, 26.3, 17.1.

(±)-2-Chloro-N-((1-(2-(4-chlorophenoxy)-2-cyclohexylpropanoyl)piperidin-4-yl)methyl)acetamide **(67) 1075314**

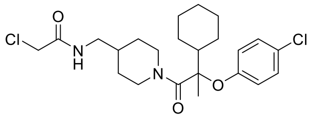

Obtained using procedure G on 0.15 mmol scale, yellow oil, 37 mg, 55% yield (over 2 steps).^{1}H NMR (400 MHz, $CDCl_3$) δ: 7.18 – 7.14 (m, 2H), 6.80 – 6.72 (m, 2H), 6.64 – 6.56 (m, 1H), 4.76 – 4.71 (m, 2H), 4.03 (b, 2H), 3.24 – 3.20 (m, 1H), 3.05 – 2.77 (m, 2H), 2.62 – 2.48 (m, 1H), 2.13 – 2.10 (m, 1H), 1.95 – 1.64 (m, 7H), 1.53 – 1.43 (m, 1H), 1.41 – 1.38 (m, 3H), 1.30 – 1.00 (m, 7H). ^{13}C NMR (100 MHz, $CDCl_3$) δ: 170.9, 166.0, 153.9, 129.3, 126.3, 118.8, 87.0,

46.0, 45.5, 44.9, 44.7, 43.1, 42.6, 36.3, 36.0, 30.7, 29.9, 29.4, 28.0, 26.4, 17.0. LCMS (ESI): m/z calcd for $C_{23}H_{32}Cl_2N_2O_3$; found $[M+H]^+$ 455.10.

Scheme 7. Procedures for the synthesis of analogs **76 (1075321), 77 (1075322), 78 (1075319), 79 (1075320)**. [a]

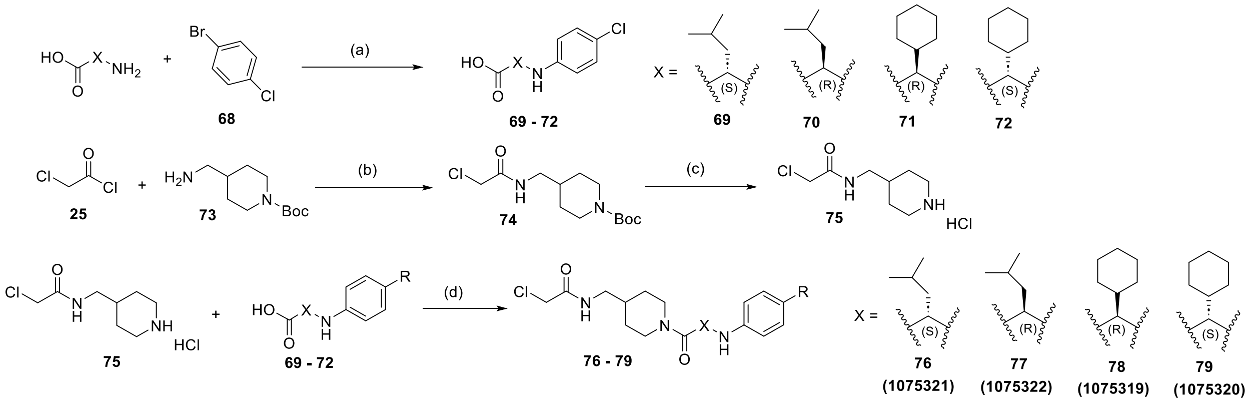

[a] Reagents and conditions: (a) K_2CO_3, CuI (5 mol%), PEG-400, 2-isobutyrylcyclohexanone (ligand, 20 mol %), water, 130°C, 7 h; (b) DIPEA, DCM, 0°C to rt, 4h; (c) 4N HCl/dioxane, rt, 3h; (d) HATU, DIPEA, DMF, 0°C to rt, 2h

Procedure A. Carboxylic acids **69 – 72** were synthesized by modifying previously published procedures.[15,16] The appropriate amino acid (1.2 equiv), 1-bromo-4-chlorobenzene (1.0 equiv), potassium carbonate (2.5 equiv), 2-isobutyrylcyclohexan-1-one (20 mol%) and PEG-400 (1.5 equiv) were taken in water. After bubbling argon gas for 10 min, copper (I) iodide (5 mol%) was added to the mixture and heated to 130 °C for 7 h. The reaction mixture was diluted with water (10 ml), adjusted to pH 4 with glacial acetic acid and extracted with EtOAc (3 x 10 ml). The combined organic phases were washed with brine, dried over $MgSO_4$, filtered and concentrated under reduced pressure. The crude product was purified with flash column chromatography (Biotage, hexane - EtOAc, 0 - 50% EtOAc in hexane).

Procedure B. 1-*N*-Boc-4-(aminomethyl)piperidine **73** (1.0 equiv, 3 mmol, 642 mg) was dissolved in 15 ml dry DCM. DIPEA (3 equiv, 9 mmol, 1.56 ml) was added. The reaction mixture was cooled at 0°C and chloroacetyl chloride **25** (1.2 equiv, 4.2 mmol, 331 µl) was added dropwise. Stirring rt for 4h. The reaction mixture was quenched with sat. $NaHCO_3$ (15 ml) and extracted with DCM (3 x 20 ml). The combined organic phases were dried over $MgSO_4$, filtered and concentrated under reduced pressure. The obtained crude was purified with flash column chromatography (Biotage, hexane - EtOAc, 0-100% EtOAc in hexane).

Procedure C. The boc-protected intermediate **74** was dissolved in 5 ml HCl/dioxane (4N). Stirring rt for 3h. The solvent was removed under reduced pressure and the obtained HCl salt was used directly in the next step.

Procedure D. The appropriate carboxylic acid **69 – 72** (1 equiv) and HATU (1.2 equiv) were dissolved in 2 ml dry DMF at 0°C. The amine HCl salt **75** (1.2 equiv) was dissolved in 1 ml DMF, and DIPEA (3 equiv) was added. The solution of the amine was added to the reaction mixture under stirring. Stirring at 0°C for 30 min, then rt for 2h. The reaction mixture was diluted with sat. NH_4Cl (10 ml) and extracted with ethyl acetate (3x10ml). The combined organic phases were washed with Brine, dried over MgSO4, filtered and concentrated under reduced pressure. The obtained crude was purified by HPLC (column C18, H_2O – CH_3CN + 0.05% formic acid, gradient 30-100% CH_3CN in H_2O, 20 min total).

tert-butyl 4-((2-chloroacetamido)methyl)piperidine-1-carboxylate **(74)**

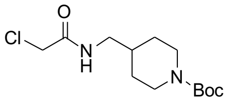

Obtained using procedure B on 3 mmol scale, yellow oil, 520 mg, 60% yield. ^{1}H NMR (400 MHz, $CDCl_3$) δ: 6.67 (b, 1H), 4.13 – 4.11 (m, 2H), 4.06 (s, 2H), 3.21 (t, *J* = 6.0 Hz, 2H), 2.68 (t, *J* = 12.4 Hz, 2H), 1.72 – 1.69 (m, 3H), 1.45 (s, 9H), 1.14 (qd, *J* = 12.9, 4.7 Hz, 2H). ^{13}C NMR (100 MHz, $CDCl_3$) δ: 166.0, 154.8, 79.5, 45.1, 42.7, 36.2, 29.6, 28.4. LCMS (ESI): m/z calcd for $C_{13}H_{23}ClN_2O_3$; found 313.13 $[M+Na]^+$

2-chloro-N-((1-((4-chlorophenyl)-L-leucyl)piperidin-4-yl)methyl)acetamide **(76) 1075321**

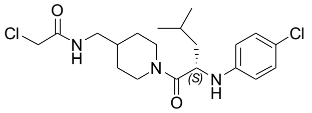

Obtained using procedure D on 0.3 mmol scale, colorless oil, 42.8 mg, 34% yield. ^{1}H NMR (400 MHz, $CDCl_3$) δ 7.10 – 7.06 (m, 2H), 6.72 (b, 1H), 6.54 – 6.50 (m, 2H), 4.59 (d, *J* = 13.0 Hz, 1H), 4.29 (dd, *J* = 9.0, 4.4 Hz, 1H), 4.05 (s, 2H), 3.95 (d, *J* = 13.3 Hz, 1H), 3.28 – 3.05 (m, 4H), 2.60 – 2.53 (m, 1H), 1.87 – 1.69 (m, 4H), 1.57 – 1.49 (m, 2H), 1.16 – 1.05 (m, 2H), 0.96 (s, 3H), 0.95 (s, 3H). ^{13}C NMR (100 MHz, $CDCl_3$) δ 171.8, 166.1, 146.3, 129.1, 122.7, 115.0, 52.3, 45.1, 44.8, 42.6, 42.1, 41.9, 36.2, 30.9, 30.5, 29.4, 24.6, 23.3, 21.9. LCMS (ESI): m/z calcd for $C_{20}H_{29}Cl_2N_3O_2$; found 414.19.

2-chloro-N-((1-((4-chlorophenyl)-D-leucyl)piperidin-4-yl)methyl)acetamide **(77) 1075322**

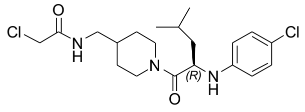

Obtained using procedure D on 0.25 mmol scale, colorless oil, 20.3 mg, 20% yield. ^{1}H NMR (400 MHz, $CDCl_3$) δ: 7.10 – 7.07 (m, 2H), 6.71 (b, 1H), 6.54 – 6.50 (m, 2H), 4.59 (d, *J* = 13.0 Hz, 1H), 4.29 (dd, *J* = 8.9, 4.2 Hz, 1H), 4.06 (s, 2H), 3.96 (d, *J* = 12.4 Hz, 1H), 3.30 – 3.05 (m, 4H), 2.60 – 2.54 (m, 1H), 1.84 – 1.70 (m, 4H), 1.58 – 1.54 (m, 1H), 1.48 – 1.42 (m, 1H), 1.20 – 1.11 (m, 2H), 0.95 (b, 6H). ^{13}C NMR (100 MHz, $CDCl_3$) δ: 171.9, 166.1, 146.3, 129.1, 122.7, 115.0, 52.4, 45.1, 44.8, 42.6, 42.2, 41.9, 36.2, 30.5, 29.5, 29.4, 24.7, 23.3, 22.0. LCMS (ESI): m/z calcd for $C_{20}H_{29}Cl_2N_3O_2$; found 414.24.

(R)-2-chloro-N-((1-(2-((4-chlorophenyl)amino)-2-cyclohexylacetyl)piperidin-4-yl)methyl)acetamide **(78) 1075319**

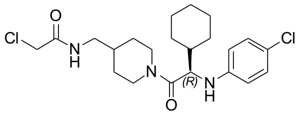

Obtained using procedure D on 0.20 mmol scale, yellow oil, 20 mg, 22% yield. ^{1}H NMR (400 MHz, $CDCl_3$) δ: 7.10 – 7.07 (m, 2H), 6.67 (b, 1H), 6.54 – 6.50 (m, 2H), 4.63 (t, *J* = 12.4 Hz, 1H), 4.06 (d, *J* = 7.7 Hz, 3H), 3.98 (d, *J* = 12.8 Hz, 1H), 3.30 – 3.02 (m, 3H), 2.60 – 2.55 (m, 1H), 1.82 – 1.64 (m, 10H), 1.25 – 1.05 (m, 7H). ^{13}C NMR (100 MHz, $CDCl_3$) δ: 171.1, 166.1, 147.0, 129.0, 122.4, 115.1, 114.9, 58.7, 45.8, 45.2, 44.9, 42.7, 42.0, 36.2, 30.3, 29.5, 28.5, 26.2, 26.1. LCMS (ESI): m/z calcd for $C_{22}H_{31}Cl_2N_3O_2$; found 440.19.

(S)-2-chloro-N-((1-(2-((4-chlorophenyl)amino)-2-cyclohexylacetyl)piperidin-4-yl)methyl)acetamide **(79) 1075320**

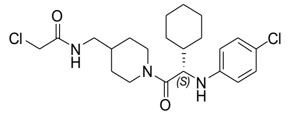

Obtained using procedure D on 0.20 mmol scale, yellow oil, 19.1 mg, 21% yield.
^{1}H NMR (400 MHz, $CDCl_3$) δ: 7.10 – 7.07 (m, 2H), 6.69 (b, 1H), 6.54 – 6.50 (m, 2H), 4.62 (t, *J* = 12.6 Hz, 1H), 4.05 (d, *J* = 8.0 Hz, 3H), 3.97 (d, *J* = 12.8 Hz, 1H), 3.29 – 3.02 (m, 3H), 2.61 – 2.53 (m, 1H), 1.81 – 1.63 (m, 10H), 1.28 – 0.98 (m, 7H). ^{13}C NMR (100 MHz, $CDCl_3$) δ: 171.1, 166.1, 146.9, 129.0, 122.4, 115.1, 114.9, 58.7, 45.7, 44.8, 42.0, 36.2, 30.3, 29.4, 28.5, 26.2, 26.1. LCMS (ESI): m/z calcd for $C_{22}H_{31}Cl_2N_3O_2$; found 440.19.

Scheme 8. Procedure for the synthesis of analogs **83 (1080270), 84 (1080291), 85 (1080268)**. [a]

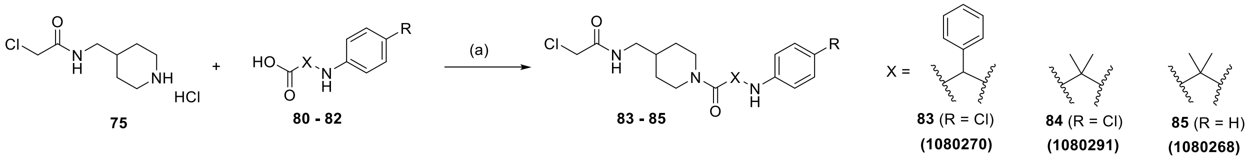

[a] Reagents and conditions: (a) HATU, DIPEA, DMF, 0ºC to rt, 2h

Procedure A. Carboxylic acids **80 – 82** were commercially available. The appropriate carboxylic acid **80 – 82** (1 equiv) and HATU (1.2 equiv) were dissolved in 2 ml dry DMF at 0ºC. The amine HCl salt **75** (1.2 equiv) was dissolved in 1 ml DMF, and DIPEA (3 equiv) was added. The solution of the amine was added to the reaction mixture under stirring. Stirring at 0ºC for 30 min, then rt for 2h. The reaction mixture was diluted with sat. NH_4Cl (10 ml) and extracted with ethyl acetate (3x10ml). The combined organic phases were washed with Brine, dried over MgSO4, filtered and concentrated under reduced pressure. The obtained crude was purified by HPLC (column C18, H_2O – CH_3CN + 0.05% formic acid, gradient 30-100% CH_3CN in H_2O, 20 min total).

2-chloro-N-((1-(2-((4-chlorophenyl)amino)-2-phenylacetyl)piperidin-4-yl)methyl)acetamide **(83) 1080270**

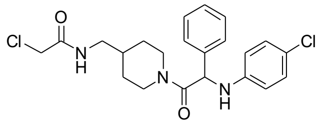

Obtained using procedure A on 0.25 mmol scale, yellow oil, 29.6 mg, yield 28%. HPLC retention time 12.0 min. ^{1}H NMR (400 MHz, $CDCl_3$, racemic mixture) δ: 7.40 – 7.28 (m, 5H), 7.05 (d, *J* = 8.5 Hz, 2H), 6.56 (d, *J* = 8.6 Hz, 2H), 5.23 – 5.20 (m, 1H), 4.67 – 4.58 (m, 1H), 4.05 – 4.00 (m, 4H), 3.24 – 3.20 (m, 1H), 3.02 – 2.97 (m, 2H), 2.63 – 2.56 (m, 1H), 1.72 – 1.61 (m, 2H), 1.54 – 1.20 (m, 2H), 0.93 – 0.90 (m, 1H), 0.22 – 0.19 (m, 1H).^{13}C NMR (100 MHz, $CDCl_3$, racemic mixture) δ: 168.5, 166.0, 144.7, 137.7, 129.1, 129.0, 128.2, 127.6, 122.4, 114.8, 58.3, 45.1, 44.7, 42.6, 36.0, 30.1, 29.3, 29.0, 28.9. LCMS (ESI): m/z calcd for $C_{22}H_{25}Cl_2N_3O_2$; found [M+H]$^+$ 434.21

2-chloro-N-((1-(2-((4-chlorophenyl)amino)-2-methylpropanoyl)piperidin-4-yl)methyl)acetamide **(84) 1080291**

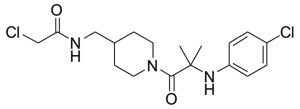

Obtained using procedure A on 0.21 mmol scale, white solid, 14.3 mg, 20% yield. HPLC retention time 11.0 min. ^{1}H NMR (400 MHz, $CDCl_3$) δ: 7.08 – 7.06 (m, 2H), 6.64 (t, *J* = 7.3 Hz, 1H), 6.47 – 6.44 (m, 2H), 4.90 – 4.76 (m, 2H), 4.01 (s, 2H), 3.10 – 3.03 (m, 5H), 1.72 – 1.66 (m, 3H), 1.52 (s, 6H), 1.02 – 0.88 (m, 2H). ^{13}C NMR (100 MHz, $CDCl_3$) δ: 172.4, 166.2, 143.8, 129.0, 122.7, 115.4, 58.5, 44.8, 42.6, 36.1, 29.8, 27.0. LCMS (ESI): m/z calcd for $C_{18}H_{25}Cl_2N_3O_2$; found [M+H]$^+$ 386.19

2-chloro-N-((1-(2-methyl-2-(phenylamino)propanoyl)piperidin-4-yl)methyl)acetamide **(85) 1080268**

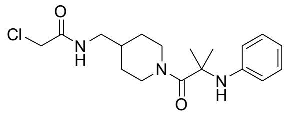

Obtained using procedure A on 0.15 mmol scale, colorless oil, 21.8 mg, 42% yield. HPLC retention time 7.0 min. ^{1}H NMR (400 MHz, $CDCl_3$) δ: 7.18 – 7.14 (m, 2H), 6.74 (t, *J* = 7.3 Hz, 1H), 6.57 (d, *J* = 7.8 Hz, 3H), 5.00 – 4.80 (m, 2H), 4.03 (s, 2H), 3.09 (t, *J* = 6.4 Hz, 2H), 2.98 – 2.90 (m, 1H), 1.76 – 1.67 (m, 3H), 1.57 (s, 6H), 1.56 – 1.41 (m, 2H) 1.03 – 0.90 (m, 2H). ^{13}C NMR (100 MHz, $CDCl_3$) δ: 172.6, 165.9, 141.6, 133.4, 129.2, 118.2, 114.4, 58.4, 44.9, 42.6, 36.2, 29.7, 27.1. LCMS (ESI): m/z calcd for $C_{18}H_{26}ClN_3O_2$; found [M+H]$^+$ 352.13

Scheme 9. Synthetic route for analog **93 (1074378)** [a]

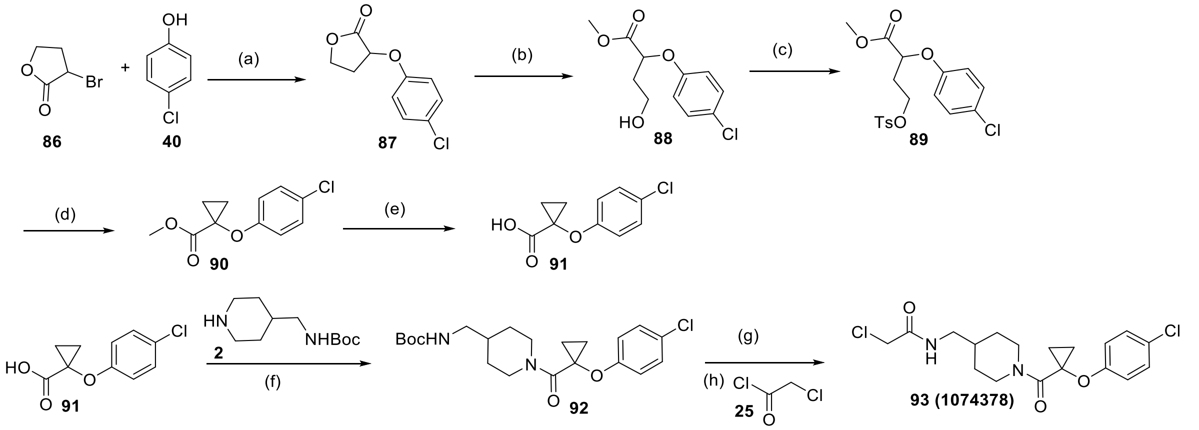

[a] Reagents and conditions: (a) NaH, DMF, 0°C to rt, 4h, then 60°C overnight; (b) I_2, MeOH, 80°C, 40h; (c) TsCl, Et_3N, DCM, 0°C to rt, overnight; (d) LiHMDS, THF, -78°C, 2h; (e) LiOH, THF/H_2O, rt, overnight; (f) 4-(boc-aminomethyl)piperidine **2**, HATU, DIPEA, DMF, 0°C to rt, overnight; (g) 4N HCl/dioxane, rt, 1h; (h) chloroacetyl chloride **25**, DIPEA, DCM, 0°C to rt, 2h.

Procedure A. To a solution of 4-chlorophenol **40** (7.0 g, 54.4 mmol, 1 equiv) in DMF (60 ml) at 0°C was slowly added sodium hydride (4.36 g, 109 mmol, 2 equiv) in portions. The reaction mixture was stirred for 4h and then 3-bromooxolan-2-one **86** (9.8 g, 59.9 mmol, 1.1 equiv) in 10ml DMF was slowly added. Stirring rt overnight. The reaction mixture was quenched with sat. $NaHC0_3$ (100ml) and then extracted with DCM (3x100ml). The combined organic phases were washed with brine, dried over $MgSO_4$, filtered, and concentrated under reduced pressure. The crude was purified with flash column chromatography (Biotage, hexane - EtOAc, 0-100% EtOAc in hexane).

Procedure B. 3-(4-chlorophenoxy)dihydrofuran-2(3*H*)-one **87** (1 gr, 4.71 mmol, 1 equiv) was dissolved in 12.0 ml MeOH. Iodine (20 mg, 0.16 mmol, 0.03 equiv) was added and the reaction mixture was heated at 80°C for 40h. The reaction was quenched with sat. $Na_2S_2O_3$ (15 ml) and extracted with EtOAc (3x20 ml). The combined organic phases were washed with brine, dried over $MgSO_4$, filtered, and concentrated under reduced pressure. The crude was purified with flash column chromatography (Biotage, hexane - EtOAc, 0-100% EtOAc in hexane).

Procedure C. To a solution of methyl 2-(4-chlorophenoxy)-4-hydroxybutanoate **88** (670 mg, 2.74 mmol, 1 equiv) in 10 ml dry DCM at 0°C was added Et_3N (460 µl, 3.3 mmol, 1.2 equiv). Under argon, tosyl chloride (570 mg, 3.0 mmol, 1.1 equiv) was added. The reaction mixture was stirred at 0°C for 1h and then rt overnight. The reaction was quenched with sat. $NaHCO_3$ (10 ml) and extracted with DCM (3 x 10 ml). The combined organic phases were dried over $MgSO_4$, filtered and concentrated under reduced pressure. The crude was purified with flash column chromatography (Biotage, hexane - EtOAc, 0-100% EtOAc in hexane).

Procedure D. To a solution of methyl 2-(4-chlorophenoxy)-4-(tosyloxy)butanoate **89** (556 mg, 1.4 mmol, 1equiv) in 10 ml dry THF at -78 °C under argon was added LiHMDS (544 µl, 2.8 mmol, 2 equiv). Stirring for 2 h. The reaction mixture was quenched with sat. NH_4Cl (10 ml) and extracted with EtOAc (3 x 10ml). The combined organic phases were dried over $MgSO_4$, filtered and concentrated under reduced pressure.

Procedure E. The methyl ester **90** (272 mg, 1.2 mmol, 1equiv) was suspended in a mixture of THF – H_2O (2:1, 10ml) and LiOH (144 mg, 6.0 mmol, 5 equiv) was added. The reaction mixture was stirred rt overnight. Solvents were removed under reduced pressure. The residue was dissolved in 10ml H_2O and extracted with EtOAc (1 x 10ml). The

aqua phase was separated, cooled at 0°C and acidified with 2N HCl until pH = 1. Extraction with EtOAc (3x 20ml), drying over $MgSO_4$, filtration and evaporation under reduced pressure.

Procedure F. At 0°C 1-(4-chlorophenoxy)cyclopropane-1-carboxylic acid **91** (242 mg, 1.14 mmol, 1 equiv) and HATU (520mg, 1.36 mmol, 1.2 equiv) were dissolved in 3ml dry DMF. 4-(Boc-aminomethyl)piperidine **2** (291 mg, 1.36 mmol, 1.2 equiv) was dissolved in 1 ml DMF, and DIPEA (607 µl, 3.4 mmol, 3 equiv) was added. The solution of the amine was added to the reaction mixture under stirring. Stirring at 0 °C for 30 min, then rt overnight. The reaction mixture was diluted with sat. NH_4Cl (10 ml) and extracted with ethyl acetate (3x10ml). The combined organic phases were washed with Brine, dried over MgSO4, filtered and concentrated under reduced pressure. The crude was purified with flash column chromatography (Biotage, hexane - EtOAc, 0-100% EtOAc in hexane). The product was confirmed with LCMS and used directly in the next step.

Procedure G. The boc-protected intermediate **92** was dissolved in 3ml HCl/dioxane (4N). Stirring rt for 3h. The solvent was removed under reduced pressure and the obtained HCl salt was used directly in the next step.

Procedure H. The HCl salt (1 equiv) was suspended in 2 ml dry DCM. At 0°C, DIPEA (4 equiv) was added. After 10 min, chloroacetyl chloride **25** was added slowly (1.2 equiv). Stirring at 0°C for 30min and then at rt for 2h. The reaction mixture was quenched with sat. $NaHCO_3$ (10 ml) and extracted with DCM (3 x 10 ml). The combined organic phases were dried over $MgSO_4$, filtered and concentrated under reduced pressure. The obtained oil was purified with HPLC (column C18, H_2O – CH_3CN + 0.05% formic acid, gradient 30-100% CH_3CN in H_2O, 20 min total).

3-(4-chlorophenoxy)dihydrofuran-2(3H)-one **(87)**

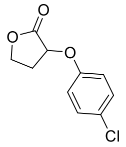

Obtained using procedure A on 54.4 mmol scale, yellow oil, 5.7 g, 49% yield. ^{1}H NMR (400 MHz, $CDCl_3$) δ: 7.25 (d, *J* = 9.0 Hz, 2H), 7.00 (d, *J* = 9.0 Hz, 2H), 5.01 – 4.83 (m, 1H), 4.61 – 4.46 (m, 1H), 4.43 – 4.29 (m, 1H), 2.83 – 2.62 (m, 1H), 2.57 – 2.34 (m, 1H). ^{13}C NMR (100 MHz, $CDCl_3$) δ: 173.3, 155.9, 129.6, 127.4, 117.3, 72.8, 65.4, 31.0, 29.7

methyl 2-(4-chlorophenoxy)-4-hydroxybutanoate **(88)**

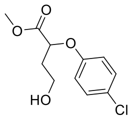

Obtained using procedure B on 4.71 mmol scale, yellow oil, 770 mg, 67% yield. ^{1}H NMR (400 MHz, $CDCl_3$) δ: 7.25 (d, *J* = 9.0 Hz, 2H), 6.85 (d, *J* = 9.0 Hz, 2H), 4.93 – 4.65 (m, 1H), 3.95 – 3.83 (m, 2H), 3.77 (s, 3H), 2.32 – 2.25 (m, 2H), 1.88 – 1.71 (m, 1H). ^{13}C NMR (100 MHz, $CDCl_3$) δ: 172.3, 156.6, 129.8, 127.1, 116.8, 74.5, 58.7, 52.7, 35.6

methyl 2-(4-chlorophenoxy)-4-(tosyloxy)butanoate **(89)**

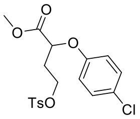

Obtained using procedure C on 2.74 mmol scale, yellow oil, 556 mg, 51% yield.^{1}H NMR (400 MHz, $CDCl_3$) δ: 7.67 – 7.65 (m, 2H), 7.20 – 7.12 (m, 4H), 6.72 – 6.45 (m, 2H), 4.67 – 4.55 (m, 1H), 4.26 – 4.12 (m, 2H), 3.67 (s, 3H), 2.33 (s, 3H), 2.30 – 2.25 (m, 2H). ^{13}C NMR (100 MHz, $CDCl_3$) δ: 170.6, 155.7, 144.8, 132.1, 129.6, 129.1, 127.5, 126.5, 116.1, 72.1, 65.4, 52.3, 31.8, 21.3.

methyl 1-(4-chlorophenoxy)cyclopropane-1-carboxylate **(90)**

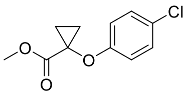

Obtained using procedure D on 1.4 mmol scale, yellow oil, 272 mg, 86% yield. ^{1}H NMR (400 MHz, $CDCl_3$) δ: 7.23 (d, *J* = 9.0 Hz, 2H), 6.72 (d, *J* = 9.0 Hz, 2H), 3.73 (s, 3H), 1.65 – 1.56 (m, 2H), 1.35 – 1.28 (m, 2H). ^{13}C NMR (100 MHz, $CDCl_3$) δ: 172.4, 155.9, 129.2, 126.5, 116.5, 71.0, 53.7, 17.3.

1-(4-chlorophenoxy)cyclopropane-1-carboxylic acid **(91)**

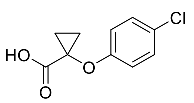

Obtained using procedure E on 1.2 mmol scale, white semi-solid, 242 mg, 95% yield. ^1H NMR (400 MHz, $CDCl_3$) δ: 11.5 (b, 1H), 7.23 (d, *J* = 9.0 Hz, 2H), 6.88 (d, *J* = 9.0 Hz, 2H), 1.70 – 1.56 (m, 2H), 1.25 – 1.29 (m, 2H). ^{13}C NMR (100 MHz, $CDCl_3$) δ: 179.2, 155.9, 129.5, 127.0, 116.9, 71.0, 18.4.

2-chloro-N-((1-(1-(4-chlorophenoxy)cyclopropane-1-carbonyl)piperidin-4-yl)methyl)acetamide **(93) 1074378**

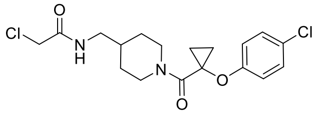

Obtained using procedure H, colorless oil, 80 mg, 20% yield (over 3 steps). ^1H NMR (400 MHz, $CDCl_3$) δ: 7.20 (d, *J* = 9.0 Hz, 2H), 6.96 (d, *J* = 9.0 Hz, 2H), 6.62 (b, 1H), 4.60 – 4.41 (m, 2H), 4.03 (s, 2H), 3.15 – 2.98 (m, 3H), 2.60 – 2.58 (m, 1H), 1.79 – 1.70 (m, 3H), 1.47 – 1.37 (m, 2H), 1.16 – 1.00 (m, 4H). ^{13}C NMR (100 MHz, $CDCl_3$) δ: 168.0, 166.1, 156.0, 129.4, 126.5, 116.6, 79.0, 60.6, 44.8, 42.6, 36.2, 30.2. LCMS (ESI): m/z calcd for $C_{18}H_{22}Cl_2N_2O_3$; found $[M+Na]^+$ 408.12

Scheme 10. Synthetic route for analogs **97 (1076406)** and **98 (1080269)** [a]

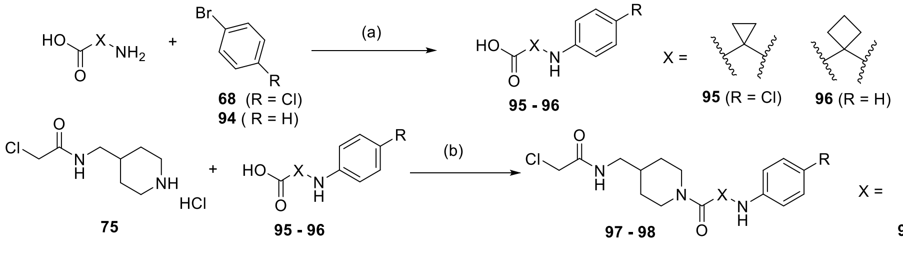

[a] Reagents and conditions: (a) K_2CO_3, CuI (5 mol%), PEG-400, 2-isobutyrylcyclohexanone (ligand, 20 mol %), water, 130°C, 7 h; (b) HATU, DIPEA, DMF, 0°C to rt, 2h

Procedure A. Carboxylic acids **95 – 96** were synthesized by modifying previously published procedures.[15,16] The appropriate amino acid (1.2 equiv), 1-bromo-4-chlorobenzene **68** or bromobenzene **94** (1.0 equiv), potassium carbonate (2.5 equiv), 2-isobutyrylcyclohexan-1-one (20 mol%) and PEG-400 (1.5 equiv) were taken in water. After bubbling argon gas for 10 min, copper (I) iodide (5 mol%) was added to the mixture and heated to 130 °C for 7 h. The reaction mixture was diluted with water (10 ml), adjusted to pH 4 with glacial acetic acid and extracted with EtOAc (3 x 10 ml). The combined organic phases were washed with brine, dried over $MgSO_4$, filtered and concentrated under reduced pressure. The crude product was purified with flash column chromatography (Biotage, hexane - EtOAc, 0 - 50% EtOAc in hexane).

Procedure B. The appropriate carboxylic acid **95 - 96** (1 equiv) and HATU (1.2 equiv) were dissolved in 2 ml dry DMF at 0°C. The amine HCl salt **75** (1.2 equiv) was dissolved in 1 ml DMF, and DIPEA (3 equiv) was added. The solution of the amine was added to the reaction mixture under stirring. Stirring at 0°C for 30 min, then rt for 2h. The reaction mixture was diluted with sat. NH_4Cl (10 ml) and extracted with ethyl acetate (3x10ml). The combined organic phases were washed with Brine, dried over MgSO4, filtered and concentrated under reduced pressure. The obtained crude was purified by HPLC (column C18, H_2O – CH_3CN + 0.05% formic acid, gradient 30-100% CH_3CN in H_2O, 20 min total).

2-chloro-N-((1-(1-((4-chlorophenyl)amino)cyclopropane-1-carbonyl)piperidin-4-yl)methyl)acetamide **(97) 1076406**

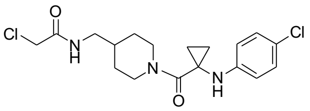

Obtained using procedure B on 0.17 mmol scale, white solid, 16.5 mg, 25% yield. HPLC retention time 10.5 min. ^1H NMR (400 MHz, $CDCl_3$) δ: 7.12 – 7.08 (m, 2H), 6.72 – 6.68 (m, 2H), 6.64 (b, 1H), 4.40 (d, *J* = 13.2 Hz, 2H), 4.12 (b, 1H), 4.03 (s, 2H), 3.16 (t, *J* = 6.0 Hz, 2H), 2.85 – 2.80 (m, 2H), 1.73 – 1.69 (m, 3H), 1.41 (q, *J* = 4.7 Hz, 2H),

1.11 – 1.05 (m, 2H), 0.90 (q, *J* = 4.6 Hz, 2H).^{13}C NMR (100 MHz, $CDCl_3$) δ: 169.7, 166.1, 145.2, 129.0, 122.9, 114.4, 44.9, 42.6, 37.9, 36.3, 29.7, 15.1. LCMS (ESI): m/z calcd for $C_{18}H_{23}Cl_2N_3O_2$; found $[M+H]^+$ 384.23, $[M+Na]^+$ 406.25.

2-chloro-N-((1-(1-(phenylamino)cyclobutane-1-carbonyl)piperidin-4-yl)methyl)acetamide **(98) 1080269**

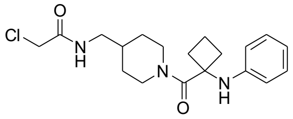

Obtained using procedure B on 0.15 mmol scale, colorless oil, 24.6 mg, 46.0% yield. HPLC retention time 8.5 min. ^{1}H NMR (400 MHz, $CDCl_3$) δ: 7.15 – 7.11 (m, 2H), 6.71 (t, *J* = 7.3 Hz, 1H), 6.58 – 6.55 (m, 3H), 4.62 – 4.59 (m, 1H), 4.20 – 4.18 (m, 1H), 3.99 (s, 2H), 3.10 – 3.00 (m, 2H), 2.90 – 2.83 (m, 2H), 2.58 – 2.54 (m, 1H), 2.17 (s, 1H), 2.08 – 1.87 (m, 4H), 1.74 – 1.60 (m, 4H), 1.00 – 0.96 (m, 2H). ^{13}C NMR (100 MHz, $CDCl_3$) δ: 172.0, 166.0, 145.4, 129.2, 118.1, 113.8, 61.1, 44.9, 42.6, 36.2, 32.9, 32.1, 29.7, 14.7. LCMS (ESI): m/z calcd for $C_{19}H_{26}ClN_3O_2$; found $[M+H]^+$ 364.24, $[M+Na]^+$ 386.19.

Scheme 11. Synthetic route for analogs **117 – 141.** [a]

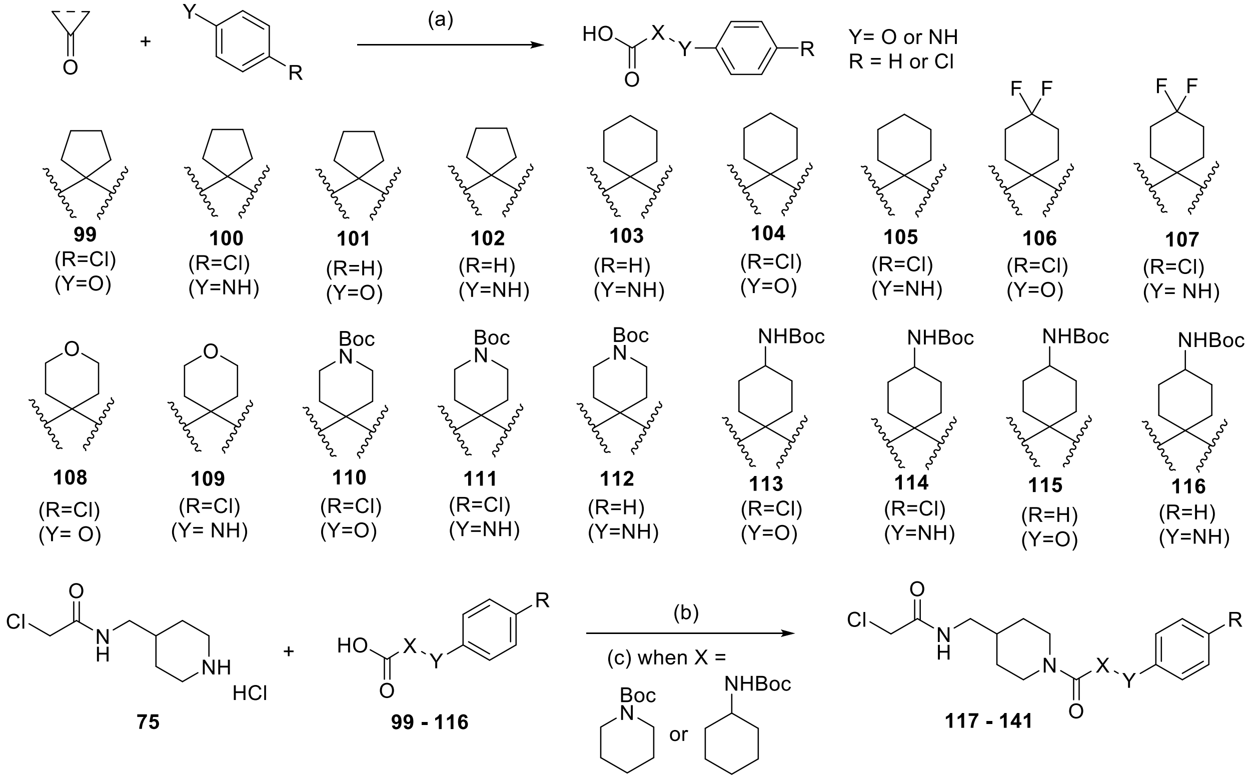

[a] Reagents and conditions: (a) NaOH, $CHCl_3$, THF, 0°C to rt overnight; (b) HATU, DIPEA, DMF, 0°C to rt, 2h; (c) 4N HCl/dioxane, rt, 3h

Procedure A. Carboxylic acids **99 – 116** were synthesized using the Bargellini reaction.[17] Under argon: The appropriate phenol or aniline (3 mmol, 1 equiv) was dissolved in dry THF (40 ml) and cooled at 0°C. NaOH powder (581 mg, 15 mmol, 5 equiv) and the appropriate ketone (9 mmol, 3 equiv) were added as solids. Dry chloroform (1.16 ml, 15 mmol, 5 equiv) was added dropwise over 30 min at 0°C. The reaction mixture was stirred at 0°C for 1h and then rt overnight. In the cases that solid formation was observed, the solid was filtered off and dissolved in water (50 ml). If solid formation was not observed, the reaction mixture was diluted with water (50 ml). In both cases, the aqua phase was extracted with EtOAc (3 x 50ml). The combined organic phases were separated, and the aqua phase was cooled at 0°C and acidified with 1N acetic acid, until pH=3. The acidified aqua phase was extracted with EtOAc (3 x 50ml). The combined organic phases were dried over $MgSO_4$, filtered and the solvent was removed

under reduced pressure. Product formation was confirmed with LCMS. The obtained products were used directly in the next step.

Procedure B. The appropriate carboxylic acid **99 – 116** (1 equiv) and HATU (1.2 equiv) were dissolved in 2 ml dry DMF at 0°C. The amine HCl salt **75** (1.2 equiv) was dissolved in 1 ml DMF, and DIPEA (3 equiv) was added. The solution of the amine was added to the reaction mixture under stirring. Stirring at 0°C for 30 min, then rt for 2h. The reaction mixture was diluted with sat. NH_4Cl (10 ml) and extracted with ethyl acetate (3x10ml). The combined organic phases were washed with Brine, dried over MgSO4, filtered and concentrated under reduced pressure. The obtained crude was purified by HPLC (column C18, H_2O – CH_3CN + 0.05% formic acid, gradient 30-100% CH_3CN in H_2O, 20 min total).

Procedure C. The purified Boc-intermediate was suspended in 3 ml HCl/dioxane (4N) Stirring rt for 3h. The solvent was removed under reduced pressure and the deprotected product was dried under vacuum.

2-Chloro-N-((1-(1-(4-chlorophenoxy)cyclopentane-1-carbonyl)piperidin-4-yl)methyl)acetamide **(117) 1075297**

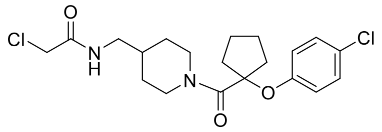

Obtained using procedure B on 0.20 mmol scale, colorless oil, 25.0 mg, 30.0% yield. ^{1}H NMR (400 MHz, $CDCl_3$) δ: 7.18 – 7.14 (m, 2H), 6.77 – 6.73 (m, 2H), 6.57 (b, 1H), 4.58 (t, *J* = 12.6 Hz, 2H), 4.01 (s, 2H), 3.17 – 3.00 (m, 2H), 2.89 – 2.82 (m, 1H), 2.55 – 2.45 (m, 2H), 2.38 – 2.32 (m, 1H), 2.13 – 2.03 (m, 2H), 1.80 – 1.69 (m, 6H), 1.68 – 1.66 (m, 1H), 0.97 (qd, *J* = 12.6, 3.8 Hz, 1H), 0.77 (qd, *J* = 12.6, 3.9 Hz, 1H). ^{13}C NMR (100 MHz, $CDCl_3$) δ: 171.0, 166.0, 154.3, 129.2, 125.9, 117.6, 90.6, 45.4, 44.8, 43.0, 42.6, 37.3, 36.8, 36.2, 30.1, 29.5, 24.7. LCMS (ESI): m/z calcd for $C_{20}H_{26}Cl_2N_2O_3$; found $[M+H]^+$ 413.09.

2-Chloro-N-((1-(1-((4-chlorophenyl)amino)cyclopentane-1-carbonyl)piperidin-4-yl)methyl)acetamide **(118) 1075306**

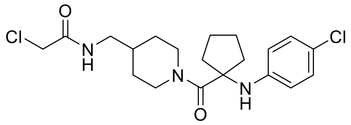

Obtained using procedure B on 0.20 mmol scale, colorless oil, 30.0 mg, 35% yield. ^{1}H NMR (400 MHz, $CDCl_3$) δ: 7.05 (d, *J* = 8.8 Hz, 2H), 6.60 (b, 1H), 6.47 (d, *J* = 8.8 Hz, 2H), 4.65 (d, *J* = 12.8 Hz, 2H), 3.99 (s, 2H), 3.86 – 3.85 (m, 1H), 3.09 – 3.07 (m, 2H), 2.91 – 2.90 (m, 1H), 2.51 – 2.36 (m, 3H), 1.85 – 1.58 (m, 9H), 0.94 – 0.92 (m, 2H). ^{13}C NMR (100 MHz, $CDCl_3$) δ: 172.5, 166.0, 144.3, 129.0, 122.3, 115.0, 68.4, 44.8, 42.6, 36.2, 29.8, 24.1. LCMS (ESI): m/z calcd for $C_{20}H_{27}Cl_2N_3O_2$; found $[M+H]^+$ 412.14.

2-Chloro-N-((1-(1-phenoxycyclopentane-1-carbonyl)piperidin-4-yl)methyl)acetamide **(119) 1075300**

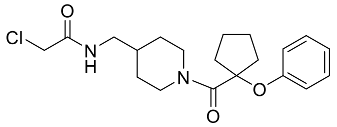

Obtained using procedure B on 0.20 mmol scale, colorless oil, 23.0 mg, 30.0% yield. ^{1}H NMR (400 MHz, $CDCl_3$) δ: 7.25 – 7.17 (m, 2H), 6.94 – 6.88 (m, 1H), 6.81 – 6.79 (m, 2H), 6.56 (b, 1H), 4.61 (dd, *J* = 13.1, 2.6 Hz, 2H), 3.98 (s, 2H), 3.05 – 3.00 (m, 2H), 2.87 – 2.80 (m, 1H), 2.53 – 2.46 (m, 2H), 2.35 – 2.31 (m, 1H), 2.16 – 2.08 (m, 2H), 1.75 – 1.62 (m, 6H), 1.51 (d, *J* = 13.2 Hz, 1H), 0.94 (qd, *J* = 12.6, 3.8 Hz, 1H), 0.70 (qd, *J* = 12.6, 3.9 Hz, 1H). ^{13}C NMR (100 MHz, $CDCl_3$) δ: 171.4, 165.9, 155.6, 129.2, 120.9, 116.3, 90.1, 45.4, 44.8, 42.9, 42.5, 37.4, 36.8, 36.1, 29.9, 29.4, 24.7. LCMS (ESI): m/z calcd for $C_{20}H_{27}ClN_2O_3$; found $[M+H]^+$ 379.19.

2-chloro-N-((1-(1-(phenylamino)cyclopentane-1-carbonyl)piperidin-4-yl)methyl)acetamide **(120) 1080271**

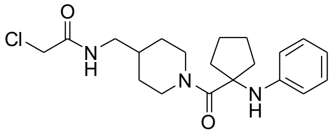

Obtained using procedure B on 0.15 mmol scale, colorless oil, 18.0 mg, 32% yield. HPLC retention time 13.5 min. ^{1}H NMR (400 MHz, $CDCl_3$) δ: 7.12 (t, *J* = 7.3 Hz, 2H), 6.69 (t, *J* = 7.3 Hz, 1H), 6.55 (d, *J* = 7.8 Hz, 3H), 4.72 – 4.70 (m, 2H), 4.00 (s, 2H), 3.07 – 3.05 (m, 2H), 2.94 – 2.93 (m, 1H), 2.55 – 2.38 (m, 4H), 1.90 – 1.87 (m, 2H), 1.76 – 1.62 (m, 7H), 0.96 – 0.93 (m, 2H). ^{13}C NMR (100 MHz, $CDCl_3$) δ: 172.9, 166.0, 145.6, 129.0, 117.6, 113.8, 68.3, 44.8, 42.5, 36.1, 29.7, 24.1. LCMS (ESI): m/z calcd for $C_{20}H_{28}ClN_3O_2$; found $[M+H]^+$ 378.24.

2-chloro-N-((1-(1-(phenylamino)cyclohexane-1-carbonyl)piperidin-4-yl)methyl)acetamide **(121) 1080272**

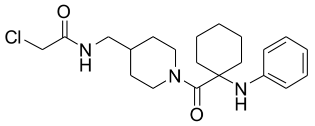

Obtained using procedure B on 0.15 mmol scale, brown oil, 12.7 mg, 22% yield. HPLC retention time 11.5 min. ^{1}H NMR (400 MHz, $CDCl_3$) δ: 7.12 (t, J = 7.7 Hz, 2H), 6.70 (t, J = 7.2 Hz, 1H), 6.54 (d, J = 8.2 Hz, 3H), 5.00 – 4.89 (m, 2H), 4.00 (s, 2H), 3.92 – 3.90 (m, 1H), 3.05 (t, J = 6.0 Hz, 2H), 2.08 – 2.04 (m, 2H), 1.98 – 1.94 (m,2H), 1.70 – 1.62 (m, 6H), 1.44 – 1.25 (m, 5H), 1.05 – 0.79 (m, 2H).^{13}C NMR (100 MHz, $CDCl_3$) δ: 173.3, 165.9, 144.9, 129.1, 117.8, 114.3, 60.0, 44.9, 42.6, 36.2, 32.8, 29.8, 25.1, 21.3. LCMS (ESI): m/z calcd for $C_{21}H_{30}ClN_3O_2$; found $[M+H]^+$ 392.29.

2-chloro-N-((1-(1-(4-chlorophenoxy)cyclohexane-1-carbonyl)piperidin-4-yl)methyl)acetamide **(122) 1080273**

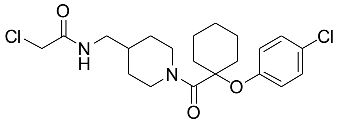

Obtained using procedure B on 0.15 mmol scale, colorless oil, 15.1 mg, 24% yield. HPLC retention time 13.5 min. ^{1}H NMR (400 MHz, $CDCl_3$) δ: 7.18 (d, J = 9.0 Hz, 2H), 6.78 (d, J = 9.0 Hz, 2H), 6.58 (t, J = 5.0 Hz, 1H), 4.72 – 4.69 (m, 2H), 4.02 (s, 2H), 3.10 (td, J = 6.2, 3.1 Hz, 2H), 2.85 (t, J = 12.5 Hz, 1H), 2.53 (t, J = 12.3 Hz, 1H), 2.21 – 2.17 (m, 2H), 1.92 – 1.82 (m, 2H), 1.73 – 1.63 (m, 4H), 1.60 – 1.54 (m, 4H), 1.31 – 1.26 (m, 1H), 1.08 – 1.00 (m, 1H), 0.80 – 0.71 (m, 1H). ^{13}C NMR (100 MHz, $CDCl_3$) δ: 171.8, 166.0, 153.6, 129.2, 126.1, 118.3, 81.9, 45.2, 44.8, 43.0, 42.6, 36.2, 32.5, 32.1, 30.1, 29.5, 25.1, 21.0. LCMS (ESI): m/z calcd for $C_{21}H_{28}Cl_2N_2O_3$; found $[M+H]^+$ 427.20, $[M+Na]^+$ 449.09.

2-chloro-N-((1-(1-((4-chlorophenyl)amino)cyclohexane-1-carbonyl)piperidin-4-yl)methyl)acetamide **(123) 1076403**

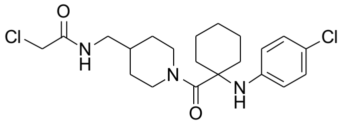

Obtained using procedure B on 0.2 mmol scale, white solid, 43.7 mg, 52% yield, HPLC retention time 12.5 min.^{1}H NMR (400 MHz, $CDCl_3$) δ: 7.06 (d, J = 8.8 Hz, 2H), 6.59 (b, 1H), 6.47 (d, J = 8.8 Hz, 2H), 4.91 – 4.79 (m, 2H), 4.01 (s, 2H), 3.95 (b, 1H), 3.08 (t, J = 5.9 Hz, 2H), 2.87 – 2.86 (m, 1H), 2.61 – 2.56 (m, 1H), 2.04 – 1.93 (m, 4H), 1.70 – 1.62 (m, 6H), 1.40 – 1.25 (m, 3H), 1.01 – 0.89 (m, 2H).^{13}C NMR (100 MHz, $CDCl_3$) δ: 172.9, 166.0, 143.5, 129.0, 122.4, 115.4, 60.1, 44.8, 42.6, 36.2, 32.2, 29.9, 25.1, 21.3. LCMS (ESI): m/z calcd for $C_{21}H_{29}Cl_2N_3O_2$; found $[M+H]^+$ 426.30.

2-Chloro-N-((1-(1-(4-chlorophenoxy)-4,4-difluorocyclohexane-1-carbonyl)piperidin-4-yl)methyl)acetamide **(124) 1075299**

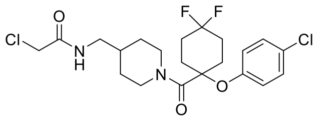

Obtained using procedure B on 0.2 mmol scale, yellow oil, 28.0 mg, 30% yield. ^{1}H NMR (400 MHz, $CDCl_3$) δ: 7.24 – 7.17 (m, 2H), 6.82 – 6.78 (m, 2H), 6.59 (b, 1H), 4.68 (t, J = 11.2 Hz, 2H), 4.02 (s, 2H), 3.11 (dd, J = 11.0, 6.2 Hz, 2H), 2.90 (t, J = 11.9 Hz, 1H), 2.57 (t, J = 11.9 Hz, 1H), 2.30 – 2.16 (m, 4H), 2.09 – 2.00 (m, 4H), 1.76 – 1.69 (m, 2H), 1.59 – 1.56 (m, 1H), 1.10 – 1.06 (m, 1H), 0.80 – 0.75 (m, 1H). ^{13}C NMR (100 MHz, $CDCl_3$) δ: 170.0, 166.0, 153.0, 129.6, 127.0, 118.1, 80.2, 45.3, 44.7, 43.2, 42.6, 36.1, 30.1, 29.5, 29.3, 29.1, 28.8. LCMS (ESI): m/z calcd for $C_{21}H_{26}Cl_2F_2N_2O_3$; found $[M+H]^+$ 463.00.

2-Chloro-N-((1-(1-((4-chlorophenyl)amino)-4,4-difluorocyclohexane-1-carbonyl)piperidin-4-yl)methyl)acetamide **(125) 1075311**

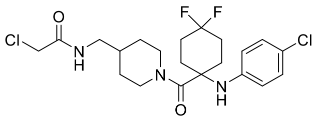

Obtained using procedure B on 0.2 mmol scale, yellow oil, 28.0 mg, 30% yield.^{1}H NMR (400 MHz, $CDCl_3$) δ: 7.13 – 7.07 (m, 2H), 6.58 (b, 1H), 6.55 – 6.49 (m, 2H), 4.85 – 4.80 (m, 2H), 4.02 (s, 2H), 3.76 – 3.75 (m, 1H), 3.09 (t, J = 6.1 Hz, 2H), 2.42 – 2.40 (m, 2H), 2.16 – 2.12 (m, 2H), 2.00 – 1.92 (m, 4H), 1.75 – 1.58 (m, 5H), 1.00 – 0.88 (m, 2H). ^{13}C NMR (100 MHz, $CDCl_3$) δ: 170.9, 166.0, 143.1, 129.3, 123.3, 115.4, 59.2, 44.8, 42.6, 36.1, 30.7, 30.0. LCMS (ESI): m/z calcd for $C_{21}H_{27}Cl_2F_2N_3O_2$; found $[M+H]^+$ 462.10.

2-Chloro-N-((1-(4-(4-chlorophenoxy)tetrahydro-2H-pyran-4-carbonyl)piperidin-4-yl)methyl)acetamide **(126) 1075305**

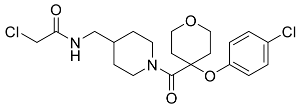

Obtained using procedure B on 0.2 mmol scale, yellow oil, 30.0 mg, 35% yield. ^{1}H NMR (400 MHz, $CDCl_3$) δ: 7.21 – 7.18 (m, 2H), 6.81 – 6.79 (m, 2H), 6.59 (b, 1H), 4.70 – 4.68 (m, 2H), 4.02 (s, 2H), 3.78 – 3.69 (m, 4H), 3.10 (dd, *J* = 11.6, 6.1 Hz, 2H), 2.88 (t, *J* = 12.0 Hz, 1H), 2.56 (t, *J* = 12.1 Hz, 1H), 2.34 – 2.21 (m, 2H), 2.09 – 2.05 (m, 2H), 1.75 – 1.69 (m, 2H), 1.57 – 1.54 (m, 1H), 1.07 – 1.05 (m, 1H), 0.78 – 0.73 (m, 1H). ^{13}C NMR (100 MHz, $CDCl_3$) δ: 170.2, 166.0, 153.2, 129.5, 126.7, 118.2, 79.4, 63.0, 62.9, 45.1, 44.7, 43.1, 42.6, 36.1, 33.0, 32.6, 30.1, 29.5. LCMS (ESI): m/z calcd for $C_{20}H_{26}Cl_2N_2O_4$; found $[M+H]^+$ 429.09.

2-Chloro-N-((1-(4-((4-chlorophenyl)amino)tetrahydro-2H-pyran-4-carbonyl)piperidin-4-yl)methyl)acetamide **(127) 1075310**

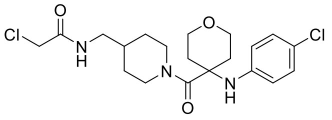

Obtained using procedure B on 0.2 mmol scale, yellow oil, 25.0 mg, 30% yield. ^{1}H NMR (400 MHz, $CDCl_3$) δ: 7.08 (d, *J* = 8.8 Hz, 2H), 6.60 (b, 1H), 6.51 (d, *J* = 8.8 Hz, 2H), 4.80 – 4.78 (m, 2H), 4.00 (s, 2H), 3.82 – 3.80 (m, 2H), 3.72 – 3.67 (m, 2H), 3.10 – 3.08 (m, 2H), 2.87 – 2.62 (m, 2H), 2.38 – 2.37 (m, 2H), 1.83 (d, *J* = 13.7 Hz, 2H), 1.70 – 1.65 (m, 4H), 0.95 – 0.90 (m, 2H). ^{13}C NMR (100 MHz, $CDCl_3$) δ: 171.3, 166.0, 143.0, 129.2, 123.0, 115.4, 63.6, 58.0, 44.8, 42.6, 36.1, 33.9, 29.8. LCMS (ESI): m/z calcd for $C_{20}H_{27}Cl_2N_3O_3$; found $[M+H]^+$ 428.14.

tert-butyl 4-(4-((2-chloroacetamido)methyl)piperidine-1-carbonyl)-4-(4-chlorophenoxy)piperidine-1-carboxylate **(128) 1076395**

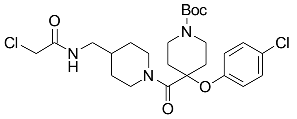

Obtained using procedure B on 0.3 mmol scale, white solid, 48.0 mg, 30% yield. HPLC retention time 14min. ^{1}H NMR (400 MHz, $CDCl_3$) δ: 7.19 (d, *J* = 9.0 Hz, 2H), 6.78 (d, *J* = 8.9 Hz, 2H), 6.61 (b, 1H), 4.67 (d, *J* = 13.1 Hz, 2H), 4.01 (s, 2H), 3.87 – 3.82 (m, 2H), 3.10 – 3.09 (m, 4H), 2.88 (t, *J* = 12.3 Hz, 1H), 2.55 (t, *J* = 12.3 Hz, 1H), 2.14 – 2.13 (m, 4H), 1.74 – 1.68 (m, 2H), 1.59 – 1.54 (m, 1H), 1.43 (s, 9H), 1.07 – 1.04 (m, 1H), 0.76 – 0.75 (m, 1H). ^{13}C NMR (100 MHz, $CDCl_3$) δ: 170.3, 166.0, 154.7, 153.1, 129.5, 126.7, 118.2, 80.1, 79.7, 45.1, 44.7, 43.1, 42.6, 36.1, 30.1, 29.4, 28.4. LCMS (ESI): m/z calcd for $C_{25}H_{35}Cl_2N_3O_5$; found $[M+Na]^+$ 550.24

2-chloro-N-((1-(4-(4-chlorophenoxy)piperidine-4-carbonyl)piperidin-4-yl)methyl)acetamide hydrochloride **(129) 1076397**

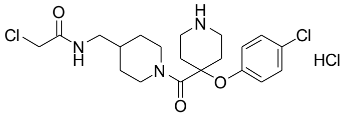

Obtained using procedure C on 0.1 mmol scale, colorless oil, 37.4 mg, 90% yield. ^{1}H NMR (400 MHz, DMSO-d_6) δ: 9.16 (b, 2H), 8.26 (t, *J* = 5.4 Hz, 1H), 7.38 (d, *J* = 8.6 Hz, 2H), 6.89 (d, *J* = 8.6 Hz, 2H), 4.64 – 4.63 (m, 1H), 4.43 (d, *J* = 12.7 Hz, 2H), 4.01 (s, 2H), 3.72 – 3.65 (m, 4H), 3.50 – 3.43 (m, 4H), 3.18 – 3.15 (m, 2H), 2.85 (t, *J* = 5.7 Hz, 2H), 2.25 – 2.24 (m, 2H), 1.63 – 1.61 (m, 1H), 1.50 – 1.47 (m, 1H). ^{13}C NMR (100 MHz, DMSO-d_6) δ: 168.1, 166.0, 152.8, 129.6, 125.9, 118.7, 77.9, 72.2, 70.5, 60.2, 44.6, 43.9, 43.6, 42.6, 35.4, 29.8. LCMS (ESI): m/z calcd for $C_{20}H_{27}Cl_2N_3O_3$; found $[M+H]^+$ 428.35.

tert-butyl 4-(4-((2-chloroacetamido)methyl)piperidine-1-carbonyl)-4-((4-chlorophenyl)amino)piperidine-1-carboxylate **(130) 1075480**

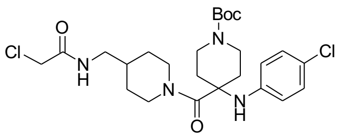

Obtained using procedure B on 0.2 mmol scale, white solid, 32 mg, 30% yield. ^{1}H NMR (400 MHz, $CDCl_3$) δ: 7.09 (d, *J* = 8.9 Hz, 2H), 6.58 (b, 1H), 6.50 (d, *J* = 8.9 Hz, 2H), 4.81 – 4.80 (m, 2H), 4.01 (s, 2H), 3.86 – 3.85 (m, 1H), 3.72 – 3.69 (m, 2H), 3.26 – 3.25 (m, 2H), 3.09 – 3.08 (m, 2H), 2.24 – 2.23 (m, 2H), 1.89 – 1.85 (m, 2H), 1.72 – 1.58 (m, 5H), 1.44 (s, 9H), 1.02 – 0.86 (m, 2H). ^{13}C NMR (100 MHz, $CDCl_3$) δ: 171.3, 166.0, 154.7, 143.0, 129.2, 123.2, 115.4, 79.8, 58.8, 44.8, 42.6, 36.2, 29.7, 28.4. LCMS (ESI): m/z calcd for $C_{25}H_{36}Cl_2N_4O_4$; found $[M+Na]^+$ 549.24.

2-chloro-N-((1-(4-(4-chloro phenylamino)piperidine-4-carbonyl)piperidin-4-yl)methyl)acetamide hydrochloride **(131) 1075481**

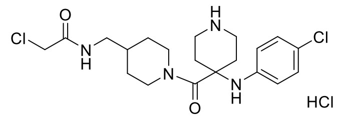

Obtained using procedure C on 0.03 mmol scale, white solid, 11.8 mg, 92% yield. ^{1}H NMR (400 MHz, DMSO-*d$_6$*) δ: 8.99 (b, 2H), 8.24 (t, *J* = 5.3 Hz, 1H), 7.09 (d, *J* = 8.7 Hz, 2H), 6.55 (d, *J* = 8.7 Hz, 2H), 4.56 – 4.42 (m, 2H), 4.00 (s, 2H), 3.71 – 3.65 (m, 4H), 3.49 – 3.42 (m, 4H), 3.32 – 3.29 (m, 2H), 3.00 – 2.97 (m, 2H), 2.82 – 2.80 (m, 2H), 2.30 – 2.26 (m, 1H), 2.05 – 2.01 (m, 1H), 1.56 – 1.54 (m, 2H). ^{13}C NMR (100 MHz, DMSO-*d$_6$*) δ: 170.2, 165.9, 151.1, 144.2, 128.6, 119.9, 114.4, 108.9, 77.9, 72.2, 70.5, 60.2, 56.7, 43.6, 42.6, 35.5, 27.6. LCMS (ESI): m/z calcd for $C_{20}H_{28}Cl_2N_4O_2$; found $[M+H]^+$ 427.14.

tert-butyl 4-(4-((2-chloroacetamido)methyl)piperidine-1-carbonyl)-4-(phenylamino)piperidine-1-carboxylate **(132) 1076396**

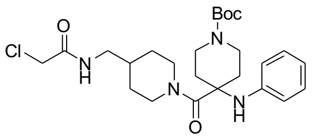

Obtained using procedure B on 0.3 mmol scale, white solid, 51 mg, 33% yield. HPLC retention time 12 min. ^{1}H NMR (400 MHz, $CDCl_3$) δ: 7.12 (t, *J* = 7.9 Hz, 2H), 6.71 (t, *J* = 7.3 Hz, 1H), 6.56 (d, *J* = 7.9 Hz, 3H), 4.84 – 4.83 (m, 2H), 3.99 (s, 2H), 3.90 – 3.89 (m, 1H), 3.72 (d, *J* = 12.7 Hz, 2H), 3.27 – 3.25 (m, 2H), 3.05 – 3.04 (m, 2H), 2.25 – 2.23 (m, 2H), 1.92 – 1.89 (m, 3H), 1.69 – 1.64 (m, 4H), 1.43 (s, 9H), 0.96 – 0.95 (s, 2H).^{13}C NMR (100 MHz, $CDCl_3$) δ: 171.7, 166.0, 154.7, 144.4, 129.3, 118.4, 114.3, 79.7, 58.6, 44.8, 42.6, 36.1, 29.7, 28.4. LCMS (ESI): m/z calcd for $C_{25}H_{37}ClN_4O_4$; found $[M+H]^+$ 493.37, $[M+Na]^+$ 515.28

2-chloro-N-((1-(4-(phenylamino)piperidine-4-carbonyl)piperidin-4-yl)methyl)acetamide hydrochloride **(133) 1076398**

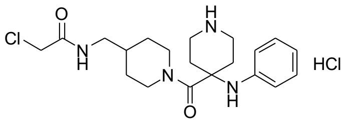

Obtained using procedure C on 0.1 mmol scale, blue oil, 38.8 mg, 98% yield. ^{1}H NMR (400 MHz, DMSO-*d$_6$*) δ: 9.08 (b, 1H), 8.92 (b, 1H), 8.22 (t, *J* = 5.5 Hz, 1H), 7.06 (t, *J* = 7.8 Hz, 2H), 6.56 (t, *J* = 8.3 Hz, 3H), 6.34 (s, 1H), 4.62 (b, 1H), 4.44 (b, 1H), 4.00 (s, 2H), 3.73 – 3.65 (m, 4H), 3.52 – 3.44 (m, 4H), 3.13 – 3.11 (m, 2H), 2.86 – 2.77 (m, 2H), 2.22 – 2.14 (m, 2H), 1.54 – 1.53 (m, 2H), 0.80 – 0.65 (m, 1H). ^{13}C NMR (100 MHz, DMSO-*d$_6$*) δ: 170.6, 165.9, 145.2, 128.8, 116.5, 113.0, 72.2, 70.5, 60.2, 56.6, 43.6, 42.6, 35.5. LCMS (ESI): m/z calcd for $C_{20}H_{29}ClN_4O_2$; found $[M+H]^+$ 393.2.

tert-butyl (4-(4-((2-chloroacetamido)methyl)piperidine-1-carbonyl)-4-(4-chlorophenoxy)cyclohexyl) carbamate **(134) 1076401**

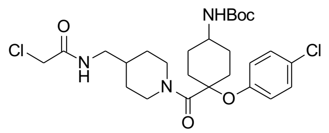

Obtained using procedure B on 0.2 mmol scale, white solid, 34.6 mg, 32% yield. HPLC retention time 14min.^{1}H NMR (400 MHz, $CDCl_3$) δ: 7.18 (d, *J* = 9.0 Hz, 2H), 6.78 (d, *J* = 9.0 Hz, 2H), 6.58 (t, *J* = 5.9 Hz, 1H), 4.68 (d, *J* = 12.6 Hz, 3H), 4.02 (s, 2H), 3.74 (b, 1H), 3.10 (t, *J* = 6.3 Hz, 2H), 2.86 (t, *J* = 12.1 Hz, 1H), 2.54 (t, *J* = 12.1 Hz, 1H), 2.20 – 2.12 (m, 2H), 2.00 – 1.96 (m, 2H), 1.88 – 1.85 (m, 2H), 1.75 – 1.66 (m, 4H), 1.58 – 1.53 (m, 1H), 1.43 (s, 9H), 1.06 – 1.04 (m, 1H), 0.75 – 0.73 (m, 1H).^{13}C NMR (100 MHz, $CDCl_3$) δ: 170.9, 166.0, 155.3, 153.5, 129.4, 126.5, 118.2, 81.3, 79.2, 45.3, 44.7, 43.04, 42.6, 36.1, 30.1, 29.5, 28.8, 28.4, 26.3. LCMS (ESI): m/z calcd for $C_{26}H_{37}Cl_2N_3O_5$; found $[M+Na]^+$ 564.25.

N-((1-(4-amino-1-(4-chlorophenoxy)cyclohexane-1-carbonyl)piperidin-4-yl)methyl)-2-chloroacetamide hydrochloride **(135) 1076402**

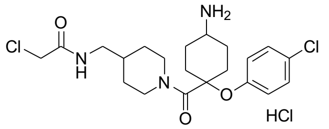

Obtained using procedure C on 0.06 mmol scale, white solid, 23.3 mg, 90% yield. ^{1}H NMR (400 MHz, DMSO-d_6) δ: 8.24 (b, 1H), 8.00 (b, 3H), 7.34 (d, *J* = 8.9 Hz, 2H), 6.85 (d, *J* = 8.9 Hz, 2H), 4.46 (d, *J* = 12.4 Hz, 2H), 4.01 (s, 2H), 3.73 – 3.67 (m, 4H), 3.52 – 3.45 (m, 4H), 2.86 – 2.84 (m, 2H), 2.33 – 2.31 (m, 2H), 1.89 – 1.81 (m, 3H), 1.66 – 1.63 (m, 3H). ^{13}C NMR (100 MHz, DMSO-d_6) δ: 169.0, 166.0, 153.6, 129.4, 125.4, 118.8, 80.7, 72.2, 70.5, 60.2, 43.6, 42.6, 35.5, 29.8. LCMS (ESI): m/z calcd for $C_{21}H_{29}Cl_2N_3O_3$; found [M+H]$^+$ 442.26.

tert-butyl (4-(4-((2-chloroacetamido)methyl)piperidine-1-carbonyl)-4-((4-chlorophenyl)amino) cyclohexyl) carbamate **(136) 1076393**

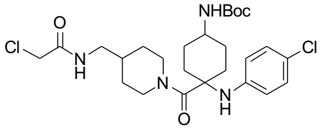

Obtained using procedure B on 0.2 mmol scale, white solid, 37.8 mg, 35% yield. HPLC retention time 14min.^{1}H NMR (400 MHz, $CDCl_3$) δ: 7.06 (d, *J* = 8.8 Hz, 2H), 6.58 (t, *J* = 5.7 Hz, 1H), 6.49 (d, *J* = 8.8 Hz, 2H), 4.82 – 4.80 (m, 2H), 4.60 (d, *J* = 6.7 Hz, 1H), 4.01 (s, 2H), 3.62 (b, 1H), 3.08 (t, *J* = 6.1 Hz, 2H), 2.90 – 2.87 (m, 1H), 2.41– 2.40 (m, 2H), 1.81 – 1.58 (m, 11H), 1.42 (s, 9H), 1.01 – 0.85 (m, 2H). ^{13}C NMR (100 MHz, $CDCl_3$) δ: 171.6, 166.0, 155.3, 143.6, 129.1, 122.8, 115.3,79.2, 59.7, 47.0, 44.8, 42.6, 36.1, 31.8, 30.0, 28.4, 27.9. LCMS (ESI): m/z calcd for $C_{26}H_{38}Cl_2N_4O_4$; found [M+H]$^+$ 541.29, [M+Na]$^+$ 563.25

N-((1-(4-amino-1-((4-chlorophenyl)amino)cyclohexane-1-carbonyl)piperidin-4-yl)methyl)-2-chloroacetamide hydrochloride **(137) 1076394**

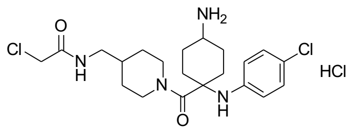

Obtained using procedure C on 0.07 mmol scale, colorless oil, 30.4 mg, 98% yield. ^{1}H NMR (400 MHz, DMSO-d_6) δ: 8.26 (t, *J* = 5.5 Hz, 1H), 8.03 (b, 3H), 7.05 (d, *J* = 8.8 Hz, 2H), 6.54 (d, *J* = 8.8 Hz, 2H), 6.18 (s, 1H), 4.64 – 4.59 (m, 2H), 4.01 (s, 2H), 3.73 – 3.65 (m, 5H), 3.52 – 3.45 (m, 5H), 3.11 – 3.10 (m, 1H), 2.83 – 2.81 (m, 2H), 2.41 – 2.40 (m, 2H), 1.90 - 1.89 (m, 2H), 1.58 – 1.57 (m, 2H), 1.24 – 1.23 (m, 1H), 0.87 – 0.80 (m, 1H). ^{13}C NMR (100 MHz, DMSO-d_6) δ: 170.5, 165.9, 145.1, 128.5, 119.4, 114.5, 72.2, 70.5, 60.2, 58.6, 47.7, 43.6, 42.6, 35.6, 31.6. LCMS (ESI): m/z calcd for $C_{21}H_{30}Cl_2N_4O_2$; found [M+H]$^+$ 441.26

tert-butyl (4-(4-((2-chloroacetamido)methyl)piperidine-1-carbonyl)-4-phenoxycyclohexyl)carbamate **(138) 1076404**

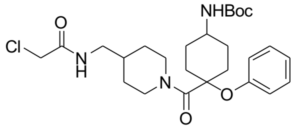

Obtained using procedure B on 0.2 mmol scale, white solid, 36.0 mg, 35% yield. ^{1}H NMR (400 MHz, $CDCl_3$) δ: 7.24 - 7.20 (m, 2H), 6.94 (t, *J* = 7.3 Hz, 1H), 6.83 (d, *J* = 7.9 Hz, 2H), 6.55 (t, *J* = 5.6 Hz, 1H), 4.75 – 4.69 (m, 3H), 4.00 (s, 2H), 3.75 (b, 1H), 3.06 (t, *J* = 6.3 Hz, 2H), 2.86 (t, *J* = 12.2 Hz, 1H), 2.53 (t, *J* = 12.2 Hz, 1H), 2.24 – 2.10 (m, 2H), 2.03 – 2.00 (m, 2H), 1.92 – 1.88 (m, 2H), 1.70 – 1.65 (m, 5H), 1.51 – 1.48 (m, 1H), 1.42 (s, 9H), 1.06 – 1.02 (m, 1H), 0.68 – 0.64 (m, 1H). ^{13}C NMR (100 MHz, $CDCl_3$) δ: 171.3, 165.9, 155.3, 154.9, 129.4, 121.5, 116.9, 80.8, 79.1, 45.6, 45.3, 44.8, 43.0, 42.6, 36.1, 29.9, 29.4, 29.1, 28.7, 28.4, 26.4. LCMS (ESI): m/z calcd for $C_{26}H_{38}ClN_3O_5$; found [M+Na]$^+$ 530.19

N-((1-(4-amino-1-phenoxycyclohexane-1-carbonyl)piperidin-4-yl)methyl)-2-chloroacetamide hydrochloride **(139) 1076405**

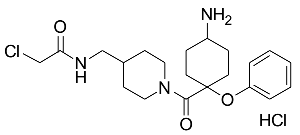

Obtained using procedure C on 0.07 mmol scale, colorless oil, 26.4 mg, 92% yield. ^{1}H NMR (400 MHz, DMSO-d_6) δ: 8.25 (b, 1H), 8.08 (b, 3H), 7.27 (t, *J* = 7.8 Hz, 2H), 6.97 (t, *J* = 7.3 Hz, 1H), 6.83 (d, *J* = 8.1 Hz, 2H), 4.64 – 4.63 (m, 2H), 4.50 – 4.49 (m, 2H), 4.00 (s, 2H), 3.72 – 3.65 (m, 4H), 3.50 – 3.34 (m, 4H), 2.82 (t, *J* = 5.7 Hz, 2H), 2.33 – 2.30 (m, 2H), 1.89 – 1.81 (m, 2H), 1.74 – 1.68 (m, 2H).^{13}C NMR (100 MHz, DMSO-d_6) δ: 169.2, 165.9, 154.8, 129.5, 121.6,

117.1, 80.2, 72.2, 70.5, 60.2, 46.6, 44.0, 43.6, 42.6, 35.5, 29.8. LCMS (ESI): m/z calcd for $C_{21}H_{30}ClN_3O_3$; found $[M+H]^+$ 408.30.

tert-butyl (4-(4-((2-chloroacetamido)methyl)piperidine-1-carbonyl)-4-(phenylamino)cyclohexyl) carbamate **(140) 1076399**

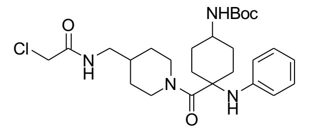

Obtained using procedure B on 0.2 mmol scale, white solid, 30.5 mg, 30% yield. HPLC retention time 12min. ^{1}H NMR (400 MHz, $CDCl_3$) δ: 7.10 (t, *J* = 7.9 Hz, 2H), 6.68 (t, *J* = 7.3 Hz, 1H), 6.55 (d, *J* = 7.9 Hz, 3H), 4.92 – 4.76 (m, 2H), 4.64 (d, *J* = 7.2 Hz, 1H), 3.98 (s, 2H), 3.62 (b, 1H), 3.04 (t, *J* = 6.2 Hz, 2H), 2.88 - 2.87 (m, 1H), 2.40 - 2.39 (m, 3H), 1.81 – 1.80 (m, 2H), 1.66 – 1.64 (m, 7H), 1.43 (s, 9H), 1.03 – 0.77 (m, 3H). ^{13}C NMR (100 MHz, $CDCl_3$) δ: 172.0, 165.9, 155.3, 145.0, 129.2, 118.1, 114.2, 79.1, 59.5, 46.9, 44.8, 42.6, 36.1, 31.8, 29.8, 28.3, 27.8. LCMS (ESI): m/z calcd for $C_{26}H_{39}ClN_4O_4$; found $[M+H]^+$ 507.33, $[M+Na]^+$ 529.29.

N-((1-(4-amino-1-(phenylamino)cyclohexane-1-carbonyl)piperidin-4-yl)methyl)-2-chloroacetamide hydrochloride **(141) 1076400**

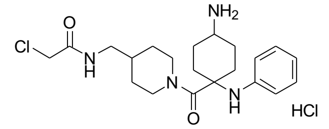

Obtained using procedure C on 0.045 mmol scale, blue oil, 17.4 mg, 92% yield.^{1}H NMR (400 MHz, DMSO-*d₆*) δ: 8.25 (t, *J* = 5.6 Hz, 1H), 8.04 (b, 3H), 7.02 (t, *J* = 7.9 Hz, 2H), 6.56 – 6.50 (m, 3H), 4.00 (s, 2H), 3.71 – 3.68 (m, 4H), 3.52 – 3.45 (m, 4H), 3.10 – 3.09 (m, 1H), 2.79 – 2.78 (m, 2H), 2.41 – 2.40 (m, 2H), 1.91 – 1.89 (m, 2H), 1.76 – 1.70 (m, 2H), 1.57 – 1.54 (s, 4H). ^{13}C NMR (100 MHz, DMSO-d_6) δ: 171.1, 165.9, 146.2, 128.7, 116.1, 113.2, 72.2, 70.5, 60.2, 58.6, 48.0, 43.6, 42.6, 35.6. LCMS (ESI): m/z calcd for $C_{21}H_{31}ClN_4O_2$; found $[M+H]^+$ 407.30

Scheme 12. General procedure for 2C- and 3C-linker chloroacetamide analogs **148 - 153**.[a]

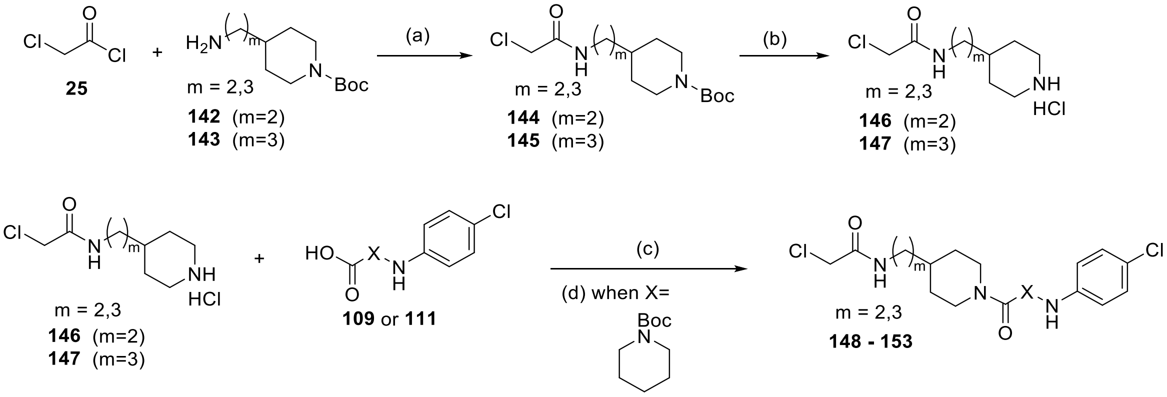

[a] Reagents and conditions: (a) DIPEA, DCM, 0°C to rt, 4h; (b) 4N HCl/dioxane, rt, 3h; (c) HATU, DIPEA, DMF, 0°C to rt, 2h; (d) 4N HCl/dioxane, rt, 3h

Procedure A. *Tert*-butyl 4-(2-aminoethyl)piperidine-1-carboxylate **142** or 1-*boc*-4-(3-aminopropyl)piperidine **143** (1 mmol, 1.0 equiv) was dissolved in 5 ml dry DCM. DIPEA (3 mmol, equiv) was added. The reaction mixture was cooled at 0°C and chloroacetyl chloride **25** (1.2 mmol, 1.2 equiv) was added dropwise. Stirring rt for 4h. The reaction mixture was quenched with sat. $NaHCO_3$ (10 ml) and extracted with DCM (3 x 10 ml). The combined organic phases were dried over $MgSO_4$, filtered and concentrated under reduced pressure. The obtained crude was purified with flash column chromatography (Biotage, hexane - EtOAc, 0-100% EtOAc in hexane).

Procedure B. The boc-protected intermediate **144** or **145** was dissolved in 3 ml HCl/dioxane (4N). Stirring rt for 3h. The solvent was removed under reduced pressure and the obtained HCl salt was used directly in the next step.

Procedure C. The appropriate carboxylic acid **109** or **111** (1 equiv) and HATU (1.2 equiv) were dissolved in 2 ml dry DMF at 0°C. The amine HCl salt **146** or **147** (1.2 equiv) was dissolved in 1 ml DMF, and DIPEA (3 equiv) was added. The solution of the amine was added to the reaction mixture under stirring. Stirring at 0°C for 30 min, then rt for 2h. The reaction mixture was diluted with sat. NH_4Cl (10 ml) and extracted with ethyl acetate (3x10ml). The combined organic phases were washed with Brine, dried over MgSO4, filtered and concentrated under reduced pressure. The obtained crude was purified by HPLC (column C18, H_2O – CH_3CN + 0.05% formic acid, gradient 30-100% CH_3CN in H_2O, 20 min total).

Procedure D. The purified Boc-intermediate was suspended in 3 ml HCl/dioxane (4N) Stirring rt for 3h. The solvent was removed under reduced pressure and the deprotected product was dried under vacuum.

Tert-butyl 4-(2-(2-chloroacetamide)ethyl)piperidine-1-carboxylate **(144)**

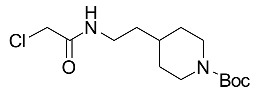

Obtained using procedure A on 1 mmol scale, yellow oil, 237.4 mg, 0.78 mmol, 78% yield. ^{1}H NMR (400 MHz, $CDCl_3$) δ: 6.71 (b, 1H), 4.00 – 3.98 (m, 2H), 3.97 (s, 2H), 3.26 (dd, J = 13.6, 6.6 Hz, 2H), 2.60 (t, J = 12.3 Hz, 2H), 1.60 (d, J = 12.8 Hz, 2H), 1.45 – 1.38 (m, 3H), 1.37 (s, 9H), 1.08 – 1.00 (m, 2H).^{13}C NMR (100 MHz, $CDCl_3$) δ: 165.7, 154.6, 79.1, 43.7, 42.4, 37.2, 33.4, 31.7, 28.2. LCMS (ESI): m/z calcd for $C_{14}H_{25}ClN_2O_3$; found $[M+Na]^+$ 327.18.

Tert-butyl 4-(3-(2-chloroacetamido)propyl)piperidine-1-carboxylate **(145)**

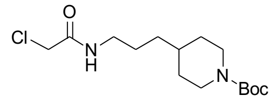

Obtained using procedure A on 1 mmol scale, yellow oil, 280 mg, 88% yield. ^{1}H NMR (400 MHz, $CDCl_3$) δ: 6.68 (b, 1H), 3.99 (s, 4H), 3.27 – 3.21 (m, 2H), 2.62 – 2.60 (m, 2H), 1.61 – 1.50 (m, 4H), 1.40 (s, 9H), 1.37 – 1.32 (m, 1H), 1.25 – 1.21 (m, 2H), 1.06 – 1.00 (m, 2H).^{13}C NMR (100 MHz, $CDCl_3$) δ: 165.8, 154.7, 79.2, 43.8, 42.5, 39.8, 35.5, 33.4, 31.9, 28.3, 26.4. LCMS (ESI): m/z calcd for $C_{15}H_{27}ClN_2O_3$; found $[M+Na]^+$ 341.0.

2-chloro-N-(2-(1-(4-((4-chlorophenyl)amino)tetrahydro-2H-pyran-4-carbonyl)piperidin-4-yl)ethyl)acetamide **(148)**
1080267

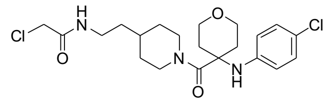

Obtained using procedure C on 0.15 mmol scale, yellow oil, 16.0 mg, 24% yield. HPLC retention time 11.0 min. ^{1}H NMR (400 MHz, $CDCl_3$) δ: 7.07 (d, J = 8.4 Hz, 2H), 6.51 (d, J = 8.3 Hz, 2H), 4.75 – 4.74 (m, 2H), 4.01 (s, 2H), 3.83 – 3.80 (m, 2H), 3.72 – 3.70 (m, 2H), 3.27 – 3.23 (m, 2H), 2.38 – 2.37 (m, 3H), 1.86 – 1.82 (m, 3H), 1.64 – 1.62 (m, 3H), 1.48 – 1.33 (m, 4H), 1.10 - 0.88 (m, 2H).^{13}C NMR (100 MHz, $CDCl_3$) δ: 171.2, 165.8, 143.1, 129.2, 122.9, 115.3, 63.6, 58.0, 42.6, 37.2, 35.8, 34.1, 33.6, 32.1. LCMS (ESI): m/z calcd for $C_{21}H_{29}Cl_2N_3O_3$; found $[M+H]^+$ 442.16.

2-chloro-N-(3-(1-(4-((4-chlorophenyl)amino)tetrahydro-2H-pyran-4-carbonyl)piperidin-4-yl)propyl)acetamide **(149)**
1075478

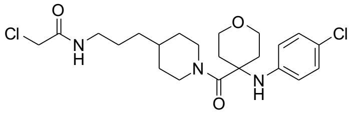

Obtained using procedure C on 0.20 mmol scale, colorless oil, 21.8mg, 24% yield. ^{1}H NMR (400 MHz, $CDCl_3$) δ: 7.06 (d, J = 8.7 Hz, 2H), 6.57 (b, 1H), 6.51 (d, J = 8.7 Hz, 2H), 4.74 – 4.73 (m, 2H), 4.01 (s, 2H), 3.82 – 3.79 (m, 2H), 3.72 – 3.68 (m, 3H), 3.57 (t, J = 6.4 Hz, 1H), 3.22 (dd, J = 13.4, 6.8 Hz, 2H), 2.36 (b, 2H), 1.89 – 1.82 (m, 3H), 1.75 – 1.73 (m, 1H), 1.60 – 1.53 (m, 2H), 1.48 – 1.40 (m, 3H), 1.15 – 1.08 (m, 2H). ^{13}C NMR (100 MHz, $CDCl_3$) δ: 170.9, 170.4, 165.8, 142.7, 129.1, 123.1, 115.6, 78.2, 67.0, 63.6, 62.6, 58.2, 44.8, 42.6, 39.8, 35.6, 33.8, 33.1, 32.2, 29.4, 27.4, 26.2. LCMS (ESI): m/z calcd for $C_{22}H_{31}Cl_2N_3O_3$; found $[M+H]^+$ 456.15, $[M+Na]^+$ 478.10.

tert-butyl 4-(4-(2-(2-chloroacetamido)ethyl)piperidine-1-carbonyl)-4-((4-chlorophenyl)amino) piperidine-1-carboxylate **(150) 1080292**

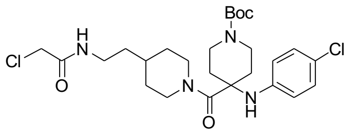

Obtained using procedure C on 0.25 mmol scale, white solid, 31.8 mg, 24% yield. HPLC retention time 13.0 min. ^{1}H NMR (400 MHz, $CDCl_3$) δ: 7.07 (d, *J* = 8.7 Hz, 2H), 6.50 (d, *J* = 8.7 Hz, 2H), 4.75 (b, 2H), 4.01 (s, 2H), 3.72 -3.69 (m, 2H), 3.29 – 3.24 (m, 4H), 2.25 – 2.23 (m, 2H), 1.90 – 1.85 (m, 3H), 1.67 – 1.59 (m, 3H), 1.48 – 1.42 (m, 3H), 1.44 (s, 9H), 1.40 – 1.34 (m, 2H), 1.10 – 0.88 (m, 2H). ^{13}C NMR (100 MHz, $CDCl_3$) δ: 171.3, 165.9, 154.7, 151.3, 143.1, 129.1, 122.9, 121.2, 115.3, 79.8, 58.7, 42.6, 39.9, 37.2, 35.7, 33.6, 32.1, 28.3. LCMS (ESI): m/z calcd for $C_{26}H_{38}Cl_2N_4O_4$; found $[M+H]^+$ 541.29.

2-chloro-N-(2-(1-(4-((4-chlorophenyl)amino)piperidine-4-carbonyl)piperidin-4-yl)ethyl)acetamide hydrochloride **(151) 1080293**

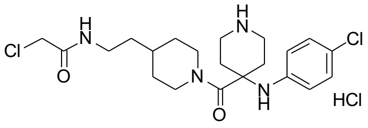

Obtained using procedure D on 0.042 mmol scale, yellow oil, 18.5 mg, 98% yield. ^{1}H NMR (400 MHz, DMSO-d_6) δ: 8.92 (b, 2H), 8.17 (b, 1H), 7.10 (d, *J* = 8.8 Hz, 2H), 6.55 (d, *J* = 8.8 Hz, 2H), 4.54 – 4.46 (m, 2H), 3.99 (s, 2H), 3.73 – 3.65 (m, 4H), 3.52 – 3.44 (m, 4H), 3.03 – 3.01 (m, 2H), 2.83 – 2.81 (m, 1H), 2.28 – 2.24 (m, 1H), 2.11 – 2.02 (m, 2H), 1.60 – 1.58 (m, 1H), 1.40 – 1.38 (m, 1H), 1.18 – 1.15 (m, 2H), 0.77 – 0.60 (m, 2H). ^{13}C NMR (100 MHz, DMSO-d_6) δ: 170.1, 165.6, 144.2, 128.6, 119.9, 114.4, 72.2, 70.5, 60.2, 56.6, 43.6, 42.6, 36.2, 35.3, 32.6. LCMS (ESI): m/z calcd for $C_{21}H_{30}Cl_2N_4O_2$; found $[M+H]^+$ 441.21.

tert-butyl 4-(4-(3-(2-chloroacetamido)propyl)piperidine-1-carbonyl)-4-((4-chlorophenyl)amino) piperidine-1-carboxylate **(152) 1076391**

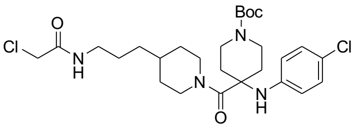

Obtained using procedure C on 0.2 mmol scale, colorless oil, 22.2 mg, 20% yield. HPLC retention time 14min. ^{1}H NMR (400 MHz, $CDCl_3$) δ: 7.08 (d, *J* = 8.8 Hz, 2H), 6.54 (b, 1H), 6.50 (d, *J* = 8.8 Hz, 2H), 4.75 – 4.74 (m, 2H), 4.03 (s, 2H), 3.87 – 3.86 (m, 1H), 3.71 (d, *J* = 14.1 Hz, 2H), 3.27 – 3.21 (m, 4H), 2.26 – 2.25 (m, 2H), 2.01 (b, 1H), 1.87 (d, *J* = 14.0 Hz, 2H), 1.60 – 1.58 (m, 7H), 1.44 (s, 9H), 1.27 – 1.25 (m, 1H), 1.15 – 1.13 (m, 2H). ^{13}C NMR (100 MHz, $CDCl_3$) δ: 171.2, 165.8, 154.7, 143.1, 129.2, 123.0, 115.4, 79.8, 58.7, 42.7, 39.9, 35.6, 33.2, 32.5, 32.1, 28.4, 26.3. LCMS (ESI): m/z calcd for $C_{27}H_{40}Cl_2N_4O_4$; found $[M+Na]^+$ 555.57.

2-chloro-N-(3-(1-(4-((4-chlorophenyl)amino)piperidine-4-carbonyl)piperidin-4-yl)propyl)acetamide hydrochloride **(153) 1076392**

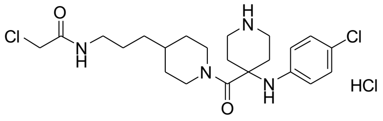

Obtained using procedure D on 0.04 mmol scale, white solid, 15.6 mg, 85% yield. ^{1}H NMR (400 MHz, DMSO-d_6) δ: 9.02 (b, 2H), 8.19 (t, *J* = 5.5 Hz, 1H), 7.08 (d, *J* = 8.9 Hz, 2H), 6.60 (s, 1H), 6.54 (d, *J* = 8.9 Hz, 2H), 4.55 – 4.46 (m, 2H), 4.00 (s, 2H), 3.71– 3.64 (m, 4H), 3.49– 3.44 (m, 4H), 3.01 – 2.96 (m, 2H), 2.83 – 2.80 (m, 1H), 2.12 – 2.10 (m, 2H), 1.58 – 1.54 (m, 2H), 1.34 – 1.26 (m, 2H), 1.00 – 0.98 (m, 2H), 0.78 – 0.75 (m, 1H), 0.60 – 0.55 (s, 1H). ^{13}C NMR (100 MHz, DMSO-d_6) δ: 170.1, 165.7, 144.2, 128.6, 119.9, 114.4, 72.2, 70.5, 60.2, 56.7, 43.6, 42.7, 34.8, 33.0, 25.8. LCMS (ESI): m/z calcd for $C_{22}H_{32}Cl_2N_4O_2$; found $[M+H]^+$ 455.26.

Scheme 13. Synthetic route for vinylsulfonamide analog **155** (**1075479**) [a]

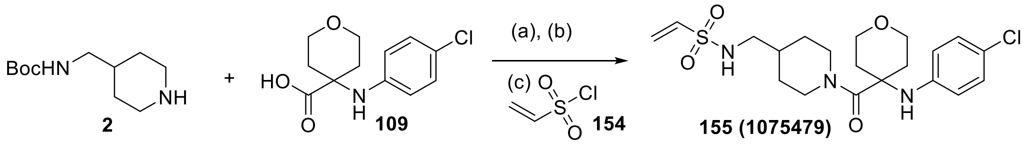

[a] Reagents and conditions: (a) 4-(boc-aminomethyl)piperidine **2**, HATU, DIPEA, DMF, 0°C to rt, 2h; (b) 4N HCl/dioxane, rt, 4h; (c) ethenesulfonyl chloride **154**, DIPEA, DCM, 0°C to rt, 2h.

Procedure A. At 0°C carboxylic acid **109** (1 equiv) and HATU (1.2 equiv) were dissolved in dry DMF (2ml for 1mmol reaction scale). 4-(Boc-aminomethyl)piperidine **2** (1.2 equiv) was dissolved in 1 ml DMF, and DIPEA (3 equiv) was added. The solution of the amine was added to the reaction mixture under stirring. Stirring at 0 °C for 30 min, then rt for 2h. The reaction mixture was diluted with sat. NH_4Cl (10 ml) and extracted with ethyl acetate (3x10ml). The combined organic phases were washed with Brine, dried over MgSO4, filtered and concentrated under reduced pressure. The crude was purified with flash column chromatography (Biotage, hexane - EtOAc, 0-100% EtOAc in hexane). The product was confirmed with LCMS and used directly in the next step.

Procedure B. The boc-protected amine was dissolved in 3ml HCl/dioxane (4N). Stirring rt for 3h. The solvent was removed under reduced pressure and the obtained HCl salt was used directly in the next step.

Procedure C. The HCl salt (1 equiv) was suspended in 2 ml dry DCM. At 0°C, Et_3N (4 equiv) was added. After 10 min, ethenesulfonyl chloride **154** was added slowly (1.2 equiv). Stirring at 0°C for 30min and then at rt for 2h. The reaction mixture was quenched with sat. $NaHCO_3$ (10 ml) and extracted with DCM (3 x 10 ml). The combined organic phases were dried over $MgSO_4$, filtered and concentrated under reduced pressure. The obtained oil was purified with HPLC (column C18, H_2O – CH_3CN + 0.05% formic acid, gradient 30-100% CH_3CN in H_2O, 20 min total).

N-((1-(4-((4-chlorophenyl)amino)tetrahydro-2H-pyran-4-carbonyl)piperidin-4-yl)methyl)ethenesulfonamide **(155) 1075479**

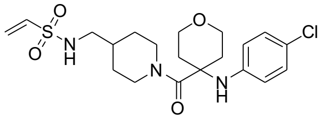

Obtained using procedure C on 0.2 mmol scale, colorless oil, 2.0 mg, 2.3% yield. ^{1}H NMR (400 MHz, $CDCl_3$) δ: 7.08 (d, *J* = 8.8 Hz, 2H), 6.51 (d, *J* = 8.9 Hz, 2H), 6.45 (dd, *J* = 16.5, 9.9 Hz, 1H), 6.22 (d, *J* = 16.5 Hz, 1H), 5.93 (d, *J* = 9.8 Hz, 1H), 4.79 – 4.78 (m, 2H), 4.18 (t, *J* = 6.0 Hz, 1H), 3.82 – 3.81 (m, 2H), 3.73 – 3.68 (m, 2H), 2.75 (t, *J* = 5.9 Hz, 2H), 2.38 – 2.37 (m, 2H), 1.86 – 1.82 (m, 2H), 1.68 – 1.65 (m, 3H), 1.25 – 1.24 (m, 3H), 0.88 – 0.87 (m, 2H). LCMS (ESI): m/z calcd for $C_{20}H_{28}ClN_3O_4S$; found $[M+H]^+$ 442.19.

Scheme 14. Synthetic route for α-chloroketone **156 (1075351)** [a]

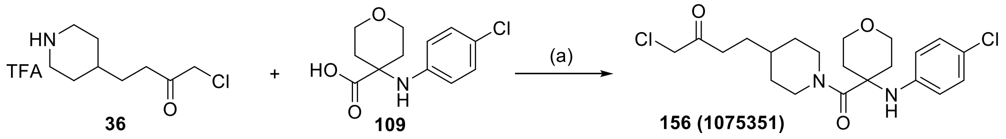

[a] Reagents and conditions: (a) HATU, DIPEA, DMF, 0°C to rt, 2h

Procedure A. At 0°C carboxylic acid **109** (1 equiv) and HATU (1.2 equiv) were dissolved in dry DMF (2ml for 1mmol reaction scale). TFA amine salt **36** (1.2 equiv) was dissolved in 1 ml DMF, and DIPEA (3 equiv) was added. The solution of the amine was added to the reaction mixture under stirring. Stirring at 0 °C for 30 min, then rt for 2h. The reaction

mixture was diluted with sat. NH_4Cl (10 ml) and extracted with ethyl acetate (3x10ml). The combined organic phases were washed with Brine, dried over MgSO4, filtered and concentrated under reduced pressure. The obtained oil was purified with HPLC (column C18, H_2O – CH_3CN + 0.05% formic acid, gradient 30-100% CH_3CN in H_2O, 20 min total).

1-*chloro-4-(1-(4-((4-chlorophenyl)amino)tetrahydro-2H-pyran-4-carbonyl)piperidin-4-yl)butan-2-one* **(156)**
1075351

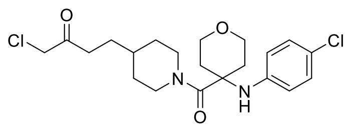

Obtained using procedure A on 0.2 mmol scale, yellow oil, 20.0 mg, 23% yield. ^1H NMR (400 MHz, $CDCl_3$) δ: 7.08 (d, *J* = 7.7 Hz, 2H), 6.52 (d, *J* = 8.1 Hz, 2H), 4.74 – 4.71 (m, 2H), 4.03 (s, 2H), 3.82 – 3.80 (m, 2H), 3.73 – 3.71 (m, 2H), 2.82 – 2.81 (m, 1H), 2.62- 2.61 (m, 1H), 2.52 – 2.50 (m, 2H), 2.39 – 2.37 (m, 2H), 1.84 (d, *J* = 11.1 Hz, 2H), 1.60 – 1.57 (m, 2H), 1.46 – 1.44 (m, 4H), 0.85 – 0.83 (m, 2H). ^{13}C NMR (100 MHz, $CDCl_3$) δ: 202.5, 171.1, 143.1, 129.2, 122.9, 115.4, 63.7, 63.7, 58.0, 48.1, 36.3, 35.2, 34.1, 34.0, 32.0, 31.9, 29.4. LCMS (ESI): m/z calcd for $C_{21}H_{29}Cl_2N_2O_3$; found $[M+H]^+$ 427.14.

Scheme 15. Synthetic route for spiro analog **160 (1080265)** [a]

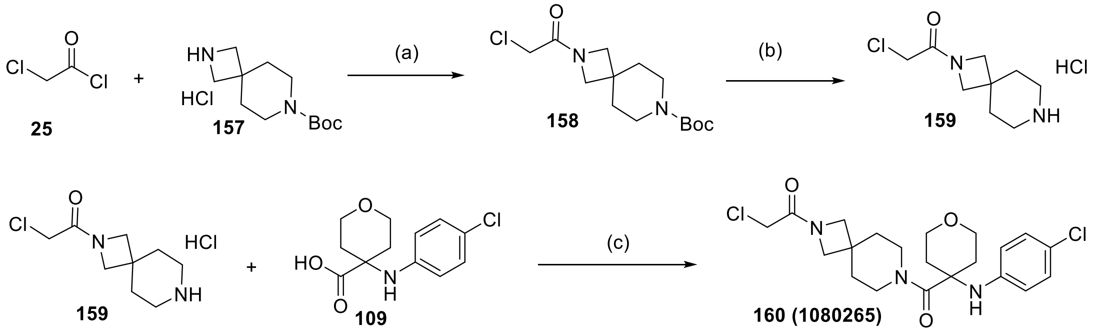

[a] Reagents and conditions: (a) DIPEA, DCM, 0°C to rt, 4h; (b) 4N HCl/dioxane, rt, 3h; (c) HATU, DIPEA, DMF, 0°C to rt, 2h

Procedure A. *Tert*-butyl 2,7-diazaspiro[3.5]nonane-7-carboxylate hydrochloride **157** (1 mmol, 1.0 equiv) was dissolved in 5 ml dry DCM. DIPEA (3 equiv, 3 mmol) was added. The reaction mixture was cooled at 0°C and chloroacetyl chloride **25** (1.2 equiv, 1.2 mmol) was added dropwise. Stirring rt for 4h. The reaction mixture was quenched with sat. $NaHCO_3$ (10 ml) and extracted with DCM (3 x 10 ml). The combined organic phases were dried over $MgSO_4$, filtered and concentrated under reduced pressure. The obtained oil was purified with flash column chromatography [Biotage, hexane – EA, 0-100% EtOAc in hexane].

Procedure B. The residue **158** was suspended in 3 ml HCl/dioxane (4N) for Boc-deprotection. Stirring rt for 3h. The solvent was removed under reduced pressure and the white solid oil was used directly in the next step.

Procedure C. 4-((4-chlorophenyl)amino)tetrahydro-2*H*-pyran-4-carboxylic acid **109** (1 equiv) and HATU (1.2 equiv) were dissolved in 2 ml dry DMF at 0 °C. The amine HCl salt **159** (1.2 equiv) was dissolved in 1 ml DMF, and DIPEA (3 equiv) was added. The solution of the amine was added to the reaction mixture under stirring. Stirring rt for 2h. The reaction mixture was diluted with sat. NH_4Cl (10 ml) and extracted with ethyl acetate (3x10ml). The combined organic phases were washed with Brine, dried over MgSO4, filtered and concentrated under reduced pressure. The obtained crude was purified by HPLC (column C18, H_2O – CH_3CN + 0.05% formic acid, gradient 30-100% CH_3CN in H_2O, 20 min total).

Tert-butyl 2-(2-chloroacetyl)-2,7-diazaspiro[3.5]nonane-7-carboxylate **(158)**

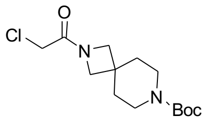

Obtained using procedure A, yellow oil, 105 mg, 35% yield. ^{1}H NMR (400 MHz, $CDCl_3$) δ: 3.93 (s, 2H), 3.86 (s, 2H), 3.73 (s, 2H), 3.34 – 3.28 (m, 4H), 1.69 (t, *J* = 5.5 Hz, 4H), 1.40 (s, 9H). ^{13}C NMR (100 MHz, $CDCl_3$) δ: 166.2, 154.5, 79.7, 60.4, 58.0, 40.5, 39.4, 34.9, 34.1, 28.3. LCMS (ESI): m/z calcd for $C_{14}H_{23}ClN_2O_3$; found $[M+H]^+$ 303.07.

2-chloro-1-(7-(4-((4-chlorophenyl)amino)tetrahydro-2H-pyran-4-carbonyl)-2,7-diazaspiro[3.5]nonan-2-yl)ethan-1-one **(160) 1080265**

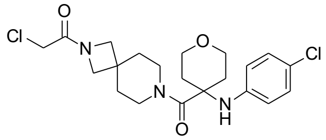

Obtained using procedure C on 0.15 mmol scale, colorless oil, 10.0 mg, 15% yield. HPLC retention time 10.5 min. ^{1}H NMR (400 MHz, $CDCl_3$) δ: 7.08 (d, *J* = 8.9 Hz, 2H), 6.51 (d, *J* = 8.9 Hz, 2H), 3.89 (s, 2H), 3.86 (s, 2H), 3.83 – 3.79 (m, 4H), 3.72 – 3.67 (m, 6H), 2.39 – 2.37 (m, 2H), 1.85 – 1.82 (m, 2H), 1.62 – 1.55 (m, 5H). ^{13}C NMR (100 MHz, $CDCl_3$) δ: 171.6, 166.3, 143.0, 129.3, 123.3, 115.4, 63.6, 60.5, 58.1, 39.4, 35.4, 34.3, 33.9. LCMS (ESI): m/z calcd for $C_{21}H_{27}Cl_2N_3O_3$; found $[M+H]^+$ 440.11.

Scheme 16. Synthetic route for spiro analog **161** (**1080266**) [a]

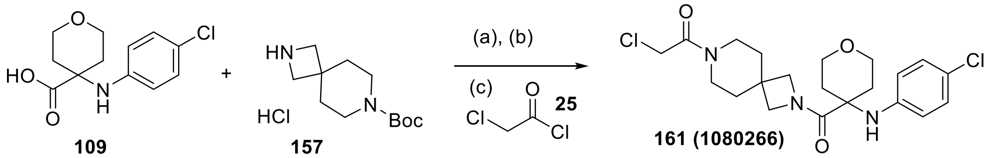

[a] Reagents and conditions: (a) HATU, DIPEA, DMF, 0°C to rt, 2h; (b) 4N HCl/dioxane, rt, 3h; (c) chloroacetyl chloride **25**, DIPEA, DCM, 0°C to rt, 4h

Procedure A. 4-((4-chlorophenyl)amino)tetrahydro-2*H*-pyran-4-carboxylic acid **109** (0.3 mmol, 1equiv) and HATU (0.36 mmol, 1.2 equiv) were dissolved in 1ml DMF. *Tert*-butyl 2,7-diazaspiro[3.5]nonane-7-carboxylate hydrochloride **157** (0.33 mmol. 1.1 equiv) and DIPEA (0.9 mmol, 3 equiv) were dissolved in 2 ml DMF. The solution of the carboxylic acid and HATU was added to the reaction mixture under stirring. Stirring rt for 2h. The reaction mixture was diluted with sat. NH_4Cl (10 ml) and extracted with ethyl acetate (3x10ml). The combined organic phases were washed with Brine, dried over $MgSO_4$, filtered and concentrated under reduced pressure. The obtained crude was purified by flash column chromatography (Biotage, hexane – EA, 0-100% EtOAc in hexane), confirmed with LCMS and was used directly in the next step. The obtained product was suspended in 1.5 ml HCl/dioxane (4N) for Boc-deprotection. Stirring rt for 3h. The solvent was removed under reduced pressure and the obtained salt was used directly in the next step. The HCl salt was suspended in 2 ml dry DCM. DIPEA (4 equiv) was added. The reaction mixture was cooled at 0°C and chloroacetyl chloride **25** (1.2 equiv) was added slowly. Stirring rt for 4h. The reaction mixture was quenched with sat. $NaHCO_3$ (10 ml) and extracted with DCM (3 x 10 ml). The combined organic phases were dried over $MgSO_4$, filtered and concentrated under reduced pressure. The obtained crude was purified by HPLC (column C18, H_2O – CH_3CN + 0.05% formic acid, gradient 30-100% CH_3CN in H_2O, 20 min total).

2-chloro-1-(2-(4-((4-chlorophenyl)amino)tetrahydro-2H-pyran-4-carbonyl)-2,7-diazaspiro[3.5]nonan-7-yl)ethan-1-one **(161) 1080266**

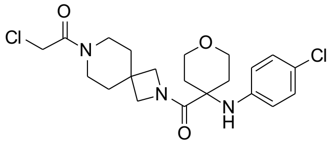

Obtained using procedure A on 0.3 mmol scale, colorless oil, 10.7 mg, 10% yield (over 3 steps). HPLC retention time 10.0 min. ^{1}H NMR (400 MHz, $CDCl_3$) δ: 7.13 (d, *J* = 8.5 Hz, 2H), 6.51 (d, *J* = 8.6 Hz, 2H), 4.02 – 4.00 (m, 2H), 3.95 – 3.92 (m, 2H), 3.86 – 3.83 (m, 2H), 3.75 (s, 2H), 3.68 – 3.62 (m, 2H), 3.56 – 3.52 (m, 1H), 3.36 – 3.32 (m, 3H), 2.34 – 2.27 (m, 2H), 1.78 – 1.74 (m, 2H), 1.64 – 1.56 (m, 5H). ^{13}C NMR (100 MHz, $CDCl_3$) δ: 173.1, 165.1, 142.7, 129.4, 123.4, 115.1, 63.3, 63.1, 62.6, 58.2, 57.1, 43.4, 40.9, 39.3, 35.3, 34.4, 34.1, 32.6, 32.0. LCMS (ESI): m/z calcd for $C_{21}H_{27}Cl_2N_3O_3$; found $[M+H]^+$ 440.16.

Scheme 17. Synthetic route for spiro analog **163** (**1080294**) [a]

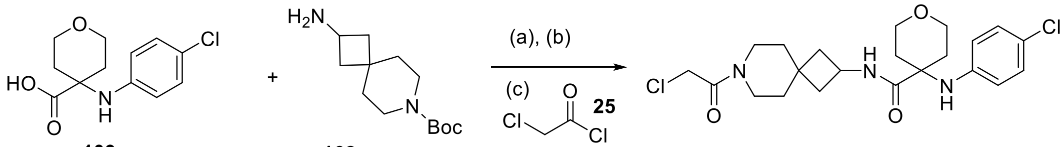

109 **162** **163 (1080294)**

[a] Reagents and conditions: (a) HATU, DIPEA, DMF, 0°C to rt, 2h; (b) 4N HCl/dioxane, rt, 3h; (c) chloroacetyl chloride **25**, DIPEA, DCM, 0°C to rt, 4h

Procedure A. 4-((4-chlorophenyl)amino)tetrahydro-2*H*-pyran-4-carboxylic acid **109** (0.3 mmol, 1equiv) and HATU (0.36 mmol, 1.2 equiv) were dissolved in 1ml DMF. *Tert*-butyl 2-amino-7-azaspiro[3.5]nonane-7-carboxylate **162** (0.33 mmol. 1.1 equiv) and DIPEA (0.9 mmol, 3 equiv) were dissolved in 2ml DMF. The solution of the amine was added to the reaction mixture under stirring. Stirring rt for 2h. The reaction mixture was diluted with sat. NH_4Cl (10 ml) and extracted with ethyl acetate (3x10ml). The combined organic phases were washed with Brine, dried over $MgSO_4$, filtered and concentrated under reduced pressure. The obtained crude was purified by flash column chromatography (Biotage, hexane – EA, 0-100% EtOAc in hexane), confirmed with LCMS and was used directly in the next step. The obtained product was suspended in 1.5 ml HCl/dioxane (4N) for Boc-deprotection. Stirring rt for 3h. The solvent was removed under reduced pressure and the obtained salt was used directly in the next step. The HCl salt was suspended in 2 ml dry DCM. DIPEA (4 equiv) was added. The reaction mixture was cooled at 0°C and chloroacetyl chloride **25** (1.2 equiv) was added slowly. Stirring rt for 4h. The reaction mixture was quenched with sat. $NaHCO_3$ (10 ml) and extracted with DCM (3 x 10 ml). The combined organic phases were dried over $MgSO_4$, filtered and concentrated under reduced pressure. The obtained crude was purified by HPLC (column C18, H_2O – CH_3CN + 0.05% formic acid, gradient 30-100% CH_3CN in H_2O, 20 min total).

N-(7-(2-chloroacetyl)-7-azaspiro[3.5]nonan-2-yl)-4-((4-chlorophenyl)amino)tetrahydro-2H-pyran-4-carboxamide **(163) 1080294**

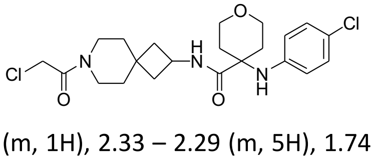

Obtained using procedure A on 0.3 mmol scale, white solid, 18.0 mg, 15% yield (over 3 steps). HPLC retention time 11.5 min. ^{1}H NMR (400 MHz, $CDCl_3$) δ: 7.15 – 7.13 (m, 2H), 7.03 (b, 1H), 6.53 – 6.51 (m, 2H), 4.41 – 4.33 (m, 1H), 4.04 – 4.02 (m, 2H), 3.86 – 3.83 (m, 2H), 3.57 – 3.52 (m, 3H), 3.42 – 3.40 (m, 2H), 3.30 – 3.28 (m, 1H), 2.33 – 2.29 (m, 5H), 1.74 – 1.69 (m, 3H), 1.63 – 1.56 (m, 3H), 1.53 – 1.51 (m, 1H), 1.47 – 1.45 (m, 1H).^{13}C NMR (100 MHz, $CDCl_3$) δ: 173.9, 165.0, 141.9, 129.1, 124.6, 117.1, 67.0, 63.1, 57.8, 43.7, 43.4, 41.0, 39.8, 39.3, 38.6, 36.0, 35.2, 32.5, 32.0. LCMS (ESI): m/z calcd for $C_{22}H_{29}Cl_2N_3O_3$; found $[M+H]^+$ 454.26.

Scheme 18. Synthetic route for spiro analog **166** (**1080295**) [a]

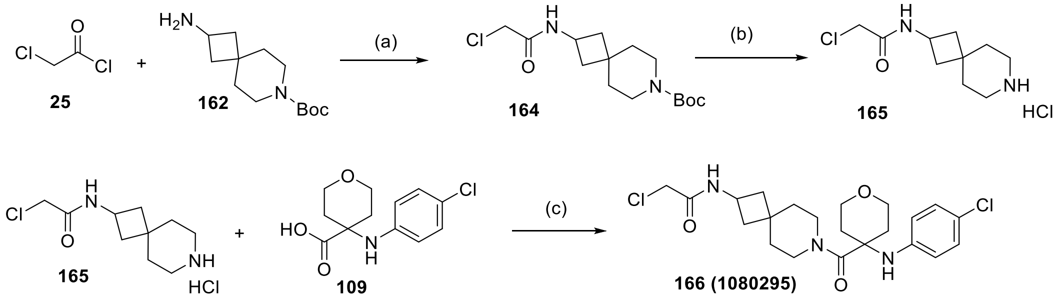

[a] Reagents and conditions: (a) *tert*-butyl 2-amino-7-azaspiro[3.5]nonane-7-carboxylate **162**, chloroacetyl chloride **25**, DIPEA, DCM, 0°C to rt, 4h; (b) 4N HCl/dioxane, rt, 3h; (c) HATU, DIPEA, DMF, 0°C to rt, 2h

Procedure A. *Tert*-Butyl 2-amino-7-azaspiro[3.5]nonane-7-carboxylate **162** (1 mmol, 1 equiv) was dissolved in 5 ml dry DCM. DIPEA (3 mmol, 3 equiv) was added. The reaction mixture was cooled at 0°C and chloroacetyl chloride **25** (1.1 mmol, 1.1 equiv) was added dropwise. Stirring rt for 4h. The reaction mixture was quenched with sat. $NaHCO_3$ (10 ml) and extracted with DCM (3 x 10 ml). The combined organic phases were dried over $MgSO_4$, filtered and concentrated under reduced pressure. The obtained oil was purified with flash column chromatography [Biotage, hexane – EA, 0-100% EtOAc in hexane].

Procedure B. The residue **164** was suspended in 3 ml HCl/dioxane (4N) for Boc-deprotection. Stirring rt for 3h. The solvent was removed under reduced pressure and the white solid oil was used directly in the next step.

Procedure C. 4-((4-chlorophenyl)amino)tetrahydro-2*H*-pyran-4-carboxylic acid **109** (1 equiv) and HATU (1.2 equiv) were dissolved in 2 ml dry DMF at 0 °C. The amine HCl salt **165** (1.1 equiv) was dissolved in 1 ml DMF, and DIPEA (3 equiv) was added. The solution of the amine was added to the reaction mixture under stirring. Stirring rt for 2h. The reaction mixture was diluted with sat. NH_4Cl (10 ml) and extracted with ethyl acetate (3x10ml). The combined organic phases were washed with Brine, dried over $MgSO_4$, filtered and concentrated under reduced pressure. The obtained crude was purified by HPLC (column C18, H_2O – CH_3CN + 0.05% formic acid, gradient 30-100% CH_3CN in H_2O, 20 min total).

Tert-butyl 2-(2-chloroacetamido)-7-azaspiro[3.5]nonane-7-carboxylate **(164)**

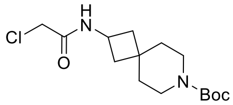

Obtained using procedure A, yellow oil, 311 mg, 98% yield. ^{1}H NMR (400 MHz, $CDCl_3$) δ: 6.95 (b, 1H), 4.26 (dd, *J* = 16.2, 8.1 Hz, 1H), 3.87 (s, 2H), 3.24 – 3.21 (m, 2H), 3.16 – 3.14 (m, 2H), 2.22 – 2.17 (m, 2H), 1.64 – 1.59 (m, 2H), 1.47 – 1.44 (m, 2H), 1.40 – 1.37 (m, 2H), 1.32 (s, 9H). ^{13}C NMR (100 MHz, $CDCl_3$) δ: 165.0, 154.5, 79.0, 42.2, 39.9, 39.4, 38.9, 35.2, 32.1, 28.1. LCMS (ESI): m/z calcd for $C_{15}H_{25}ClN_2O_3$; found $[M-Boc]^+$ 217.21.

2-chloro-N-(7-(4-((4-chlorophenyl)amino)tetrahydro-2H-pyran-4-carbonyl)-7-azaspiro[3.5]nonan-2-yl)acetamide **(166) 1080295**

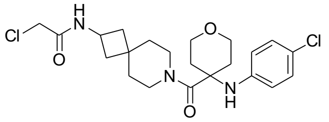

Obtained using procedure C on 0.3 mmol scale, white solid, 25.5 mg, 20% yield. HPLC retention time 11.5 min. ^{1}H NMR (400 MHz, $CDCl_3$) δ: 7.07 (d, *J* = 8.7 Hz, 2H), 6.62 (b, 1H), 6.50 (d, *J* = 8.8 Hz, 2H), 4.35 – 4.29 (m, 1H), 3.99 (s, 2H), 3.83 – 3.78 (m, 3H), 3.73 – 3.64 (m, 3H), 3.60 – 3.48 (m, 2H), 2.39 – 2.33 (m, 2H), 2.28 – 2.23 (m, 2H), 1.84 – 1.81 (m, 2H), 1.65 – 1.60 (m, 2H), 1.48 – 1.30 (m, 5H). ^{13}C NMR (100 MHz, $CDCl_3$) δ: 171.3, 165.1, 143.1, 129.2, 123.0, 115.4, 63.6, 58.0, 42.4, 40.1, 39.9, 35.9, 33.9, 32.6. LCMS (ESI): m/z calcd for $C_{22}H_{29}Cl_2N_3O_3$; found $[M+H]^+$ 454.16.

Scheme 19. Synthetic route for spiro analog **170** (**1080296**) [a]

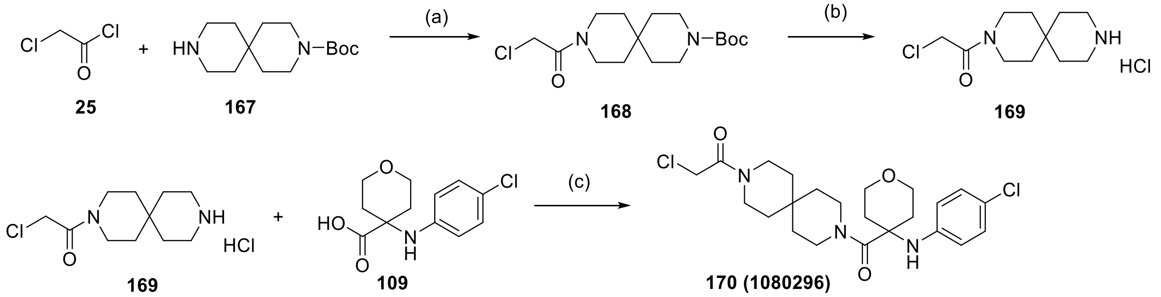

[a] Reagents and conditions: (a) *tert*-Butyl 3,9-diazaspiro[5.5]undecane-3-carboxylate **167**, chloroacetyl chloride **25**, DIPEA, DCM, 0°C to rt, 4h; (b) 4N HCl/dioxane, rt, 3h; (c) HATU, DIPEA, DMF, 0°C to rt, 2h

Procedure A. *Tert*-Butyl 3,9-diazaspiro[5.5]undecane-3-carboxylate **167** (1 mmol, 1 equiv) was dissolved in 5 ml dry DCM. DIPEA (4 mmol, 4 equiv) was added. The reaction mixture was cooled at 0°C and chloroacetyl chloride **25** (1.1 mmol, 1.1 equiv) was added dropwise. Stirring rt for 4h. The reaction mixture was quenched with sat. $NaHCO_3$ (10 ml) and extracted with DCM (3 x 10 ml). The combined organic phases were dried over $MgSO_4$, filtered and concentrated under reduced pressure. The obtained oil was purified with flash column chromatography [Biotage, hexane – EA, 0-100% EtOAc in hexane].

Procedure B. The residue **168** was suspended in 3 ml HCl/dioxane (4N) for Boc-deprotection. Stirring rt for 3h. The solvent was removed under reduced pressure and the white solid oil was used directly in the next step.

Procedure C. 4-((4-chlorophenyl)amino)tetrahydro-2*H*-pyran-4-carboxylic acid **109** (1 equiv) and HATU (1.2 equiv) were dissolved in 2 ml dry DMF at 0 °C. The amine HCl salt **169** (1.1 equiv) was dissolved in 1 ml DMF, and DIPEA (3 equiv) was added. The solution of the amine was added to the reaction mixture under stirring. Stirring rt for 2h. The reaction mixture was diluted with sat. NH_4Cl (10 ml) and extracted with ethyl acetate (3x10ml). The combined organic phases were washed with Brine, dried over $MgSO_4$, filtered and concentrated under reduced pressure. The obtained crude was purified by HPLC (column C18, H_2O – CH_3CN + 0.05% formic acid, gradient 30-100% CH_3CN in H_2O, 20 min total).

Tert-butyl 9-(2-chloroacetyl)-3,9-diazaspiro[5.5]undecane-3-carboxylate **(168)**

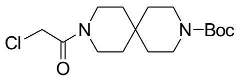

Obtained using procedure A, yellow oil, 204 mg, 62% yield. ^1H NMR (400 MHz, $CDCl_3$) δ: 3.99 (s, 2H), 3.50 – 3.46 (m, 2H), 3.40 -3.37 (m, 2H), 3.32 – 3.26 (m, 4H), 1.49 – 1.47 (m, 2H), 1.43 – 1.38 (m, 6H), 1.36 (s, 9H). ^{13}C NMR (100 MHz, $CDCl_3$) δ: 164.7, 154.6, 79.2, 41.9, 40.9, 38.9, 37.7, 35.5, 34.8, 34.3, 29.9, 28.2. LCMS (ESI): m/z calcd for $C_{16}H_{27}ClN_2O_3$; found $[M\text{-}Boc]^+$ 231.12

2-chloro-1-(9-(4-((4-chlorophenyl)amino)tetrahydro-2H-pyran-4-carbonyl)-3,9-diazaspiro[5.5] undecan-3-yl)ethan-1-one **(170) 1080296**

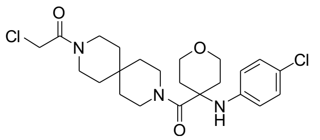

Obtained using procedure C on 0.3 mmol scale, white solid, 12.9 mg, 10% yield. HPLC retention time 11.5 min. ^1H NMR (400 MHz, $CDCl_3$) δ: 7.08 (d, *J* = 8.8 Hz, 2H), 6.52 (d, *J* = 8.8 Hz, 2H), 4.03 (s, 2H), 3.85 – 3.80 (m, 4H), 3.73 – 3.68 (m, 4H), 3.53 – 3.50 (m, 2H), 3.40 – 3.38 (m, 2H), 3.30 – 3.29 (m, 1H), 2.38 – 2.36 (m, 2H), 1.86 – 1.82 (m, 2H), 1.42 – 1.40 (m, 6H), 1.25 – 1.20 (m, 2H). ^{13}C NMR (100 MHz, $CDCl_3$) δ: 171.3, 165.0, 143.1, 129.2, 123.1, 115.3, 63.6, 58.0, 42.1, 41.0, 37.9, 36.1, 35.4, 34.3, 34.0, 30.2. LCMS (ESI): m/z calcd for $C_{23}H_{31}Cl_2N_3O_3$; found $[M+H]^+$ 468.32.

Scheme 20. Synthetic route for spiro analog **174** (**1080297**) [a]

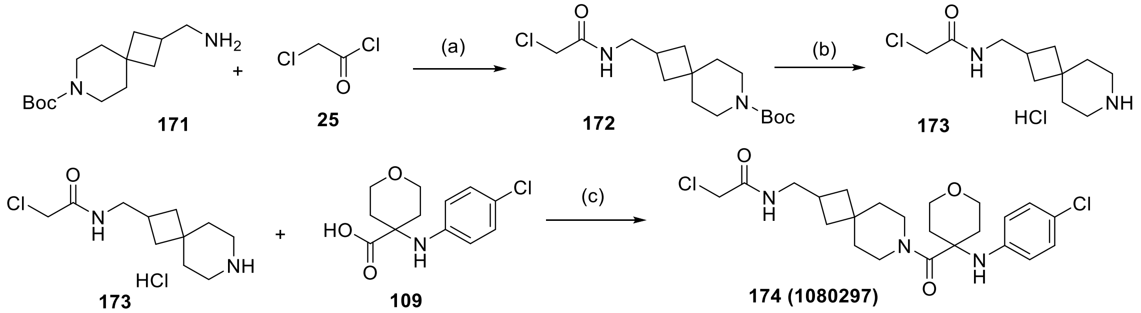

[a] Reagents and conditions: (a) *tert*-butyl 2-(aminomethyl)-7-azaspiro[3.5]nonane-7-carboxylate **171**, chloroacetyl chloride **25**, DIPEA, DCM, 0°C to rt, 4h; (b) 4N HCl/dioxane, rt, 3h; (c) HATU, DIPEA, DMF, 0°C to rt, 2h

Procedure A. *Tert*-butyl 2-(aminomethyl)-7-azaspiro[3.5]nonane-7-carboxylate **(171)** (1 mmol, 1 equiv) was dissolved in 5 ml dry DCM. DIPEA (3 equiv, 3 mmol) was added. The reaction mixture was cooled at 0°C and chloroacetyl chloride **25** (1.1 mmol, 1.1 equiv) was added dropwise. Stirring rt for 4h. The reaction mixture was quenched with sat. $NaHCO_3$ (15 ml) and extracted with DCM (3 x 20 ml). The combined organic phases were dried over $MgSO_4$, filtered and concentrated under reduced pressure. The obtained oil was purified with flash column chromatography [Biotage, hexane – EA, 0-100% EtOAc in hexane].

Procedure B. The residue **172** was suspended in 3 ml HCl/dioxane (4N) for Boc-deprotection. Stirring rt for 3h. The solvent was removed under reduced pressure and the white solid oil was used directly in the next step.

Procedure C. 4-((4-chlorophenyl)amino)tetrahydro-2*H*-pyran-4-carboxylic acid **109** (1 equiv) and HATU (1.2 equiv) were dissolved in 2 ml dry DMF at 0 °C. The amine HCl salt **173** (1.1 equiv) was dissolved in 1 ml DMF, and DIPEA (3 equiv) was added. The solution of the amine was added to the reaction mixture under stirring. Stirring rt for 2h. The reaction mixture was diluted with sat. NH_4Cl (10 ml) and extracted with ethyl acetate (3x10ml). The combined organic phases were washed with Brine, dried over $MgSO_4$, filtered and concentrated under reduced pressure. The obtained crude was purified by HPLC (column C18, H_2O – CH_3CN + 0.05% formic acid, gradient 30-100% CH_3CN in H_2O, 20 min total).

Tert-butyl 2-((2-chloroacetamido)methyl)-7-azaspiro[3.5]nonane-7-carboxylate **(172)**

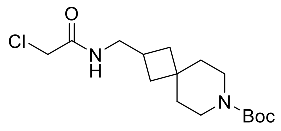

Obtained using procedure A, yellow oil, 246.6 mg, 75% yield. ^1H NMR (400 MHz, $CDCl_3$) δ: 6.74 (s, 1H), 3.93 (s, 2H), 3.24 – 3.20 (m, 4H), 3.16 – 3.14 (m, 2H), 2.40 – 2.36 (m, 1H), 1.87 – 1.71 (m, 2H), 1.46 – 1.35 (m, 6H), 1.33 (s, 9H). ^{13}C NMR (100 MHz, $CDCl_3$) δ: 165.8, 154.6, 79.0, 45.4, 42.4, 40.3, 39.0, 36.1, 35.1, 33.7, 28.2, 27.9. LCMS (ESI): m/z calcd for $C_{16}H_{27}ClN_2O_3$; found $[M+Na]^+$ 353.23

N-((7-(2-chloroacetyl)-7-azaspiro[3.5]nonan-2-yl)methyl)-4-((4-chlorophenyl)amino)tetrahydro-2H-pyran-4-carboxamide **(174) 1080297**

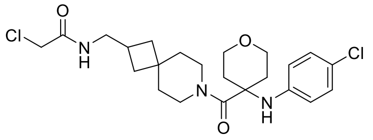

Obtained using procedure C on 0.22 mmol scale, colorless oil, 9.4 mg, 10% yield. HPLC retention time 13.0 min. ^1H NMR (400 MHz, $CDCl_3$) δ: 7.08 – 7.06 (m, 2H), 6.51 – 6.49 (m, 3H), 4.02 (s, 2H), 3.85 – 3.76 (m, 3H), 3.72 – 3.67 (m, 4H), 3.60 – 3.56 (m, 1H), 3.32 – 3.29 (m, 2H), 2.44 – 2.34 (m, 3H), 1.90 – 1.81 (m, 5H), 1.45 – 1.28 (m, 6H). ^{13}C NMR (100 MHz, $CDCl_3$) δ: 171.3, 165.9, 143.1, 129.2, 123.0, 115.4, 63.6, 58.0, 45.5, 42.7, 35.4, 34.2, 34.0, 28.0. LCMS (ESI): m/z calcd for $C_{23}H_{31}Cl_2N_3O_3$; found $[M+H]^+$ 468.22.

Scheme 21. Synthetic route for spiro analog **175** (**1080298**) [a]

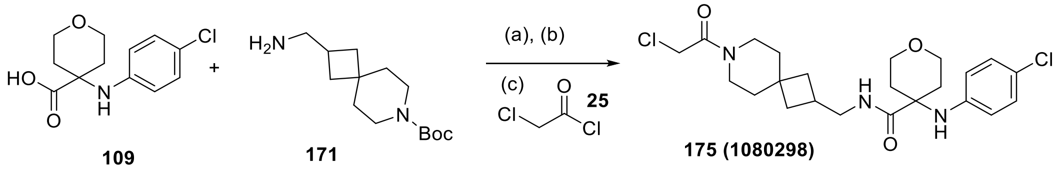

[a] Reagents and conditions: (a) HATU, DIPEA, DMF, 0°C to rt, 2h; (b) 4N HCl/dioxane, rt, 3h; (c) chloroacetyl chloride **25**, DIPEA, DCM, 0°C to rt, 4h

Procedure A. 4-((4-chlorophenyl)amino)tetrahydro-2*H*-pyran-4-carboxylic acid **109** (0.3 mmol, 1equiv) and HATU (0.36 mmol, 1.2 equiv) were dissolved in 1ml DMF. *Ter*t-butyl 2-(aminomethyl)-7-azaspiro[3.5]nonane-7-carboxylate **171** (0.33 mmol. 1.1 equiv) and DIPEA (0.9 mmol, 3 equiv) were dissolved in 2ml DMF. The solution of the amine was added to the reaction mixture under stirring. Stirring rt for 2h. The reaction mixture was diluted with sat. NH_4Cl (10 ml) and extracted with ethyl acetate (3x10ml). The combined organic phases were washed with Brine, dried over $MgSO_4$, filtered and concentrated under reduced pressure. The obtained crude was purified by flash column chromatography (Biotage, hexane – EA, 0-100% EtOAc in hexane), confirmed with LCMS and was used directly in the next step. The obtained product was suspended in 1.5 ml HCl/dioxane (4N) for Boc-deprotection. Stirring rt for 3h. The solvent was removed under reduced pressure and the obtained salt was used directly in the next step. The HCl salt was suspended in 2 ml dry DCM. DIPEA (4 equiv) was added. The reaction mixture was cooled at 0°C and chloroacetyl chloride **25** (1.2 equiv) was added slowly. Stirring rt for 4h. The reaction mixture was quenched with sat. $NaHCO_3$ (10 ml) and extracted with DCM (3 x 10 ml). The combined organic phases were dried over $MgSO_4$, filtered and concentrated under reduced pressure. The obtained crude was purified by HPLC (column C18, H_2O – CH_3CN + 0.05% formic acid, gradient 30-100% CH_3CN in H_2O, 20 min total).

2-chloro-N-(7-(4-((4-chlorophenyl)amino)tetrahydro-2H-pyran-4-carbonyl)-7-azaspiro[3.5]nonan-2-yl)acetamide **(175) 1080298**

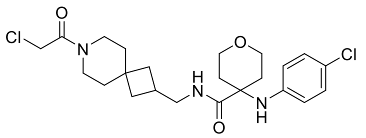

Obtained using procedure A on 0.3 mmol scale, white solid, 23.8 mg, 17% yield (over 3 steps). HPLC retention time 11.5 min. ^{1}H NMR (400 MHz, $CDCl_3$) δ: 7.12 (d, *J* = 8.8 Hz, 2H), 6.93 (b, 1H), 6.53 (d, *J* = 8.8 Hz, 2H), 4.03 (s, 2H), 3.85 (dt, *J* = 12.0, 4.0 Hz, 2H), 3.60 – 3.48 (m, 3H), 3.42 – 3.36 (m, 2H), 3.31 – 3.27 (m, 3H), 2.35 (ddd, *J* = 14.6, 12.3, 6.9 Hz, 3H), 1.86 – 1.79 (m, 2H), 1.78 – 1.72 (m, 3H), 1.64 – 1.62 (m, 1H), 1.57 – 1.55 (m, 1H), 1.51 – 1.49 (m, 1H), 1.46 – 1.41 (m, 3H).^{13}C NMR (100 MHz, $CDCl_3$) δ: 174.5, 164.9, 141.9, 129.1, 124.5, 117.1, 63.1, 58.0, 45.3, 43.3, 41.1, 39.5, 39.1, 36.7, 35.8, 35.3, 34.0, 32.2, 28.3. LCMS (ESI): m/z calcd for $C_{23}H_{31}Cl_2N_3O_3$; found $[M+H]^+$ 468.42.

Scheme 22. Synthetic route for analogs **178** (**1080299**) and **179 (1080300)** [a]

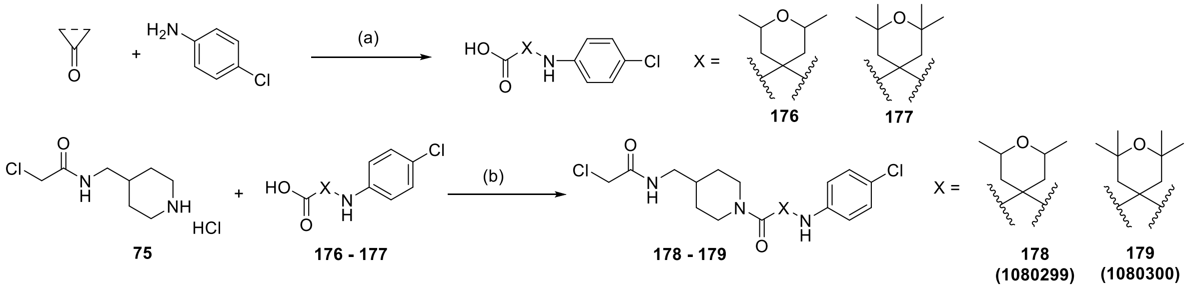

[a] Reagents and conditions: (a) NaOH, $CHCl_3$, THF, 0°C to rt overnight; (b) HATU, DIPEA, DMF, 0°C to rt, 2h

Procedure A. Carboxylic acids **176** and **177** were synthesized using the Bargellini reaction.[17] Under argon: The 4-chloro-aniline (3 mmol, 1 equiv) was dissolved in dry THF (40 ml) and cooled at 0°C. NaOH powder (581 mg, 15 mmol, 5 equiv) and the appropriate ketone (9 mmol, 3 equiv) were added as solids. Dry chloroform (1.16 ml, 15 mmol, 5 equiv) was added dropwise over 30 min at 0°C. The reaction mixture was stirred at 0°C for 1h and then rt overnight. In the cases that solid formation was observed, the solid was filtered off and dissolved in water (50 ml). If solid formation was not observed, the reaction mixture was diluted with water (50 ml). In both cases, the aqua phase was extracted with EtOAc (3 x 50ml). The combined organic phases were separated, and the aqua phase was cooled at 0°C and acidified with 1N acetic acid, until pH=3. The acidified aqua phase was extracted with EtOAc (3 x 50ml). The combined organic phases were dried over $MgSO_4$, filtered and the solvent was removed under reduced pressure. Product formation was confirmed with LCMS. The obtained products were used directly in the next step.

Procedure B. The appropriate carboxylic acid **176** or **177** (1 equiv) and HATU (1.2 equiv) were dissolved in 2 ml dry DMF at 0°C. The amine HCl salt **75** (1.2 equiv) was dissolved in 1 ml DMF, and DIPEA (3 equiv) was added. The solution of the amine was added to the reaction mixture under stirring. Stirring at 0°C for 30 min, then rt for 2h. The reaction mixture was diluted with sat. NH_4Cl (10 ml) and extracted with ethyl acetate (3x10ml). The combined organic phases were washed with Brine, dried over MgSO4, filtered and concentrated under reduced pressure. The obtained crude was purified by HPLC (column C18, H_2O – CH_3CN + 0.05% formic acid, gradient 30-100% CH_3CN in H_2O, 20 min total).

2-chloro-N-((1-(4-((4-chlorophenyl)amino)-2,6-dimethyltetrahydro-2H-pyran-4-carbonyl)piperidin-4-yl)methyl)acetamide **(178) 1080299**

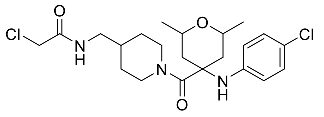

Obtained using procedure B on 0.11 mmol scale, colorless oil, 4.8 mg, 10% yield. HPLC retention time 10.0 min. ^{1}H NMR (400 MHz, $CDCl_3$) δ: 7.07 (d, *J* = 8.4 Hz, 2H), 6.57 (b, 1H), 6.52 (d, *J* = 8.4 Hz, 2H), 4.82 – 4.80 (m, 2H), 4.01 (s, 2H), 3.86 – 3.84 (m, 2H), 3.15 – 3.09 (m, 2H), 2.60 – 2.55 (m, 4H), 1.72 – 1.69 (m, 4H), 1.18 (s, 6H), 0.90 – 0.85 (m, 4H). ^{13}C NMR (100 MHz, $CDCl_3$) δ: 171.0, 166.0, 143.6, 129.2, 123.1, 115.7, 69.9, 66.6, 59.7, 44.8, 43.7, 42.6, 36.2, 29.9, 22.0. LCMS (ESI): m/z calcd for $C_{22}H_{31}Cl_2N_3O_3$; found $[M+H]^+$ 456.26.

2-chloro-N-((1-(4-((4-chlorophenyl)amino)-2,2,6,6-tetramethyltetrahydro-2H-pyran-4-carbonyl)piperidin-4-yl)methyl)acetamide **(179) 1080300**

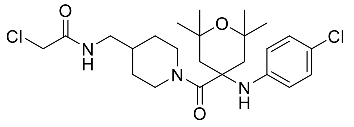

Obtained using procedure B on 0.2 mmol scale, off-white solid, 12.2 mg, 13% yield. HPLC retention time 11.0 min. ^{1}H NMR (400 MHz, $CDCl_3$) δ: 7.08 (d, *J* = 8.9 Hz, 2H), 6.57 (b, 1H), 6.44 (d, *J* = 8.9 Hz, 2H), 4.76 – 4.64 (m, 2H), 4.01 (s, 2H), 3.08 – 3.06 (m, 2H), 2.36 – 2.34 (m, 2H), 2.06 – 2.02 (m, 2H), 1.75 – 1.52 (m, 3H), 1.48 – 1.37 (m, 3H), 1.27 (s, 12H), 0.94 – 0.92 (m, 2H). ^{13}C NMR (100 MHz, $CDCl_3$) δ: 172.2, 166.0, 143.6, 129.2, 122.7, 115.1, 71.3, 70.8, 59.3, 44.8, 42.6, 36.2, 32.7, 29.7. LCMS (ESI): m/z calcd for $C_{24}H_{35}Cl_2N_3O_3$; found $[M+H]^+$ 484.32.

Scheme 23. Synthetic route for spiro analog **180** (**1083743**) [a]

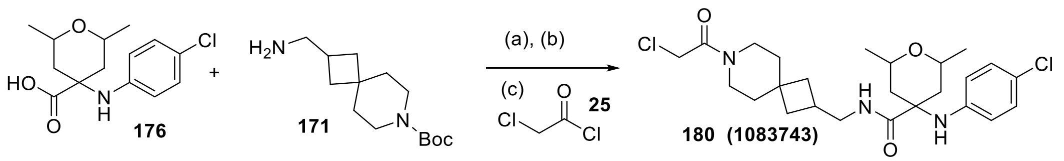

[a] Reagents and conditions: (a) HATU, DIPEA, DMF, 0°C to rt, 2h; (b) 4N HCl/dioxane, rt, 3h; (c) chloroacetyl chloride **25**, DIPEA, DCM, 0°C to rt, 4h

Procedure A. 4-((4-chlorophenyl)amino)-2,6-dimethyltetrahydro-2*H*-pyran-4-carboxylic acid **176** (0.41 mmol, 1equiv) and HATU (0.49 mmol, 1.2 equiv) were dissolved in 2ml DMF. *Ter*t-butyl 2-(aminomethyl)-7-azaspiro[3.5]nonane-7-carboxylate **171** (0.45 mmol. 1.1 equiv) and DIPEA (1.23 mmol, 3 equiv) were dissolved in 3ml DMF. The solution of the amine was added to the reaction mixture under stirring. Stirring rt for 2h. The reaction mixture was diluted with sat. NH_4Cl (20 ml) and extracted with ethyl acetate (3x20ml). The combined organic phases were washed with Brine, dried over $MgSO_4$, filtered and concentrated under reduced pressure. The obtained crude was purified by flash column chromatography (Biotage, hexane – EA, 0-100% EtOAc in hexane), confirmed with LCMS and was used directly in the next step. The obtained product was suspended in 2.0 ml HCl/dioxane (4N) for Boc-deprotection. Stirring rt for 3h. The solvent was removed under reduced pressure and the obtained salt was used directly in the next step. The HCl salt was suspended in 2 ml dry DCM. DIPEA (4 equiv, 50 µl) was added. The reaction mixture was cooled at 0°C and chloroacetyl chloride **25** (1.2 equiv, 20 µl) was added slowly. Stirring rt for 4h. The reaction mixture was quenched with sat. $NaHCO_3$ (10 ml) and extracted with DCM (3 x 10 ml). The combined organic phases were dried over $MgSO_4$, filtered and concentrated under reduced pressure. The obtained crude was purified by HPLC (column C18, H_2O – CH_3CN + 0.05% formic acid, gradient 30-100% CH_3CN in H_2O, 20 min total).

N-((7-(2-chloroacetyl)-7-azaspiro[3.5]nonan-2-yl)methyl)-4-((4-chlorophenyl)amino)-2,6-dimethyltetrahydro-2H-pyran-4-carboxamide **(180) 1083743**

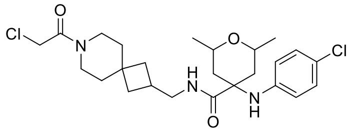

Obtained using the procedure A on 0.41 mmol scale, yellow solid, 32.2 mg, 18% yield (over 3 steps). HPLC retention time 12.0 min. ^{1}H NMR (400 MHz, $CDCl_3$) δ: 7.09 (d, *J* = 8.8 Hz, 2H), 6.92 – 6.90 (m, 1H), 6.52 (d, *J* = 8.8 Hz, 2H), 4.04 – 3.99 (m, 3H), 3.91 – 3.80 (m, 1H), 3.55 (t, *J* = 6.5 Hz, 1H), 3.50 – 3.47 (m, 1H), 3.41 – 3.30 (m, 2H), 3.29 – 3.26 (m, 3H), 2.40 – 2.34 (m, 1H), 2.28 – 2.25 (m, 2H), 1.94 – 1.91 (m, 1H), 1.83 – 1.78 (m, 2H), 1.69 – 1.67 (m, 1H), 1.63 – 1.60 (m, 1H), 1.55 – 1.46 (m, 3H), 1.44 – 1.39 (m, 2H), 1.16 (s, 3H), 1.15 (s, 3H). ^{13}C NMR (100 MHz, $CDCl_3$) δ: 174.7, 164.9, 142.3, 129.0, 124.5, 117.1, 78.0, 69.8, 62.0, 59.0, 45.1, 43.3, 41.3, 41.0, 39.4, 38.7, 36.7, 35.8, 35.3, 33.9, 29.4, 28.3, 27.4, 21.9. LCMS (ESI): m/z calcd for $C_{25}H_{35}Cl_2N_3O_3$; found $[M+H]^+$ 496.23.

Scheme 24. Synthetic route for spiro analog **181** (**1083744**) [a]

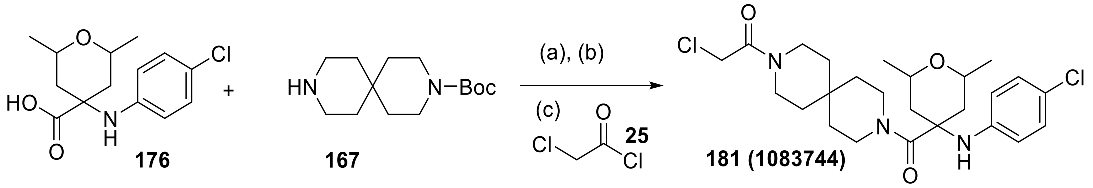

[a] Reagents and conditions: (a) HATU, DIPEA, DMF, 0°C to rt, 2h; (b) 4N HCl/dioxane, rt, 3h; (c) chloroacetyl chloride **25**, DIPEA, DCM, 0°C to rt, 4h

Procedure A. 4-((4-chlorophenyl)amino)-2,6-dimethyltetrahydro-2*H*-pyran-4-carboxylic acid **176** (0.41 mmol, 1equiv) and HATU (0.49 mmol, 1.2 equiv) were dissolved in 2ml DMF. *Tert*-Butyl 3,9-diazaspiro[5.5]undecane-3-carboxylate **167** (0.45 mmol. 1.1 equiv) and DIPEA (1.23 mmol, 3 equiv) were dissolved in 3ml DMF. The solution of the amine was added to the reaction mixture under stirring. Stirring rt for 2h. The reaction mixture was diluted with sat. NH_4Cl (20 ml) and extracted with ethyl acetate (3x20ml). The combined organic phases were washed with Brine, dried over $MgSO_4$, filtered and concentrated under reduced pressure. The obtained crude was purified by flash column chromatography (Biotage, hexane – EA, 0-100% EtOAc in hexane), confirmed with LCMS and was used directly in the next step. The obtained product was suspended in 2.0 ml HCl/dioxane (4N) for Boc-deprotection. Stirring rt for 3h. The solvent was removed under reduced pressure and the obtained salt was used directly in the next step. The HCl salt was suspended in 2 ml dry DCM. DIPEA (4 equiv, 50 µl) was added. The reaction mixture was

cooled at 0°C and chloroacetyl chloride **25** (1.2 equiv, 20 µl) was added slowly. Stirring rt for 4h. The reaction mixture was quenched with sat. $NaHCO_3$ (10 ml) and extracted with DCM (3 x 10 ml). The combined organic phases were dried over $MgSO_4$, filtered and concentrated under reduced pressure. The obtained crude was purified by HPLC (column C18, H_2O – CH_3CN + 0.05% formic acid, gradient 30-100% CH_3CN in H_2O, 20 min total).

2-chloro-1-(9-(4-((4-chlorophenyl)amino)-2,6-dimethyltetrahydro-2H-pyran-4-carbonyl)-3,9-diazaspiro[5.5]undecan-3-yl)ethan-1-one (**181) 1083744**

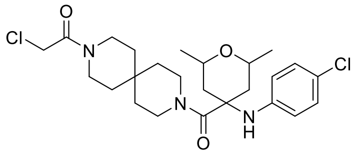

Obtained using procedure A on 0.41 mmol scale, off-white solid, 47.0 mg, 24% yield (over 3 steps). HPLC retention time 11.0 min. ^{1}H NMR (400 MHz, $CDCl_3$) δ: 7.05 (d, J = 8.9 Hz, 2H), 6.51 (d, J = 8.9 Hz, 2H), 4.01 (s, 2H), 3.85 – 3.80 (m, 3H), 3.68 – 3.64 (m, 2H), 3.58 – 3.52 (m, 2H), 3.50 – 3.45 (m, 1H), 3.37 – 3.32 (m, 2H), 2.52 – 2.50 (m, 2H), 2.23 – 2.20 (m, 1H), 1.83 – 1.78 (m, 1H), 1.69 – 1.65 (m, 1H), 1.58 – 1.48 (m, 3H), 1.44 – 1.36 (m, 4H), 1.27 – 1.24 (m, 1H), 1.17 (s, 3H), 1.16 (s, 3H). ^{13}C NMR (100 MHz, $CDCl_3$) δ: 171.0, 170.4, 164.9, 143.7, 129.1, 123.0, 115.5, 79.6, 70.3, 70.0, 62.1, 59.6, 44.8, 43.6, 42.0, 41.7, 41.0, 37.9, 36.1, 34.2, 30.2, 29.3, 27.3, 21.9. LCMS (ESI): m/z calcd for $C_{25}H_{35}Cl_2N_3O_3$; found $[M+H]^+$ 496.28.

5. REPRESENTATIVE NMR SPECTRA

N-((1-(2-(4-chlorophenoxy)-2-methylpropanoyl)piperidin-4-yl)methyl)ethenesulfonamide **(17) 1074199**

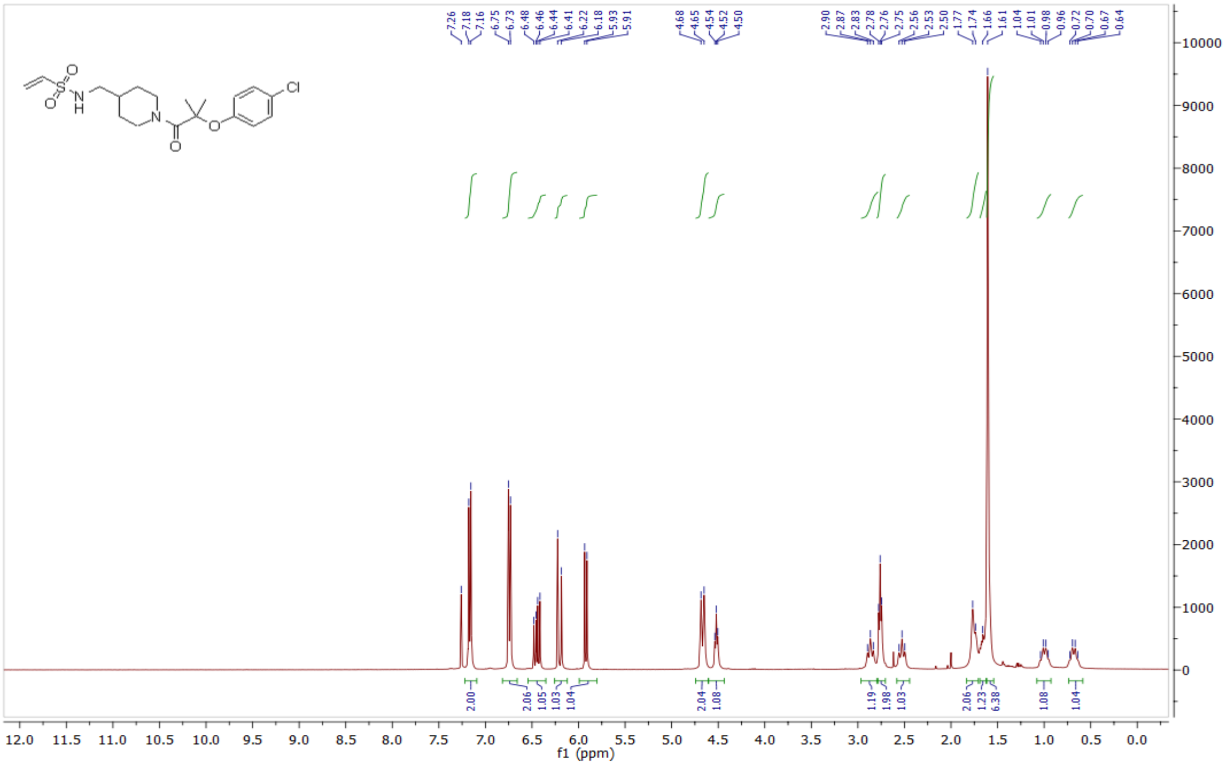

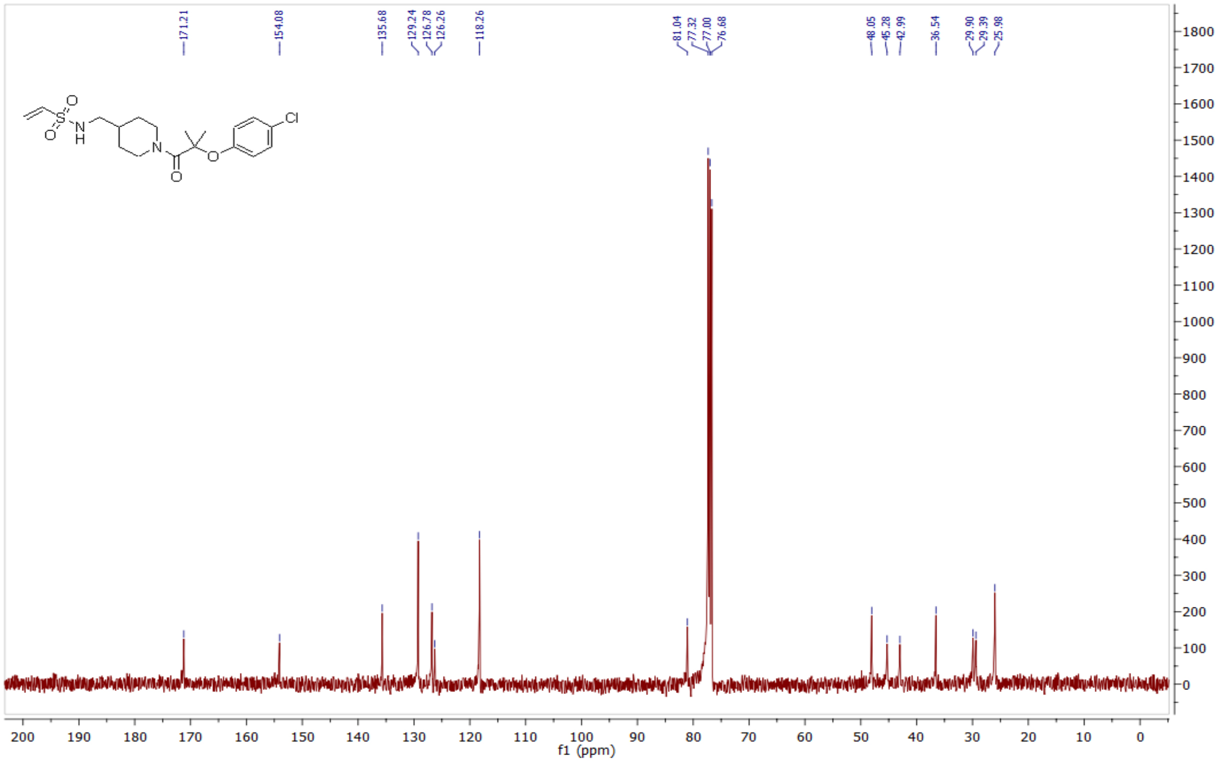

2-chloro-N-(1-(2-(4-chlorophenoxy)-2-methylpropanoyl)piperidin-4-yl)acetamide **(26) 1074360**

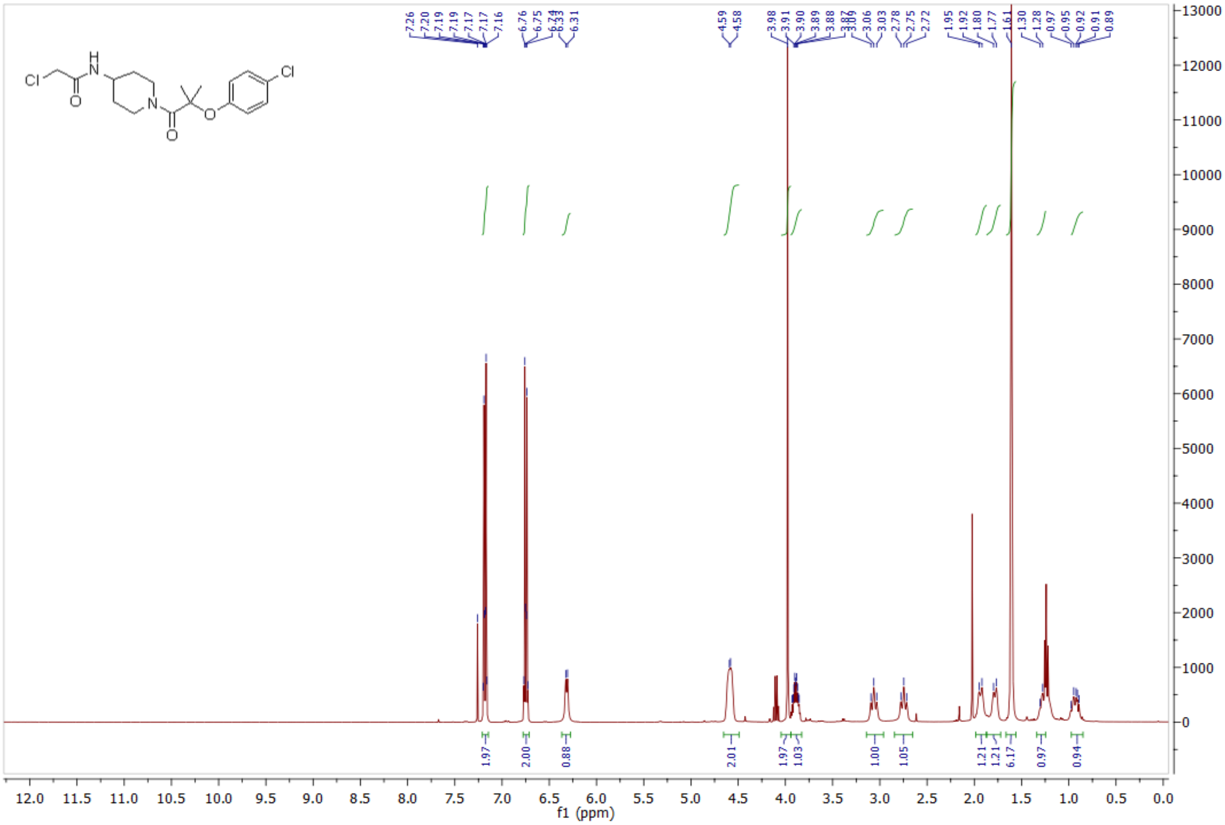

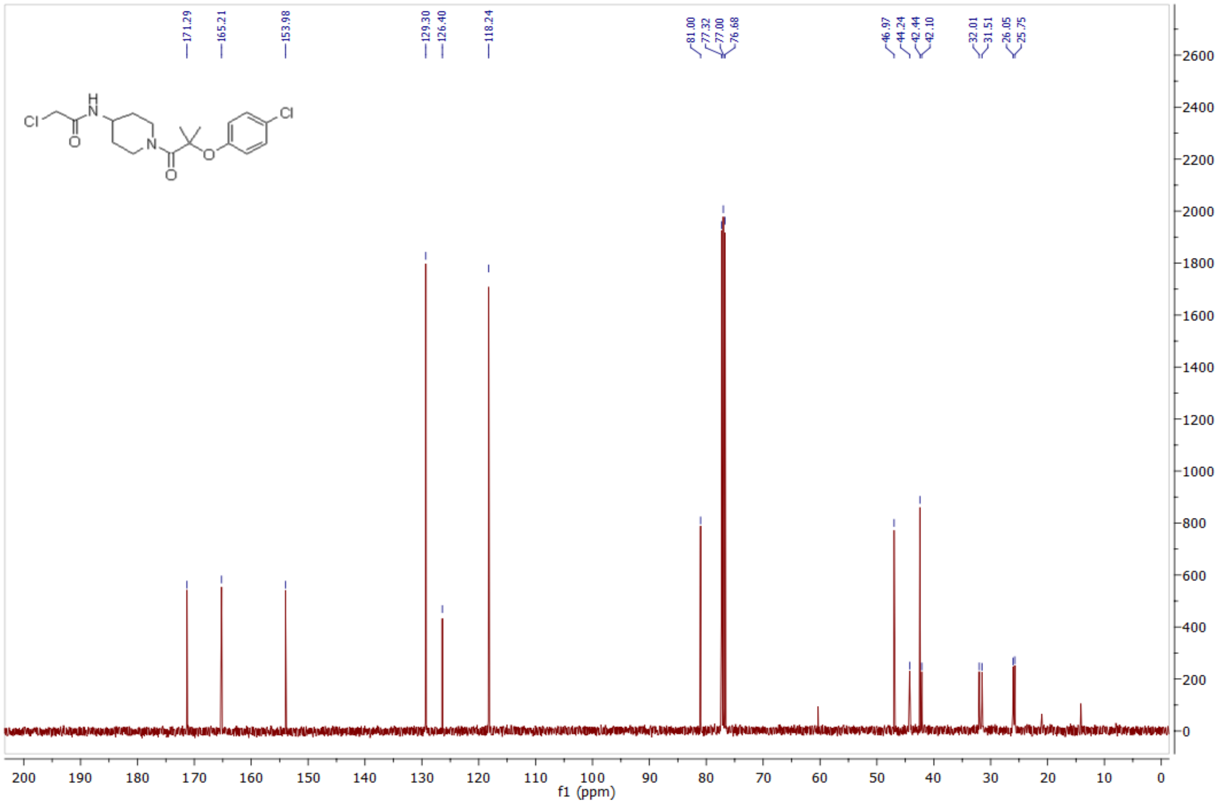

2-chloro-N-(1-(2-(4-chlorophenoxy)-2-methylpropanoyl)piperidin-4-yl)acetamide **(27) 1074202**

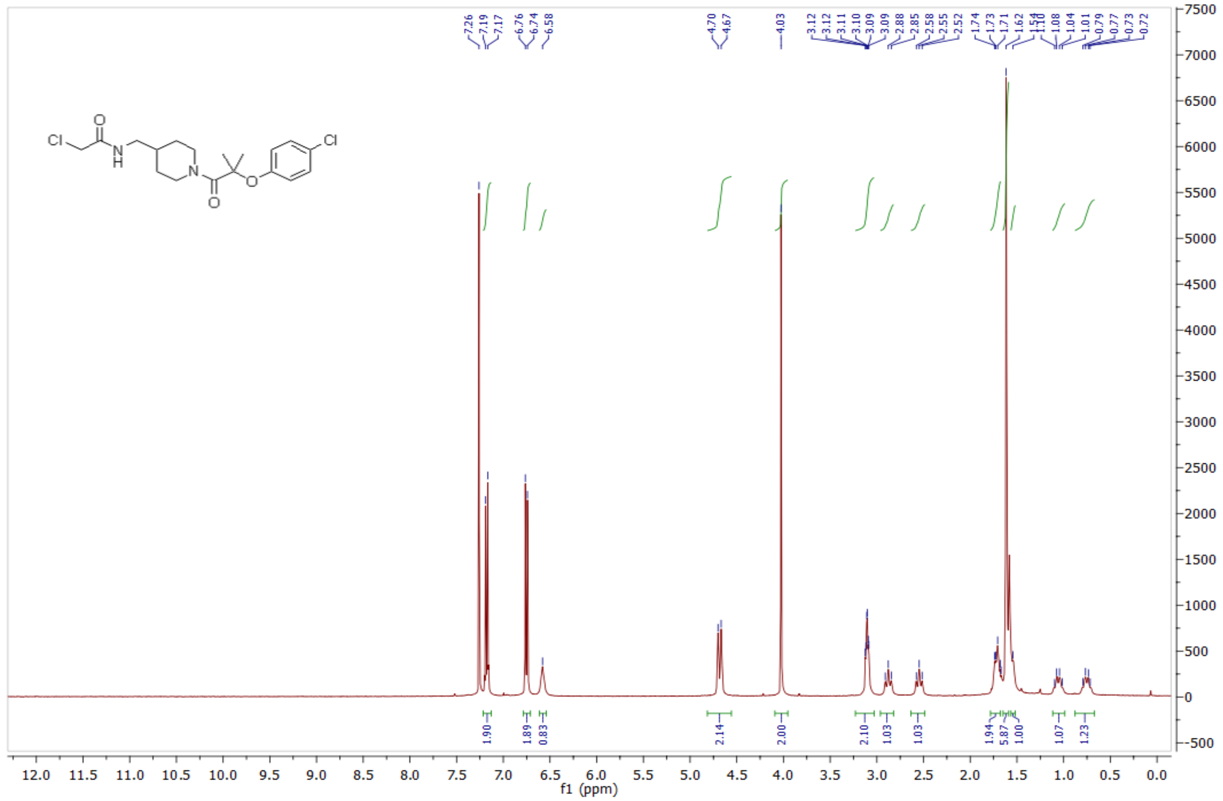

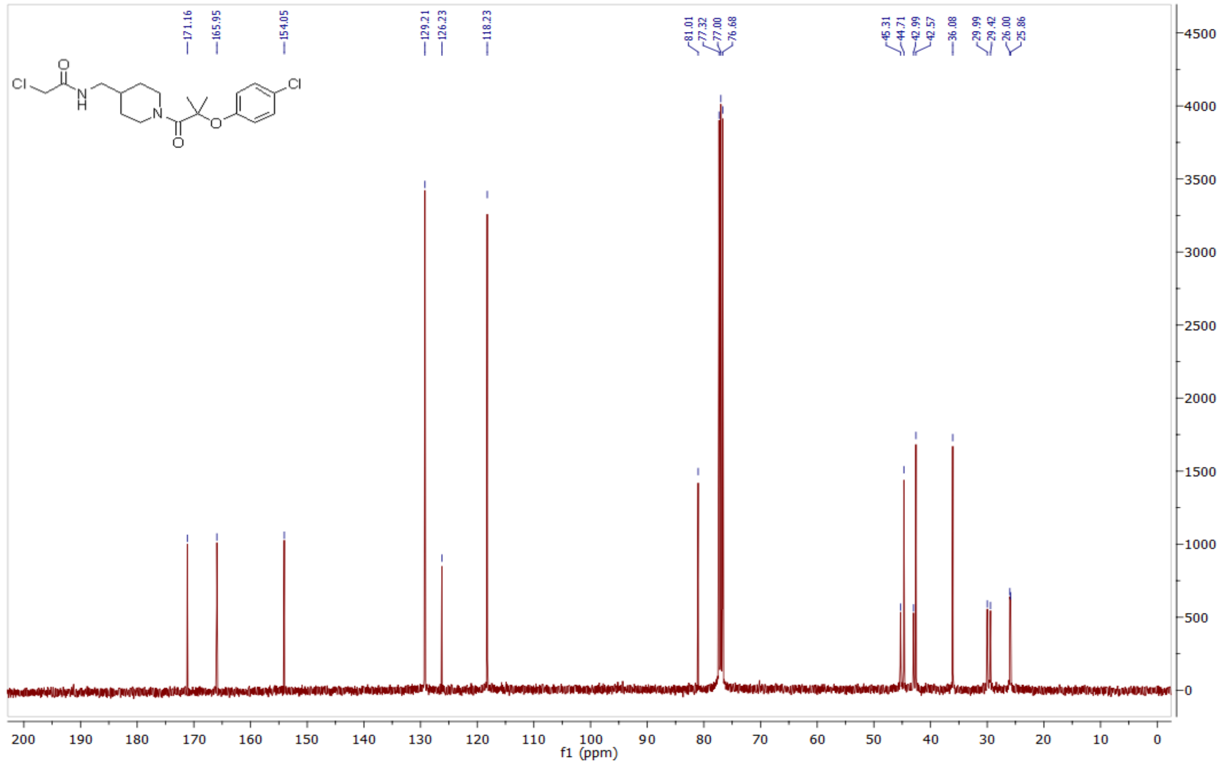

2-chloro-N-(2-(1-(2-(4-chlorophenoxy)-2-methylpropanoyl)piperidin-4-yl)ethyl)acetamide **(28) 1074210**

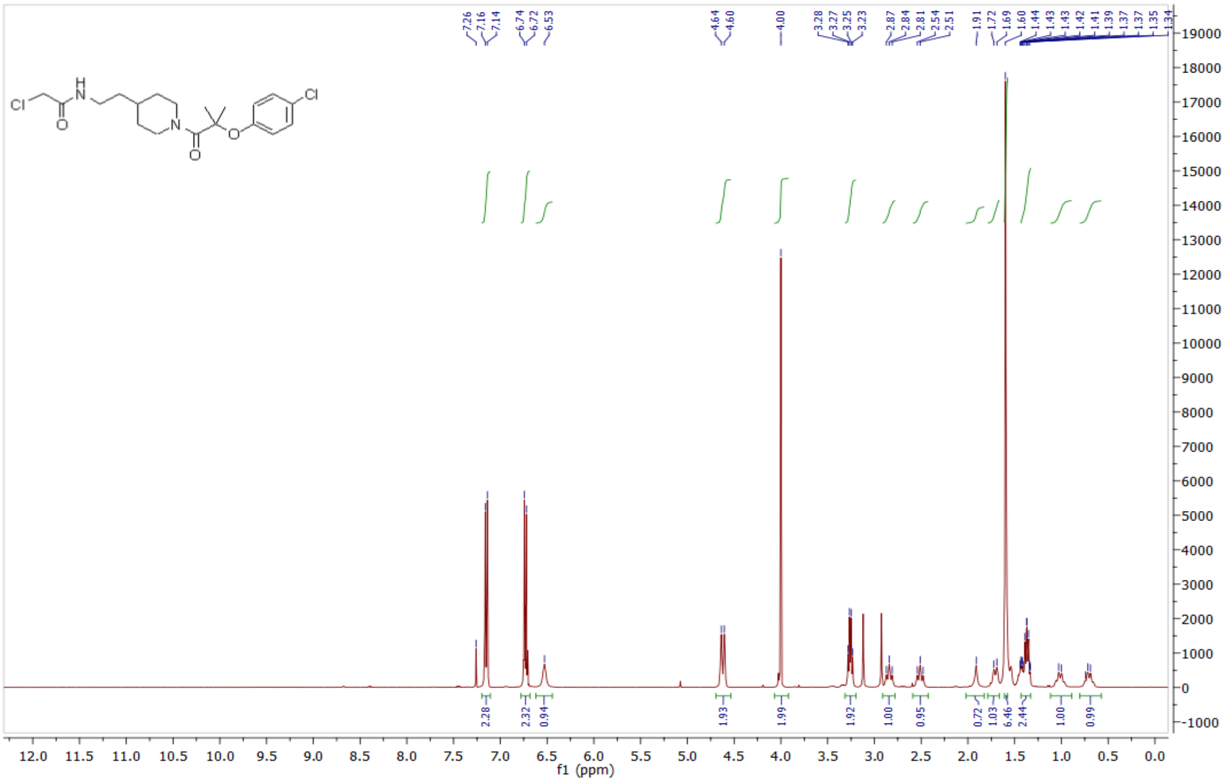

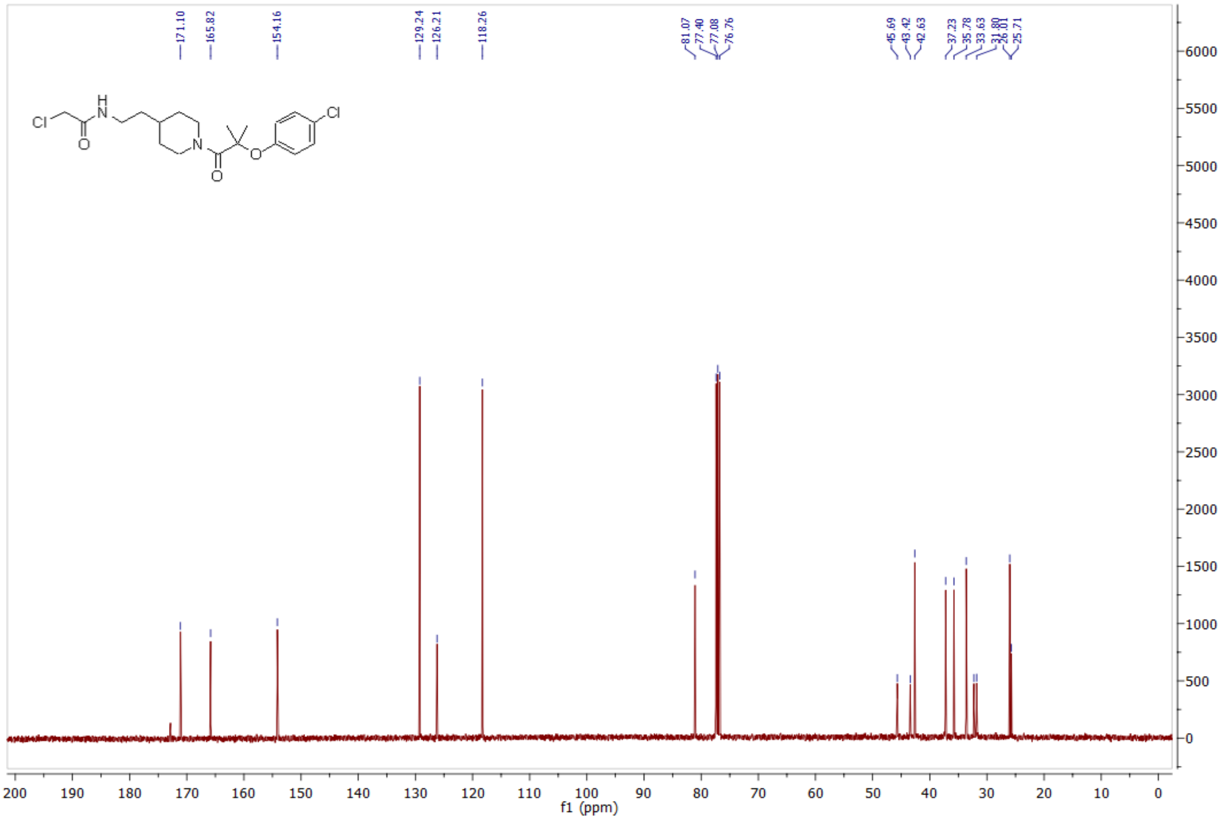

2-chloro-N-(3-(1-(2-(4-chlorophenoxy)-2-methylpropanoyl)piperidin-4-yl)propyl)acetamide **(29) 1074359**

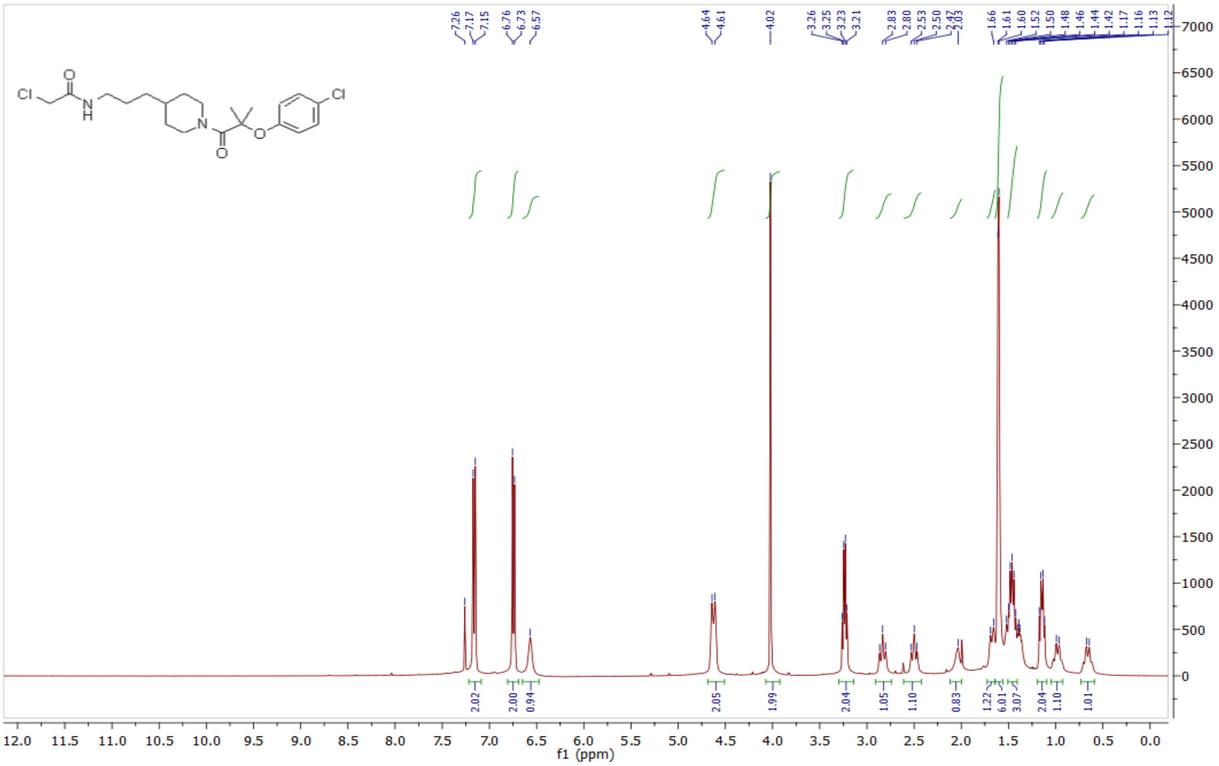

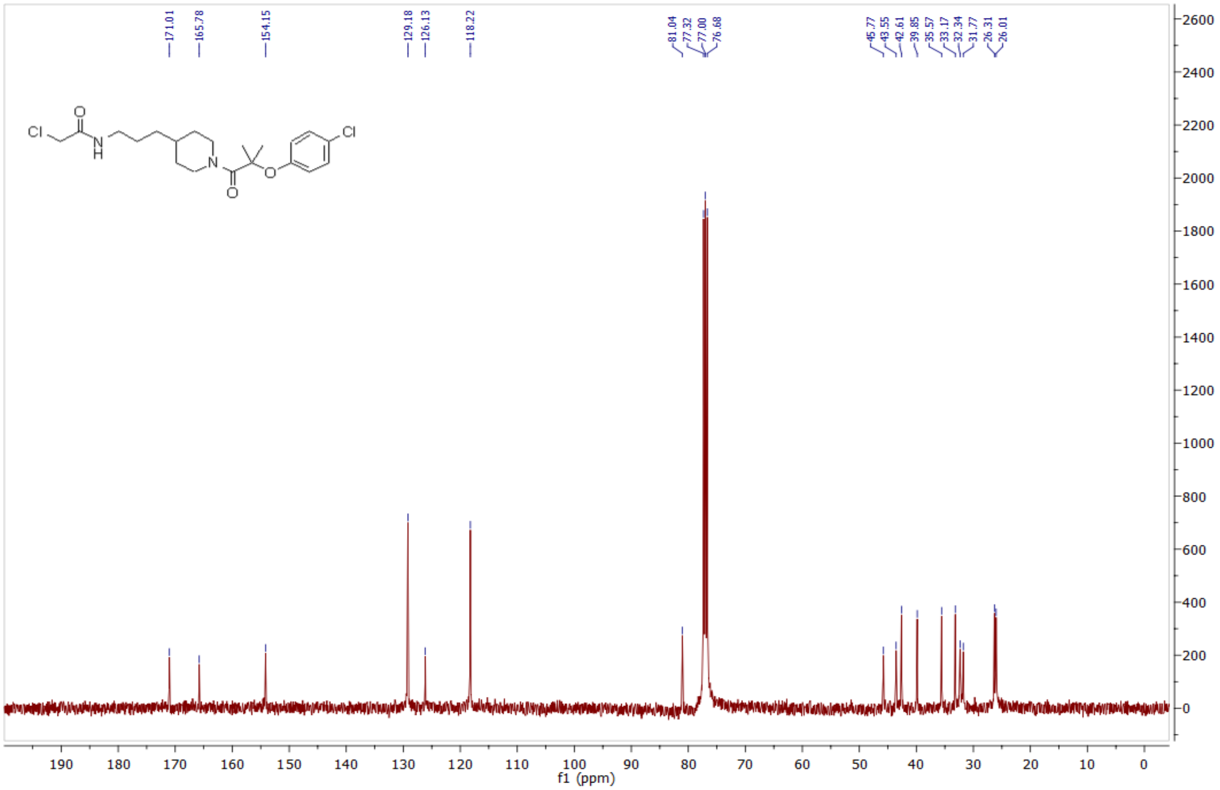

1-chloro-4-(1-(2-(4-chlorophenoxy)-2-methylpropanoyl)piperidin-4-yl)butan-2-one **(37) 1074203**

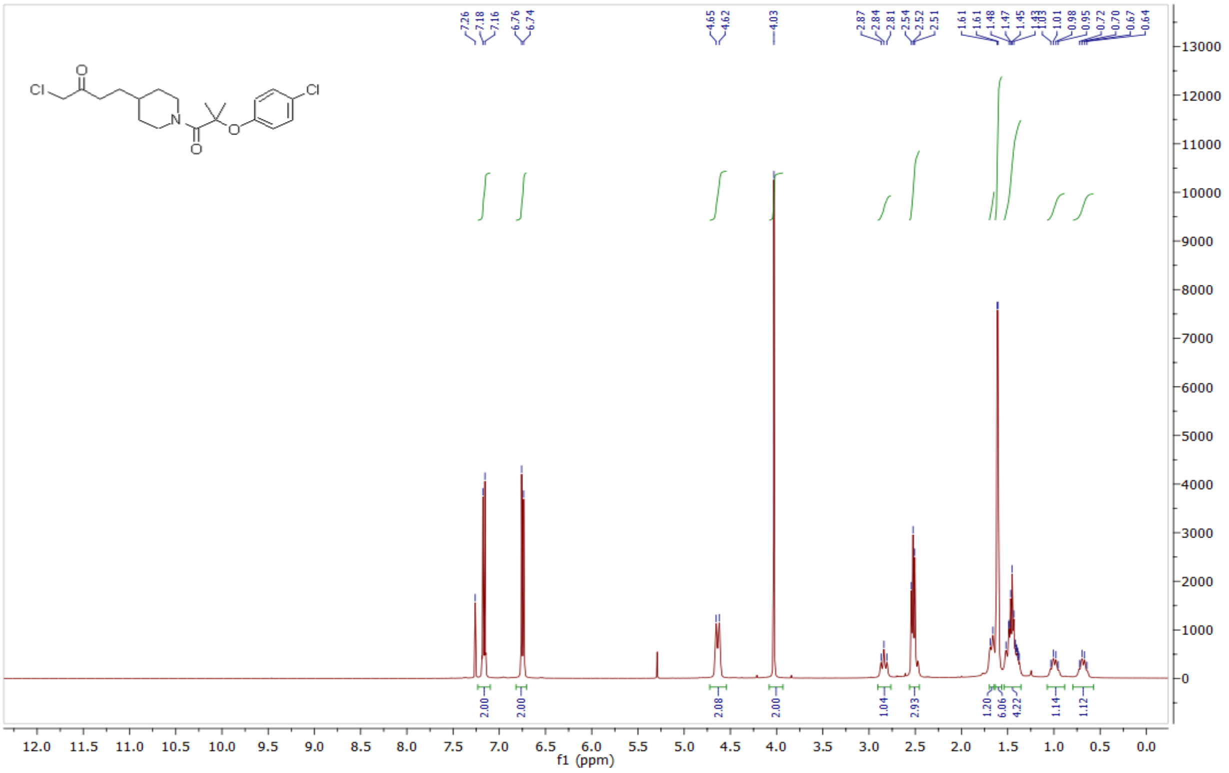

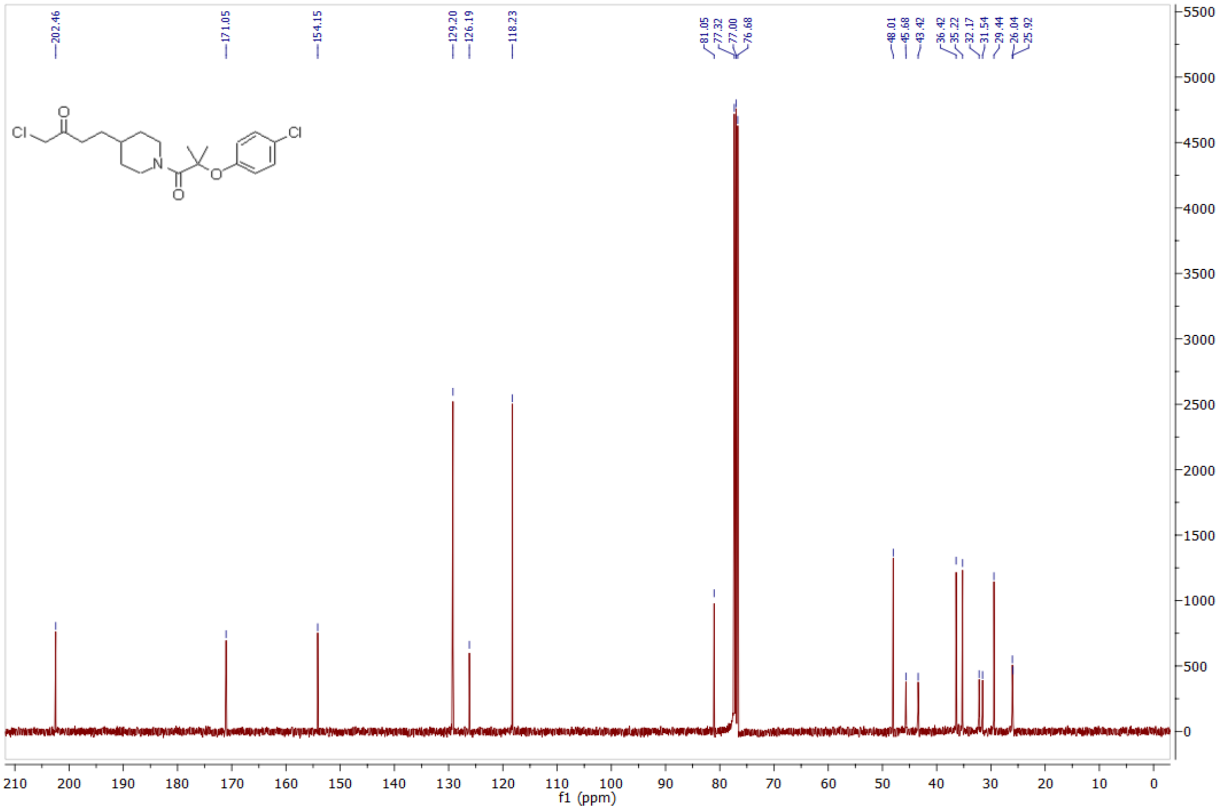

tert-butyl 4-((2-chloroacetamido)methyl)piperidine-1-carboxylate **(74)**

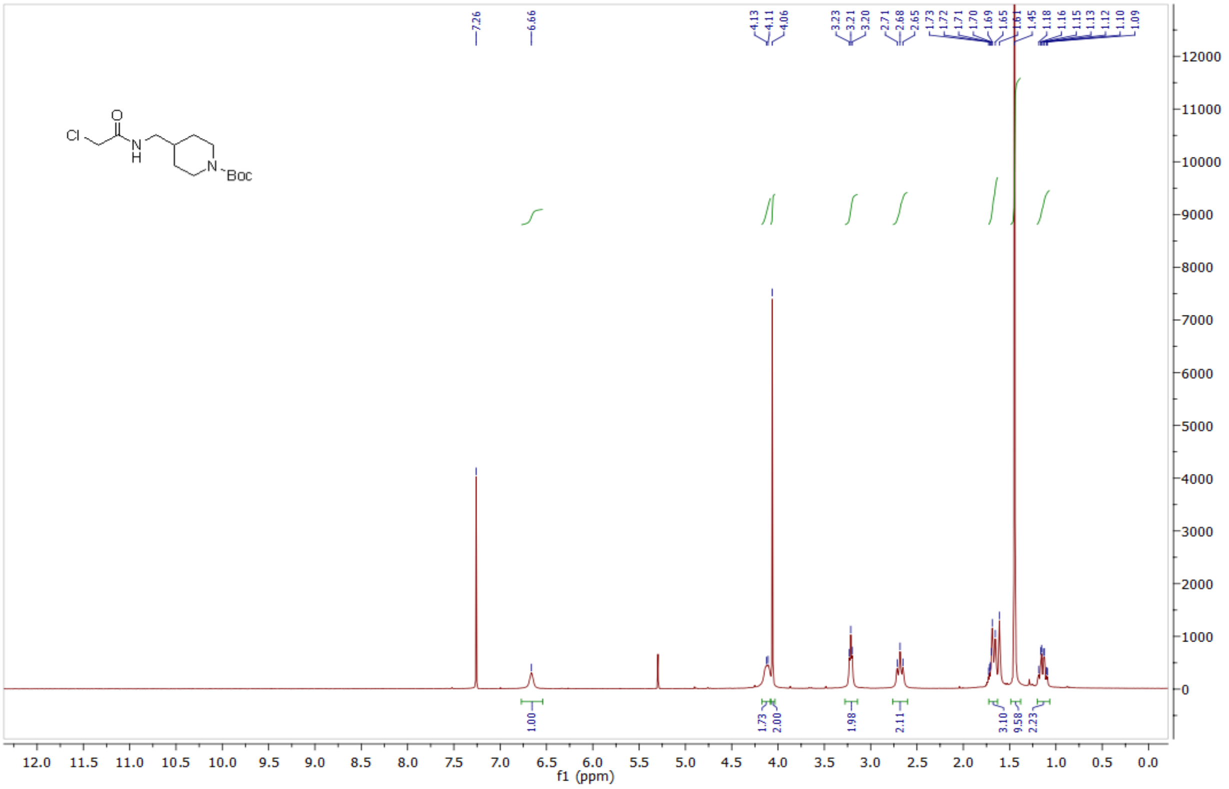

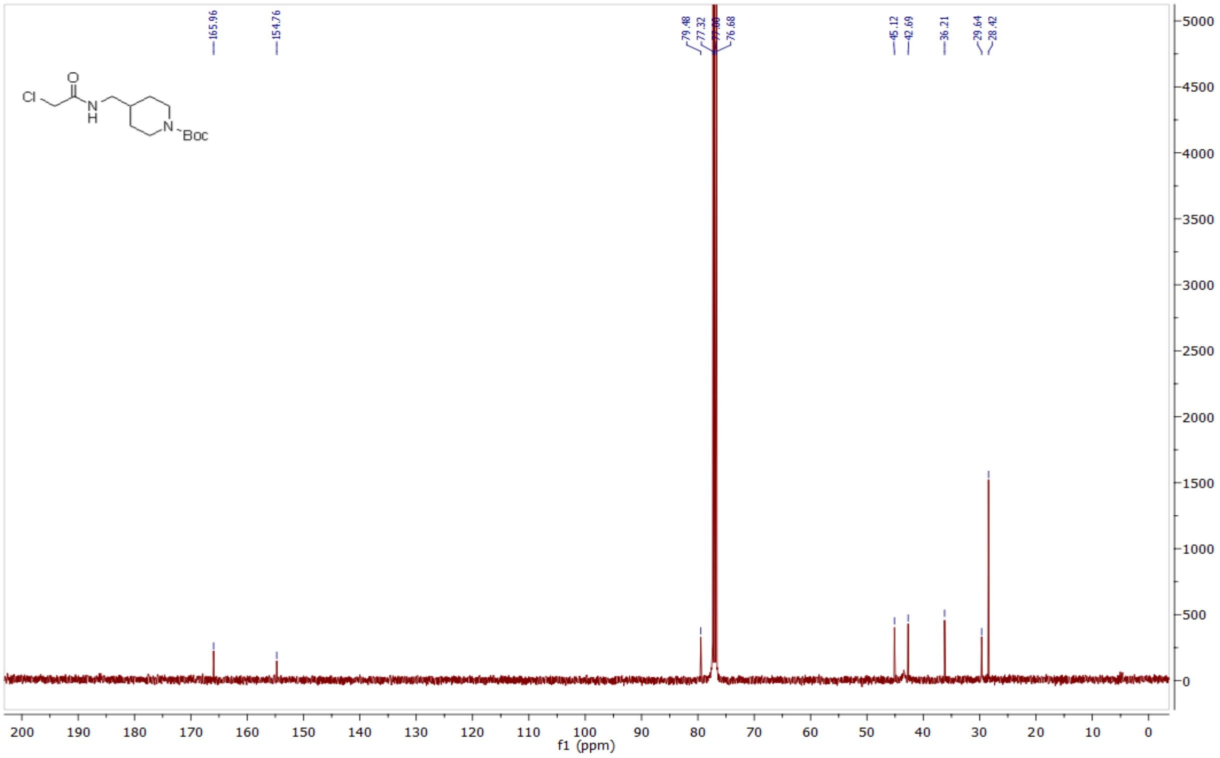

2-chloro-N-((1-(2-((4-chlorophenyl)amino)-2-methylpropanoyl)piperidin-4-yl)methyl)acetamide **(84) 1080291**

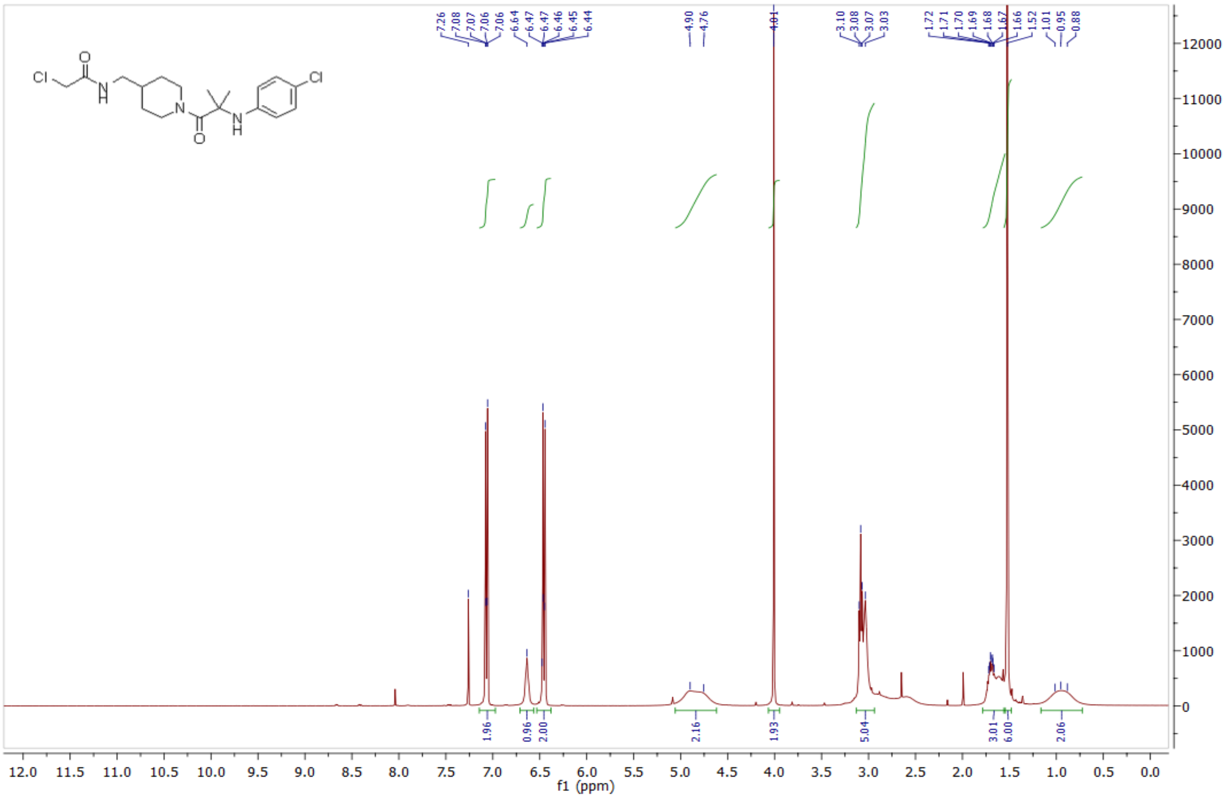

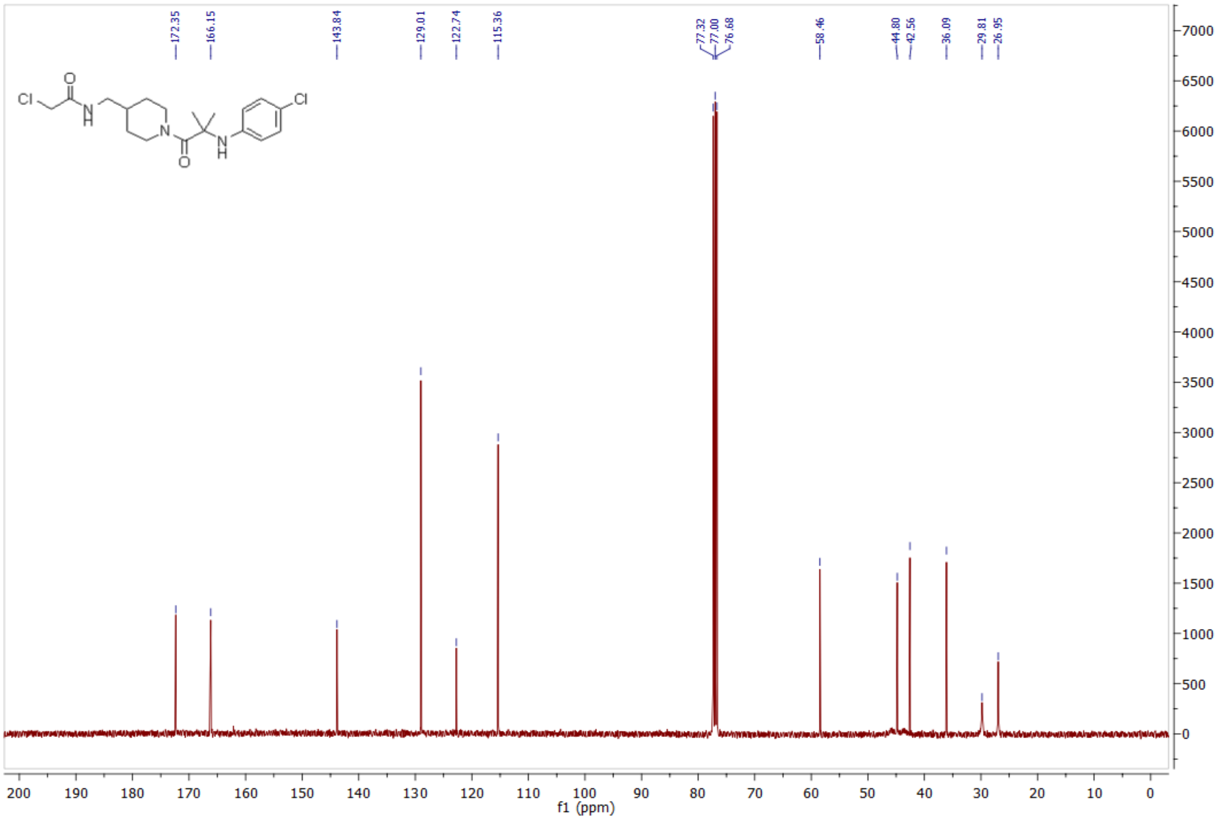

2-chloro-N-((1-(1-((4-chlorophenyl)amino)cyclopropane-1-carbonyl)piperidin-4-yl)methyl)acetamide **(97) 1076406**

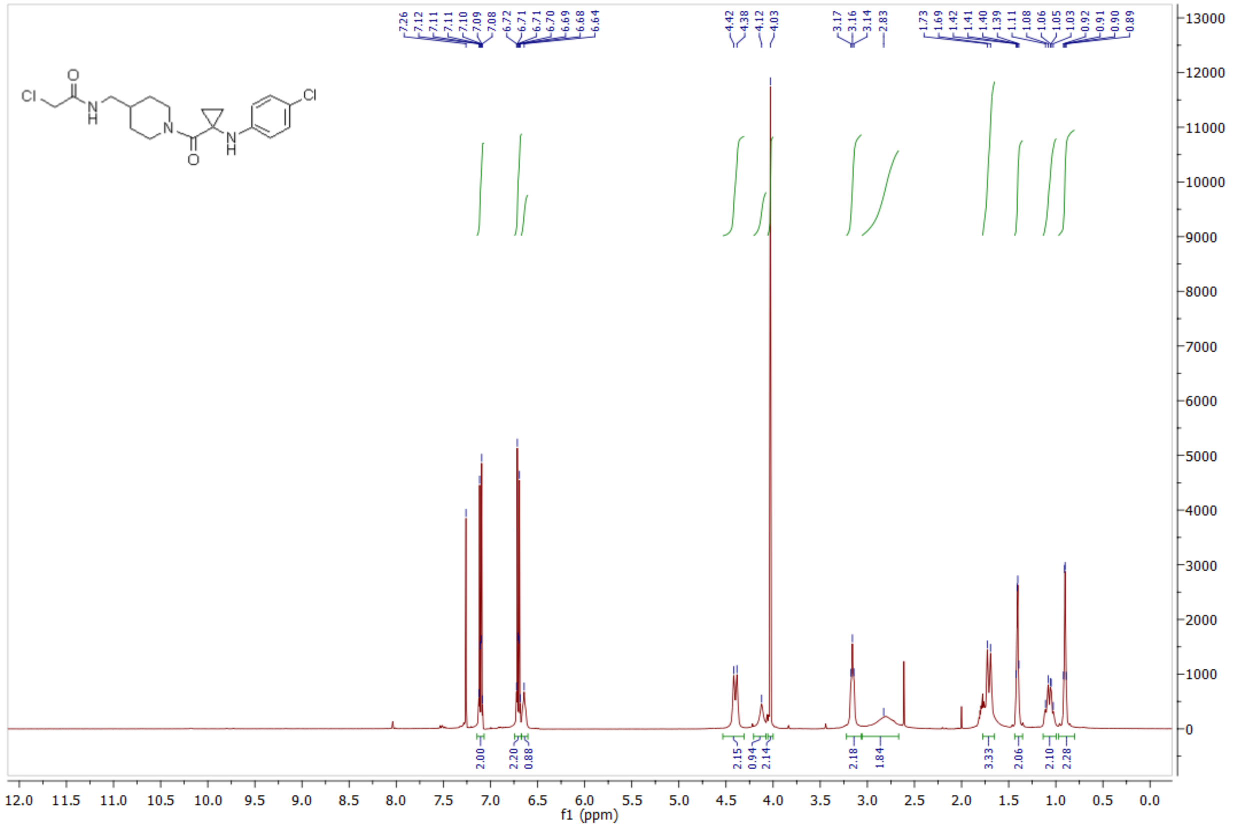

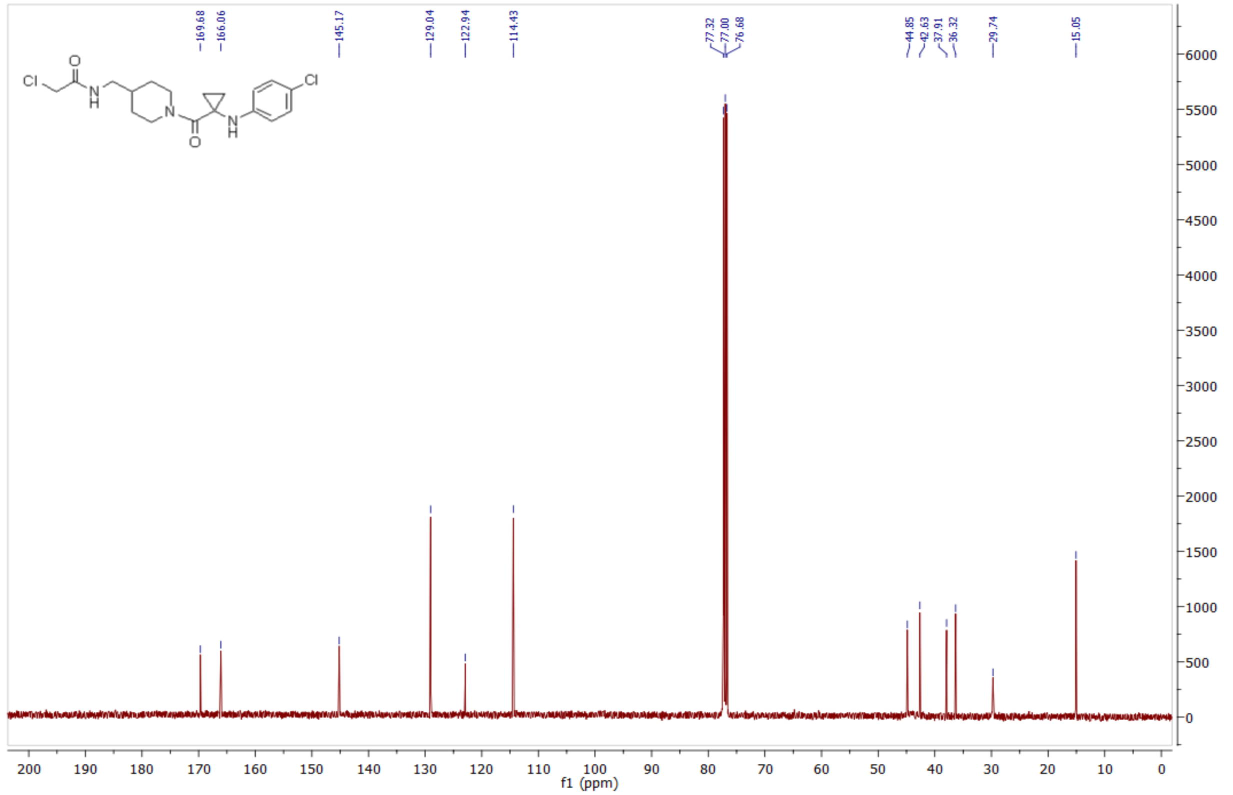

2-chloro-N-((1-(1-(phenylamino)cyclobutane-1-carbonyl)piperidin-4-yl)methyl)acetamide **(98) 1080269**

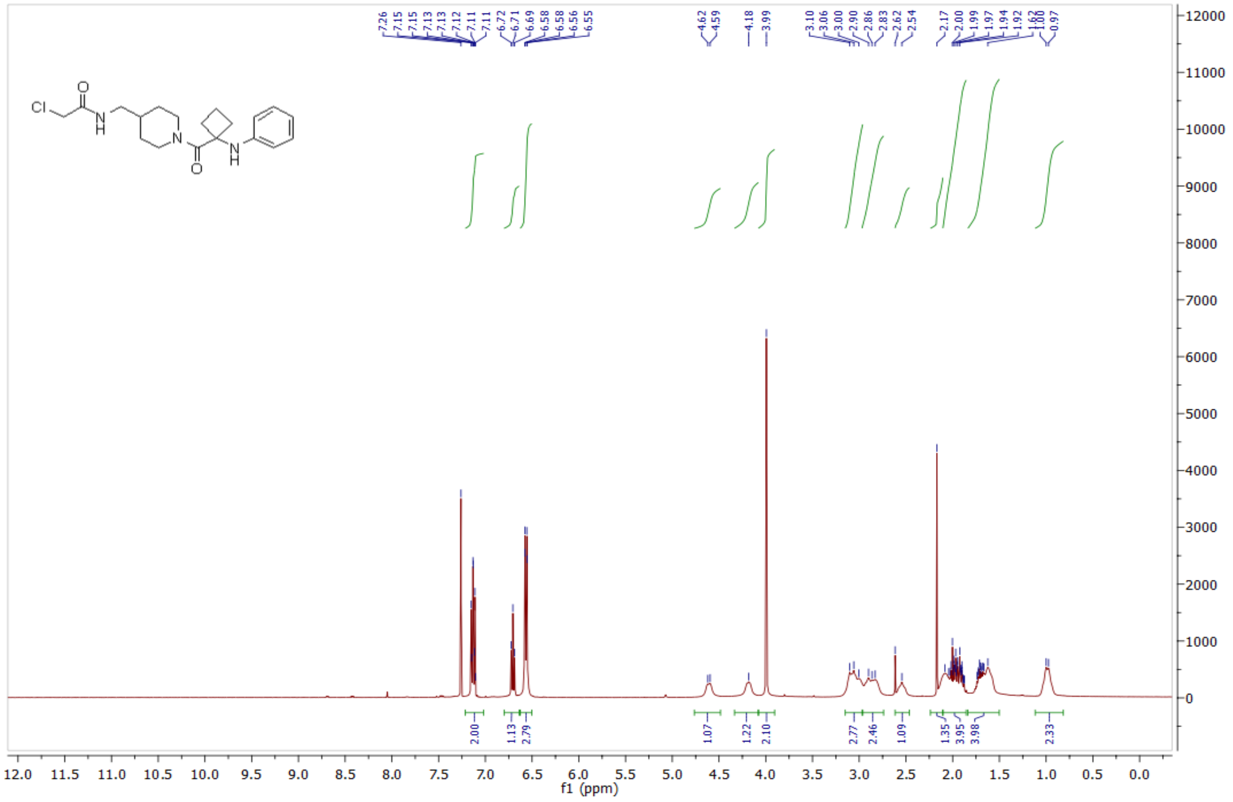

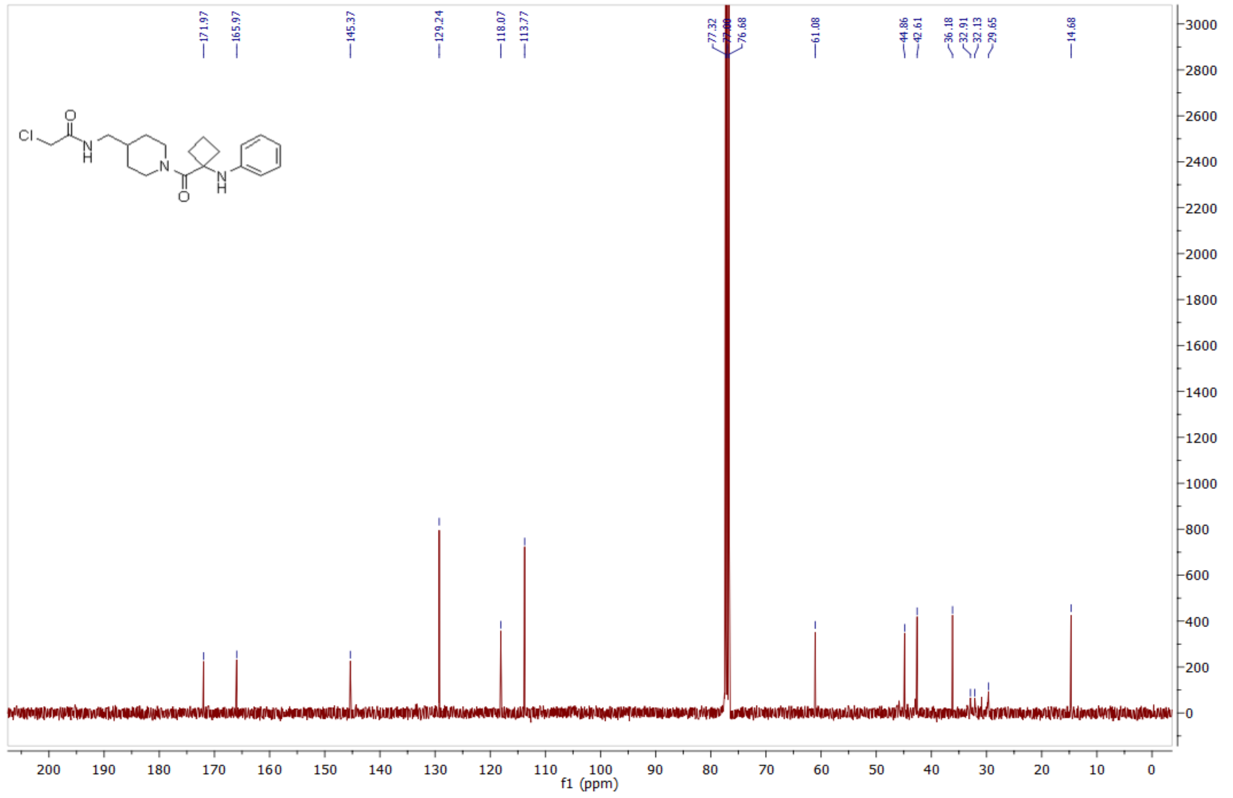

2-Chloro-N-((1-(1-(4-chlorophenoxy)cyclopentane-1-carbonyl)piperidin-4-yl)methyl)acetamide **(117) 1075297**

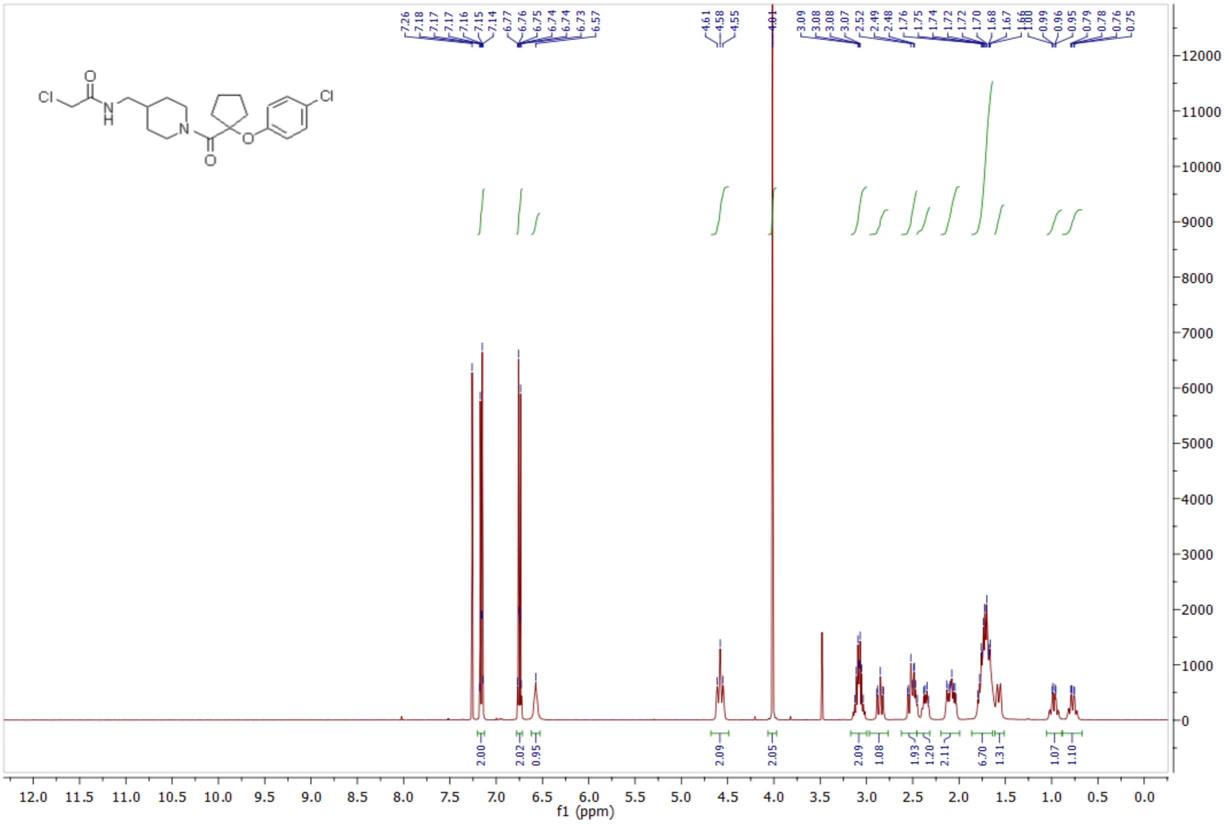

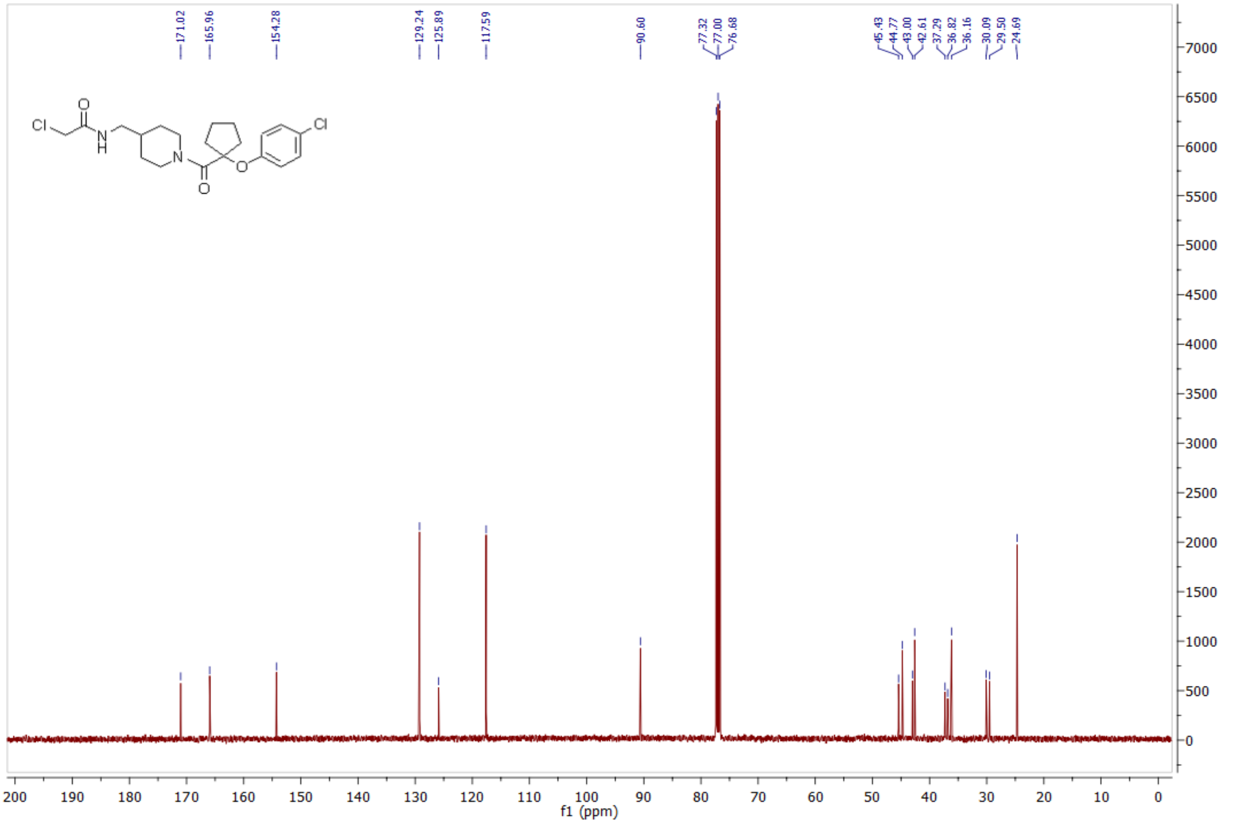

2-Chloro-N-((1-(1-((4-chlorophenyl)amino)cyclopentane-1-carbonyl)piperidin-4-yl)methyl)acetamide **(118) 1075306**

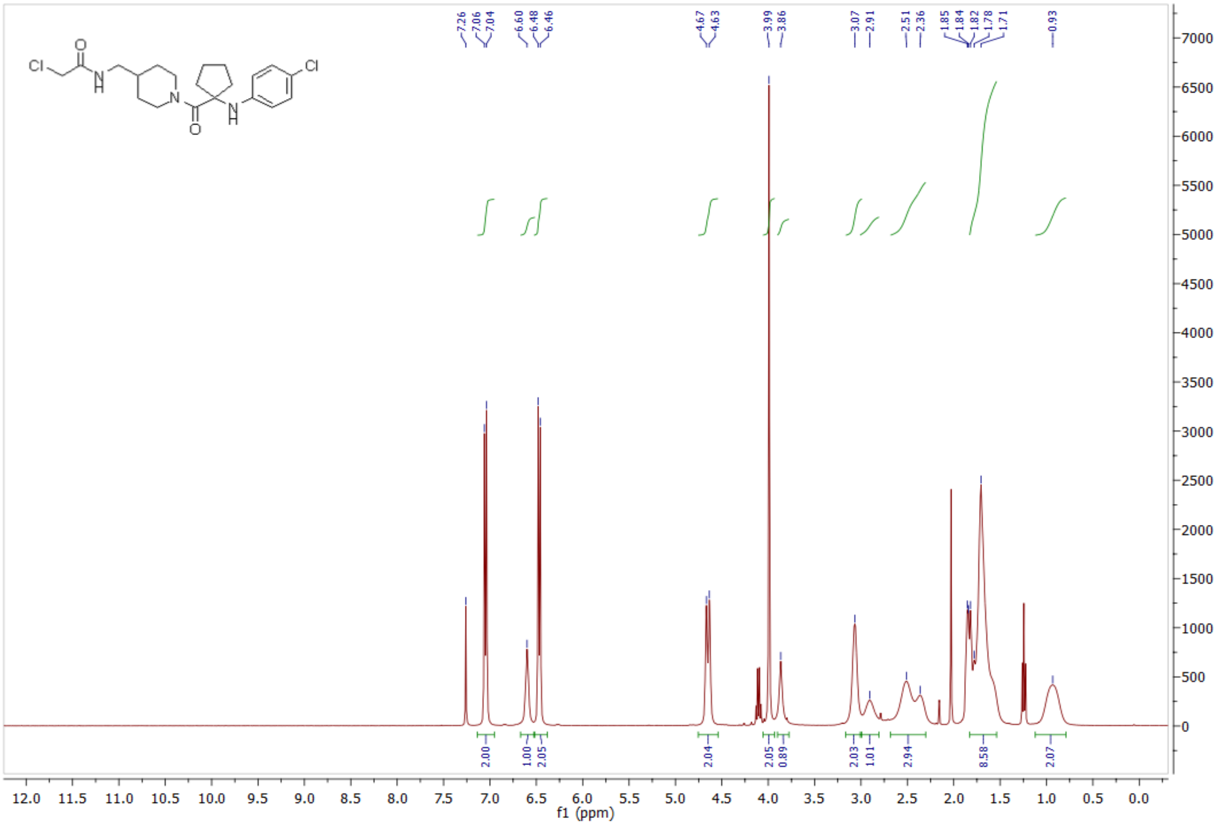

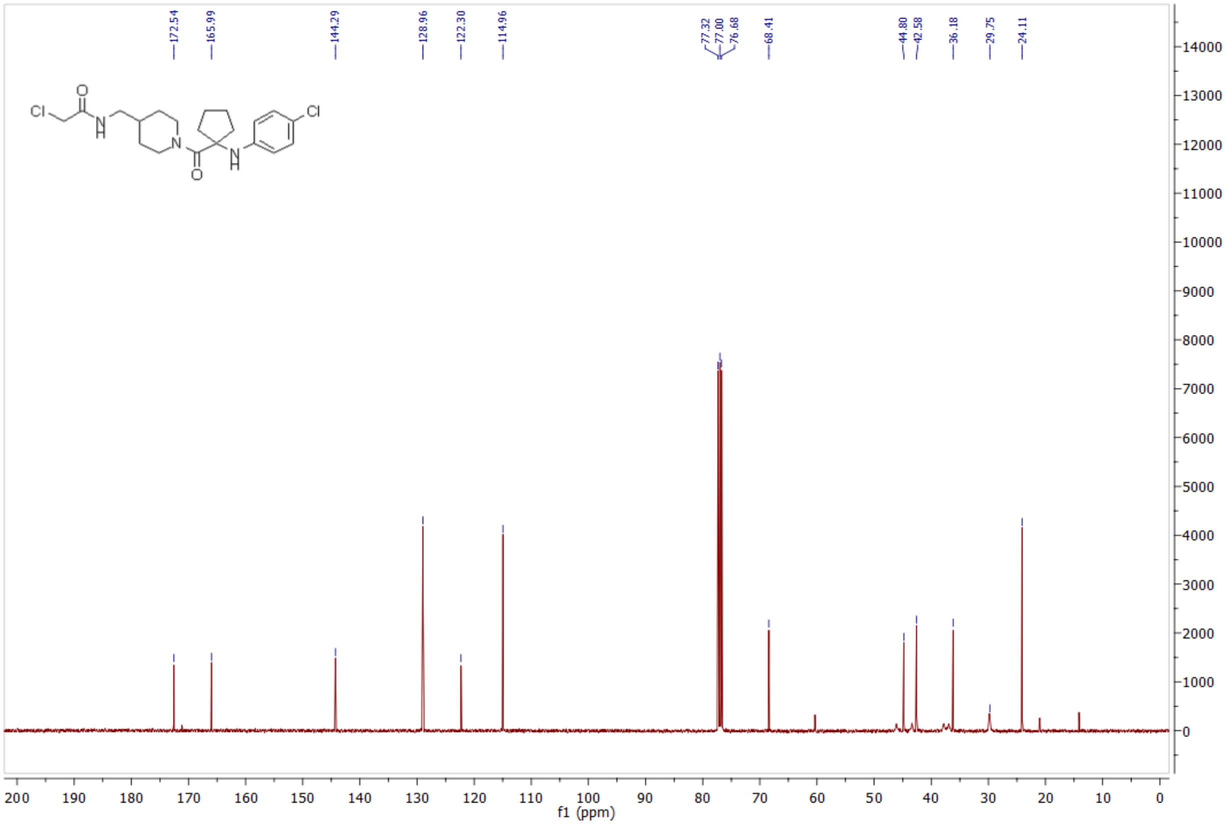

2-Chloro-N-((1-(1-phenoxycyclopentane-1-carbonyl)piperidin-4-yl)methyl)acetamide **(119) 1075300**

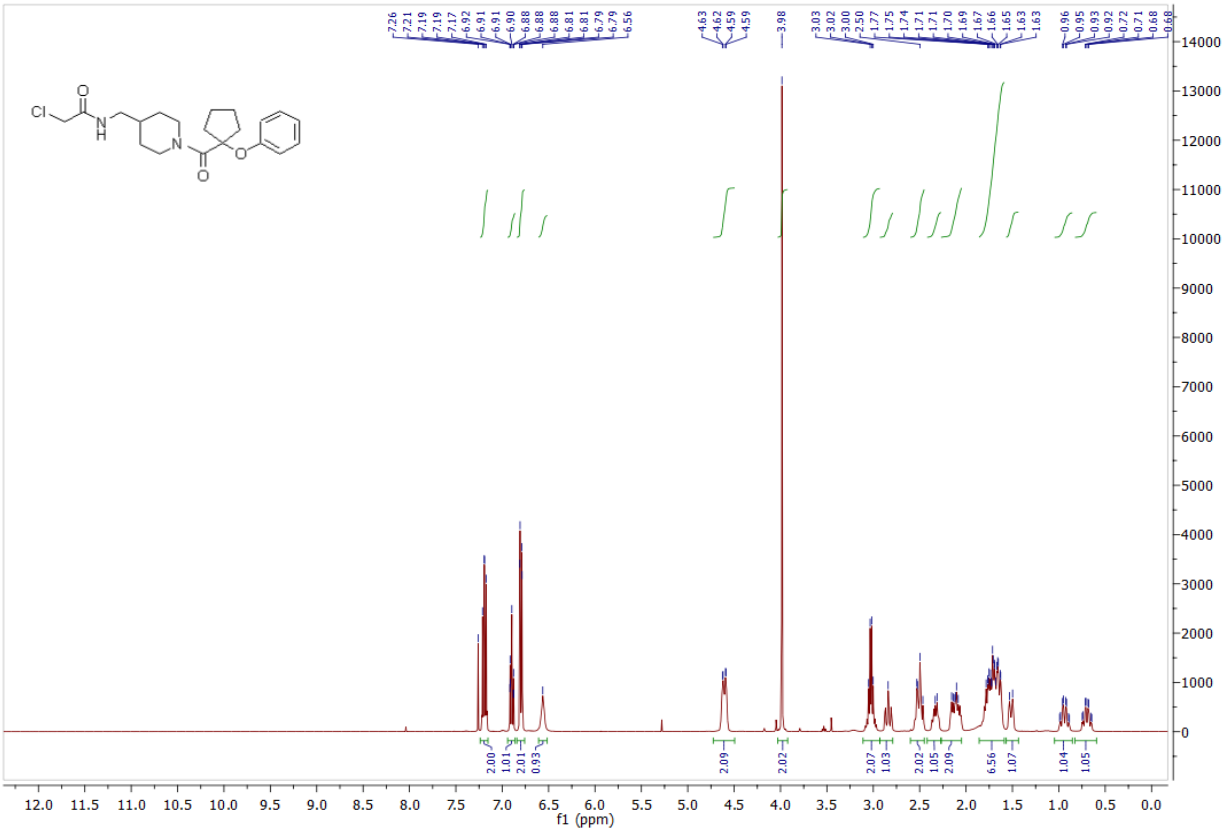

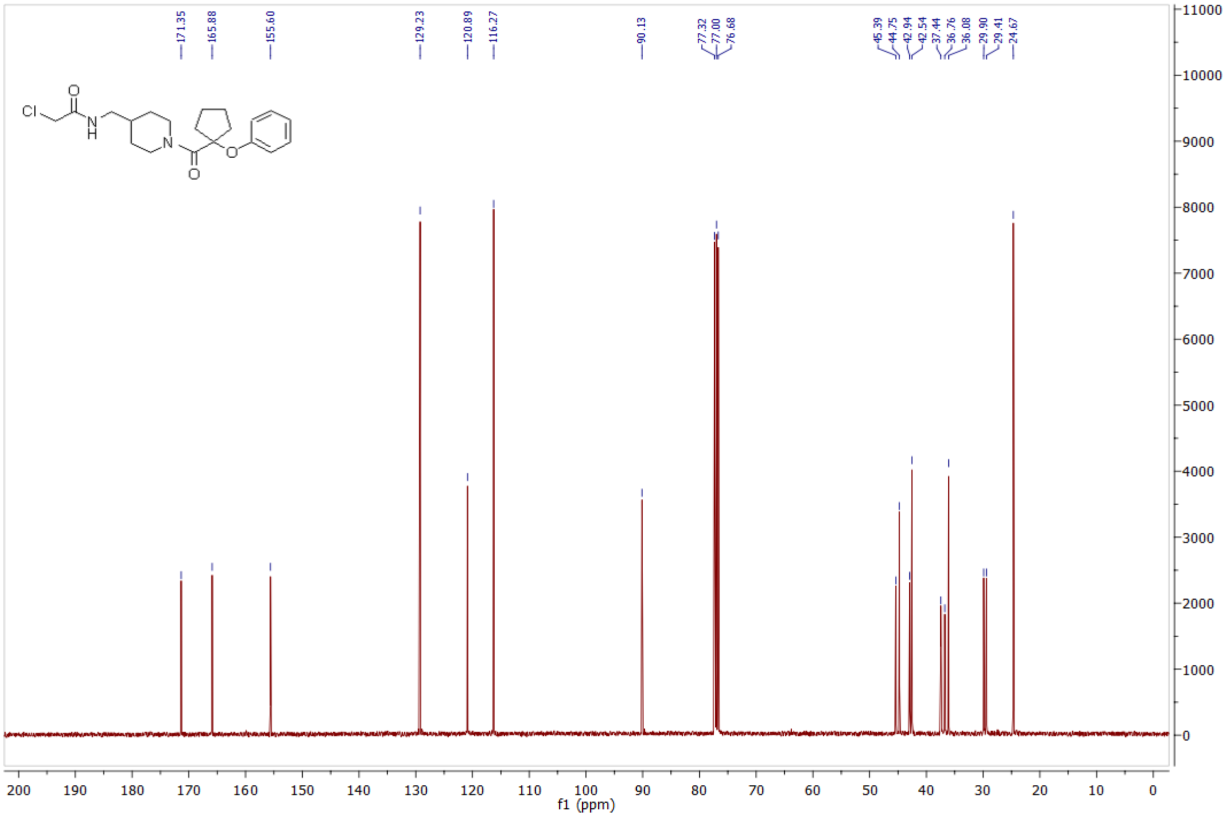

2-chloro-N-((1-(1-(4-chlorophenoxy)cyclohexane-1-carbonyl)piperidin-4-yl)methyl)acetamide **(122) 1080273**

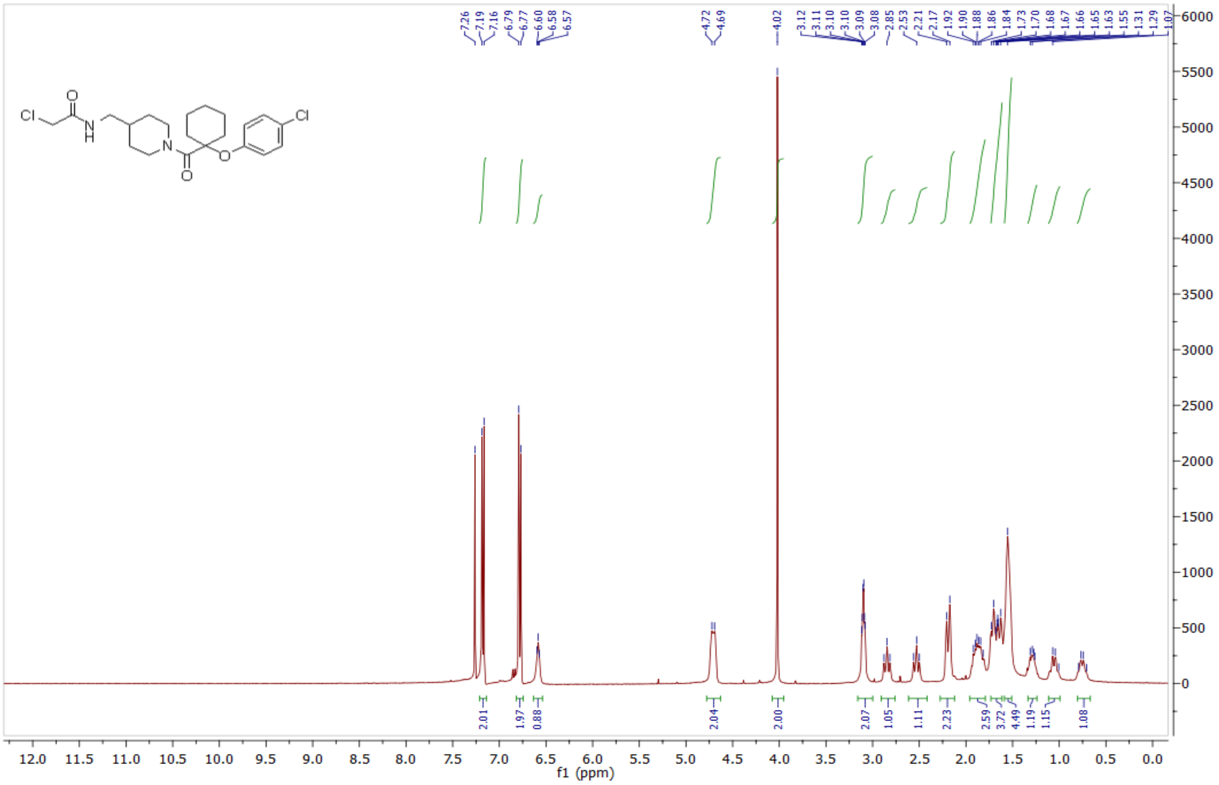

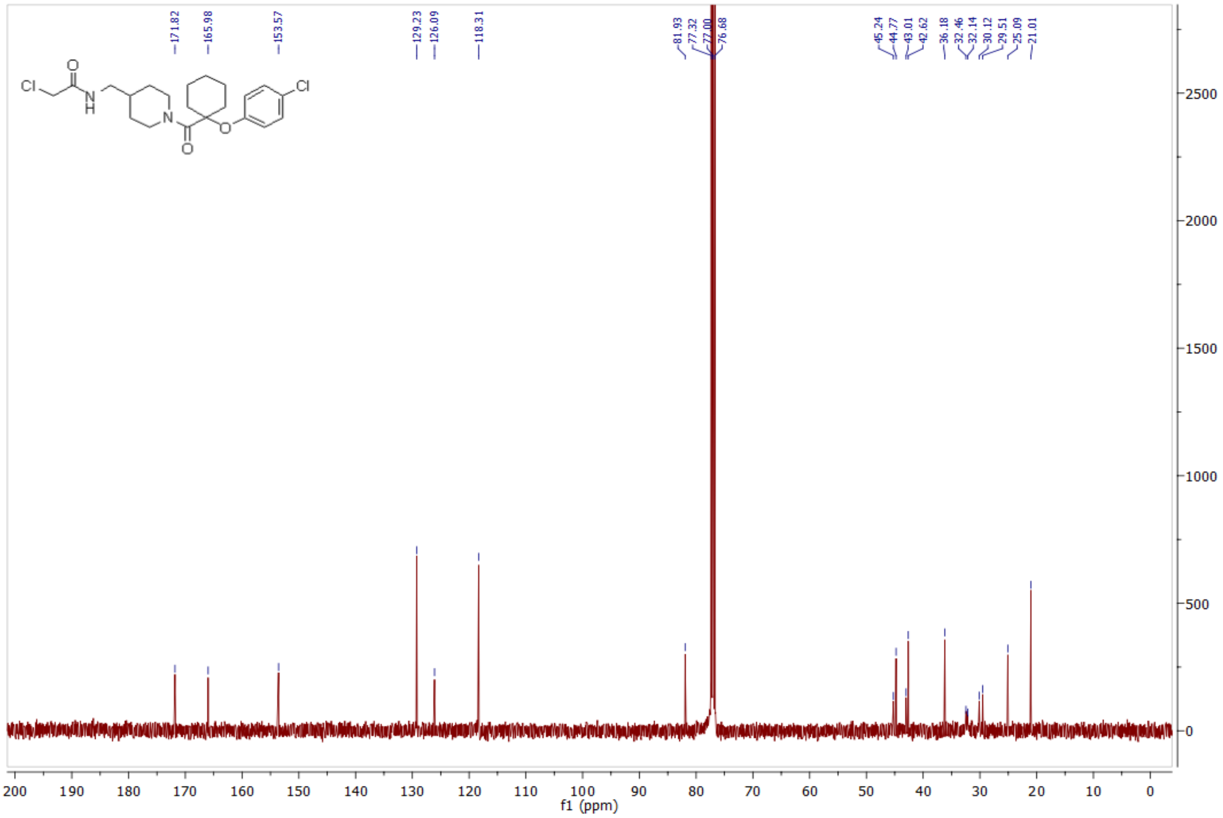

2-chloro-N-((1-(1-((4-chlorophenyl)amino)cyclohexane-1-carbonyl)piperidin-4-yl)methyl)acetamide **(123) 1076403**

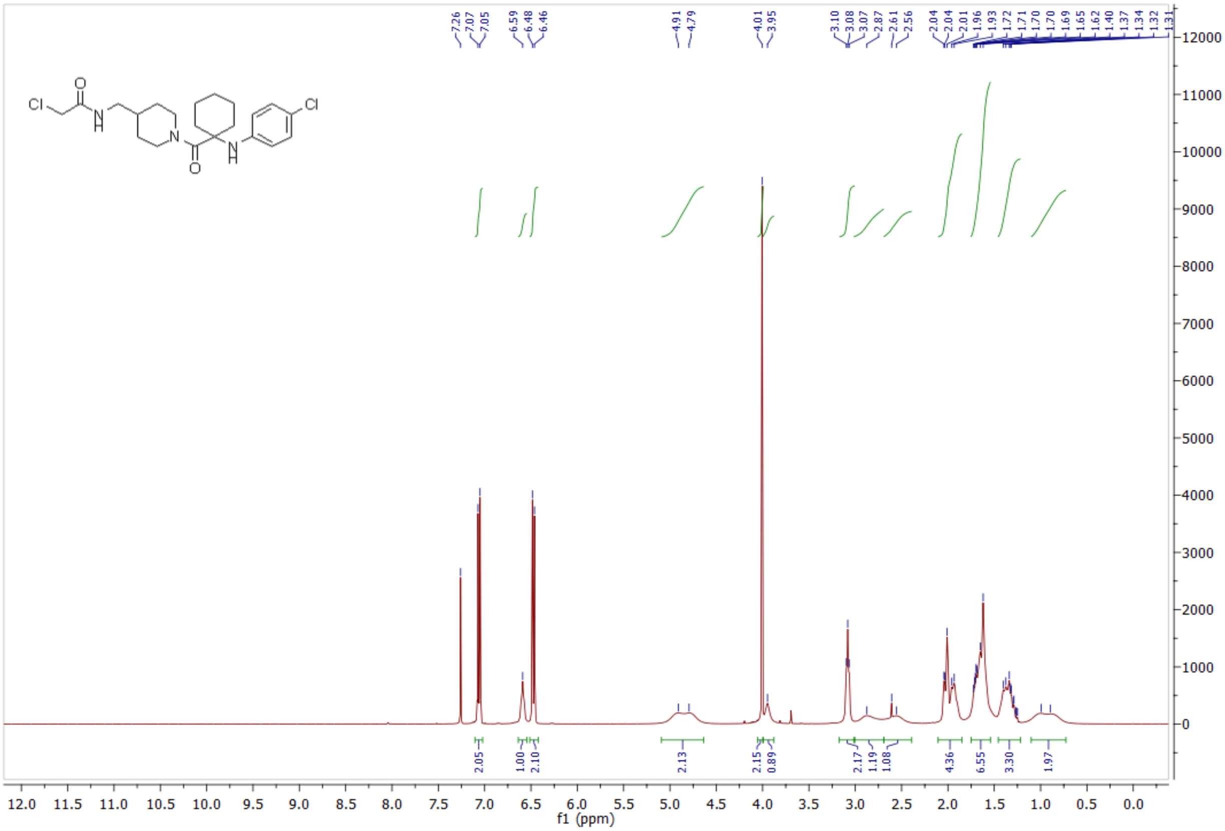

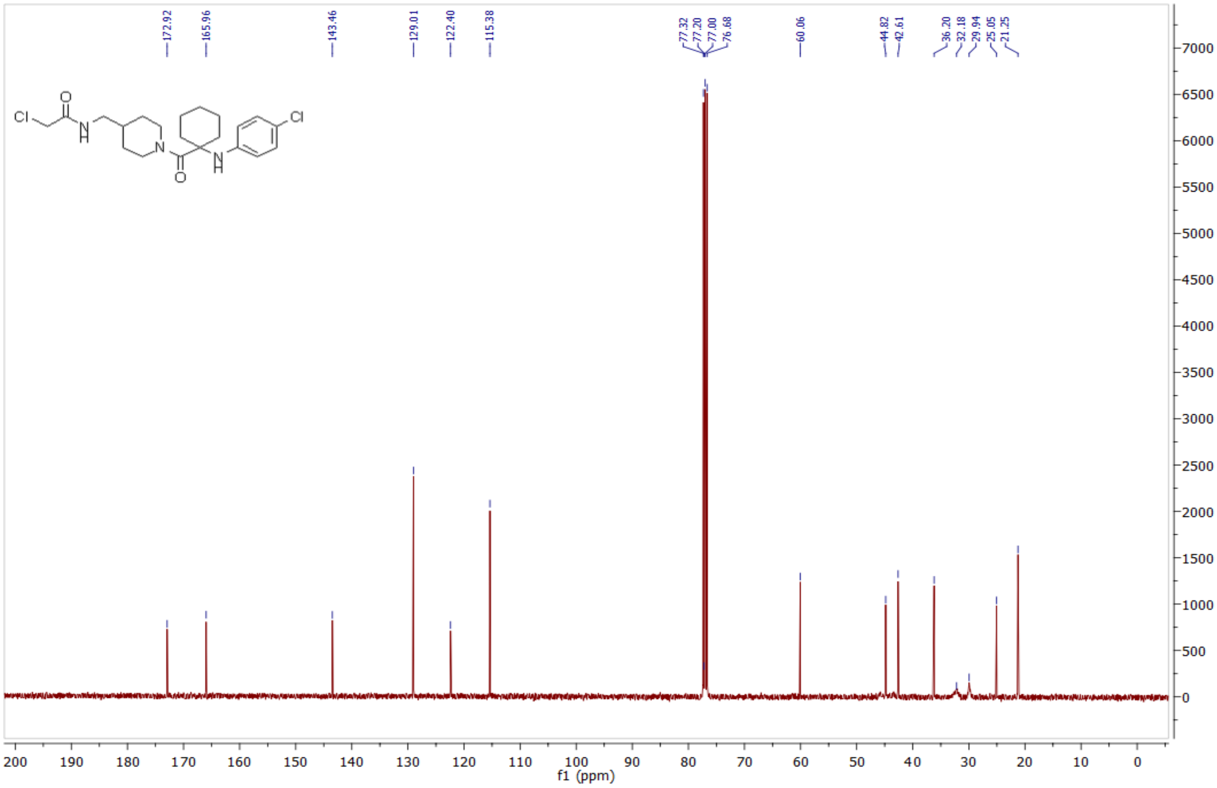

2-Chloro-N-((1-(1-(4-chlorophenoxy)-4,4-difluorocyclohexane-1-carbonyl)piperidin-4-yl)methyl)acetamide **(124)**
1075299

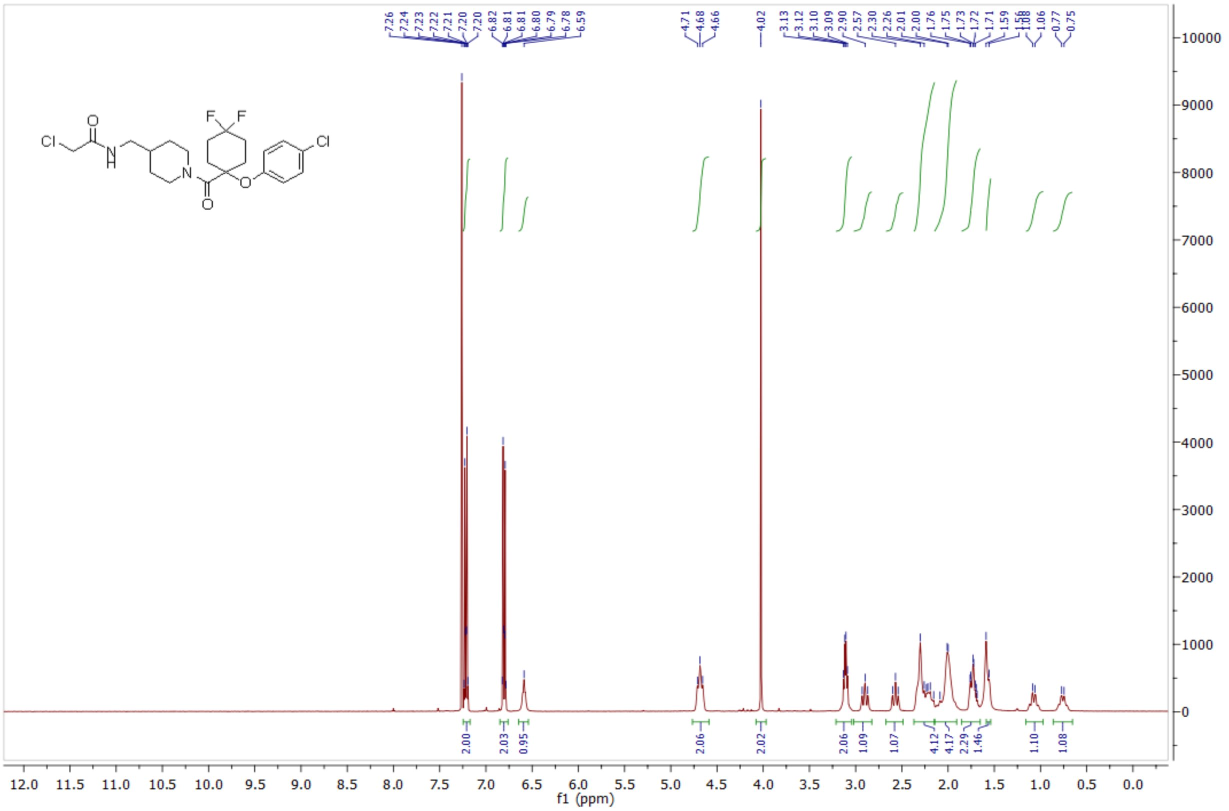

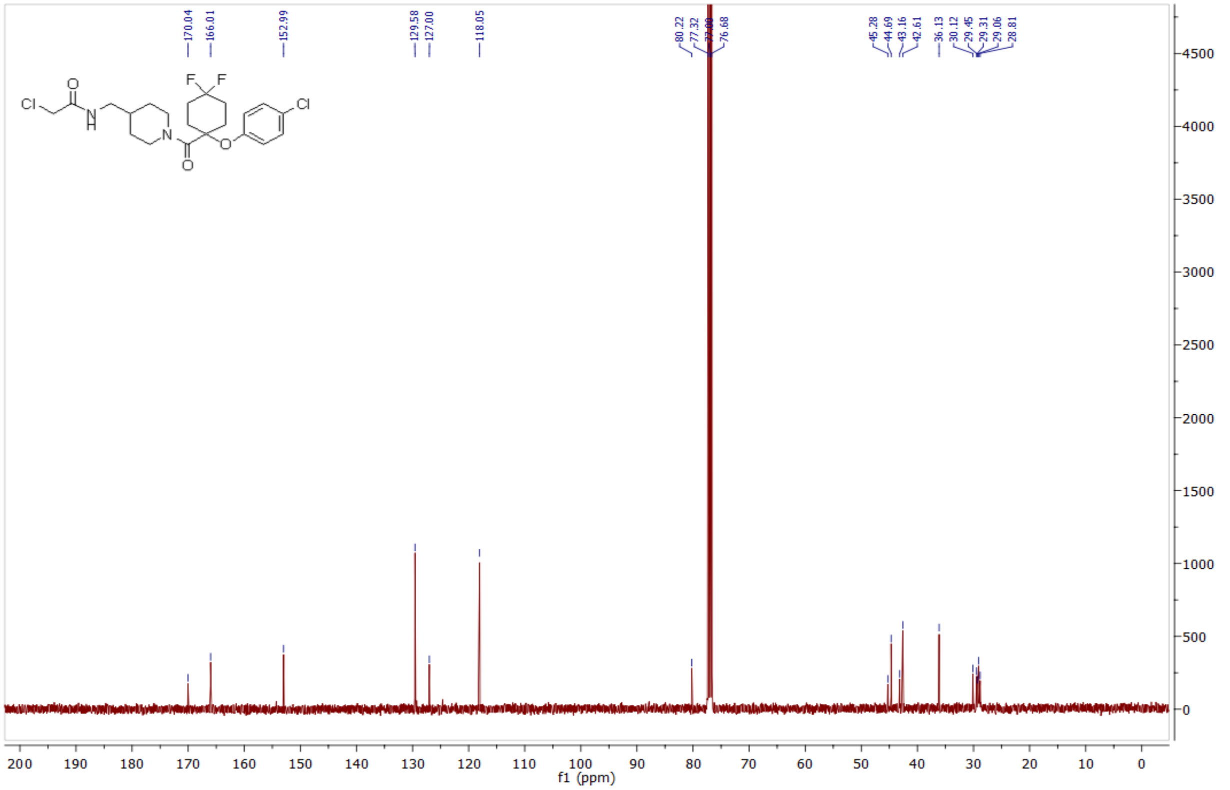

2-Chloro-N-((1-(1-((4-chlorophenyl)amino)-4,4-difluorocyclohexane-1-carbonyl)piperidin-4-yl)methyl)acetamide

(125) 1075311

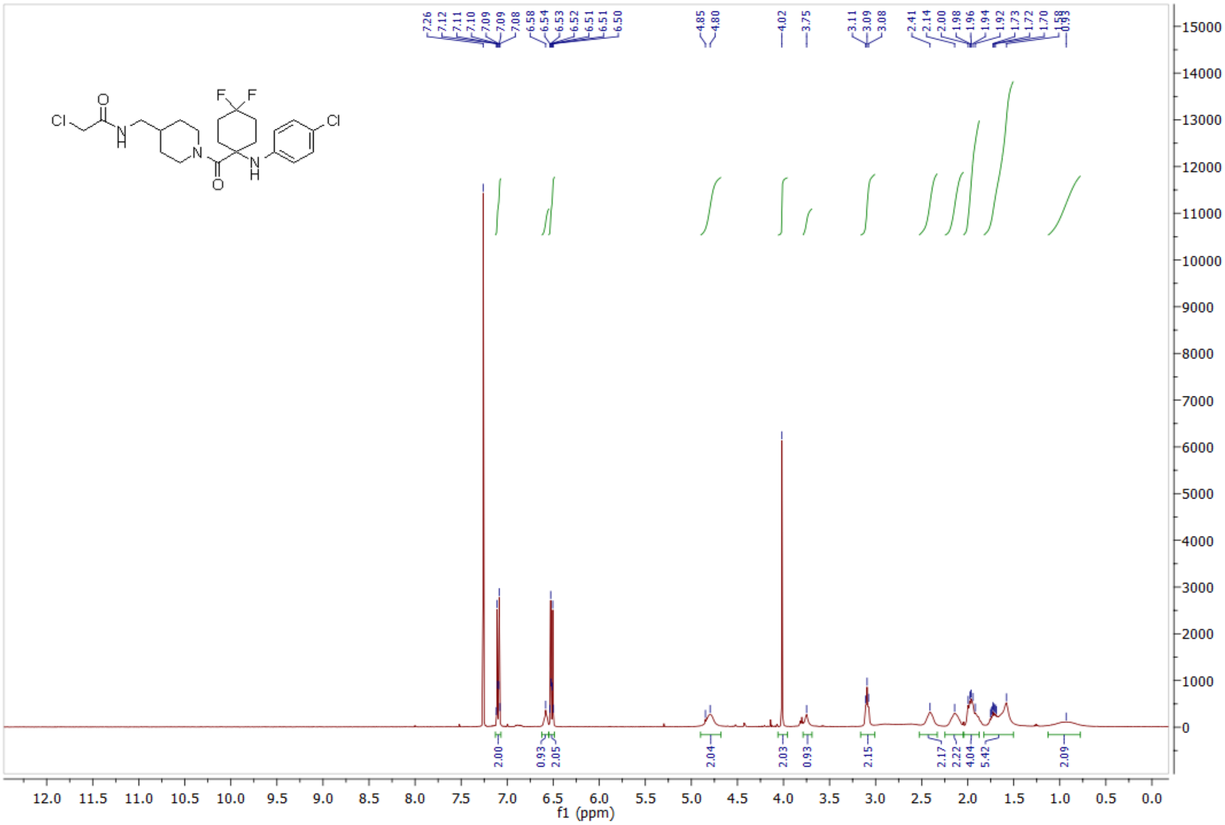

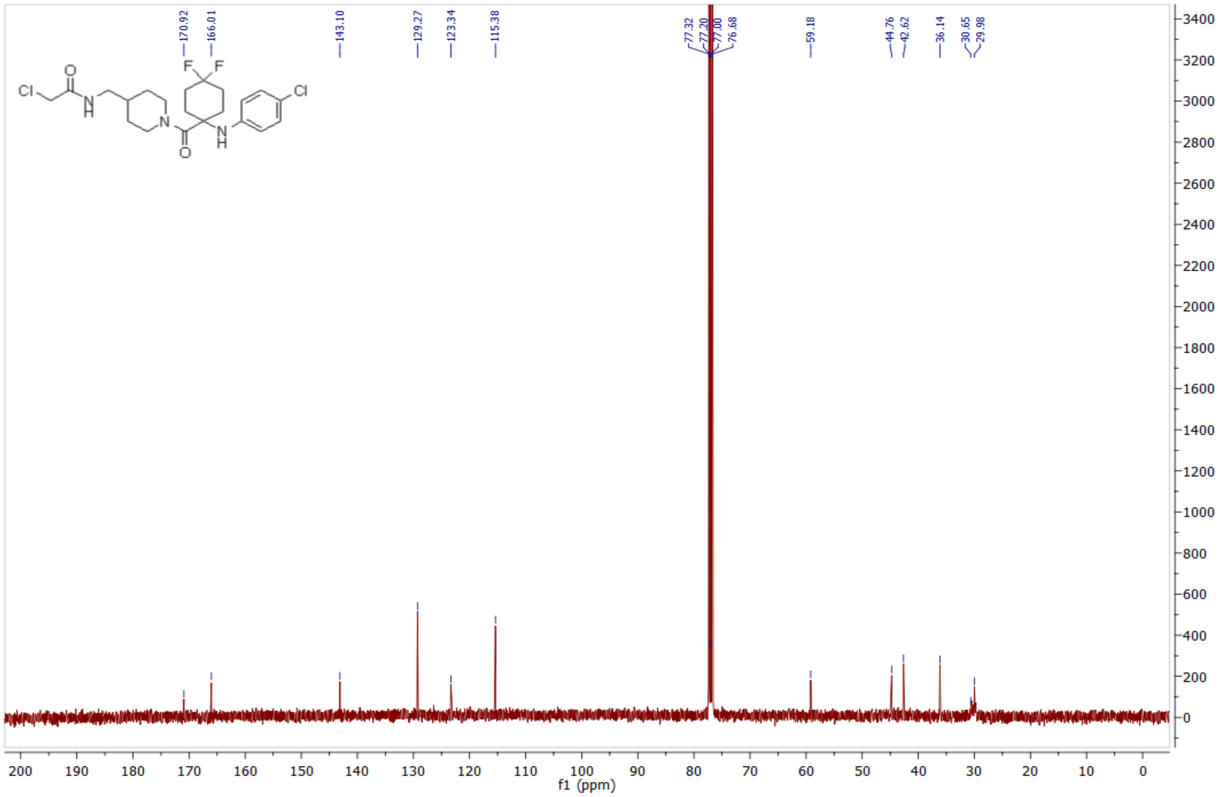

2-Chloro-N-((1-(4-(4-chlorophenoxy)tetrahydro-2H-pyran-4-carbonyl)piperidin-4-yl)methyl)acetamide
(126) 1075305

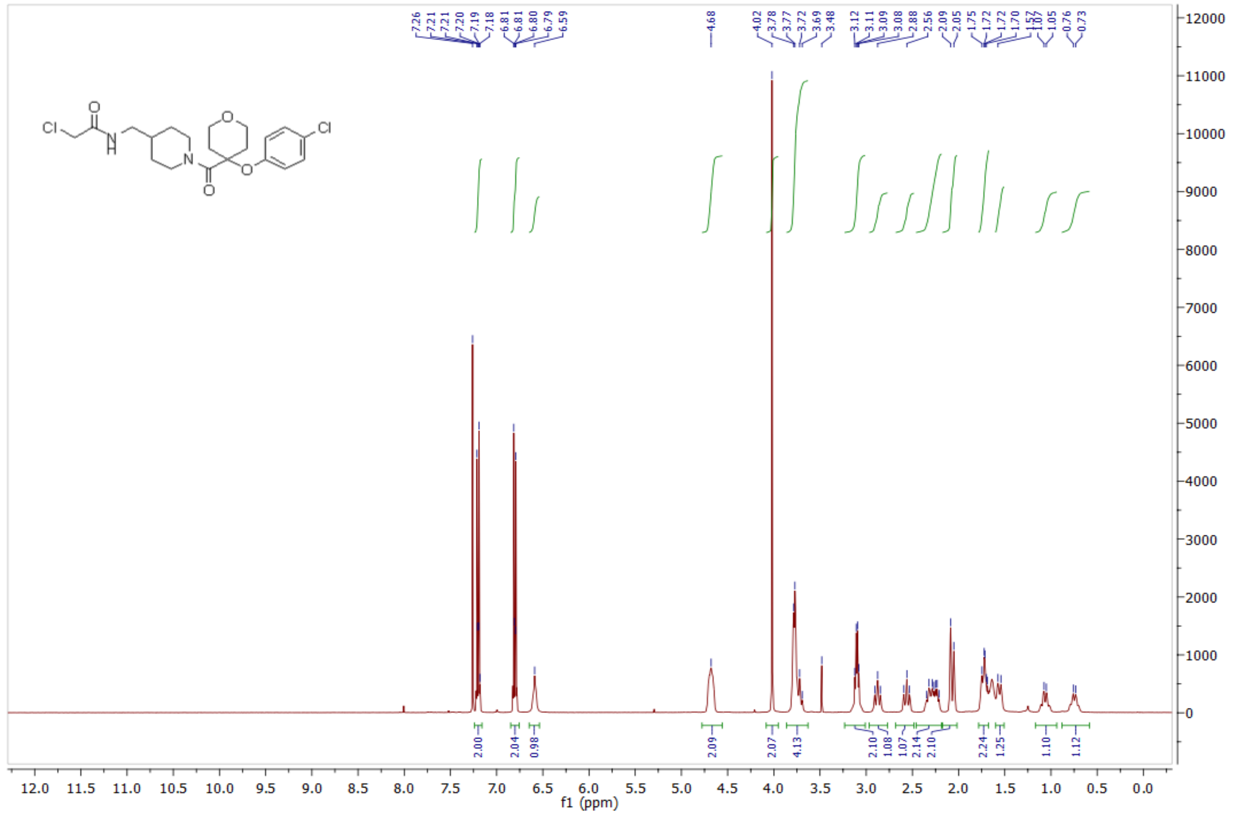

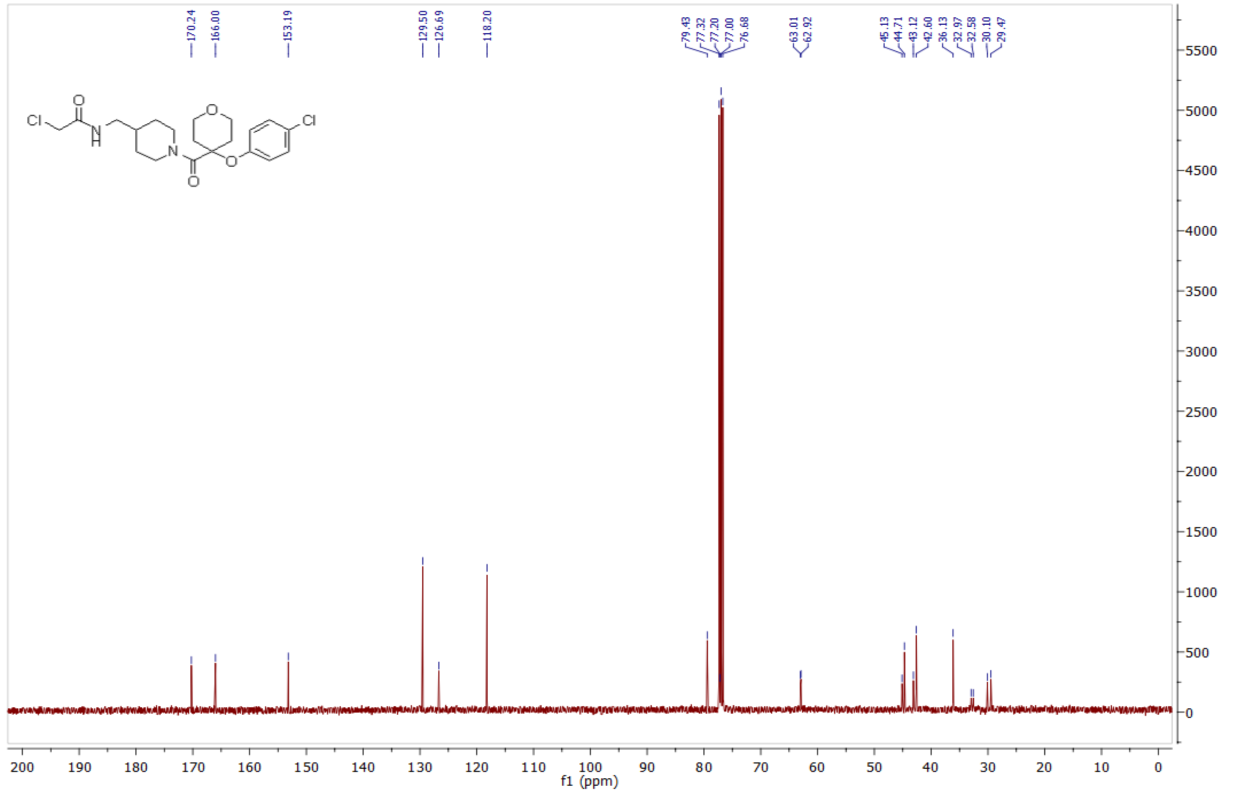

2-Chloro-N-((1-(4-((4-chlorophenyl)amino)tetrahydro-2H-pyran-4-carbonyl)piperidin-4-yl)methyl)acetamide **(127)**
1075310

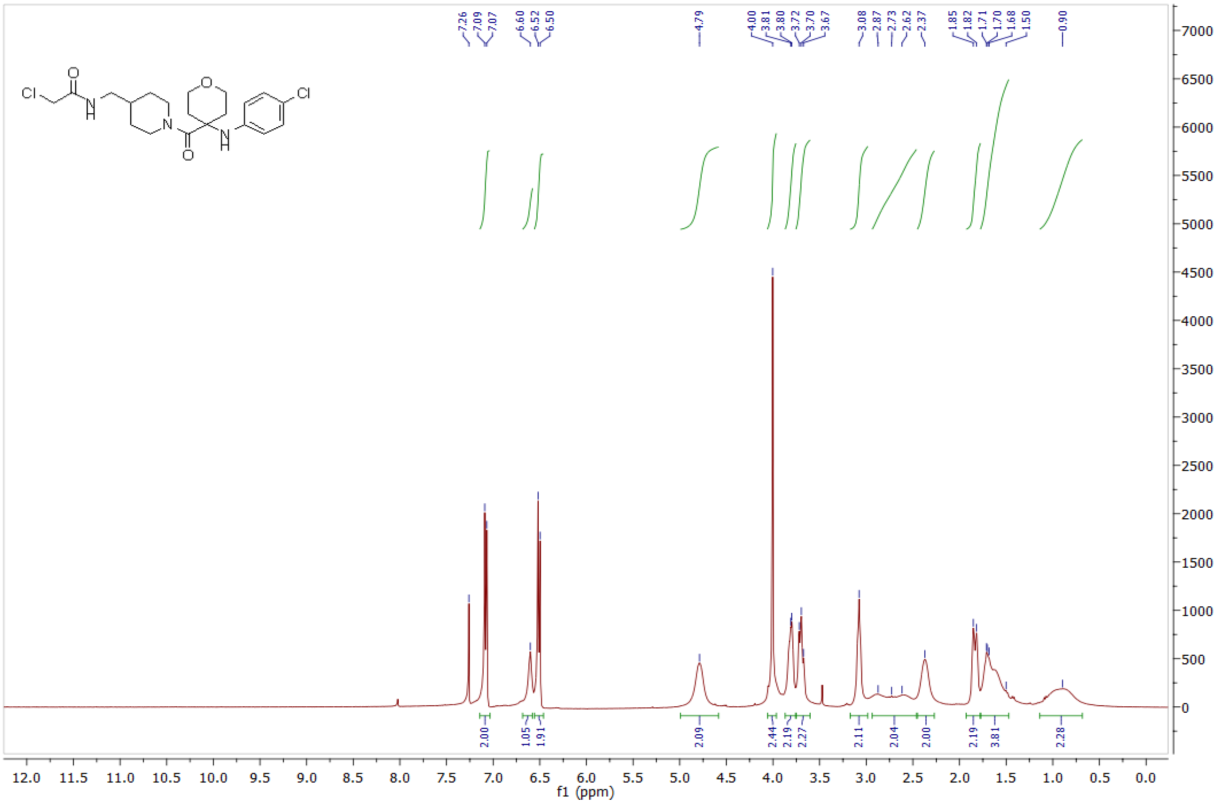

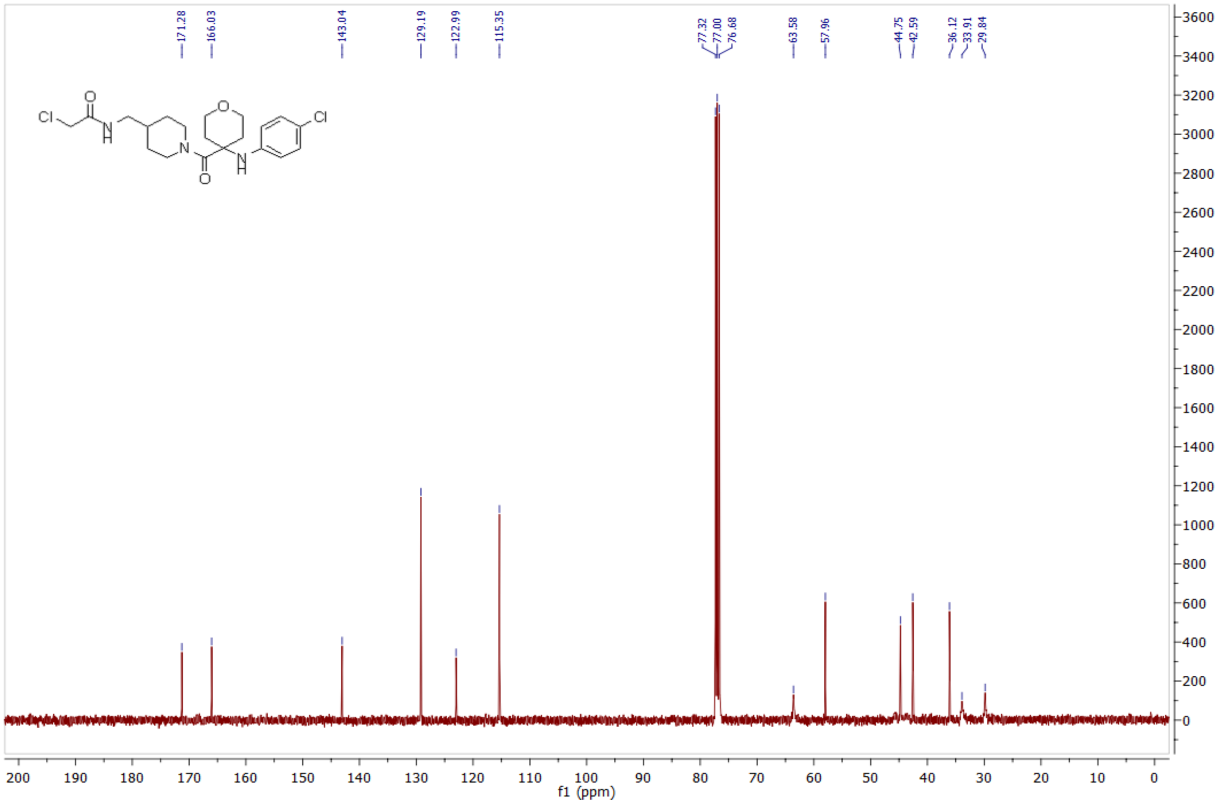

tert-butyl (4-(4-((2-chloroacetamido)methyl)piperidine-1-carbonyl)-4-((4-chlorophenyl)amino) cyclohexyl) carbamate **(136) 1076393**

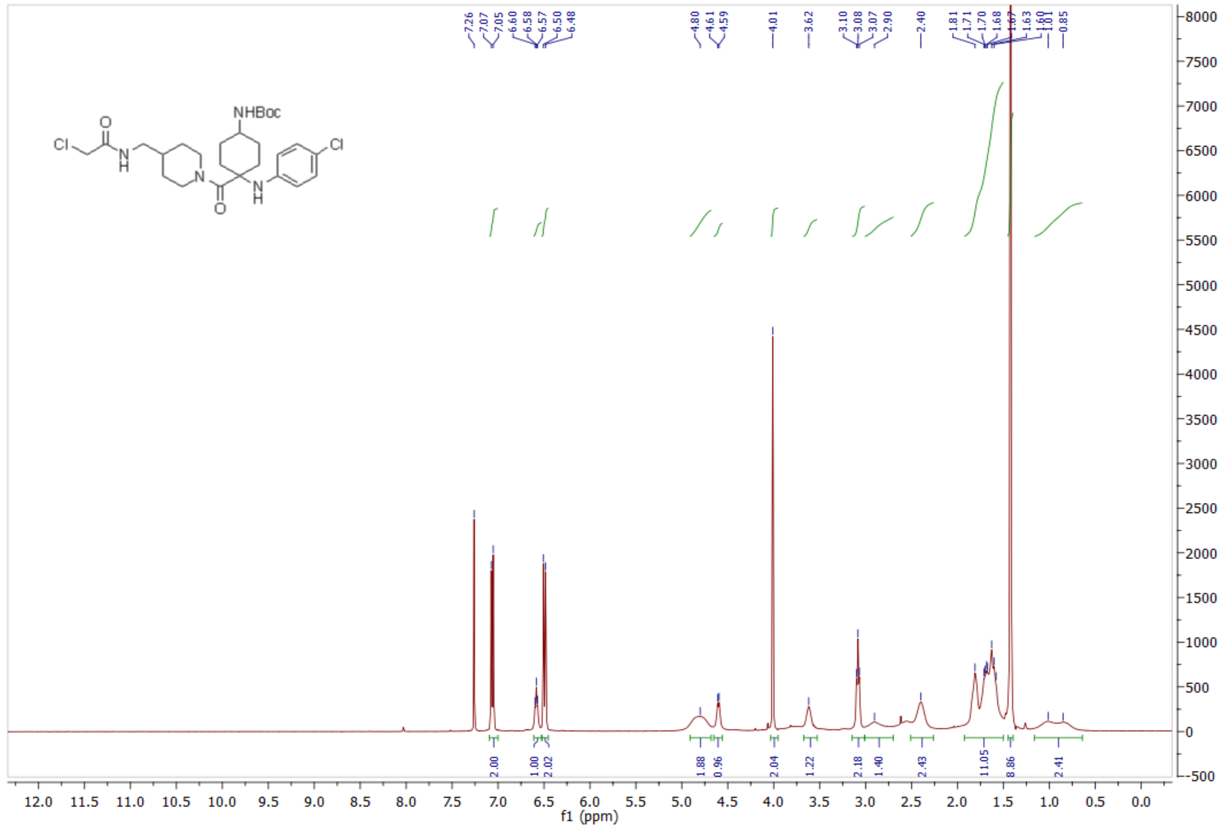

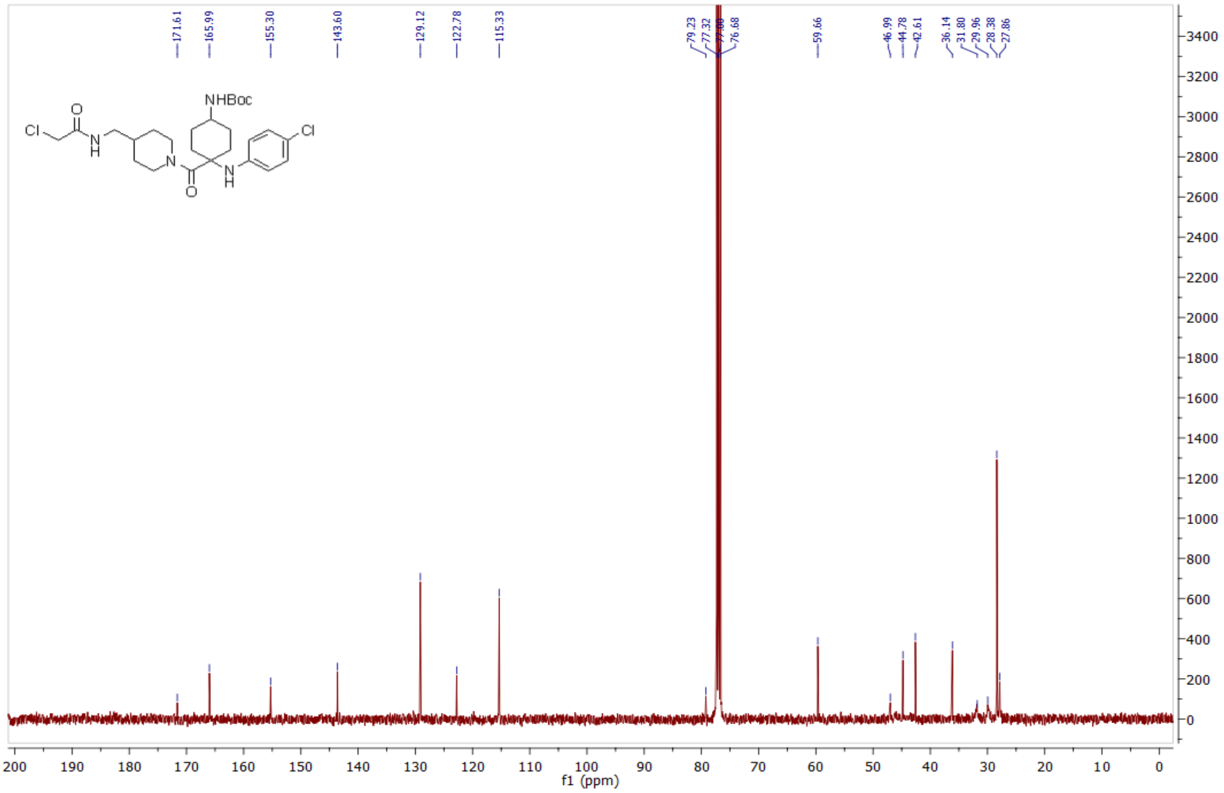

N-((1-(4-amino-1-((4-chlorophenyl)amino)cyclohexane-1-carbonyl)piperidin-4-yl)methyl)-2-chloroacetamide hydrochloride **(137) 1076394**

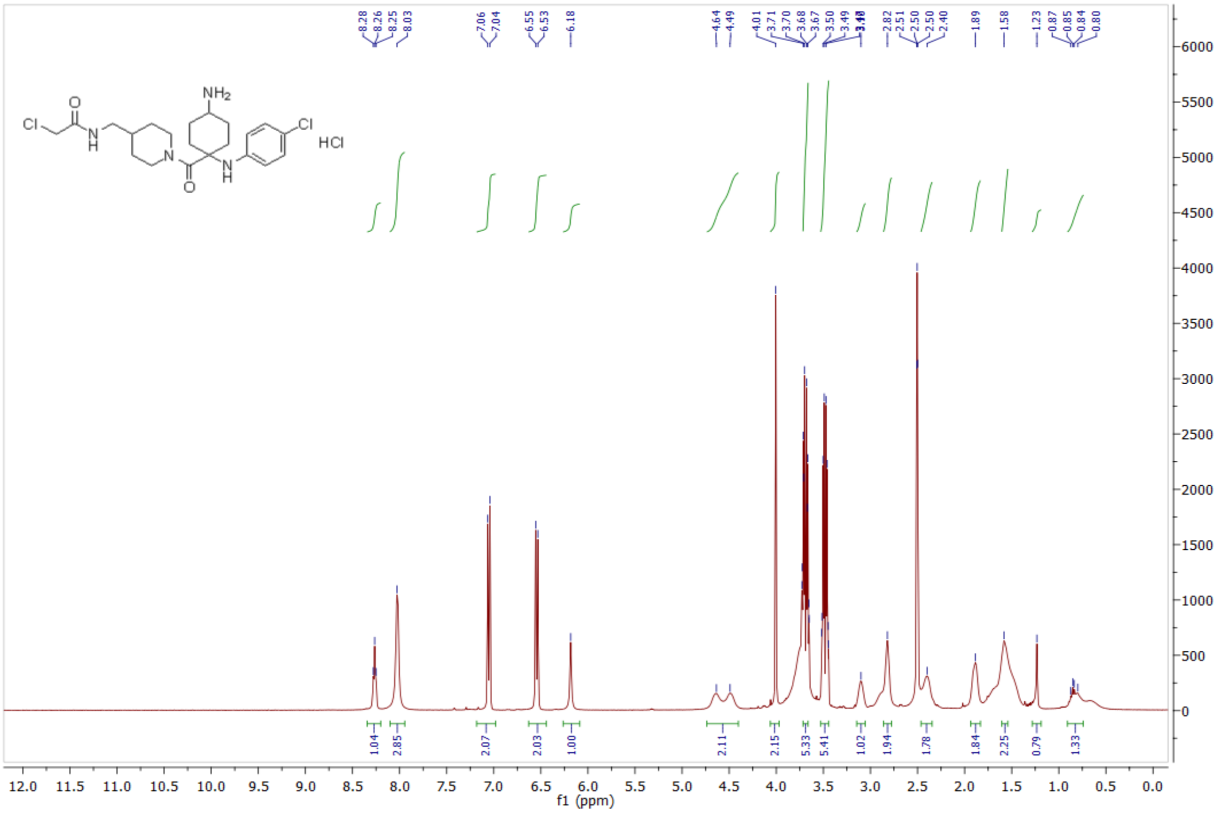

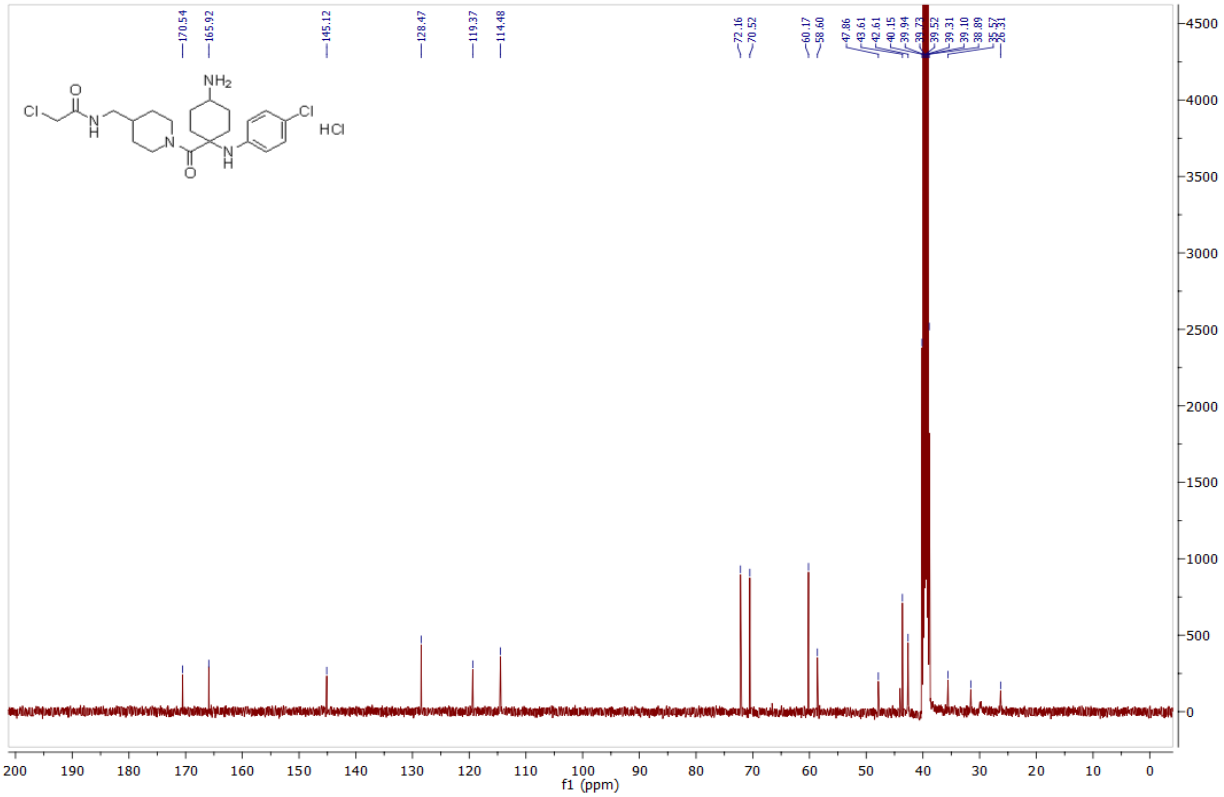

Tert-butyl 4-(2-(2-chloroacetamide)ethyl)piperidine-1-carboxylate **(144)**

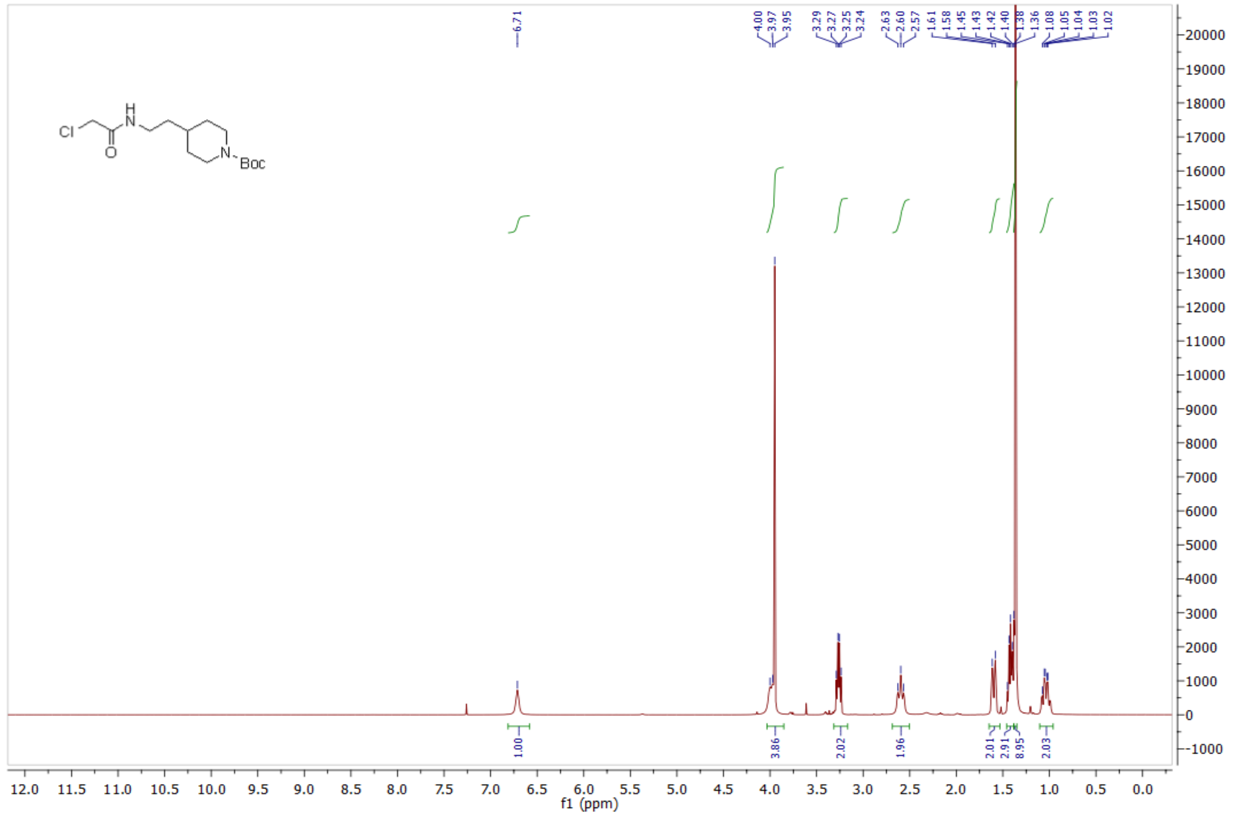

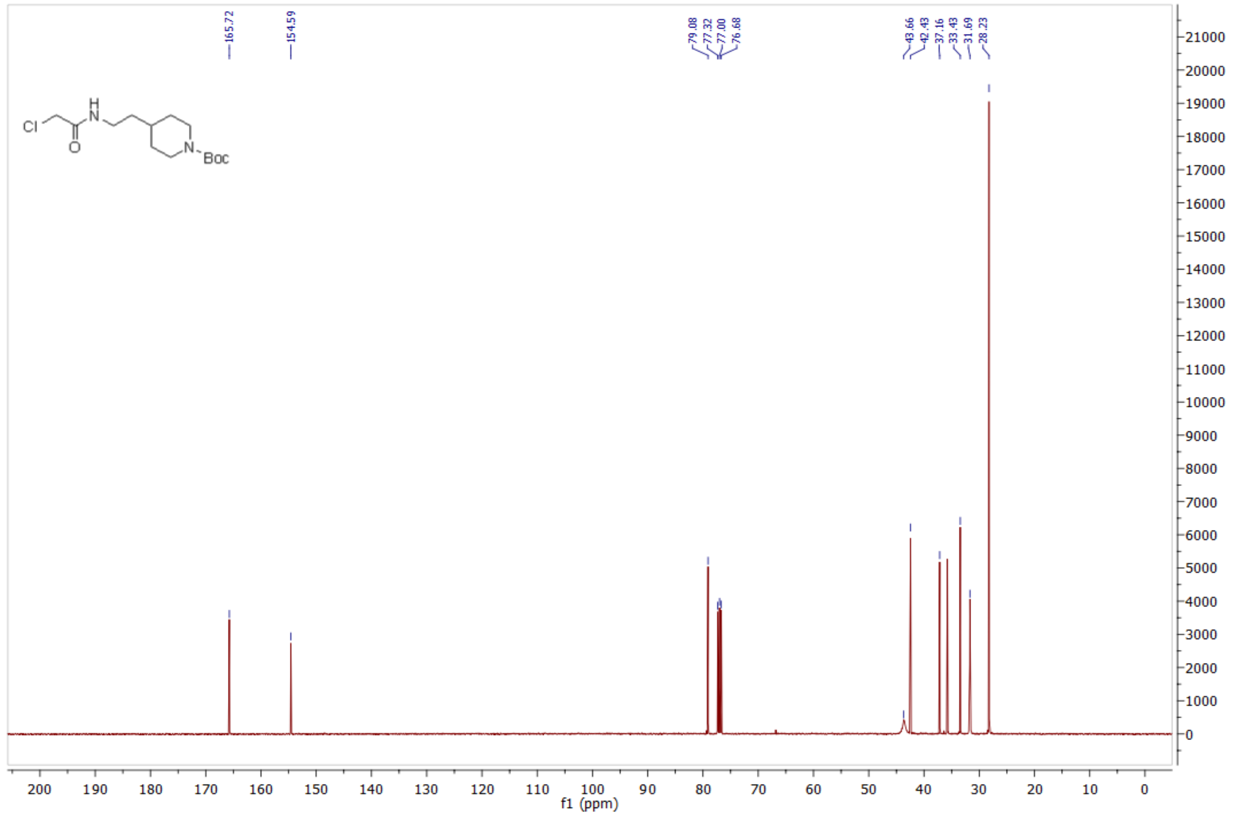

Tert-butyl 4-(3-(2-chloroacetamido)propyl)piperidine-1-carboxylate **(145)**

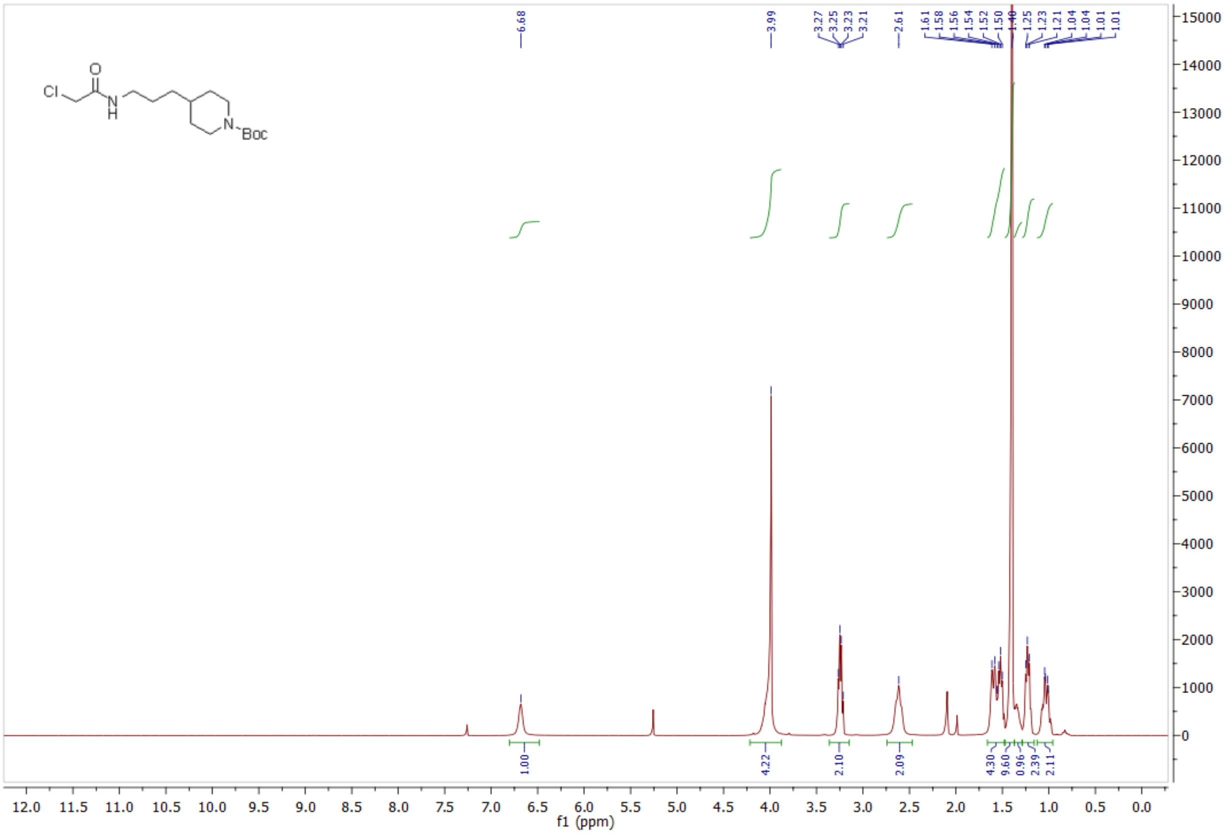

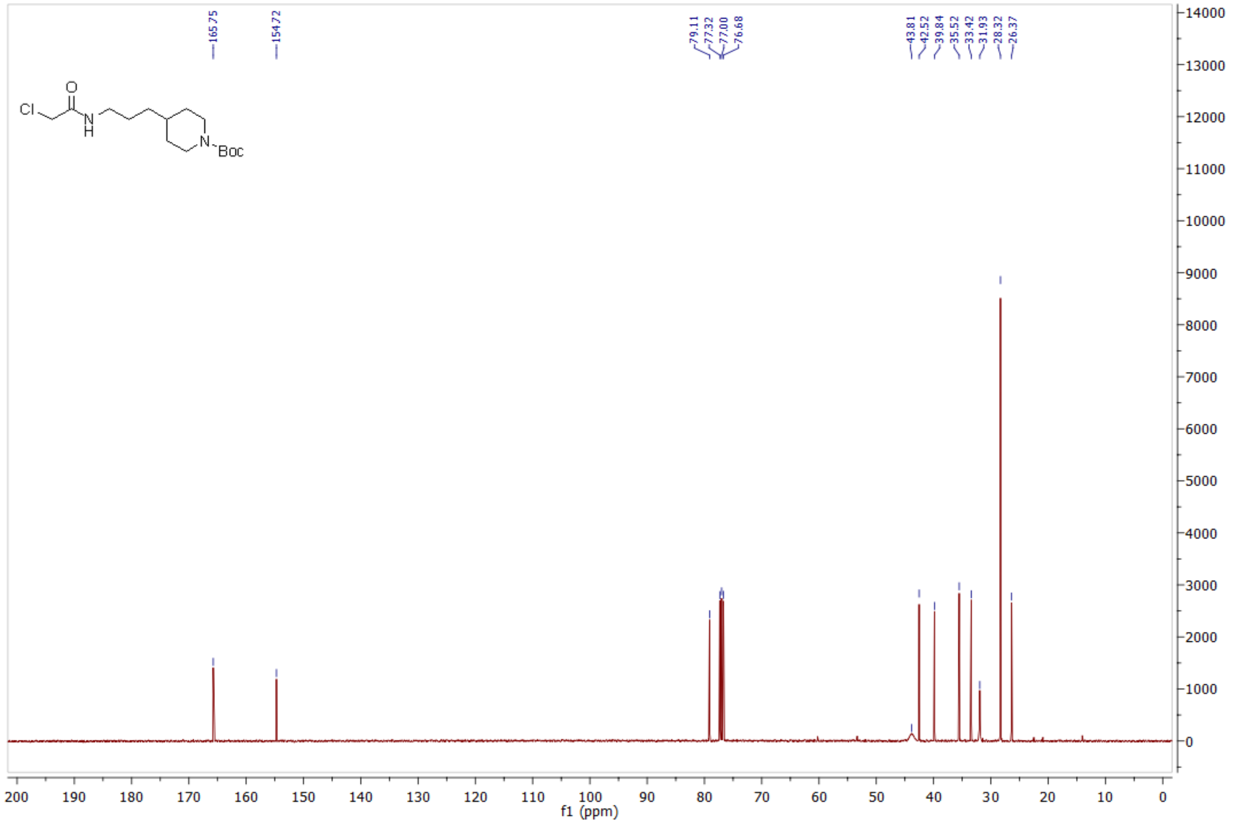

2-chloro-N-(2-(1-(4-((4-chlorophenyl)amino)tetrahydro-2H-pyran-4-carbonyl)piperidin-4-yl)ethyl)acetamide **(148)**
1080267

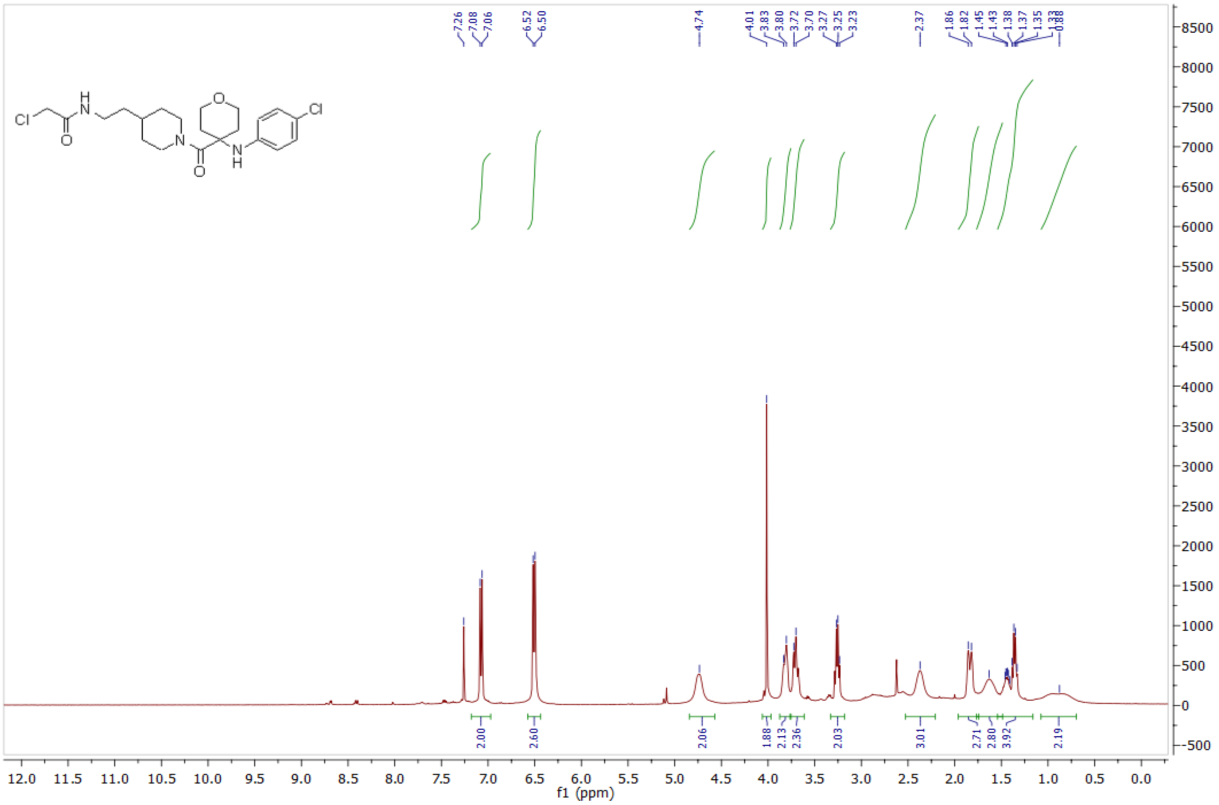

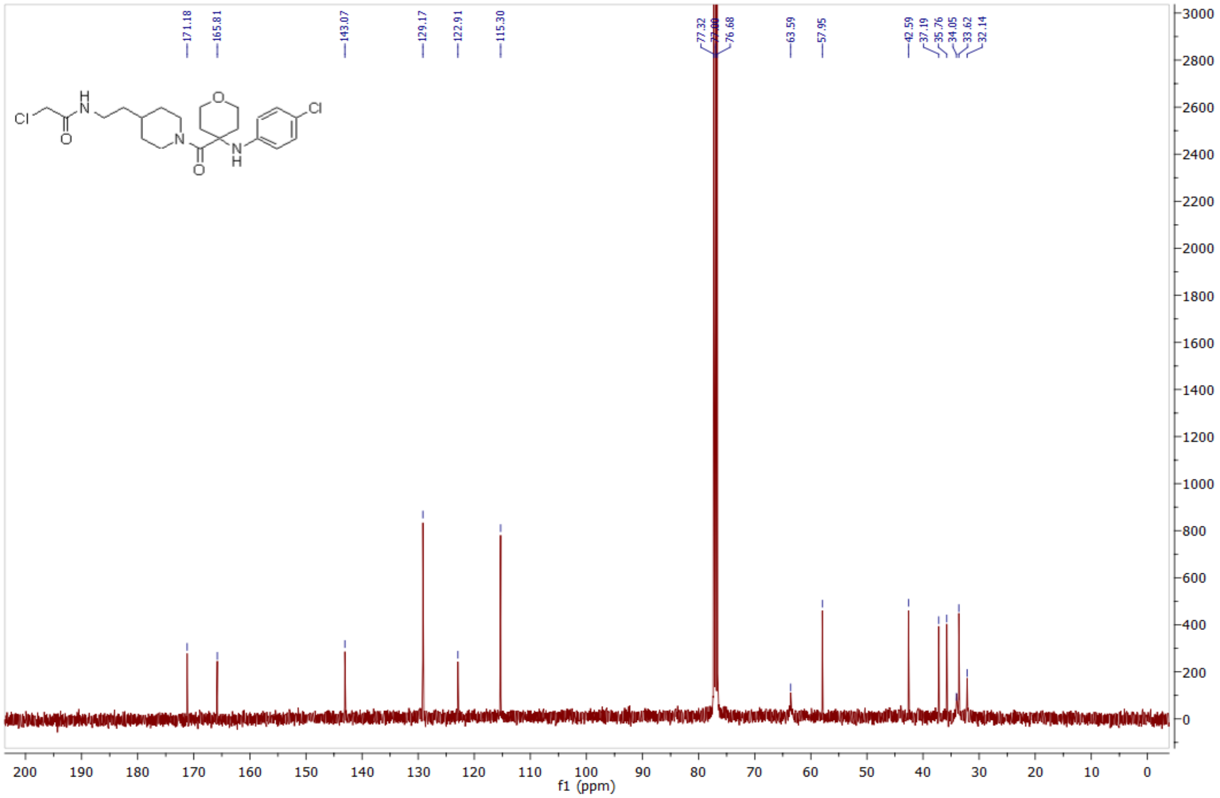

2-chloro-N-(3-(1-(4-((4-chlorophenyl)amino)tetrahydro-2H-pyran-4-carbonyl)piperidin-4-yl)propyl)acetamide **(149)**
1075478

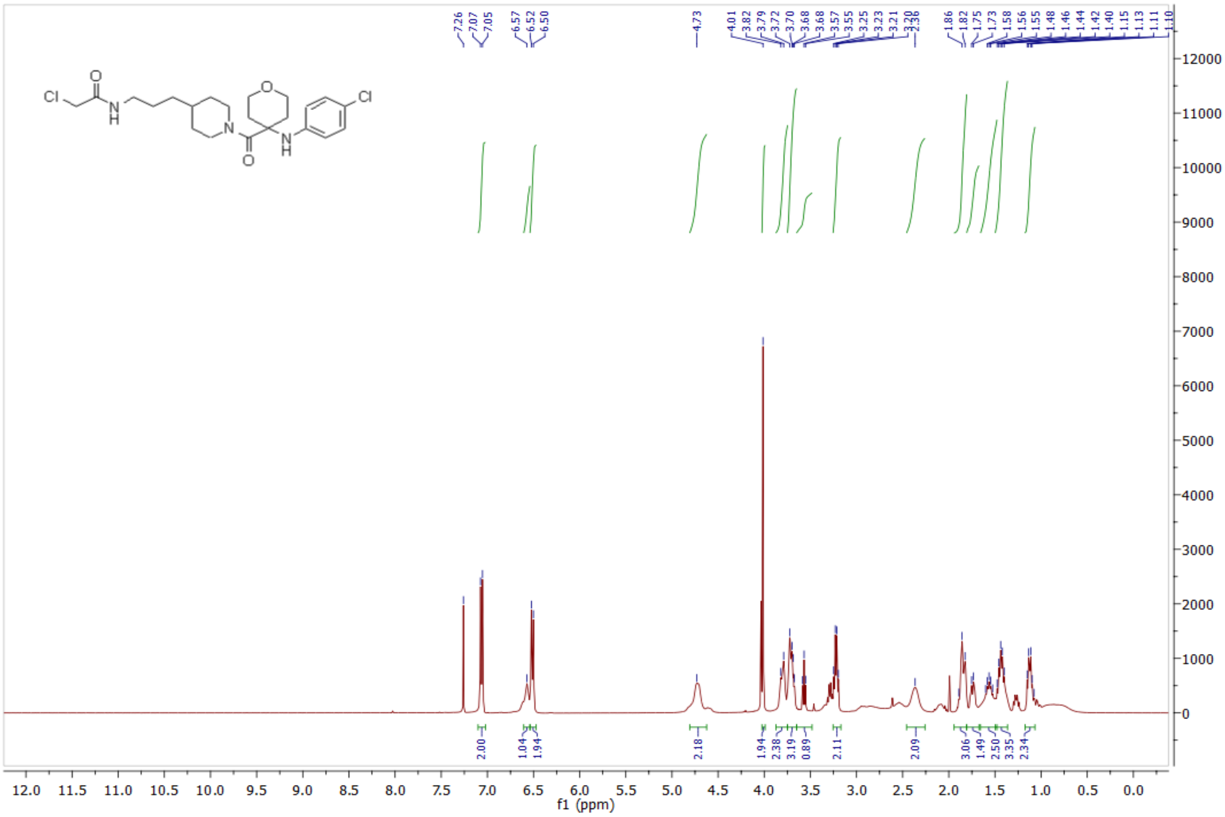

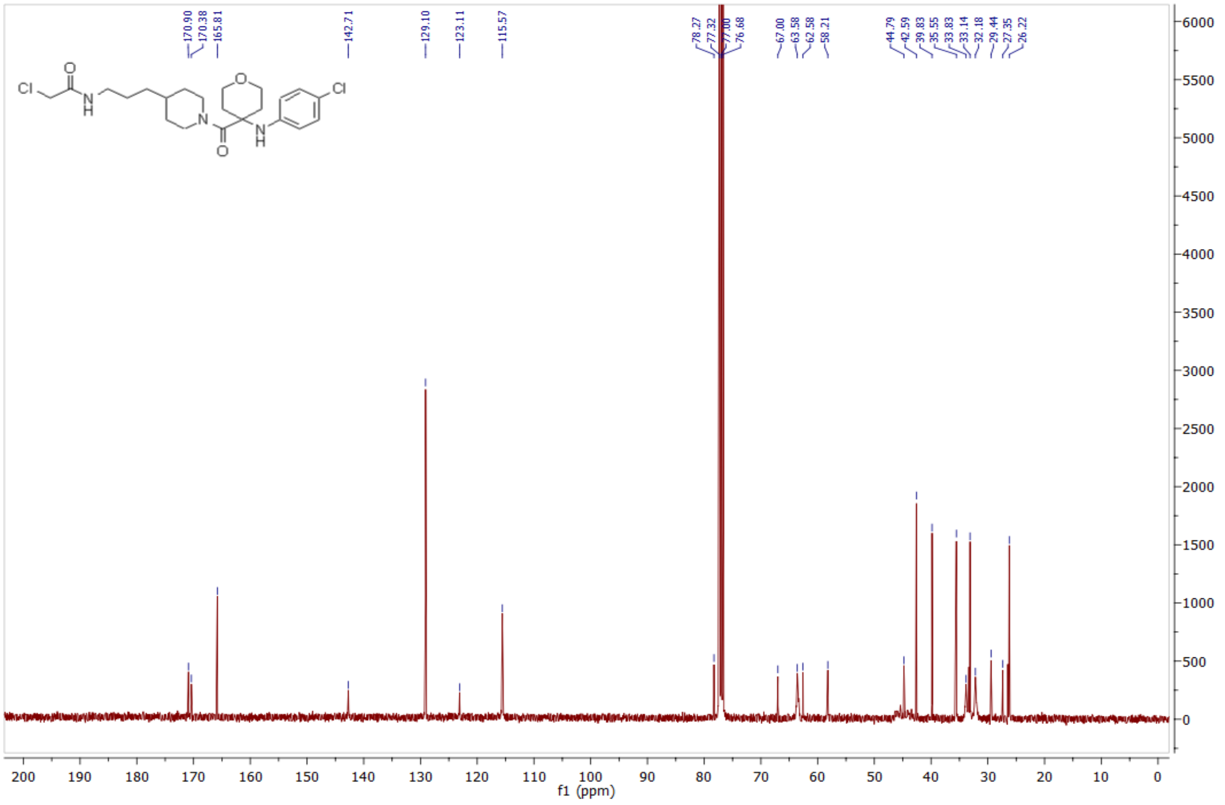

1-*chloro-4-(1-(4-((4-chlorophenyl)amino)tetrahydro-2H-pyran-4-carbonyl)piperidin-4-yl)butan-2-one* **(156)**
1075351

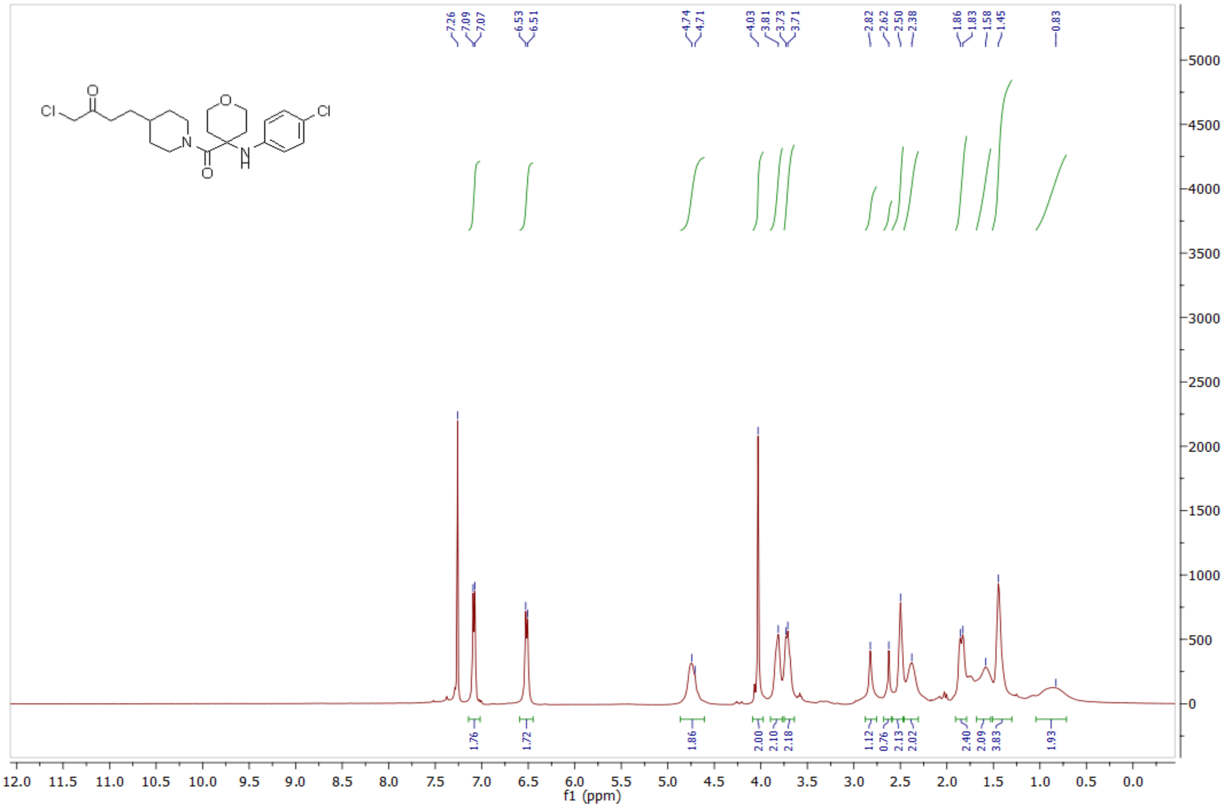

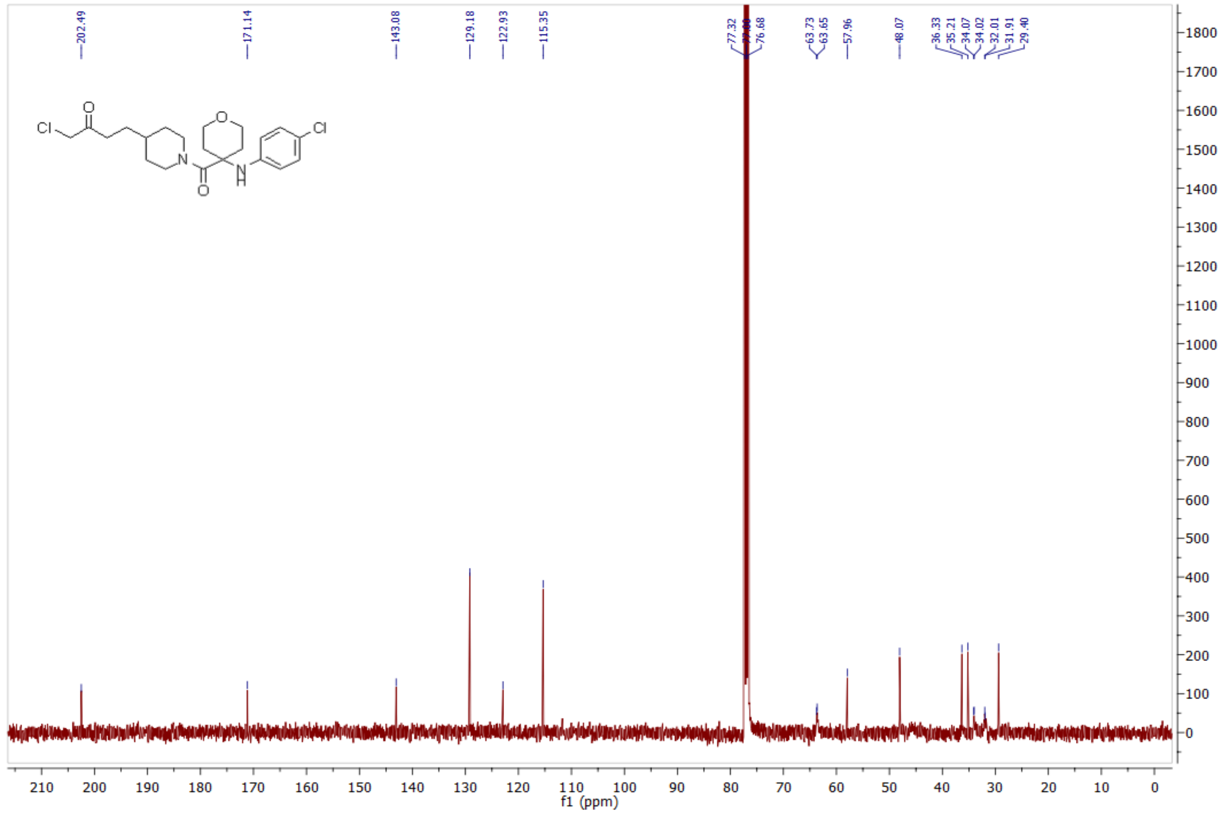

Tert-butyl 2-(2-chloroacetyl)-2,7-diazaspiro[3.5]nonane-7-carboxylate **(158)**

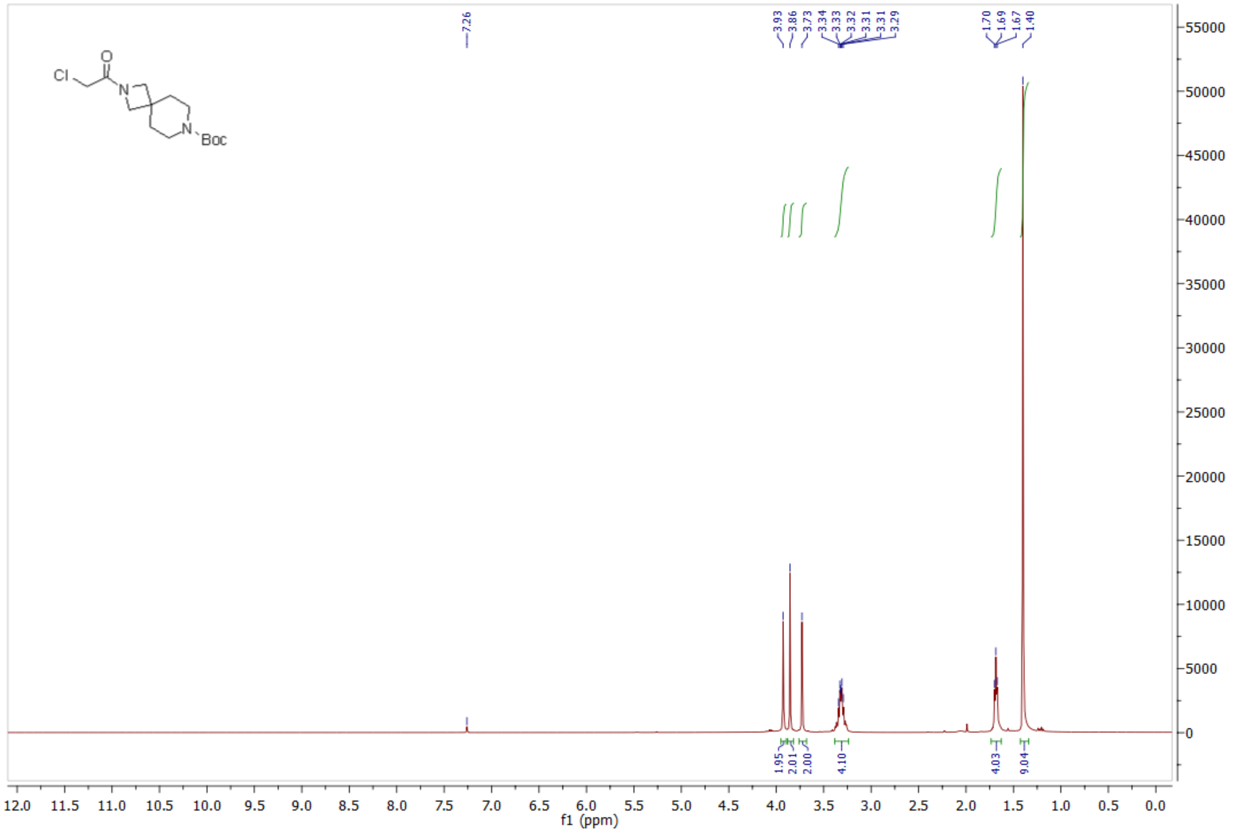

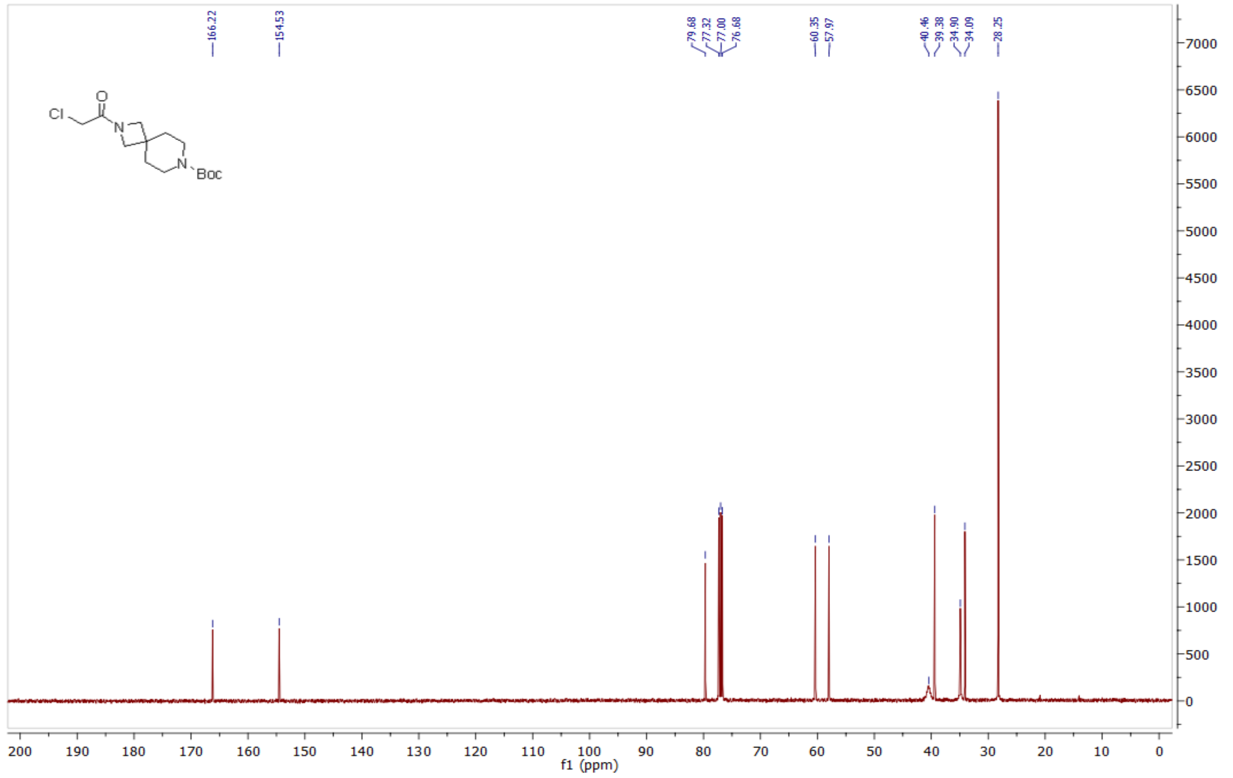

2-chloro-1-(7-(4-((4-chlorophenyl)amino)tetrahydro-2H-pyran-4-carbonyl)-2,7-diazaspiro[3.5]nonan-2-yl)ethan-1-one **(160) 1080265**

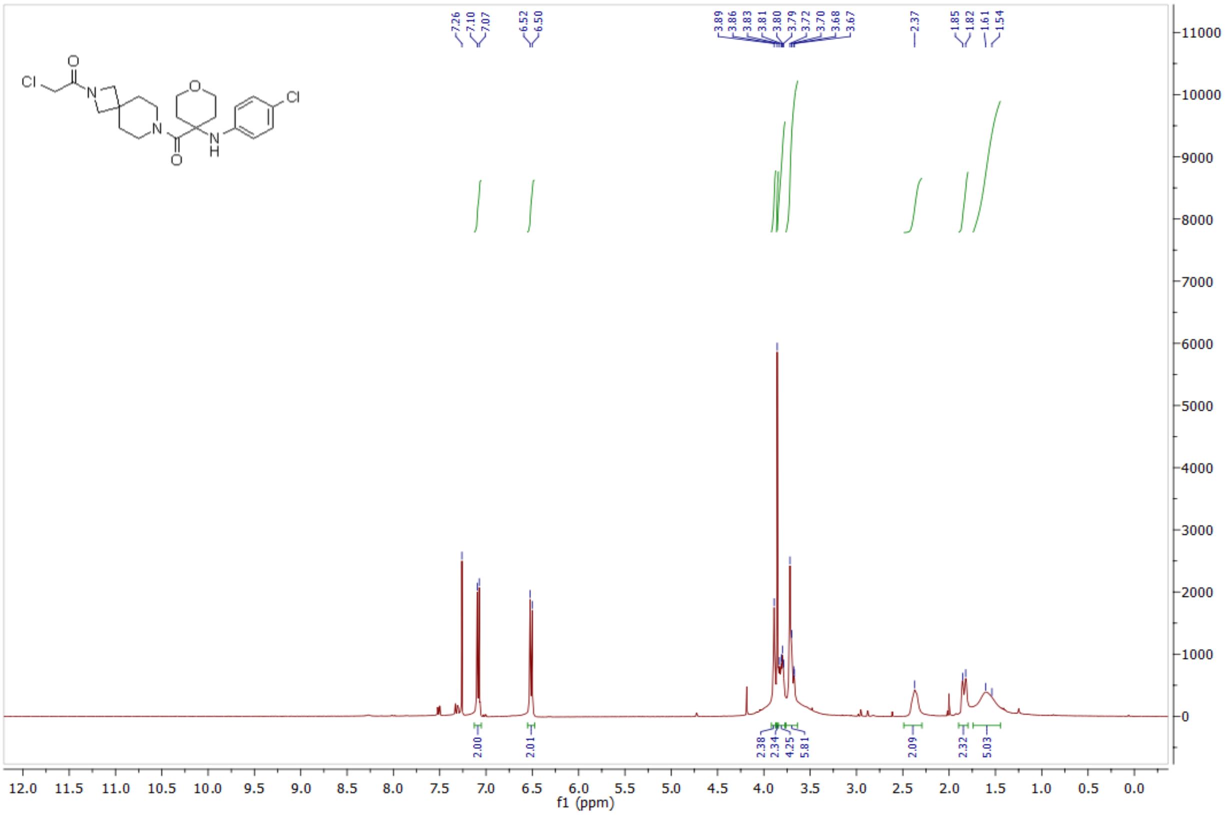

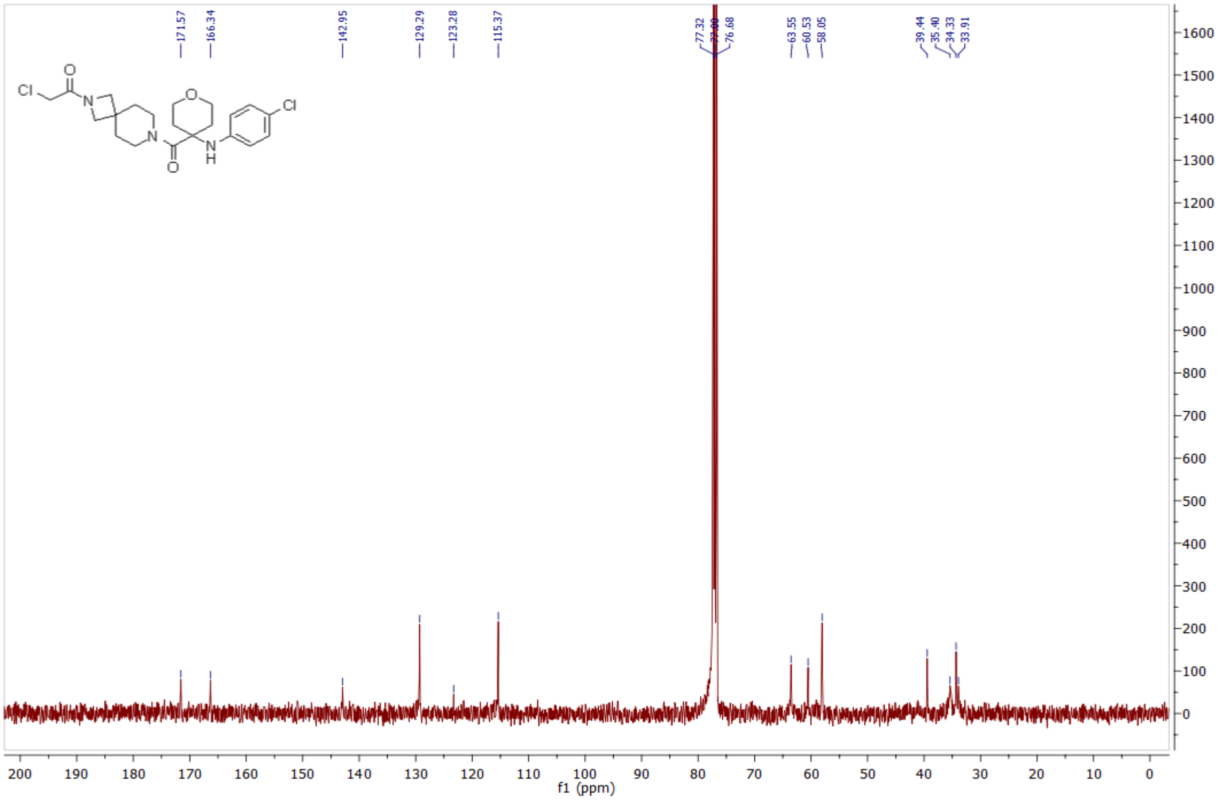

2-chloro-1-(2-(4-((4-chlorophenyl)amino)tetrahydro-2H-pyran-4-carbonyl)-2,7-diazaspiro[3.5]nonan-7-yl)ethan-1-one **(161) 1080266**

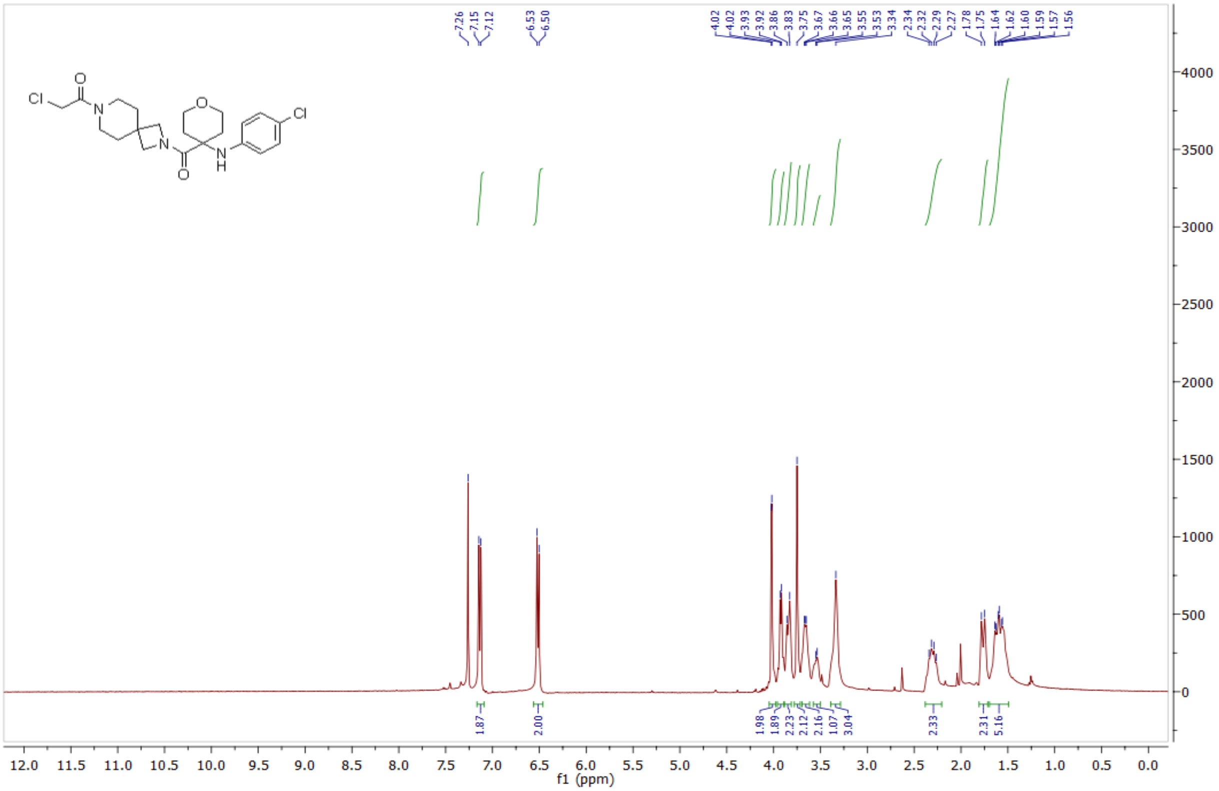

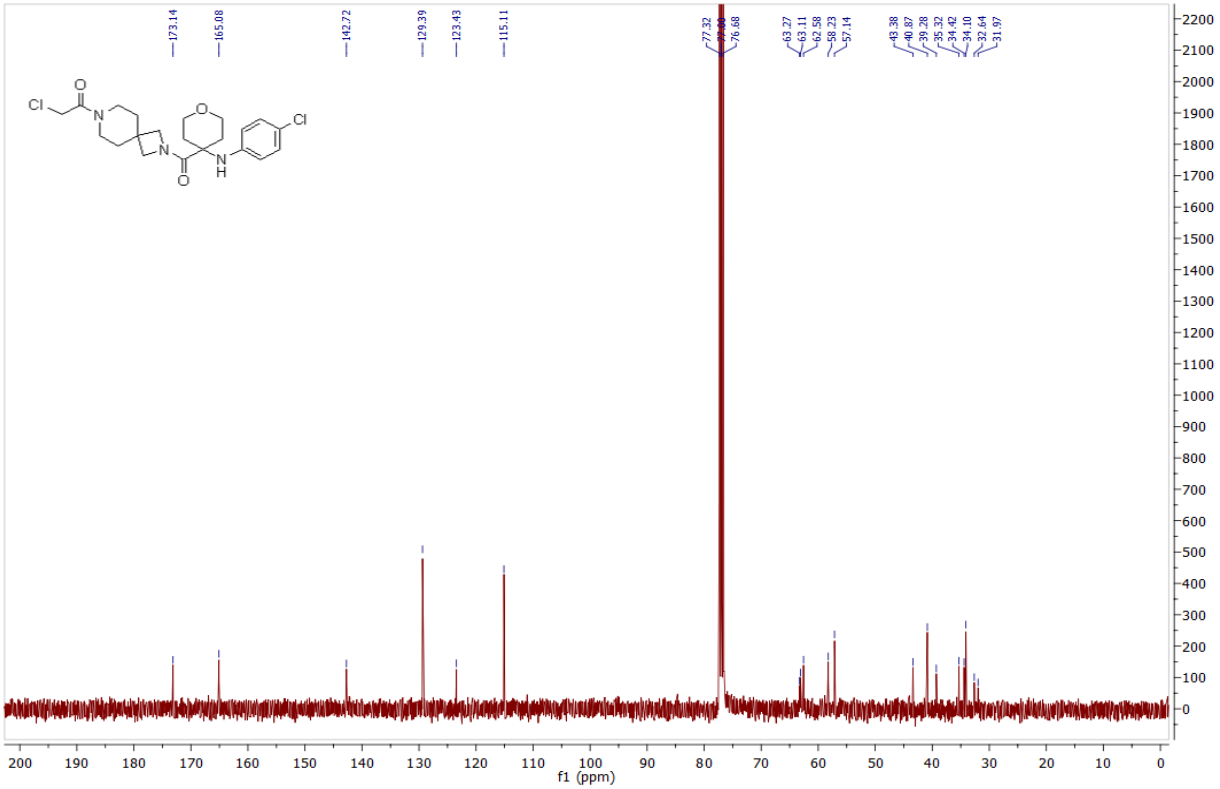

N-(7-(2-chloroacetyl)-7-azaspiro[3.5]nonan-2-yl)-4-((4-chlorophenyl)amino)tetrahydro-2H-pyran-4-carboxamide
(163) 1080294

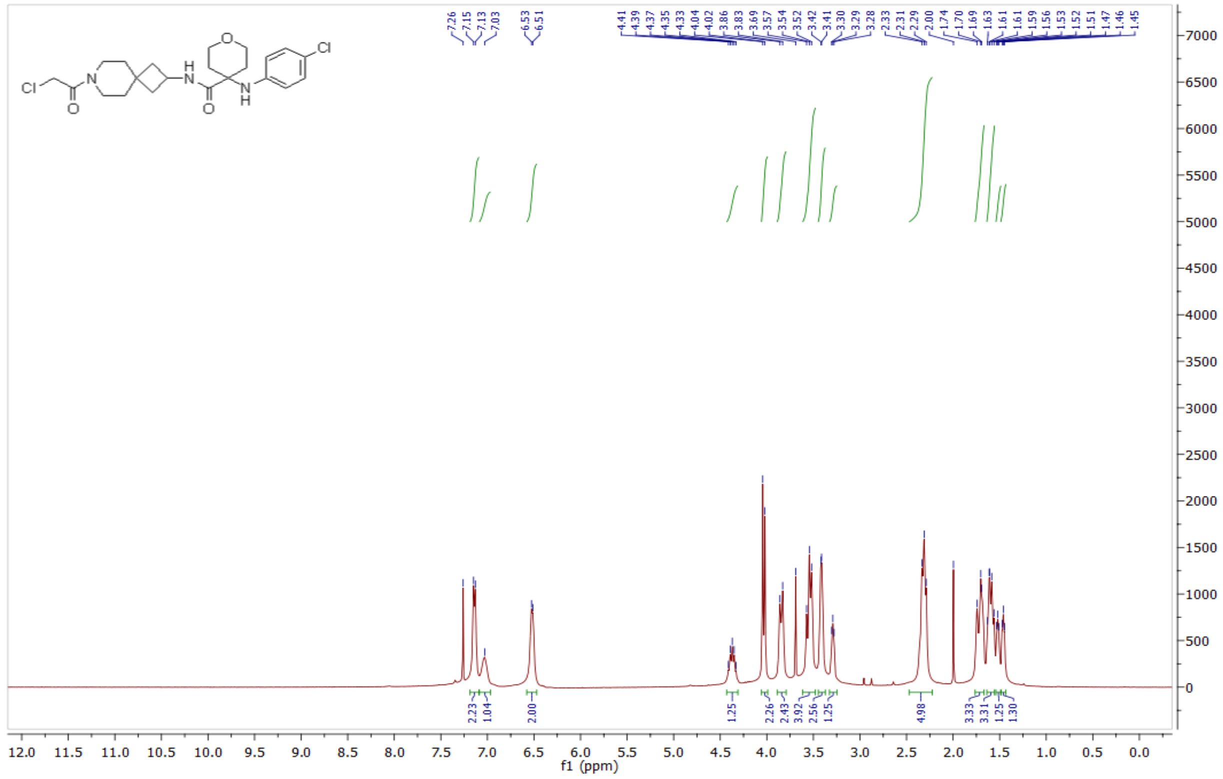

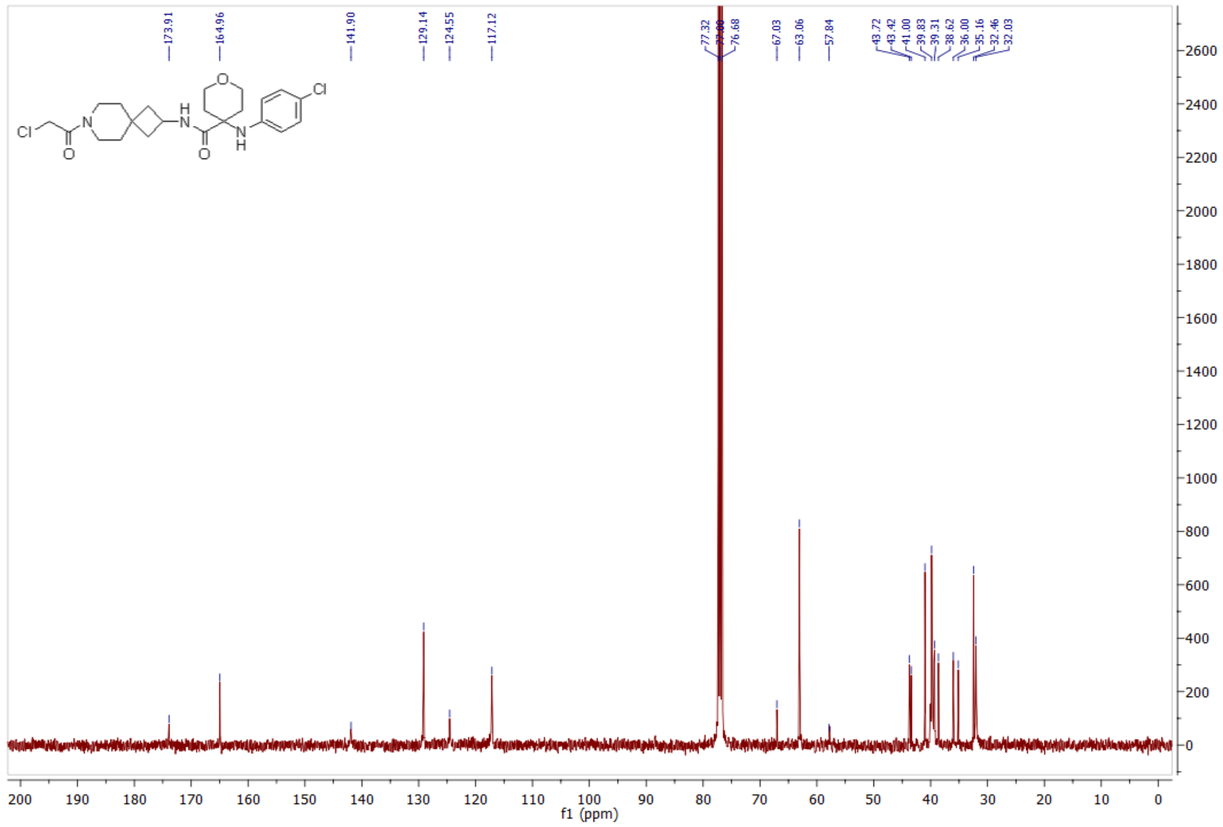

Tert-butyl 2-(2-chloroacetamido)-7-azaspiro[3.5]nonane-7-carboxylate **(164)**

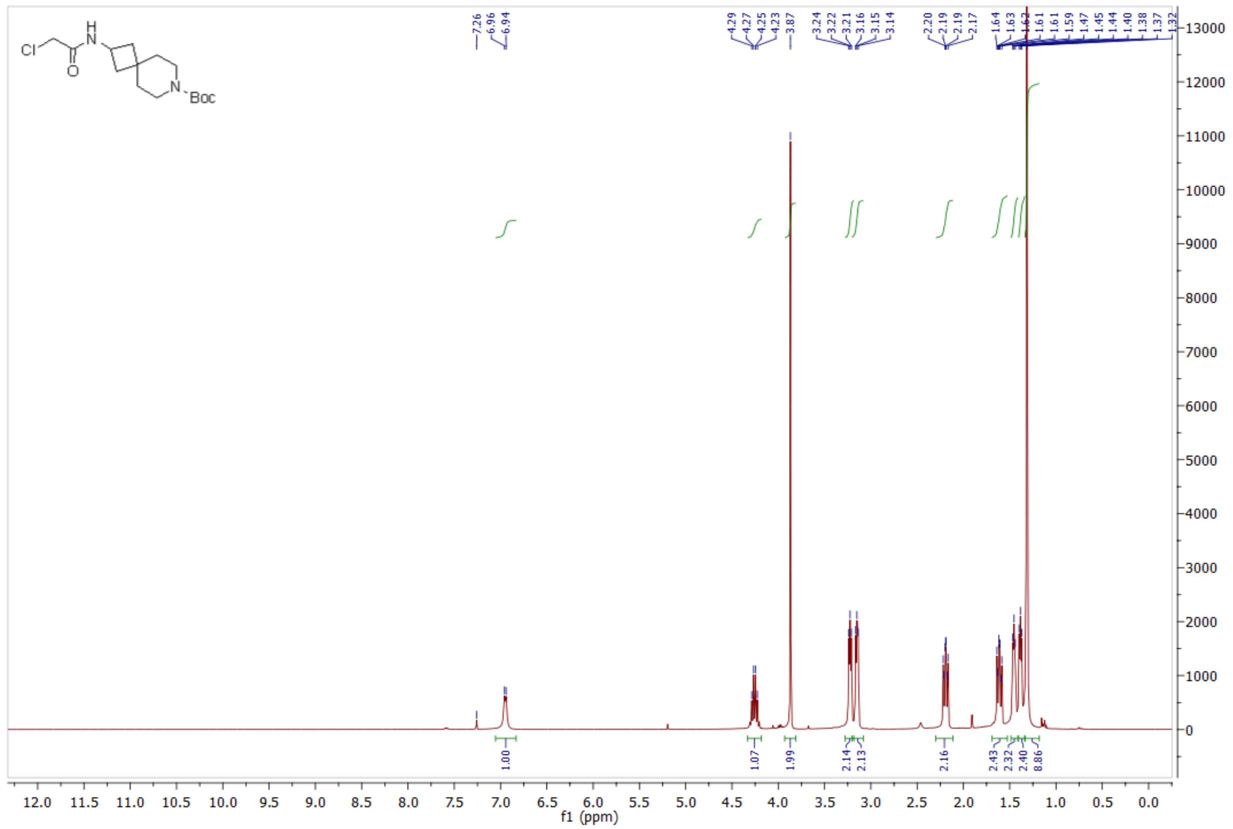

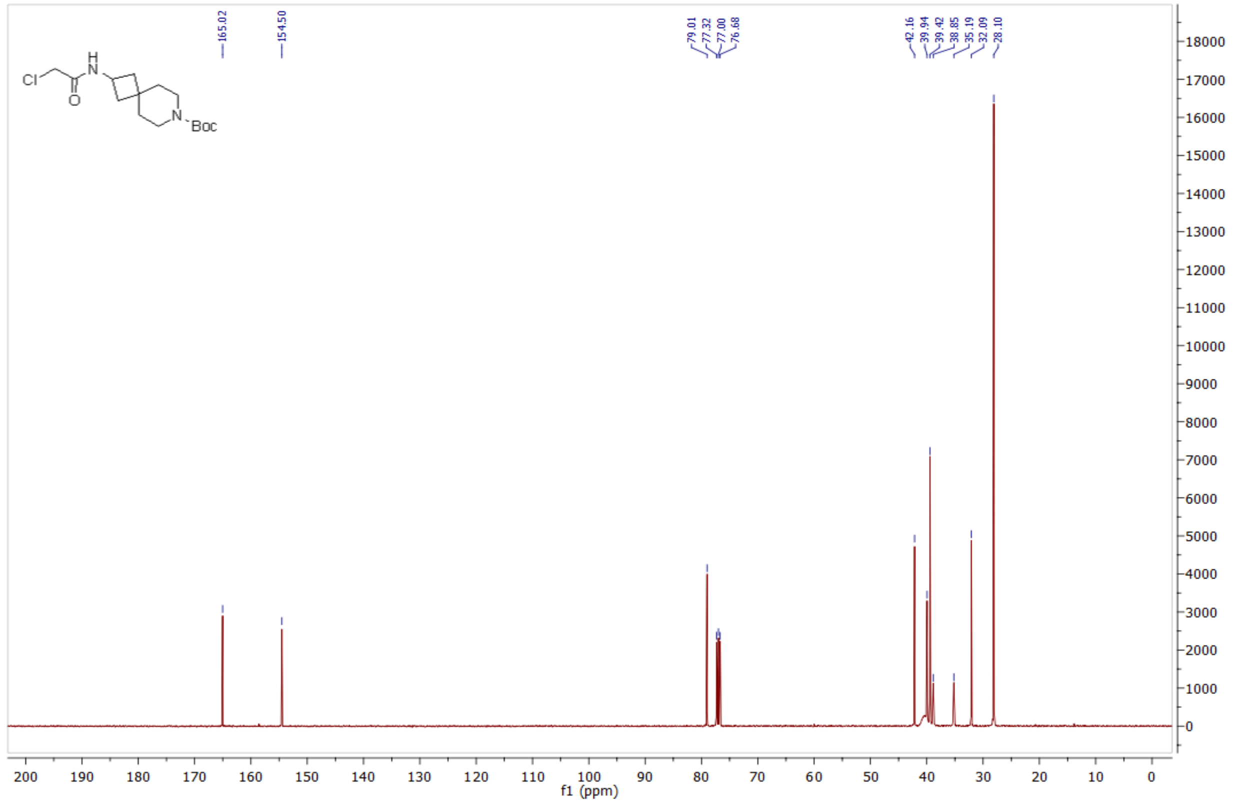

2-chloro-N-(7-(4-((4-chlorophenyl)amino)tetrahydro-2H-pyran-4-carbonyl)-7-azaspiro[3.5]nonan-2-yl)acetamide
(166) 1080295

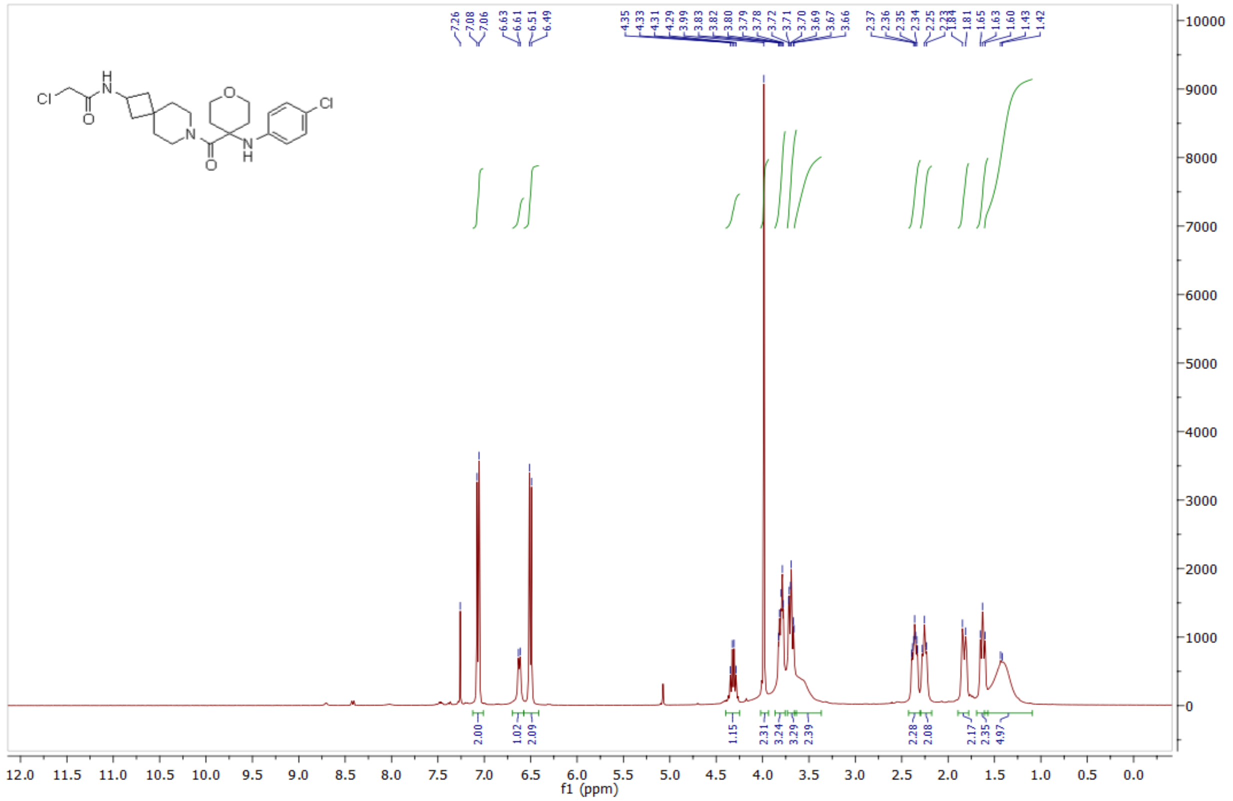

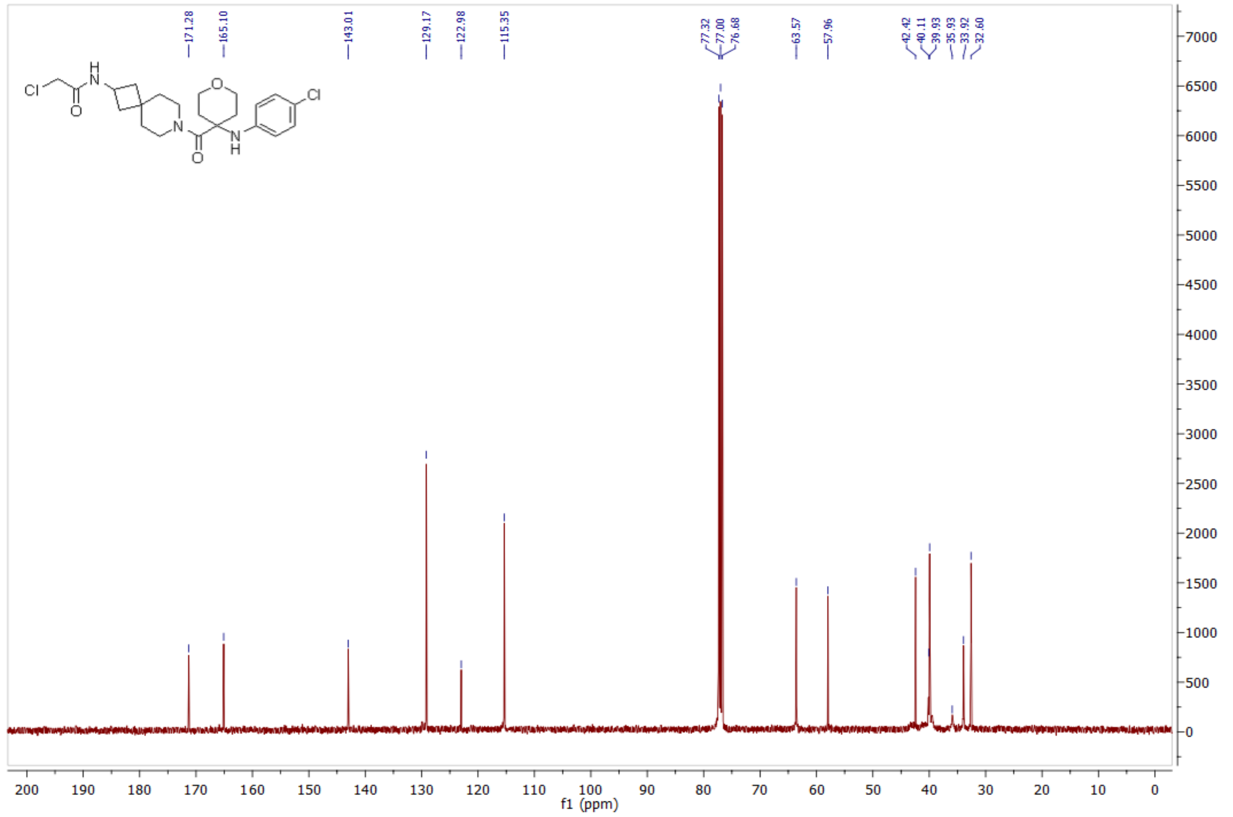

Tert-butyl 9-(2-chloroacetyl)-3,9-diazaspiro[5.5]undecane-3-carboxylate **(168)**

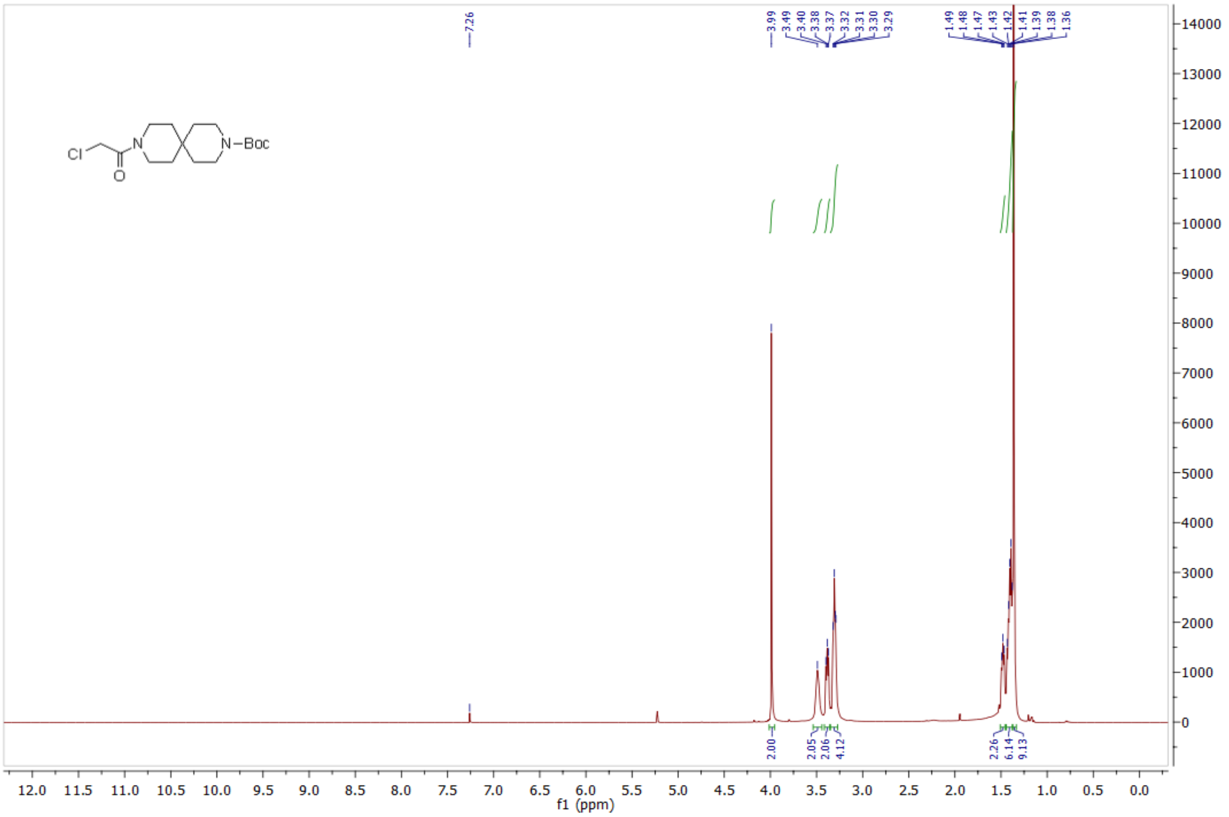

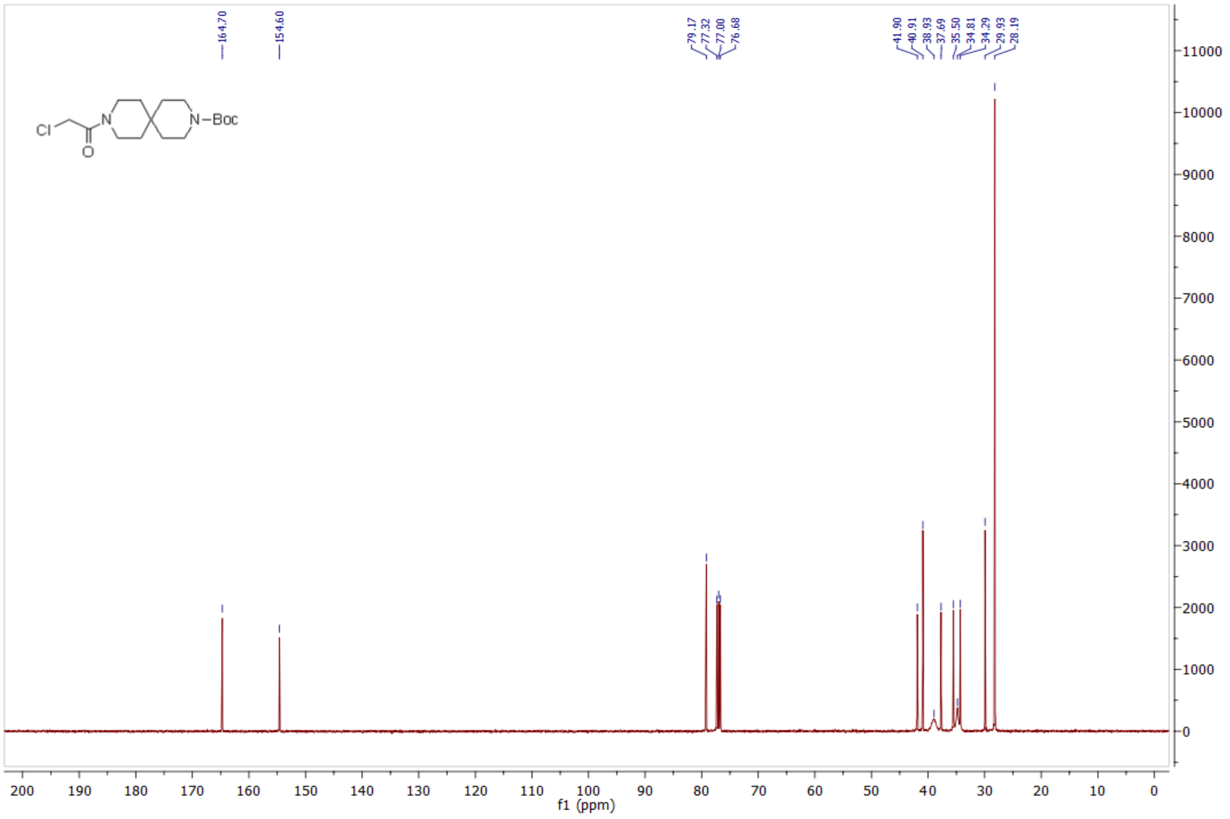

2-chloro-1-(9-(4-((4-chlorophenyl)amino)tetrahydro-2H-pyran-4-carbonyl)-3,9-diazaspiro[5.5] undecan-3-yl)ethan-1-one **(170) 1080296**

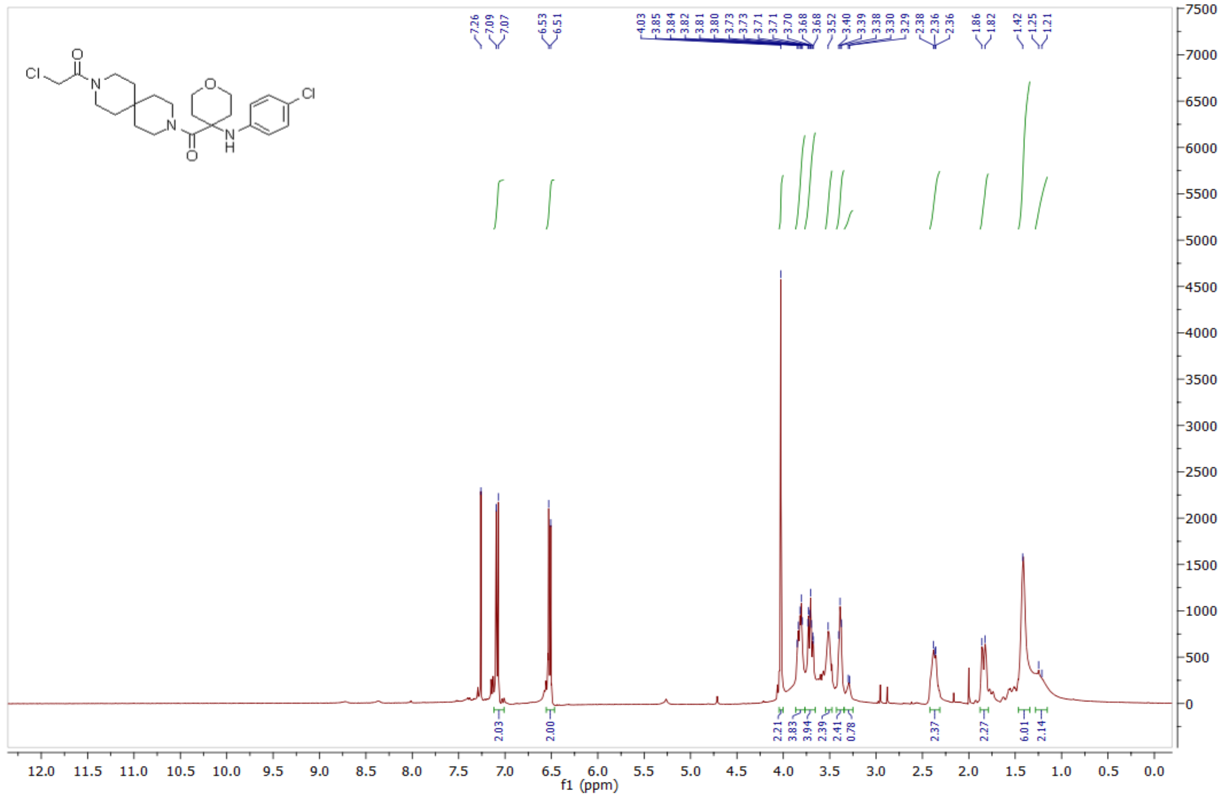

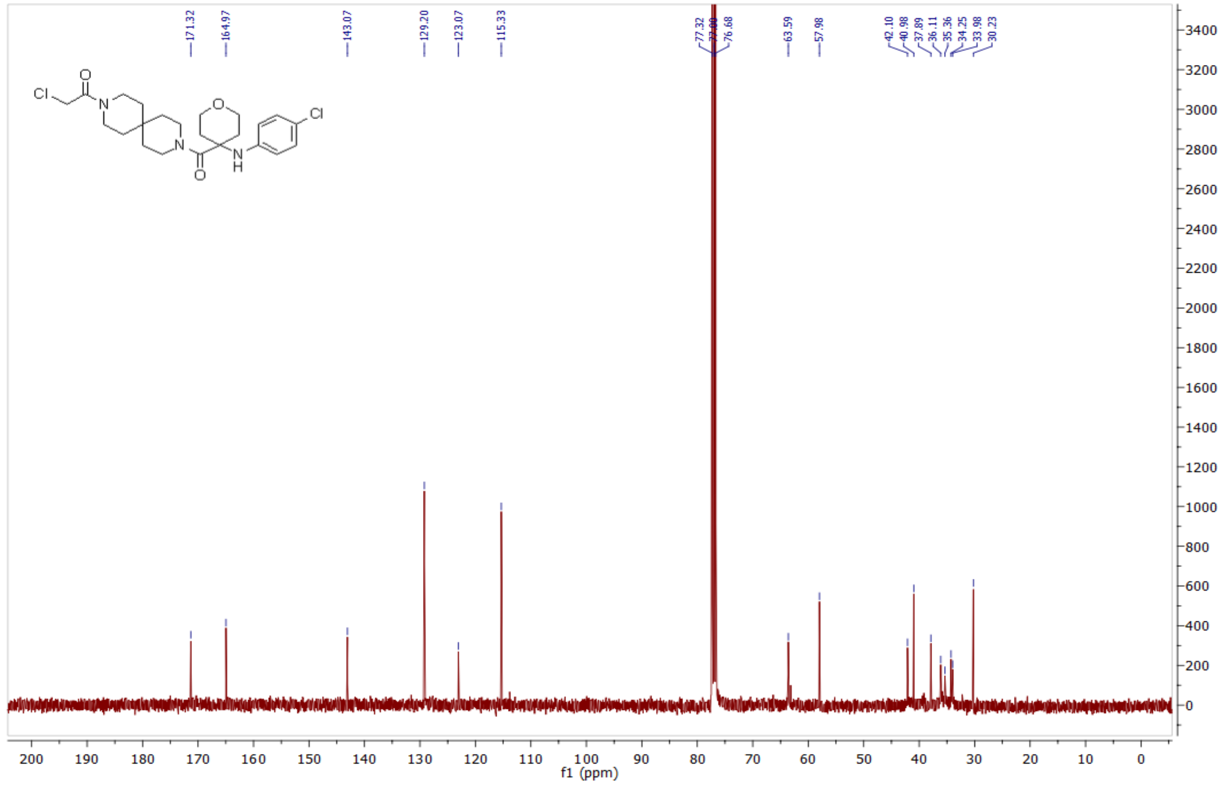

Tert-butyl 2-((2-chloroacetamido)methyl)-7-azaspiro[3.5]nonane-7-carboxylate **(172)**

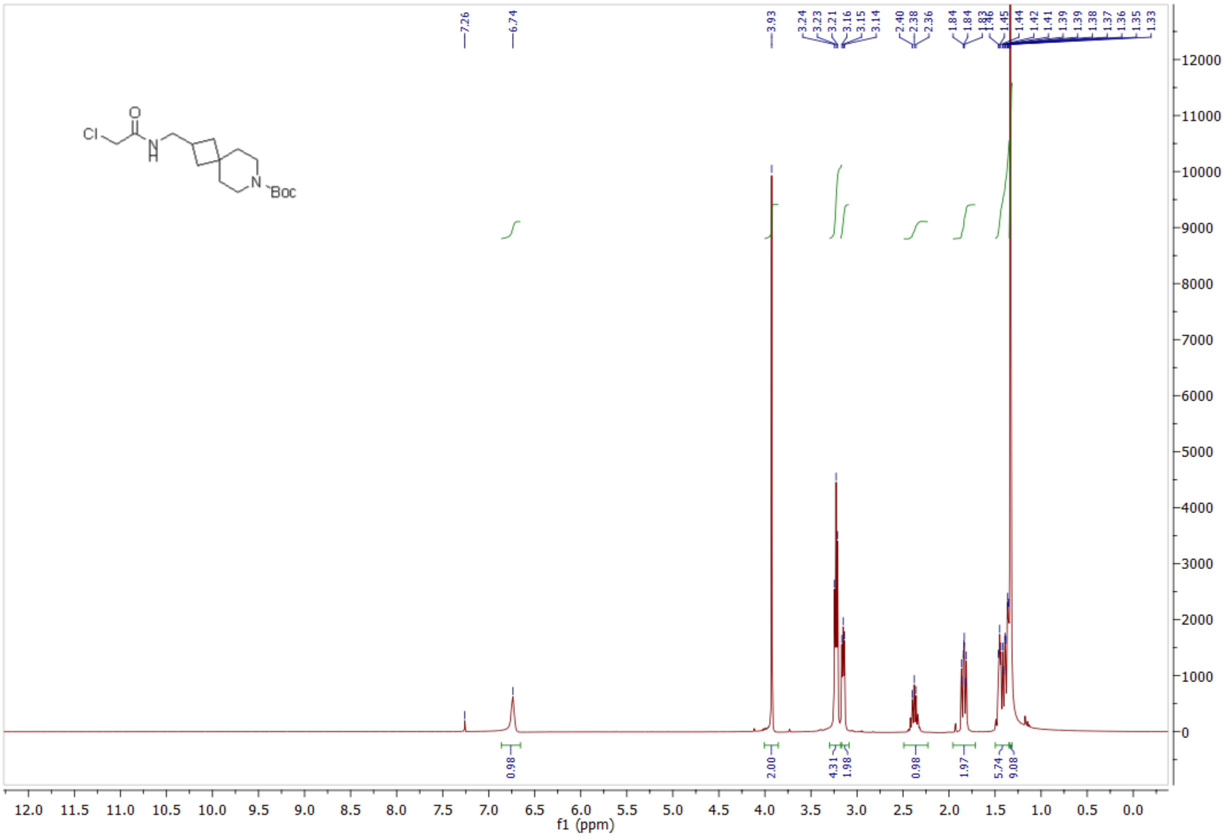

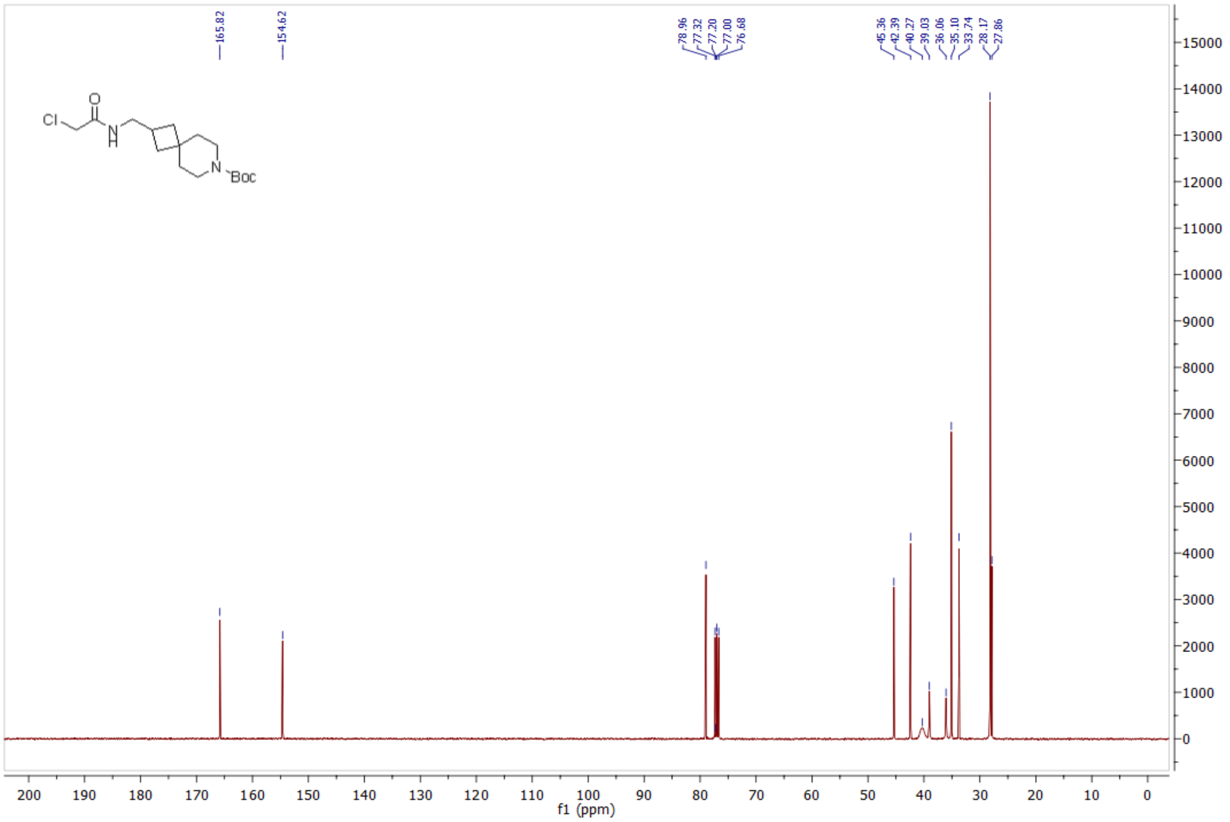

N-((7-(2-chloroacetyl)-7-azaspiro[3.5]nonan-2-yl)methyl)-4-((4-chlorophenyl)amino)tetrahydro-2H-pyran-4-carboxamide **(174) 1080297**

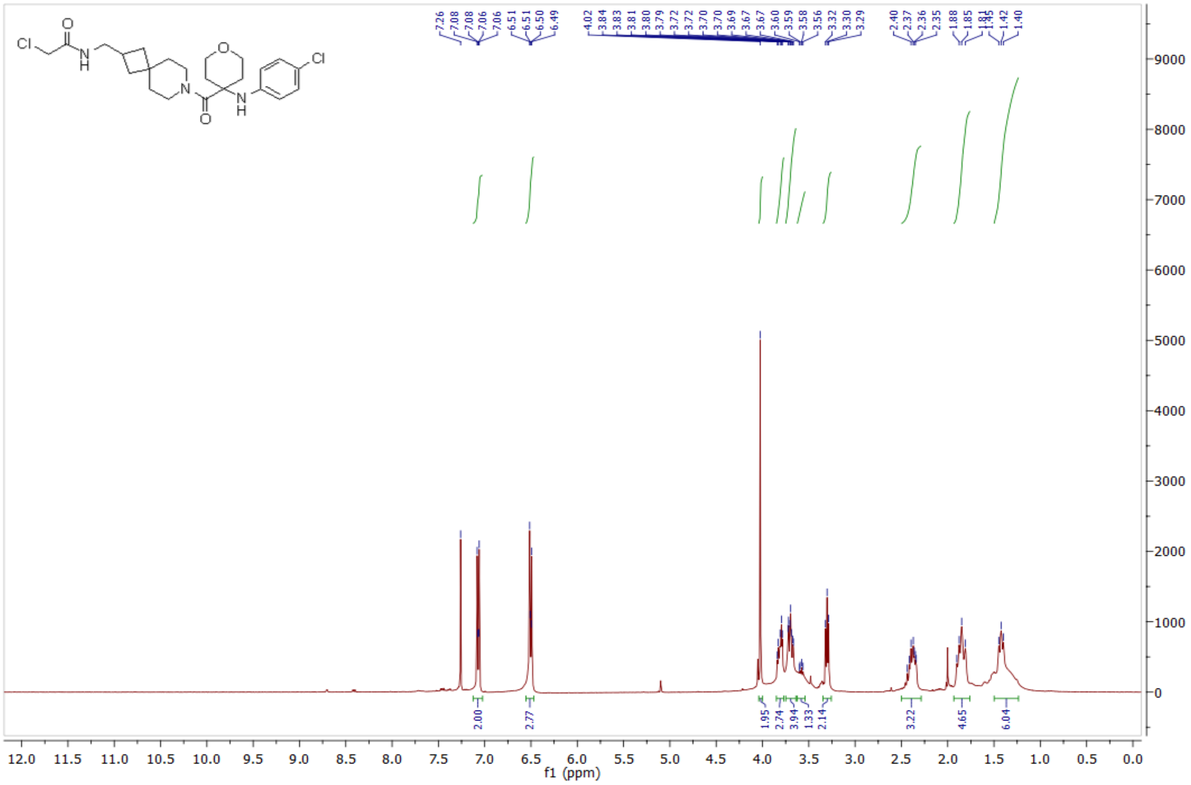

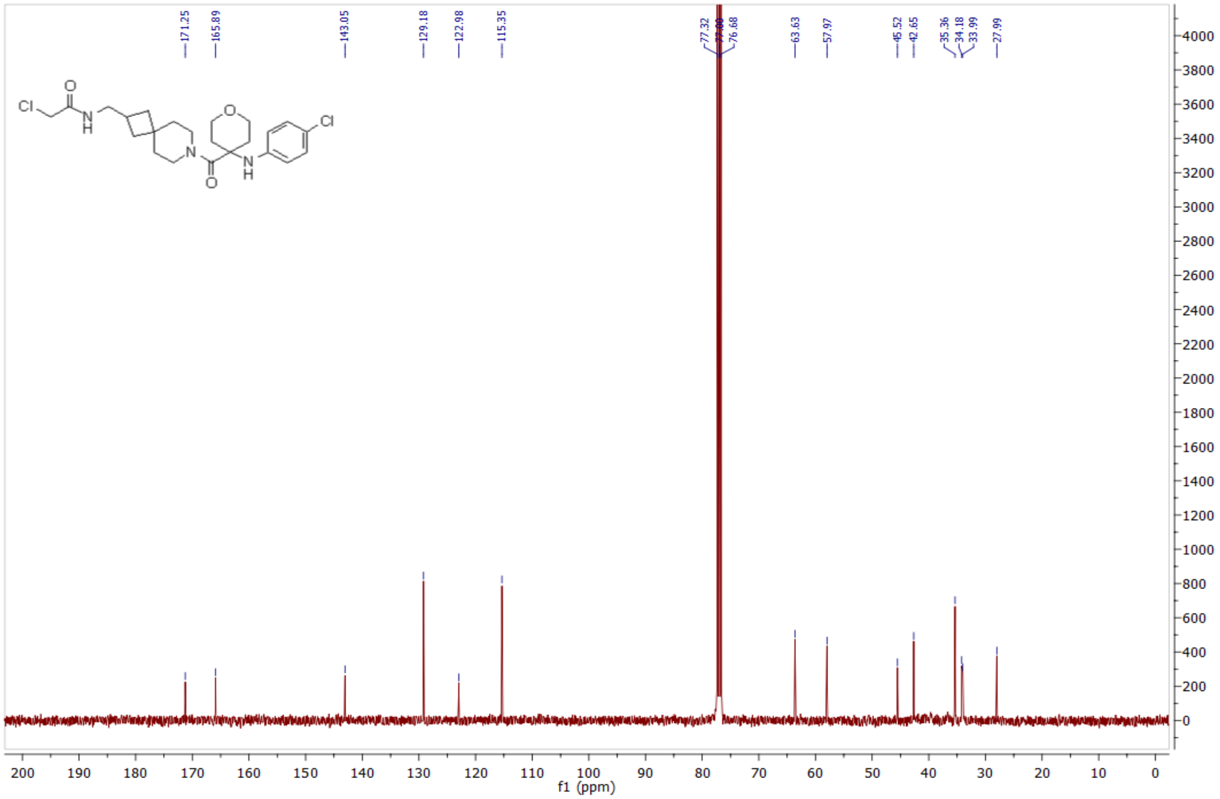

2-chloro-N-(7-(4-((4-chlorophenyl)amino)tetrahydro-2H-pyran-4-carbonyl)-7-azaspiro[3.5]nonan-2-yl)acetamide
(175) 1080298

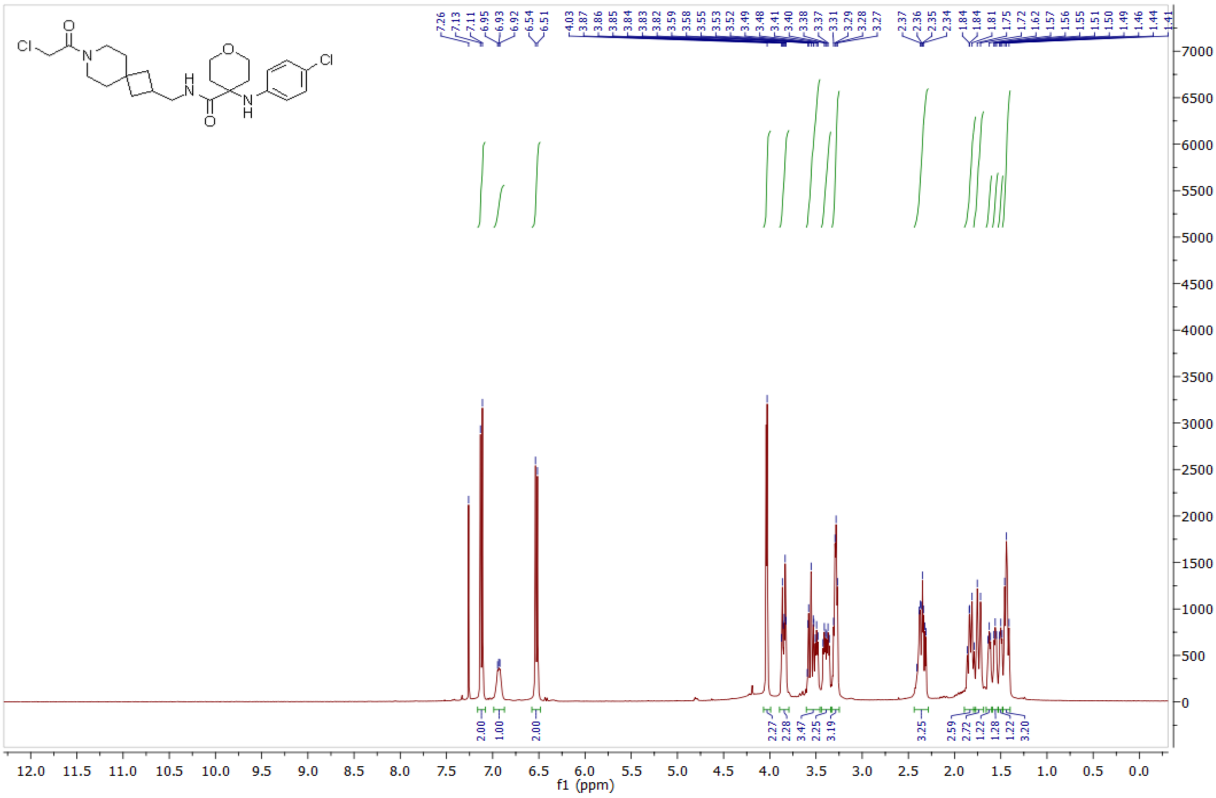

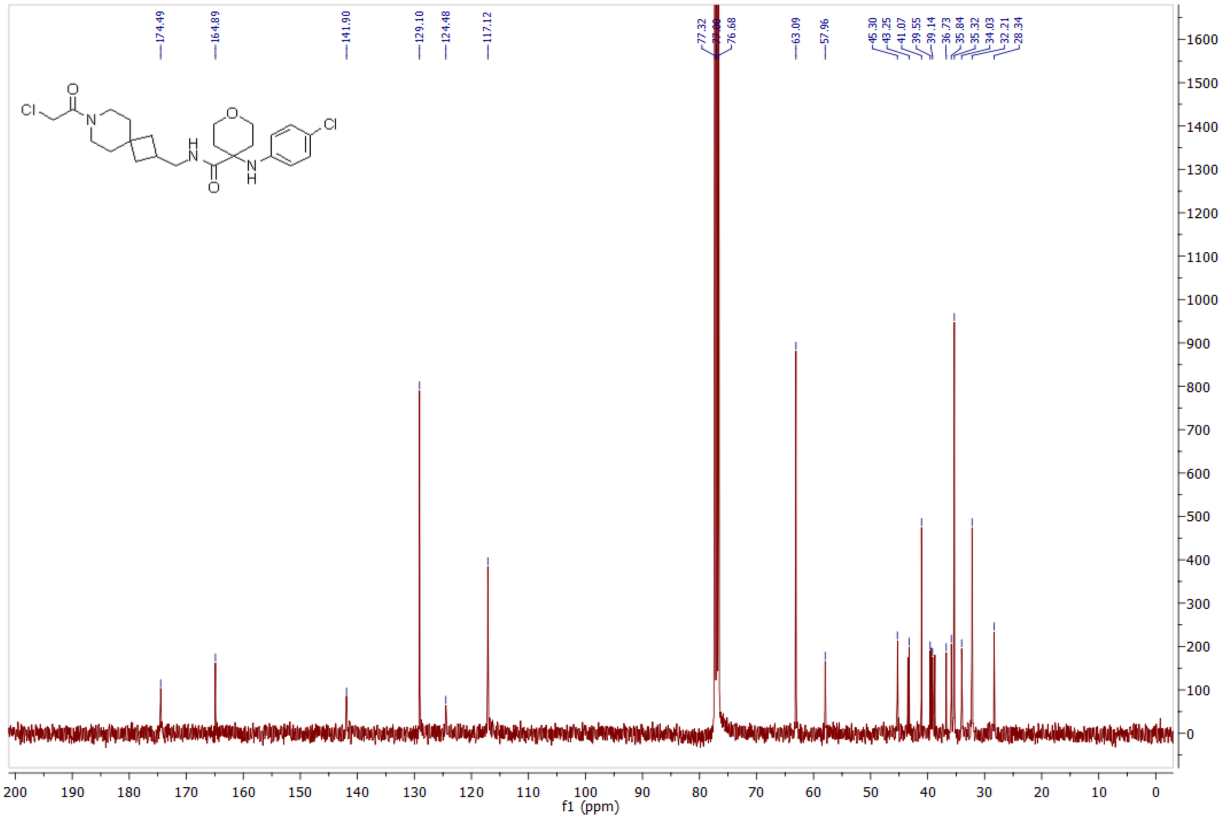

N-((7-(2-chloroacetyl)-7-azaspiro[3.5]nonan-2-yl)methyl)-4-((4-chlorophenyl)amino)-2,6-dimethyltetrahydro-2H-pyran-4-carboxamide **(180) 1083743**

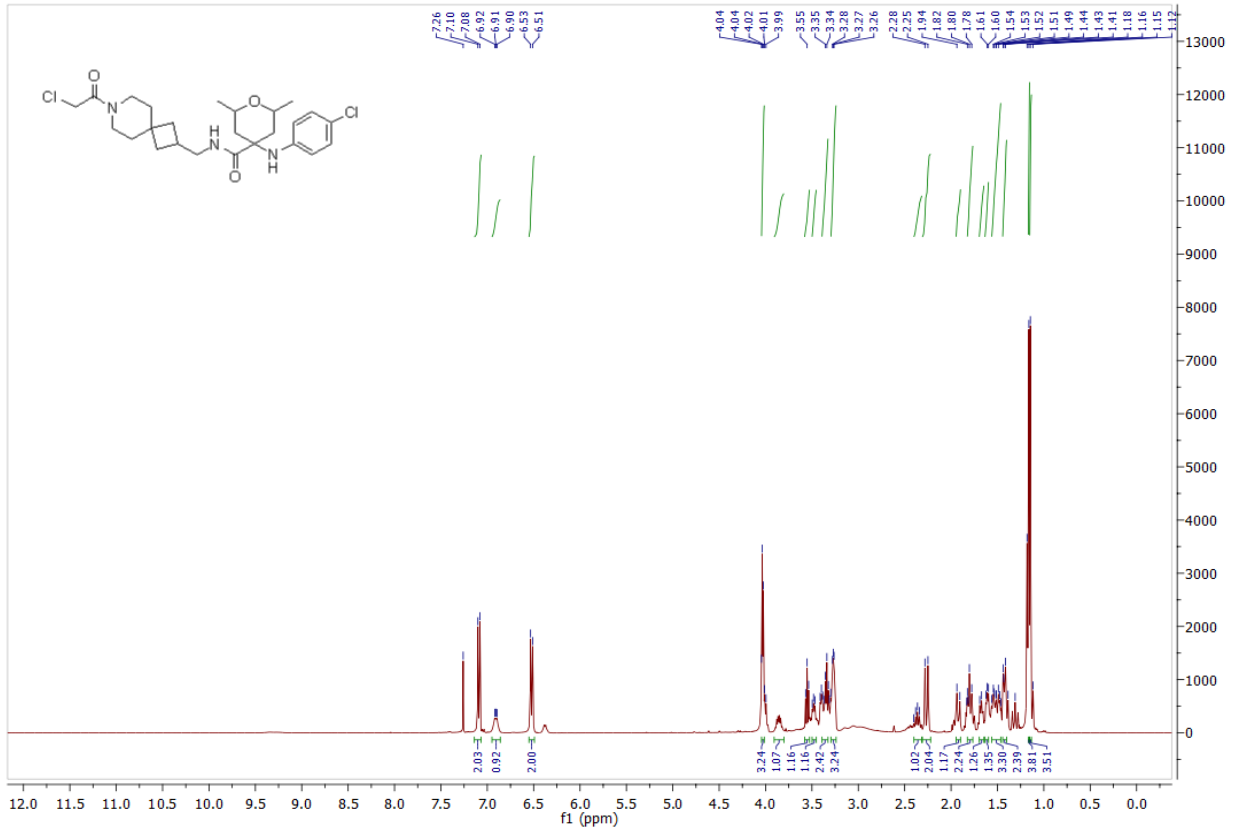

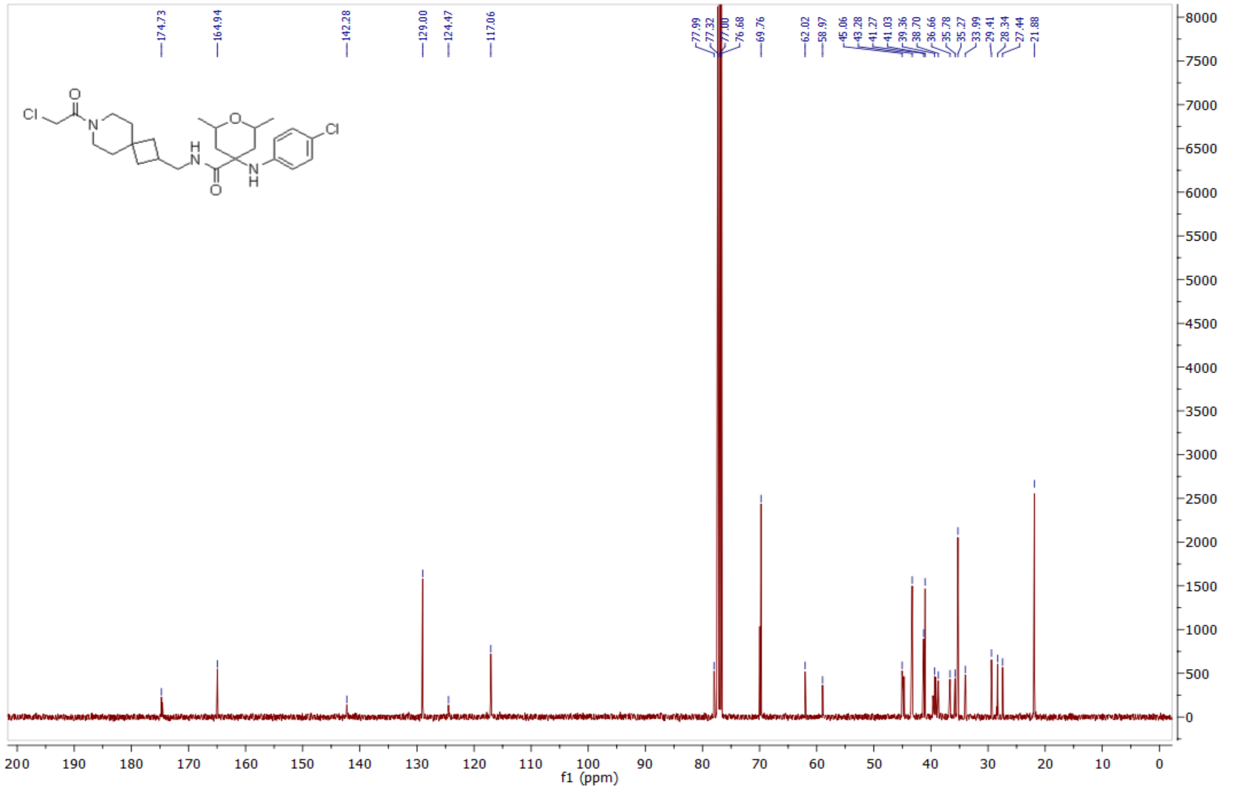

2-chloro-1-(9-(4-((4-chlorophenyl)amino)-2,6-dimethyltetrahydro-2H-pyran-4-carbonyl)-3,9-diazaspiro[5.5]undecan-3-yl)ethan-1-one (**181) 1083744**

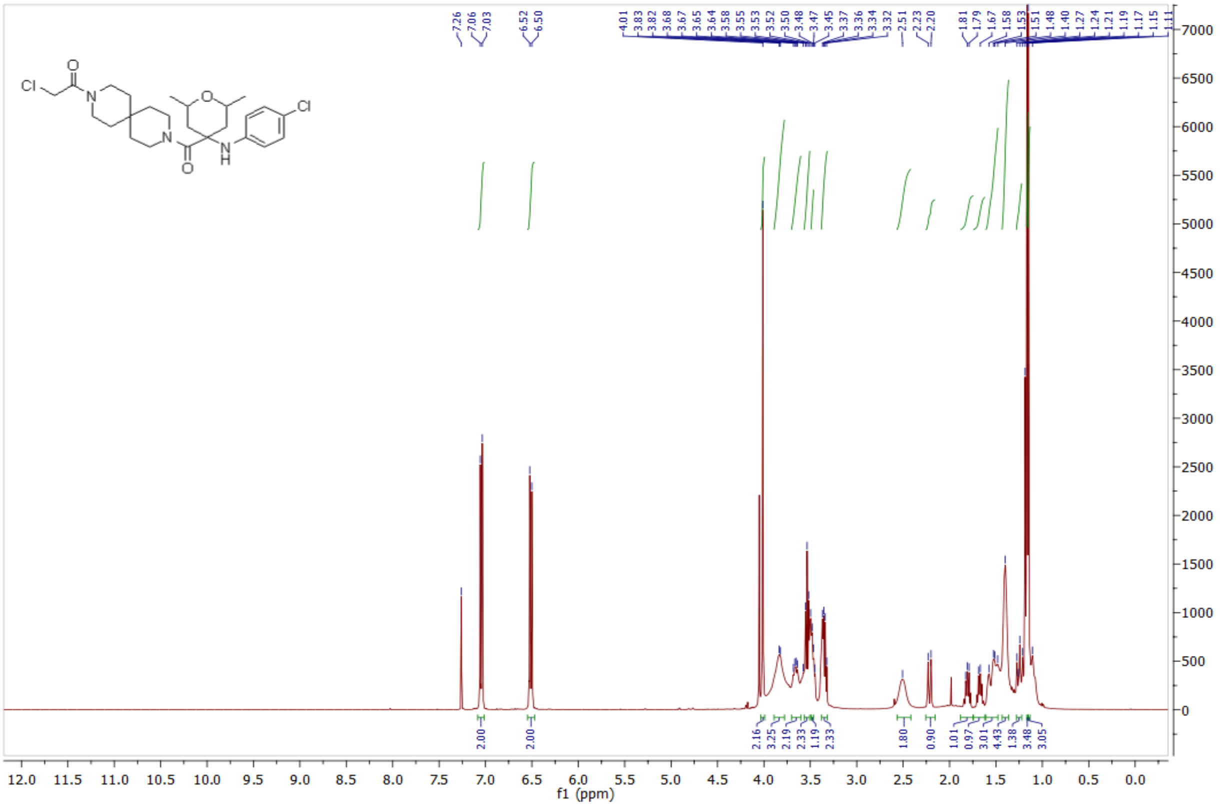

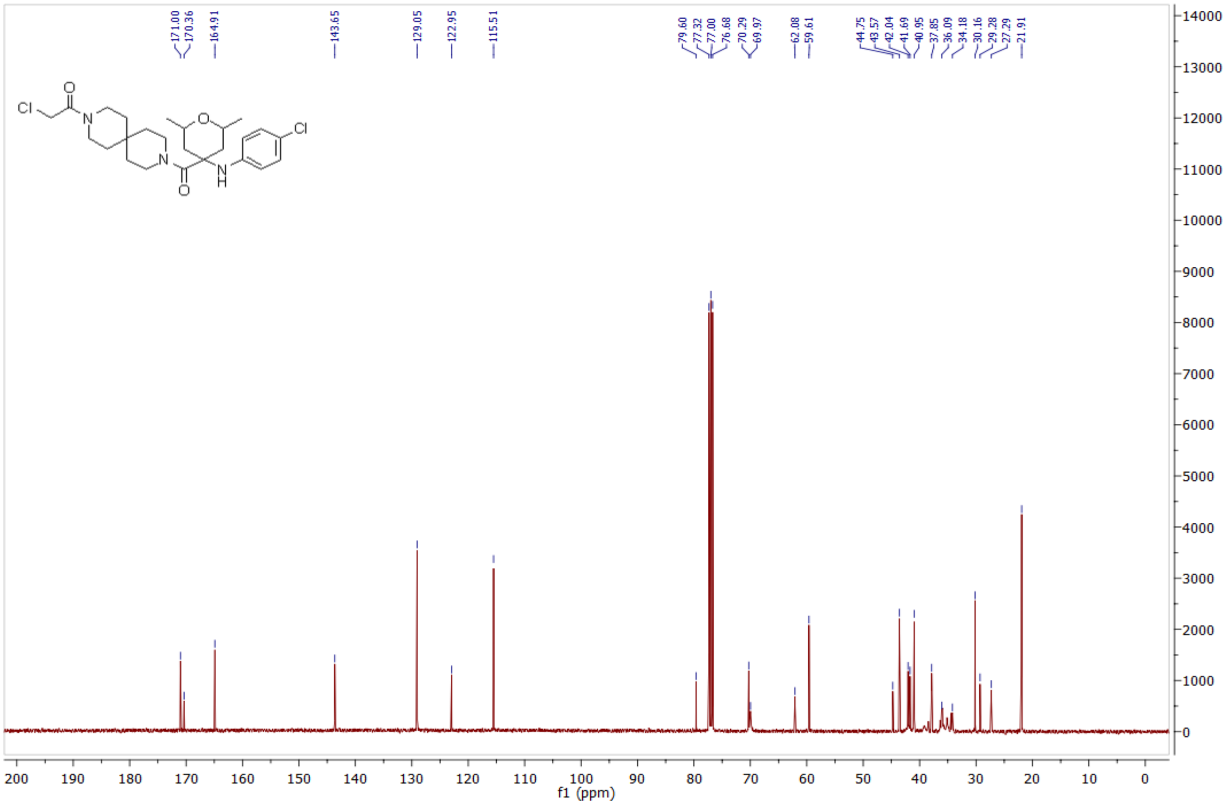

6. References

(1) Hallenbeck, K. K.; Davies, J. L.; Merron, C.; Ogden, P.; Sijbesma, E.; Ottmann, C.; Renslo, A. R.; Wilson, C.; Arkin, M. R. A Liquid Chromatography/Mass Spectrometry Method for Screening Disulfide Tethering Fragments. *SLAS Discov.* **2018**, *23* (2), 183–192. https://doi.org/10.1177/2472555217732072.

(2) Clabbers, M. T. B.; Gruene, T.; Parkhurst, J. M.; Abrahams, J. P.; Waterman, D. G. Electron Diffraction Data Processing with *DIALS*. *Acta Crystallogr. Sect. Struct. Biol.* **2018**, *74* (6), 506–518. https://doi.org/10.1107/S2059798318007726.

(3) Potterton, L.; Agirre, J.; Ballard, C.; Cowtan, K.; Dodson, E.; Evans, P. R.; Jenkins, H. T.; Keegan, R.; Krissinel, E.; Stevenson, K.; Lebedev, A.; McNicholas, S. J.; Nicholls, R. A.; Noble, M.; Pannu, N. S.; Roth, C.; Sheldrick, G.; Skubak, P.; Turkenburg, J.; Uski, V.; von Delft, F.; Waterman, D.; Wilson, K.; Winn, M.; Wojdyr, M. *CCP* 4 *i* 2: The New Graphical User Interface to the *CCP* 4 Program Suite. *Acta Crystallogr. Sect. Struct. Biol.* **2018**, *74* (2), 68–84. https://doi.org/10.1107/S2059798317016035.

(4) Evans, P. R.; Murshudov, G. N. How Good Are My Data and What Is the Resolution? *Acta Crystallogr. D Biol. Crystallogr.* **2013**, *69* (7), 1204–1214. https://doi.org/10.1107/S0907444913000061.

(5) Evans, P. R. An Introduction to Data Reduction: Space-Group Determination, Scaling and Intensity Statistics. *Acta Crystallogr. D Biol. Crystallogr.* **2011**, *67* (4), 282–292. https://doi.org/10.1107/S090744491003982X.

(6) Vagin, A.; Teplyakov, A. Molecular Replacement with *MOLREP*. *Acta Crystallogr. D Biol. Crystallogr.* **2010**, *66* (1), 22–25. https://doi.org/10.1107/S0907444909042589.

(7) Emsley, P.; Lohkamp, B.; Scott, W. G.; Cowtan, K. Features and Development of *Coot*. *Acta Crystallogr. D Biol. Crystallogr.* **2010**, *66* (4), 486–501. https://doi.org/10.1107/S0907444910007493.

(8) Long, F.; Nicholls, R. A.; Emsley, P.; Gražulis, S.; Merkys, A.; Vaitkus, A.; Murshudov, G. N. *AceDRG* : A Stereochemical Description Generator for Ligands. *Acta Crystallogr. Sect. Struct. Biol.* **2017**, *73* (2), 112–122. https://doi.org/10.1107/S2059798317000067.

(9) Moriarty, N. W.; Grosse-Kunstleve, R. W.; Adams, P. D. *Electronic Ligand Builder and Optimization Workbench* (*ELBOW*): A Tool for Ligand Coordinate and Restraint Generation. *Acta Crystallogr. D Biol. Crystallogr.* **2009**, *65* (10), 1074–1080. https://doi.org/10.1107/S0907444909029436.

(10) Afonine, P. V.; Grosse-Kunstleve, R. W.; Echols, N.; Headd, J. J.; Moriarty, N. W.; Mustyakimov, M.; Terwilliger, T. C.; Urzhumtsev, A.; Zwart, P. H.; Adams, P. D. Towards Automated Crystallographic Structure Refinement with *Phenix.Refine*. *Acta Crystallogr. D Biol. Crystallogr.* **2012**, *68* (4), 352–367. https://doi.org/10.1107/S0907444912001308.

(11) Adams, P. D.; Afonine, P. V.; Bunkóczi, G.; Chen, V. B.; Davis, I. W.; Echols, N.; Headd, J. J.; Hung, L.-W.; Kapral, G. J.; Grosse-Kunstleve, R. W.; McCoy, A. J.; Moriarty, N. W.; Oeffner, R.; Read, R. J.; Richardson, D. C.; Richardson, J. S.; Terwilliger, T. C.; Zwart, P. H. *PHENIX* : A Comprehensive Python-Based System for Macromolecular Structure Solution. *Acta Crystallogr. D Biol. Crystallogr.* **2010**, *66* (2), 213–221. https://doi.org/10.1107/S0907444909052925.

(12) Murshudov, G. N.; Skubák, P.; Lebedev, A. A.; Pannu, N. S.; Steiner, R. A.; Nicholls, R. A.; Winn, M. D.; Long, F.; Vagin, A. A. *REFMAC* 5 for the Refinement of Macromolecular Crystal Structures. *Acta Crystallogr. D Biol. Crystallogr.* **2011**, *67* (4), 355–367. https://doi.org/10.1107/S0907444911001314.

(13) Joosten, R. P.; Long, F.; Murshudov, G. N.; Perrakis, A. The *PDB_REDO* Server for Macromolecular Structure Model Optimization. *IUCrJ* **2014**, *1* (4), 213–220. https://doi.org/10.1107/S2052252514009324.

(14) Karplus, P. A.; Diederichs, K. Linking Crystallographic Model and Data Quality. *Science* **2012**, *336* (6084), 1030–1033. https://doi.org/10.1126/science.1218231.

(15) Sharma, K. K.; Mandloi, M.; Rai, N.; Jain, R. Copper-Catalyzed N-(Hetero)Arylation of Amino Acids in Water. *RSC Adv.* **2016**, *6* (99), 96762–96767. https://doi.org/10.1039/C6RA23364C.
(16) Sharma, K. K.; Sharma, S.; Kudwal, A.; Jain, R. Room Temperature N-Arylation of Amino Acids and Peptides Using Copper(I) and β-Diketone. *Org. Biomol. Chem.* **2015**, *13* (16), 4637–4641. https://doi.org/10.1039/C5OB00288E.
(17) Butcher, K. J.; Hurst, J. Aromatic Amines as Nucleophiles in the Bargellini Reaction. *Tetrahedron Lett.* **2009**, *50* (21), 2497–2500. https://doi.org/10.1016/j.tetlet.2009.03.044.
